# Investigation of cuproptosis regulator-mediated modification patterns and SLC30A7 function in GBM

**DOI:** 10.18632/aging.205545

**Published:** 2024-02-22

**Authors:** Wanli Yu, Shikai Gui, Jiabao Xie, Lunshan Peng, Juexian Xiao, Haitao Luo, Zhennan Tao, Zujue Cheng

**Affiliations:** 1 Department of Neurosurgery, The Second Affiliated Hospital, Jiangxi Medical College, Nanchang University, Jiangxi 330006, China; 2 Institute of Neuroscience, Nanchang University, Nanchang, Jiangxi 330036, China; 3 Department of Neurosurgery, Affiliated Drum Tower Hospital, School of Medicine, Nanjing University, Nanjing 210008, China

**Keywords:** GBM, cuproptosis, tumour microenvironment, ATP7A, SLC30A7

## Abstract

Background: Copper-dependent controlled cell death (cuproptosis) is a novel cell death modality that is distinct from known cell death mechanisms. Nonetheless, the potential role of the cuproptosis regulator in tumour microenvironment (TME) of GBM remains unknown.

Methods: Based on 13 widely recognised cuproptosis regulators, the cuproptosis regulation patterns and the biological characteristics of each pattern were comprehensively assessed in GBMs. Machine learning strategies were used to construct a CupScore to quantify the cuproptosis regulation patterns of individual tumours. A PPI network was constructed to predict core-associated genes of cuproptosis regulators. The function of the novel cuproptosis regulators SLC30A7 was examined by *in vitro* and *in vivo* experiment.

Results: We identified three distinct cuproptosis regulation patterns, including immune activation, metabolic activation, and immunometabolic double deletion patterns. The CupScore was shown to predict the abundance of tumour inflammation, molecular subtype, stromal activity, gene variation, signalling pathways, and patient prognosis. The low CupScore subtype was characterised by immune activation, isocitrate dehydrogenase mutations, sensitivity to chemotherapy, and clinical benefits. The high CupScore subtype was characterised by activation of the stroma and metabolism and poor survival. Novel cuproptosis regulator SLC30A7 knockdown inhibited the cuproptosi via JAK2/STAT3/ATP7A pathway in GBM.

Conclusion: Cuproptosis regulators have been shown to play a vital role in TME complexity. Constructing CupScores were trained to evaluate the regulation patterns of cuproptosis in individual tumours. The novel cuproptosis-related genes SLC30A7 was involved in regulation the tumorigenicity of GBM cell via JAK2/STAT3/ATP7A pathway *in vitro* and *in vivo*.

## INTRODUCTION

Multicellular organisms present different controlled programmed cell death modalities such as apoptosis, necroptosis, pyroptosis, and ferroptosis [1]. Among these, ferroptosis, a novel cell death modality named in 2012, has recently become a research focus [2]. Similar to iron, copper is an indispensable microelement in living organisms and usually is present at extremely low levels in mammalian cells. Cellular copper ions can also exhibit cytotoxicity when their concentration exceeds a threshold for maintaining homeostatic mechanisms [3]. In March 2022, Tsvetkov et al. demonstrated a novel copper-dependent controlled cell death modality in human cells, named cuproptosis, which is distinct from known cell death mechanisms [4]. The team further unravelled the mechanism of cuproptosis, which occurs through the direct binding of copper ions to lipoylated components of the tricarboxylic acid cycle (TCA) in mitochondrial respiration, leading to lipoylated protein aggregation and subsequent loss of Fe-S cluster proteins, resulting in proteotoxic stress and ultimately cell death. More importantly, this study also identified 13 key genes affecting cuproptosis, including the *FDX1* gene encoding an elesclomol molecular target protein and six genes involved in mitochondrial metabolism (*LIPT1, LIAS*, and *DLD*) and protein lipoylation modification (*DLAT*, *PDHA1*, and *PDHB*). Four protein lipoylation enzymes (DBT, GCSH, DLST, and DLAT) are involved in metabolic complexes that regulate carbon entry into the TCA cycle. In addition, DLAT is an essential component of the Pyruvate dehydrogenase (PDH) complex. The three copper ionophores include the copper importer SLC31A1 (CTR1) and copper exporters ATP7A and ATP7B. An in-depth understanding of these regulators would help reveal the mechanism of copper dysregulation syndromes, including Menke’s and Wilson’s diseases [5, 6]. Studies have confirmed that high levels of FDX1 and protein lipoylation promote cell death induced by copper ionophores [4]. Thus, cuproptosis regulatory factors may be potential therapeutic targets in cancer cells.

Targeted therapy has shown striking clinical efficacy in patients with several solid tumours such as lung and breast cancers. Unfortunately, the clinical benefits for most patients with glioblastoma multiforme (GBM) are minimal or have no clinical benefit, which is far from a met clinical need [7]. The new metabolic mode of cell death, cuproptosis, has brought dawn to the treatment of GBM [8]. The intractability of tumours is largely attributed to their unique metabolic and immunological modalities [9]. Malignant cells proliferate rapidly and survive in harsh environments by reprogramming their metabolism and energy production [10]. The microenvironment in which tumour cells depend for growth and survival also plays a crucial role in tumour progression [11]. Cancer cell interactions with tumour microenvironment (TME) components result in multiple biological behavioural changes, such as proliferation and invasion, metabolic alterations, inhibition of apoptosis, and immune escape [12]. Recently, evidence suggested that cuproptosis contributes toward promoting tumorigenesis and remodeling of TME [13–15]. Copper combined with αPD-L1 enhances cancer immunotherapy [16]. A study describes the overall characteristic immune cell infiltration of the TME mediated by the interconnected functions of multiple regulators of cuproptosis in pan-cancer [17]. Therefore, characterisation of TME cell infiltration driven by cuproptosis regulators will enhance our understanding of the antitumour response of TME components and benefit the search for immunotherapy strategies.

In this study, cuproptosis regulation patterns and TME cell-infiltrating characteristics of each pattern were identified in 469 patients with GBM. Three distinct cuproptosis regulation phenotypes were identified: immune activation, metabolic activation, and immunometabolic double deletion. Interestingly, we discovered that the metabolic activation phenotype was a cuproptosis phenotype. Moreover, we established the cuproptosis score (CupScore) to comprehensively evaluate the cuproptosis regulation pattern in individual tumours. These findings suggest that cuproptosis regulation plays a vital role in the TME. Interestingly, we found that the novel cuproptosis-related genes SLC30A7 was involved in regulating the cuproptosis of GBM cell through the JAK2/STAT3/ATP7A pathway. Thus, SLC30A7 may be valuable in directing therapeutic intervention plans for GBM.

## METHODS

### Acquisition and processing of GBM expression datasets

The workflow of this study is shown in Supplementary Figure 1A. Gene expression data and clinical features of GBM samples were obtained from the Gene Expression Omnibus (GEO; https://www.ncbi.nlm.nih.gov/geo/), ArrayExpress (https://www.ebi.ac.uk/arrayexpress/), Chinese Glioma Genome Atlas (CGGA; http://www.cgga.org.cn/), and The Cancer Genome Atlas (TCGA; http://cancergenome.nih.gov/) databases. In total, six eligible GBM cohorts with survival information (GSE7696, GSE16011, GSE108474, ArrayExpress-E-TABM-898, CGGA-GBM, and TCGA-GBM) were included for further analysis. The “affy” package was performed for background adjustment and quantile normalisation of GEO, ArrayExpress, and TCGA databases. Copy number aberrations and somatic mutation data from TCGA-GBM were downloaded from the Broad Institute (https://www.broadinstitute.org/). TCGA RNA-seq data (FPKM value) downloaded from the Genomic Data Commons (GDC, https://portal.gdc.cancer.gov/) were transformed into transcripts per kilobase million (TPM) values [18]. The ComBat algorithm from the ‘SVA’ R package was used to correct the non-biological technical biases among the different datasets. All the eligible GBM datasets with complete information are listed in Supplementary Table 1.

### Consensus clustering of cuproptosis regulation patterns

A total of 13 cuproptosis regulators were extracted from four integrated datasets (GSE7696, GSE16011, GSE108474, and ArrayExpress-E-TABM-898) to distinct different cuproptosis modification patterns using the ConsensusClusterPlus package. These 13 cuproptosis regulators included mitochondrial metabolism genes (LIPT1, LIAS, and DLD), protein lipoylation modification genes (DLAT, PDHA1, and PDHB), protein lipoylation enzymes (DBT, GCSH, DLST, and DLAT), copper importer SLC31A1 (CTR1), and copper exporters ATP7A and ATP7B. A consensus clustering algorithm was used to determine the number of clusters and 1000 iterations to ensure the stability of the classification [19]. The consensus cumulative distribution function (CDF), consensus matrix (CM), and consensus heatmap were performed to determine the optimal number of clusters. The principle of unsupervised clustering analysis is described [19, 20], including how to assign each sample to a cluster.

### Gene set variation analysis (GSVA) and functional annotation

GSVA analysis was used to investigate the variation in biological processes between different cuproptosis regulation patterns with the R package ‘GSVA’. Gene sets of “c2.cp.kegg. v7.5.1. symbols” were derived from the MSigDB database with a false discovery rate cutoff value of < 0.01. The “clusterProfiler” package was used to explore Gene Ontology (GO) and Kyoto Encyclopedia of Genes and Genomes (KEGG) enrichment analyses of differentially expressed genes (DEGs) between different cuproptosis regulation patterns.

### Estimation of TME cell infiltration

We quantified the relative abundance of immune cell infiltration in the GBM tumour microenvironment using single-sample gene set enrichment analysis (ssGSEA). The enrichment score in the ssGSEA analysis, which represents cell infiltration, was normalised to a unity distribution from 0 to 1. The abundance of 22 distinct leukocyte subsets with the gene expression profile of GBM was evaluated using CIBERSORT 44.

### DEG analysis

The “limma” package of R software was used to identify the DEGs among the cuproptosis clusters based on the expression of 13 cuproptosis regulators. The cutoff criteria were set as | log2 fold change | >0.7 and *p* < 0.05.

### Development of the CupScore

A cuproptosis scoring scheme was established to quantify the cuproptosis regulation patterns of individual patients using principal component analysis (PCA). The procedures for establishment of cuproptosis gene signature (termed as CupScore) were as follows:

The limma R package was used to identify DEGs between different modification patterns, and the overlap genes from different cuproptosis clusters were extracted. The criteria for identifying DEGs was set as adjusted *P*-value < 0.001. The overlap DEGs were applied to classify patients into several groups using unsupervised clustering method. The consensus clustering algorithm was used to determine the number of gene clusters and their stability. DEGs were used for prognostic analysis using a univariate Cox regression model (*P*-value < 0.05). Then, the genes with the significant prognosis were performed to construct cuproptosis gene signature using principal component analysis (PCA) method. This method concentrated on the score of a set comprising the most significantly associated genes and involved scaling down the score of genes that were not tracked to other members of the set. The CupScore, which is described according to a GGI-like procedure [21], was calculated as follows: CupScore = ∑(*PC*1*i*+*PC*2*i*), and the “*i*” is the expression of cuproptosis phenotype-related genes.

### Correlation analysis of CupScore and other biological pathways or clinical information

Correlation analysis was performed to deeply explore the correlation between the cuproptosis gene signature and other related biological processes, including (1) angiogenesis; (2) antigen processing machinery; (3) CD8 T effector; (4) cell cycle; (5) DNA damage repair; (6) DNA replication; (7) epithelial-mesenchymal transition (EMT1), EMT2, and EMT3; (8) FGFR3-related genes; (9) immune checkpoint; (10) mismatch repair; (11) nucleotide excision repair; (12) pan-fibroblast TGF-β response signature; and (13) WNT target [22].

We further analysed the relationship between the CupScore and other related clinical information, including age, sex, isocitrate dehydrogenase (IDH), 06- methylguanine DNA methyltransferase, molecular subtype, temozolomide (TMZ), and tumour somatic mutation.

### Construction of a protein-protein interaction (PPI) network

The mRNAs were included in a PPI network using the STRING database (https://string-db.org/) with a confidence score of >0.7. Cytoscape (version 3.8.1) was used to visualise the PPI network [7].

### Cell culture

Glioma primary cells (HG6, HG9) were obtained from tumour tissue of GBM patients in the Department of Neurosurgery, Affiliated Drum Tower Hospital, School of Medicine, Nanjing University. Primary cell line was cultured in DMEM medium mixed with 10% fetal bovine serum.

### Chemical reagents, antibodies, and transfection

The JAK2/STAT3 inhibitor WP1066 was purchased from Selleck Chemicals (Boston, MA, USA). Antibodies for p-JAK, JAK, p-STAT3, STAT3, ATP7A were purchased from Proteintech Group (Wuhan, China). SLC30A7 overexpression plasmids and short hairpin RNA (shRNA) were produced by GV112 vector (hU6-MCS-CMV-Puromycin; GeneChem, China). On the basis of the manufacturer’s recommendation, lentiviral vectors expressing shRNA or scrambled transfected into cells. Steady cell clones transfected with shRNA expressing constructs were chosen with puromycin intervention after infection.

### Immunohistochemistry

The antibiotin protein-biotin method got accustomed to performing immunostaining on paraffin-embedded sections. Slides were dewaxed in xylene, then rehydrated in graded ethanol, then the endogenous peroxidase activity was then quenched with 0.3% hydrogen peroxide (China), and the strong antigen recovery solution was heated to 37°C to recover the antigen. 5% goat serum (Solarbio, China) was used to block nonspecific proteins. Primary antibodies (1:100 dilutions) were used to incubate sections at 4°C overnight, subsequently the appropriate biotinylated secondary antibody was added (1:100 dilutions) (ZSGBBio, China) at 37 °C for 60 minutes. The, slides were then hatched with ABC peroxidase and diaminobenzidine (ZSGBBio, China). Next, the slides were counter-stained for nuclear staining by Mayer hematoxylin solution (Solarbio, China). For going on H&E staining, the slides were deparaffinized and rehydrated. Next, the slides were stained by nuclear staining, subsequently re-staining using HE kit (Solarbio, China). The images were taken with an inverted microscope (Olympus, Japan). The human tissue samples used in this study research has complied with the relevant national and institutional policies.

### Colony formation assay

For the colony formation assay, transfected cells (1 × 10^3^ cells/well) were cultured in 12-well plate. The cells were fixed with methanol and stained with 0.4% crystal violet solution when number of colonies more than 50 cells.

### Transwell migration assay

To evaluate cell migratory ability, Transwell assays were performed in 24-well cell culture chambers with 8 mm pore Transwell inserts precoated with Matrigel. In brief, cells were seeded in 200 μL of culture medium containing 1% FBS. Five hundred microliters of medium containing 50% FBS was added to each lower chamber. After 24 h, T cells migrating through the membrane of Transwell inserts were stained with crystal violet and photographed by microscopy.

### Western blot

For Western blot, after the cell protein sample was extracted, the protein concentration was detected by the BCA kit (Beyotime, China) and balanced, and a 1/3 proportion of loading Buffer was added for high-temperature (100°C) denaturing for 15 minutes. Then, the samples were added to the glue-plate sample adding tank. Electrophoresis was carried out at constant voltage for about 90 minutes, and then membrane was carried out at constant current for about 90 minutes. Then, it was sealed with milk, incubated with primary antibody at 4°C overnight, and then exposed after incubation with secondary antibody the next day.

### Nude mouse tumour model

Primary cells were used to establish a model of intracranial tumour in Female nude mice, refer to previous studies for details [23]. The cells stably transfected with SLC30A7 related lentivirus were divided into three groups as Scramble, shSLC30A7-1 and SLC30A7-2. These cells that had been transfected with luciferase encoding lentivirus (GeneChem, China) were stereotaxically injected into the intracranial of mice (*n* = 6 in each group) to establish tumour models. At day 7, 14, and 28, intracranial tumour size was assessed using the IVIS spectral real-time imaging system (Blandford, USA). For HE, mice brains were immobilized in 4% paraformaldehyde and then embedded in paraffin.

### Statistical analysis

Statistical analyses were performed using R, version 3.6.1. Student’s *t*-test was used to evaluate the statistical significance of normally distributed variables, and Wilcoxon rank-sum test analysis was performed for non-normally distributed variables. Comparisons of ≥2 groups were conducted using analysis of variance and Kruskal-Wallis tests [24]. The correlation between two groups was determined using the Pearson correlation analysis. The chi-square test or Fisher’s exact test was used for the statistical analysis of clinical information and gene clusters. Kruskal-Wallis and log-rank tests were used to analyse the association between the cuproptosis regulation pattern and prognosis. Univariate and multivariate analyses were used to establish a Cox proportional hazards regression model. A receiver operating characteristic (ROC) curve was used to evaluate the power of the CupScore model. The area under the curve was calculated using ‘timeROC’ package. Waterfall function was performed to show the mutation landscape in high- and low-CupScore patients with GBM using the maftools package in TCGA. The landscape of the copy number variation (CNV) of 13 cuproptosis regulators in 23 pairs of chromosomes was plotted using “RCircos” package. Statistical significance was set at *P* < 0.05. The bar chart is represented by mean standard deviation from at least three experimental replicates. Most of the experiments were statistically analyzed using Student’s *t* test. One-way analysis of variance (ANOVA) followed by Tukey’s post hoc test was used to assess differences between groups. The data were analyzed by graph pad prism 6. Significance of *p*-values were set at ^NS^*P* > 0.05, ^*^P < 0.05, ^**^P < 0.01, ^***^P < 0.001, ^****^P < 0.0001.

### Limitations of the study

Our study only explored the biological characteristics and predictive ability of the constructing cuproptosis regulation patterns in GBMs, which is not universal for other tumours. In this study, there is insufficient evidence on the effectiveness and toxic side effects of therapeutic strategies based on targeting cuproptosis regulators or pathways, which still needs more research to investigate.

### Key points

1. Genomic information from eligible GBM cohorts was employed to comprehensively evaluate the biological characteristics of distinct cuproptosis regulation patterns.
2. Three distinct cuproptosis regulation patterns were identified to differ in immune infiltration, biological processes, and prognosis.
3. Three regulated genomic phenotypes (cuproptosis gene clusters A, B and C) were determined by clustering analysis of the phenotype-related DEGs.
4. Machine-learning strategies for constructing CupScores were trained to quantify cuproptosis regulation patterns of individual samples and perform a comprehensive analysis of the regulators’ genome.
5. The novel cuproptosis regulator SLC30A7 predicted by PPI network was involved in regulation the tumorigenicity of GBM cell through the JAK2/STAT3/ATP7A pathway *in vitro* and *in vivo*.

### Availability of data and materials

Clinical information and high-throughput sequencing-counts were retrieved from the GTEx, TCGA and CGGA data portal, which is a publicly available database.

## RESULTS

### Landscape of genetic and expression variation of cuproptosis regulators in GBM

**Figure 1:**
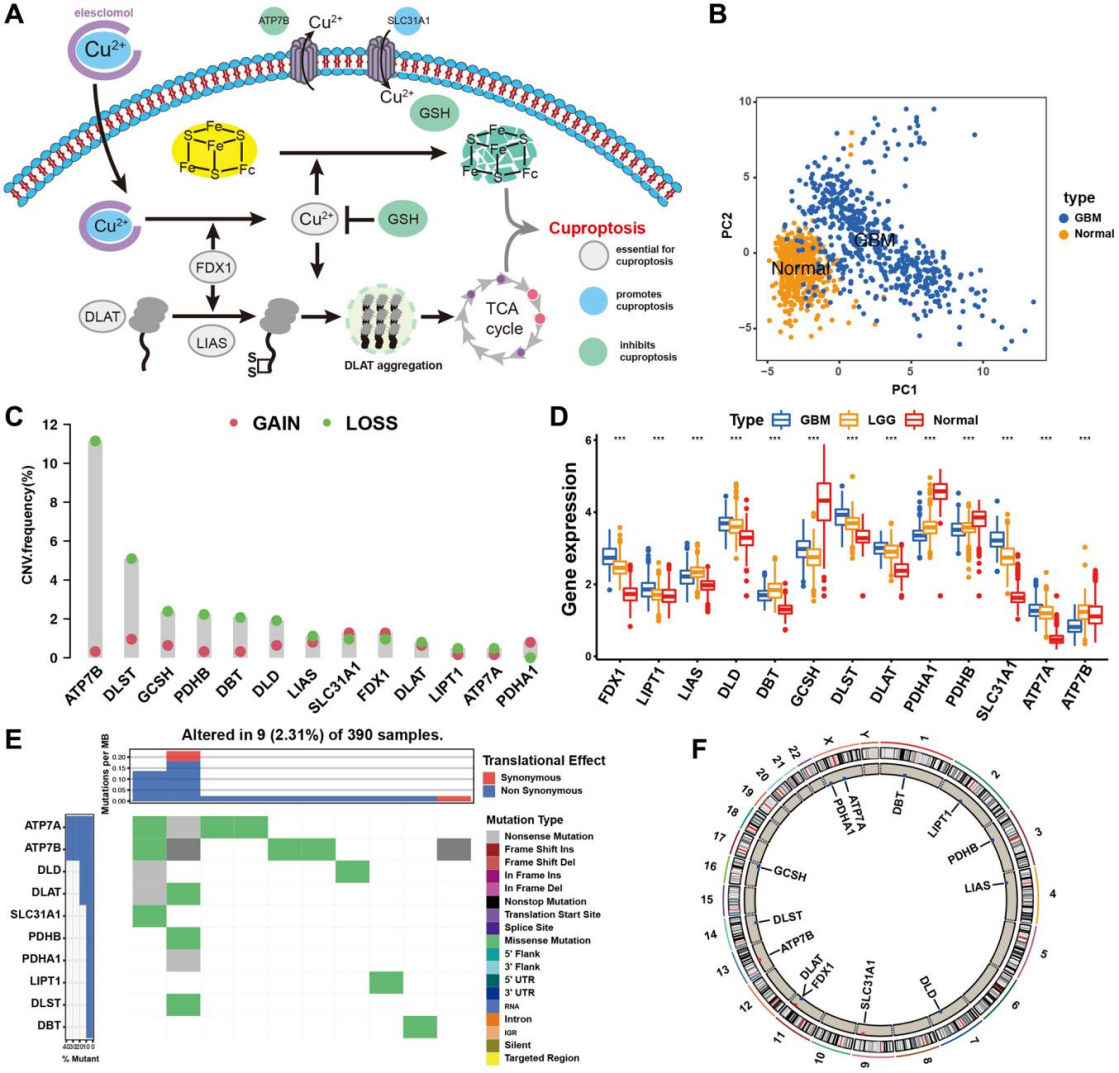
**Landscape of genetic and expression variation of cuproptosis regulators in GBM.** (**A**) Essential regulators in cuproptosis events, and their biological functions. (**B**) Principal component analysis of total 13 cuproptosis regulators for distinguishing tumour from normal patients in merge cohort (TCGA and GTEx). (**C**) The CNV mutation frequency of 13 cuproptosis regulators in TCGA cohort. The column represented the alteration frequency. The deletion frequency, green dot; The amplification frequency, red dot. (**D**) Differential expression of cuproptosis regulators between normal and GBM tissues. GBM, blue; LGG, yellow; Normal, red. Significant results are indicated as ^*^p < 0.05, ^**^p < 0.01, and ^***^p < 0.001. (**E**) The mutation frequency of key cuproptosis regulators in TCGA-GBM cohort. Each column represented individual patients. The upper barplot showed TMB. (**F**) The location of CNV alteration of cuproptosis regulators on chromosomes in TCGA-GBM cohort.

Thirteen known regulators of cuproptosis were analysed in this study. A schematic of the potential biological functions exploited by cuproptosis regulators to influence metabolic alterations is shown in Figure 1A. We found that the expression of these 13 cuproptosis regulators completely distinguished GBM samples from normal samples (Figure 1B). An investigation of the frequency of CNV alterations revealed the presence of CNV alterations in the 13 cuproptosis regulators. ATP7B, DLST, and GCSH showed copy number deletions, whereas SLC31A1 and FDX1 had CNV amplification frequencies (Figure 1C). As cuproptosis regulators with CNV amplification were significantly higher in GBM tissues (SLC31A1 and FDX1) and vice versa (ATP7B, DLST, and GCSH) than in normal brain tissues (Figure 1C, 1D), these genetic variations could be prominent factors altering the expression of cuproptosis regulators in patients with GBM. Analysis of the incidence of CNVs and somatic mutations of the 13 cuproptosis regulators in GBM revealed that these regulators were altered at a frequency of 2.31% (9 mutations) in the 390 samples. *ATP7A* exhibited the highest mutation frequency, followed by *ATP7B*, whereas *FDX1*, *LIAS*, and *GCSH* did not show any mutations in the GBM samples (Figure 1E). Further analyses revealed a significant mutation-negative relationship between *ATP7A* and *DLD* (Supplementary Figure 1B). The locations of CNV alterations in the 13 cuproptosis regulators on the chromosomes are shown in Figure 1F. The above analyses indicated significant differences and links in the genomic and transcriptomic landscapes of cuproptosis regulators between normal and GBM samples. Thus, altered expression and genetic variation of cuproptosis regulators play crucial roles in regulating GBM initiation and progression.

### Cuproptosis specific regulation patterns and biological characteristics of each pattern

**Figure 2:**
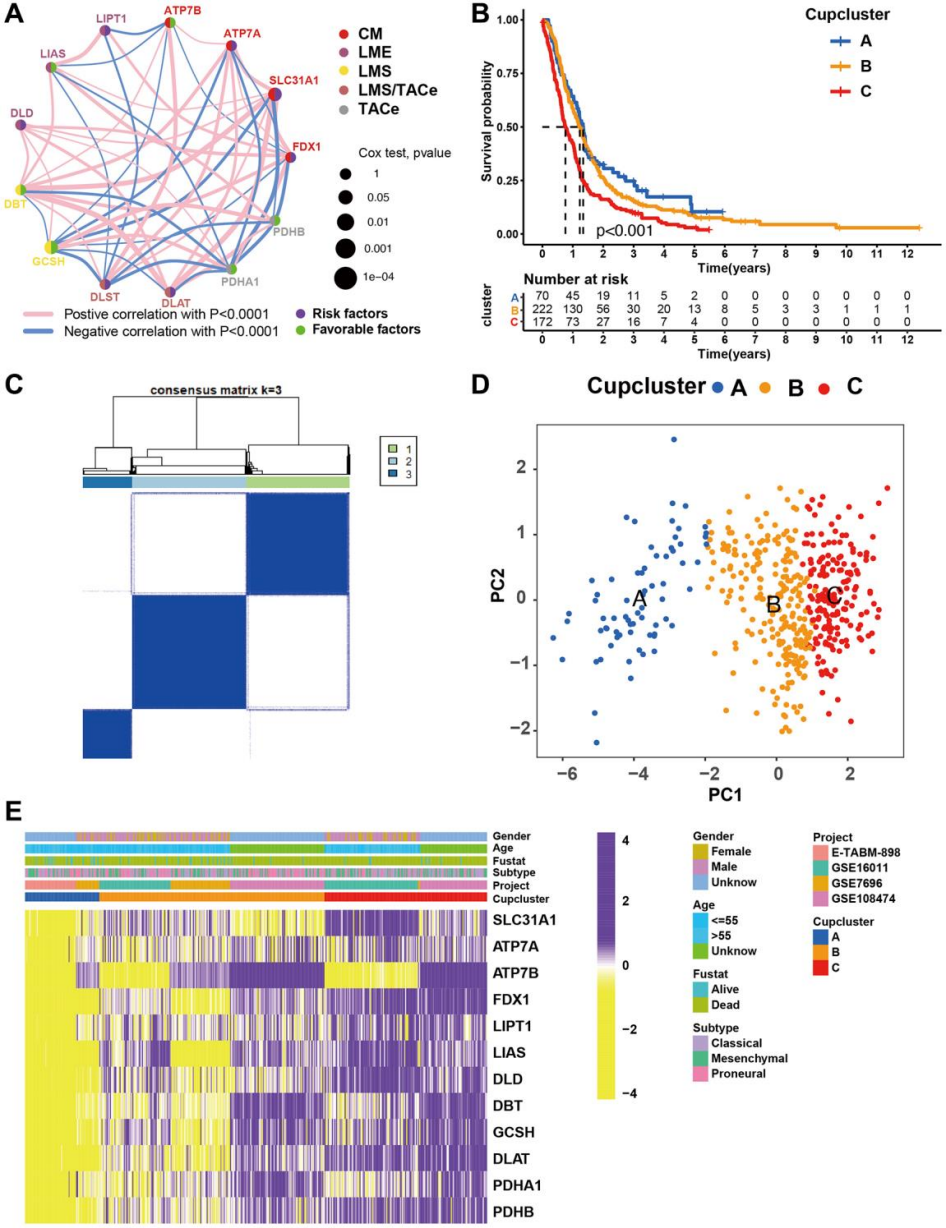
**Patterns of cuproptosis specific regulation.** (**A**) The interaction of expression on 13 cuproptosis regulators in GBM. Different biological functions of cuproptosis regulators were depicted by circles in different colors. The lines linking regulators showed their interactions, pink represented positive correlation, and blue represented negative correlation. The circle size represented the effect of each regulator on the prognosis by *P*-value. Purple dots in the circle showed risk factors of prognosis; Green dots in the circle showed favorable factors of prognosis. Abbreviations: CM: copper metabolism; LME: lipoylation modified enzyme; LMS: lipoylation modified substrates; TACe: tricarboxylic acid cycle enzymes. (**B**) Survival analyses of GBM (*n* = 469) with three cuproptosis regulation patterns; cuproptosis cluster A (*n* = 70), cuproptosis cluster B (*n* = 223), and cuproptosis cluster C (*n* = 176). Kaplan-Meier curves with *p* < 0.001 showed significant differences in OS among the three patterns. (**C**) Unsupervised clustering of 13 cuproptosis regulators in the GBM cohort (Consensus clustering matrix for k = 3). (**D**) Principal component analysis of GBM (*n* = 469, GSE7696, GSE16011, GSE108474, ArrayExpress-E-TABM-898), showing significant differences the transcriptome profiles of three cuproptosis regulation patterns. (**E**) Gene expression of overlapping cuproptosis regulators in GBM cohort (GSE7696, GSE16011, GSE108474, ArrayExpress-E-TABM-898). Cupcluster, gender, age, molecular subtypes, and survival status were used as patient annotations. Yellow: low expression; Purple: high expression.

Two datasets (TCGA and CGGA) with complete clinical information were included in this analysis. A univariate Cox regression model was used to determine the prognostic value of the 13 cuproptosis regulators in patients with GBM (Supplementary Figure 1C). Survival analysis suggested that 10 of the 13 regulators had significant survival effects in GBM (Supplementary Table 2). The cuproptosis regulator network revealed that cuproptosis regulators not only exhibited significant correlations in expression within the same functional category but also between CM, LME, LMS, and TACE (Figure 2A and Supplementary Table 3). We found that FDX1 positively correlated with the expression of SLC31A1, ATP7A, LIPT1, LIAS, DBT, and DLAT but negatively correlated with ATP7B, GCSH, and PDHA1, suggesting that FDX1 broadly influences the process of lipoylation modification of proteins. These results suggest that perturbing events among different classes of regulators may lead to the formation of different cuproptosis regulation patterns and characteristic TME alterations in GBM.

**Figure 3:**
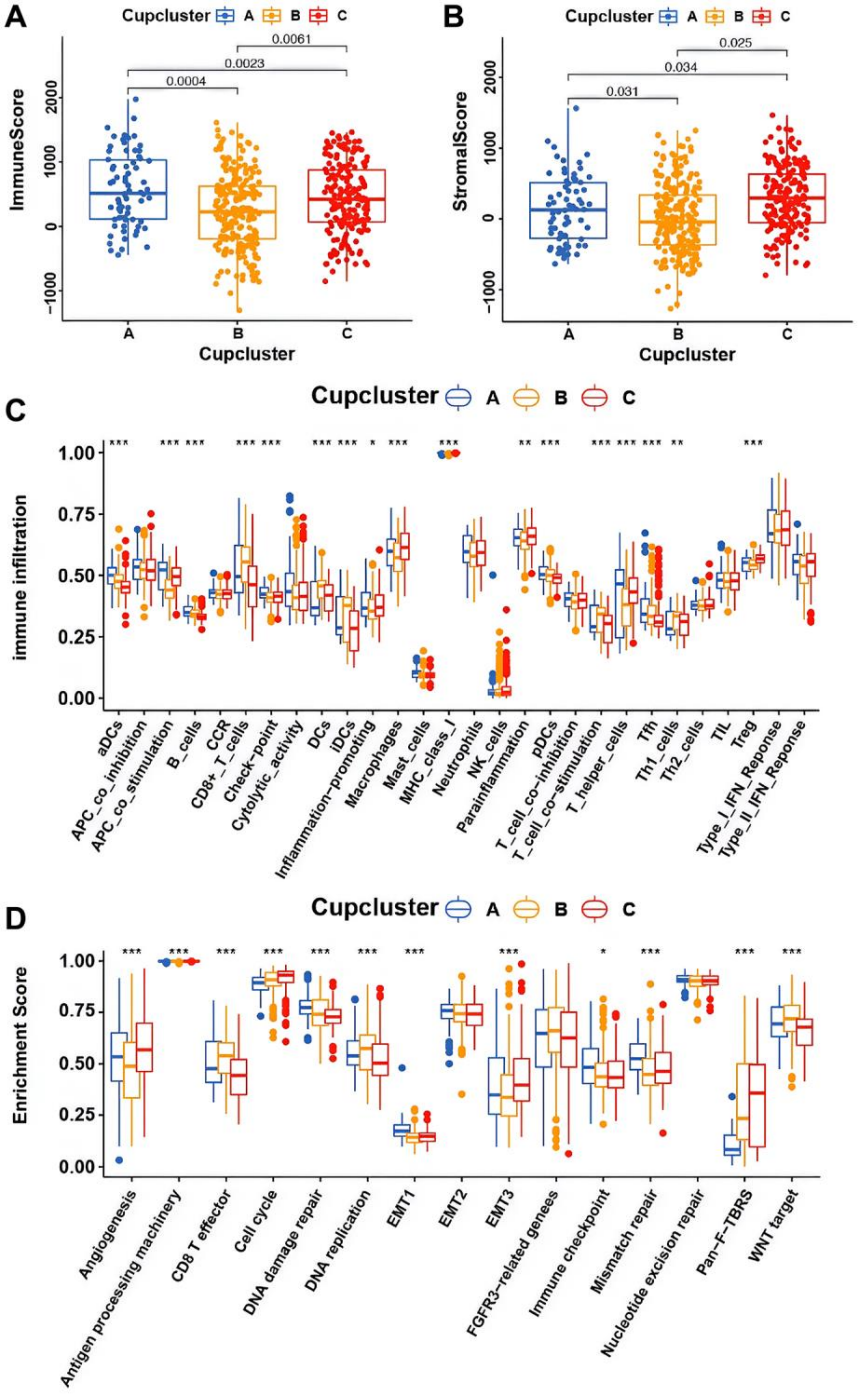
**Potential characteristics in distinct cuproptosis-related phenotypes.** (**A**) Differences in immune score among three cuproptosis regulation patterns in merge cohort (*P* < 0.05, Kruskal-Wallis test). (**B**) Differences in stromal score among three cuproptosis regulation patterns in merge cohort (*P* < 0.05, Kruskal-Wallis test). (**C**) The fraction of TME infiltrating cell in distinct cuproptosis regulation patterns using the CIBERSORT algorithm. The bottom and top of the boxes were interquartile range of values. The thick line in the boxes indicated median value. Significant results are indicated as ^*^p < 0.05, ^**^p < 0.01, and ^***^p < 0.001. (**D**) Differences of stroma-activated pathways in three cuproptosis regulation patterns. Significant results are indicated as ^*^p < 0.05, ^**^p < 0.01, and ^***^p < 0.001.

Four datasets (GSE7696, GSE16011, GSE108474, and ArrayExpress-E-TABM-898) were used to identify three distinct cuproptosis regulation patterns based on the 13 cuproptosis regulators using the unsupervised clustering method (Figure 2C). There was a significant difference in the cuproptosis transcriptional profile among the three cuproptosis regulation patterns (Figure 2D). Prognostic analysis revealed that among those in Cupcluster A, B, and C, patients in Cupcluster C showed the worst survival performance (*p* < 0.001; Figure 2B). Cuproptosis regulators differ significantly in three distinct modes of regulation. The regulators of cuproptosis were most highly expressed in Cupcluster C patients and least expressed in Cupcluster A patients (Figure 2E), which indicated that the Cupcluster C group may have an active cuproptosis phenotype. The results of the ESTIMATE algorithm showed that Cupcluster A exhibited a high immune score and that Cupcluster C had a high stromal score (Supplementary Table 4), which meant that Cupcluster A may have significantly increased immune cell infiltration, and Cupcluster C has a pro-tumorigenic phenotype (Figure 3A, 3B). Analyses of TME cell infiltration revealed that CupCluster A was significantly enriched in immune cell infiltration, including macrophages, dendritic cells, B cells, and antigen-presenting cell stimulation (Figure 3C). We hypothesize that the diverse immune cells enriched in Cupcluster A tumours may inhibit the initiation and progression of cancer, thereby exhibiting a distinct survival advantage. Immune cell-rich tumours have been shown to have distinct survival advantages [25–27]. A GSVA enrichment analysis was used to further explore the biological characteristics of each pattern (Supplementary Table 5). Cupcluster A showed enrichment in immune activation-related pathways such as antigen processing and presentation, extracellular matrix receptor interaction, and leukocyte transendothelial migration (Supplementary Figure 2A). Cupcluster C was markedly enriched in metabolic activation pathways, including citrate cycle, TCA cycle, cuproptosis, and nucleotide sugar metabolism (Supplementary Figure 2B). Cupcluster B was enriched in the immunometabolism pathway. Subsequent analyses revealed that the CupCluster C regulatory pattern was significantly associated with stromal activation, including angiogenesis, EMT, and transforming growth factor beta, which was consistent with the shorter survival in CupCluster C (Figure 3D). The specific correlation between each TME infiltrating cell type and each cuproptosis regulator was examined using Spearman’s correlation analysis (Supplementary Figure 3B). We focused on FDX1, as an important cuproptosis regulator, which was negatively correlated with immune score (Supplementary Figure 3A) and revealed its relationship with TME infiltrating immune cells using the ESTIMATE algorithm (Supplementary Figure 3C). The results showed that FDX1 expression affected the infiltration of a small number of immune cells. Therefore, we hypothesised that FDX1 may be involved in regulating tumour progression through metabolic pathways rather than the surrounding immune microenvironment. Based on the above analysis, we unexpectedly found that the three cuproptosis regulation patterns had significantly different biological characteristics. Cupcluster A was classified as an immunoinflammatory phenotype characterised by increased immune cell infiltration, Cupcluster B was classified as an immunometabolism-deficient phenotype characterised by the absence of immune cell infiltration and metabolic activity, and Cupcluster C was classified as a metabolically active phenotype characterised by high stromal activity.

### Generation of cuproptosis gene signatures and functional annotation

**Figure 4:**
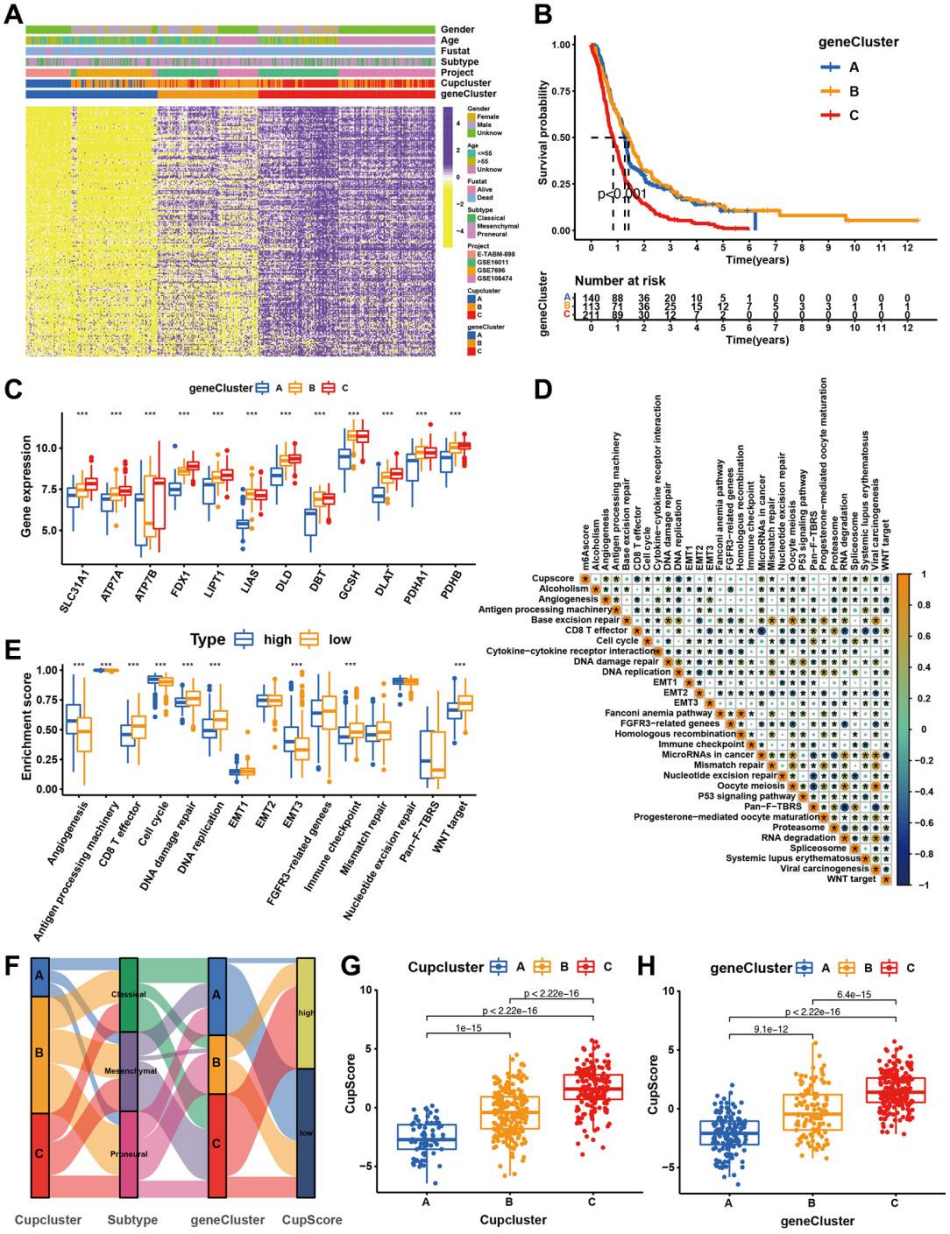
**Construction of cuproptosis signatures.** (**A**) Unsupervised clustering of overlapping cuproptosis regulation patterns-related DEGs to classify patients into different genomic subtypes (cuproptosis gene clusters A, B, and C). The cupcluster, genecluster, gender, age, molecular subtypes, and survival status were used as patient annotations. (**B**) Kaplan-Meier plotter was used to estimate the survival of patients in the cuproptosis gene clusters. (p < 0.001, Log-rank test). (**C**) Differential expression of cuproptosis regulators in three gene cluster. Significant results are indicated as ^*^p < 0.05, ^**^p < 0.01, and ^***^p < 0.001. (**D**) Correlation analysis of CupScore and other known gene signatures using Spearman analysis. (**E**) Differences in stroma-activated pathways between high- and low-CupScore groups. (**F**) Alluvial diagram showing the changes of cuproptosis clusters, molecular subtypes, gene cluster and CupScore. (**G**) Differences in CupScore among three cuproptosis regulation patterns in merge cohort (*P* < 0.001, Kruskal-Wallis test). (**H**) Differences in CupScore among three gene clusters in merge cohort (*P* < 0.001, Kruskal-Wallis test).

To further investigate the potential biological characteristics of each cuproptosis regulatory phenotype, 272 cuproptosis pattern-related DEGs were identified using the limma package (Supplementary Figure 4A). GO and KEGG enrichment analyses of DEGs revealed that these significantly enriched biological processes were associated with cuproptosis metabolism and immunity, which again demonstrated that the cuproptosis regulation phenotype plays a non-negligible role in the TME (Supplementary Figure 4C, 4D). The DEGs were prognostically filtered to 205 genes using univariate Cox regression analysis (Supplementary Table 6). To verify this regulatory mechanism, unsupervised clustering analysis was used to classify patients into distinct genomic subtypes based on the acquired 205 cuproptosis phenotype associated DEGs. The analysis revealed three distinct cuproptosis-regulated genomic phenotypes (cuproptosis gene clusters A-C) (Supplementary Figure 4B). We observed that patients with GBM in the cuproptosis gene cluster C exhibited more mesenchymal subtypes and death status than those in the cuproptosis gene clusters A and B. The opposite pattern was observed in cuproptosis gene clusters A and B. Patients with survival status or proneural subtype were mainly clustered in cuproptosis gene cluster A (Figure 4A). Survival analysis also showed that among the patients in the cuproptosis gene clusters A, B, and C, those in the cuproptosis gene cluster C exhibited the worst survival (Figure 4B). Most of the cuproptosis regulators were highly expressed in cuproptosis gene cluster C (Figure 4C). To further explore the function of the distinct cuproptosis gene clusters A-C, we examined other known signatures in patients with GBM (Supplementary Table 7). The results also showed that gene cluster A was significantly associated with immune activation states, and gene cluster C was characterised by stromal activation and cancer promotion (Supplementary Figure 4E). The above analyses have shown important roles for cuproptosis regulators in shaping the surrounding landscape of GBM. However, these analyses were based on patient populations and did not accurately assess the mode of cuproptosis regulation in individual patients. To further explore the heterogeneity and complexity of cuproptosis regulators in individual tumours, we constructed a scoring system, CupScore, for cuproptosis regulatory patterns in individual GBM patients. A total of 469 patients with GBM were randomly allocated to the training and validation cohorts. A total of 205 cuproptosis phenotype-related DEGs were subjected to LASSO regression followed by PCA (Supplementary Figure 5A, 5B and Supplementary Table 8). With a cutoff value of 0.3054, patients were divided into low- or high-CupScore groups in the training and validation sets. Patients with a low CupScore showed a prominent survival benefit (Supplementary Figure 5C, 5D). The CupScore was confirmed to be a robust and independent prognostic biomarker for GBM in univariate and multivariate Cox regression analyses (Supplementary Figure 5E, 5F). The ROC curve further demonstrated the ability of the CupScore signature to predict patient outcomes (Supplementary Figure 5G, 5H). Dramatic changes in the attributes of individual GBM patients were observed in the alluvial diagram (Figure 4F). We explored the association between CupScore and known signatures using both correlation analyses (Figure 4D). Analysis of relevant pathway activities showed that low CupScore in patients with GBM was associated with Wnt and CD8 T pathways, whereas high CupScore was related to enhanced activation of angiogenesis and EMT pathways (Figure 4E). The Kruskal–Wallis test revealed that CupScore was the lowest in Cupcluster A and highest in Cupcluster C (Figure 4G). Similarly, CupScore was lowest in gene cluster A and highest in cluster C (Figure 4H). These results strongly suggest that a low CupScore could be associated with immune activation, whereas a high CupScore could be associated with stromal activation. Patients with a high CupScore showed a poor survival benefit. Therefore, CupScore provides a better assessment of cuproptosis regulation patterns for individual tumours.

### Characteristics of cuproptosis metabolism in clinical information and tumour somatic mutation

**Figure 5:**
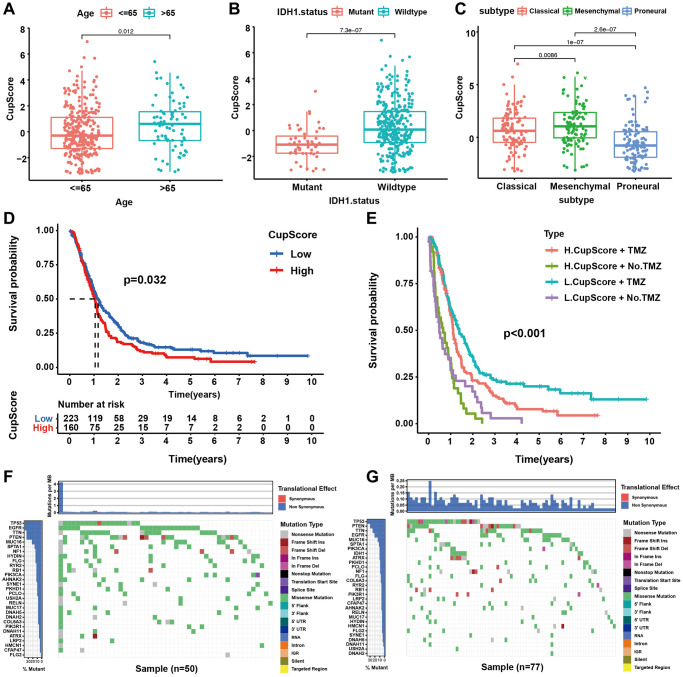
**Characteristics of cuproptosis metabolism in molecular subtypes and tumour somatic mutation.** (**A**) Differences in CupScore between different age status. (*P* = 0.012, Kruskal-Wallis test). (**B**) Differences in CupScore between different IHD1 status. (*P* < 0.001, Kruskal-Wallis test). (**C**) Differences in CupScore between different GBM molecular subtypes. (*P* < 0.001, Kruskal-Wallis test). (**D**) Survival analyses for high (*n* = 160) and low (*n* = 223) CupScore GBM groups in merge cohort (TCGA and CGGA, *P* = 0.032, Log-rank test). (**E**) Survival analyses for subgroup patients stratified by both CupScore and treatment with pharmacological chemotherapy (TMZ, temozolomide) using Kaplan–Meier curves. (*P* < 0.001, Kruskal-Wallis test). (**F**, **G**) The waterfall plot of tumour somatic mutation established by those with high- (**F**) and low CupScore (**G**).

We further explored the relationship between cuproptosis regulation modalities and clinical traits, mutant phenotypes, and molecular subtypes through correlation analysis of the CupScore system in TCGA and CGGA cohorts. We found that older patients (Figure 5A), IDH wild-type patients (Figure 5B), and mesenchymal subtype (Figure 5C) patients were significantly associated with a higher CupScore, implying that these patients may be ascribed a Cupcluster-C modification pattern and stromal activation phenotype with worse clinical outcomes. Using the same cutoff value, patients were divided into low or high CupScore groups, with 223 and 160 patients, respectively. Kaplan-Meier curves indicated that low CupScore was markedly related to the overall survival of 383 patients in the merged cohort (Figure 5D). We examined the ability of the CupScore signature to predict the efficacy of adjuvant chemotherapy (TMZ) in GBM patients. We found that patients with a low CupScore showed a significant treatment advantage among those who received TMZ (Figure 5E). Interestingly, we were surprised to find that the predictive ability of the CupScore was not disturbed by adjuvant chemotherapy, with or without TMZ, and the low CupScore group exhibited a significant survival advantage (Figure 5E). The maftools package was used to show the distribution differences of somatic mutations between low and high CupScores in TCGA-GBM. We found that the high CupScore group presented a slightly higher tumour mutation burden than the low CupScore group, with the rate of the 5^th^ most significantly mutated gene being 17% versus 12% (Figure 5F, 5G). The above results indicated that the CupScore could also be used to evaluate certain clinical characteristics of patients, such as older age, wild-type IDH, and molecular subtypes.

### Novel cuproptosis-related genes and their prognostic value

**Figure 6:**
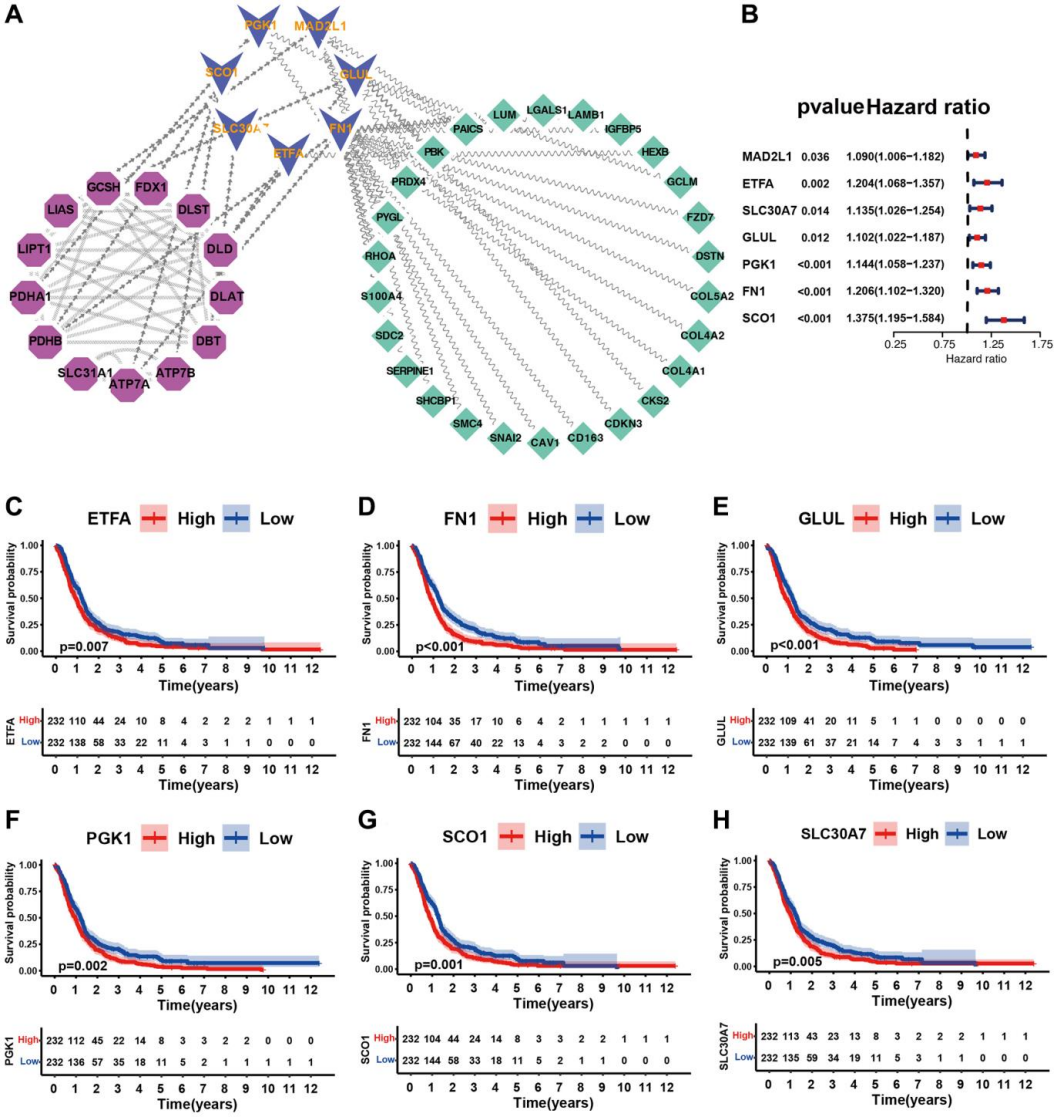
**Identification of cuproptosis-related novel genes and their potential characteristics.** (**A**) Identification of cuproptosis-related novel genes using PPI network. Purple represented known cuproptosis regulators, blue represented novel cuproptosis regulators, and green represented indirect cuproptosis regulators. (**B**) Forest plot of novel cuproptosis regulators using univariate Cox regression analysis in merge cohort (GSE7696, GSE16011, GSE108474, ArrayExpress-E-TABM-898). (**C**–**H**) Kaplan-Meier survival curves for patients of GBM with high and low gene expression in merge dataset (GSE7696, GSE16011, GSE108474, ArrayExpress-E-TABM-898).

To further explore the regulatory mechanism of cuproptosis, we identified several new cuproptosis regulators using PPI network analysis based on 13 cuproptosis regulators and 205 cuproptosis regulation pattern related DEGs. Among these genes, seven were direct cuproptosis regulators and 26 were indirect regulators (Figure 6A), which provides strong foundations for further research on the occurrence and development of cuproptosis. Prognostic analysis suggested that all seven direct regulators of cuproptosis were risk factors (Figure 6B). Survival analysis showed that GBM patients with high expression (ETFA, FN1, GLUL, PGK1, SCO1, and SLC30A7) possessed poorer prognosis (Figure 6C–6H). These results strongly suggest that the newly identified cuproptosis regulators may contribute to tumour-promoting characteristics.

### SLC30A7 overexpression predicted poor survival and SLC30A7 knockdown inhibited cell proliferation, migration, invasion and reverse EMT *in vitro*

**Figure 7:**
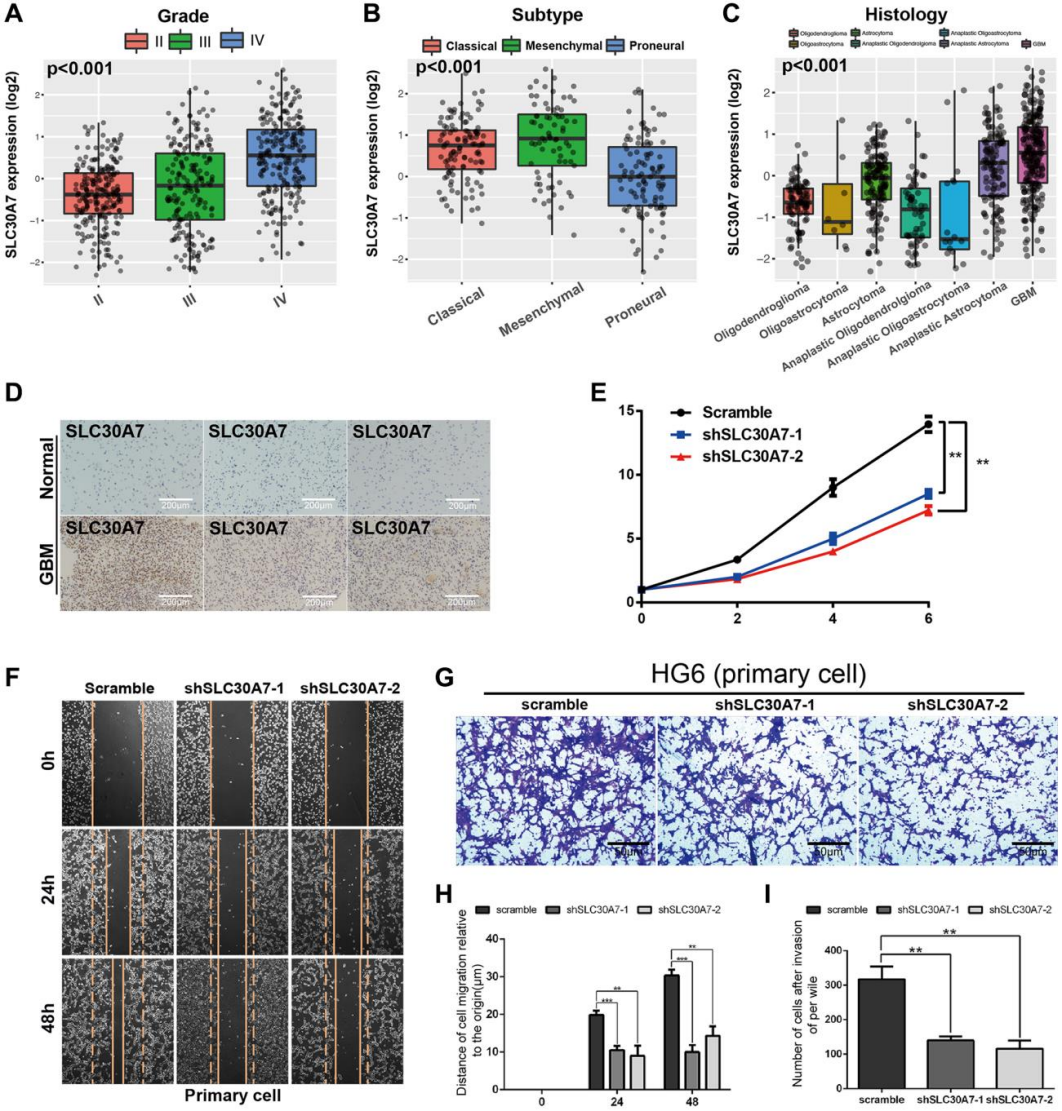
**The expression of SLC30A7 and its effect on proliferation, invasion, migration and epithelial mesenchymal transformation of primary GBM cells.** (**A**–**C**) Boxplots showing the SLC30A7 distributions according to grade, molecular subtype, and histology in CGGA cohort (*P* < 0.001). (**D**) Expression of SLC30A7 in normal tissues and GBM. (**E**) Proliferation of cells stably transfected with SLC30A7 knockdown lentivirus in different group. Significant results are indicated as ^**^p < 0.01. (**F**–**I**) Cell scratch and Trans-well assays detected the invasion and migration of GBM primary cells after inhibition of SLC30A7, H is the statistic of the migration distance in scratch assay at different time points, I is the counting of the invasion penetrated out chamber cells after Transwell assay. Significant results are indicated as ^**^p < 0.01, and ^***^p < 0.001.

Disruption of zinc homeostasis has been found to be causally associated with tumorigenesis in various cancer patients [28]. As a Golgi located zinc transporter, SLC30A7 exhibits anti oxidative stress effect and induced apoptosis via the NFE2L2/HMOX1 pathway under high glucose (HG) conditions (32949653). SLC30A7 may be involved in tumour initiation and progression [29]. But there are few reports about SLC30A7 in gliomas. To explore the prognostic value of SLC30A7 in gliomas, public databases were used to analyzed SLC30A7 expression. SLC30A7 was increased in higher WHO grade, mesenchymal subtype and GBM histology (Figure 7A–7C). GBM patients with high SLC30A7 expression possessed a shorter survival (Figure 6H). Immunohistochemistry (IHC) analysis revealed that SLC30A7 was upregulated in GBM tissues than normal tissues (Figure 7D). To investigate the oncogenic role of SLC30A7 in glioma cells, SLC30A7-silenced cell models was constructed by transfecting siRNAs into DT001 cells (Figure 7H, 7I). CCK-8 and wound-healing and Transwell assay verified that silencing SLC30A7 significantly attenuated the proliferation, migration, and invasion of glioma cells (Figure 7E–7G). These data suggest that SLC30A7 may regulate the EMT process to facilitates malignant behavior in GBM cells.

### SLC30A7 knockdown inhibited the tumorigenicity of GBM cell *in vivo*

**Figure 8:**
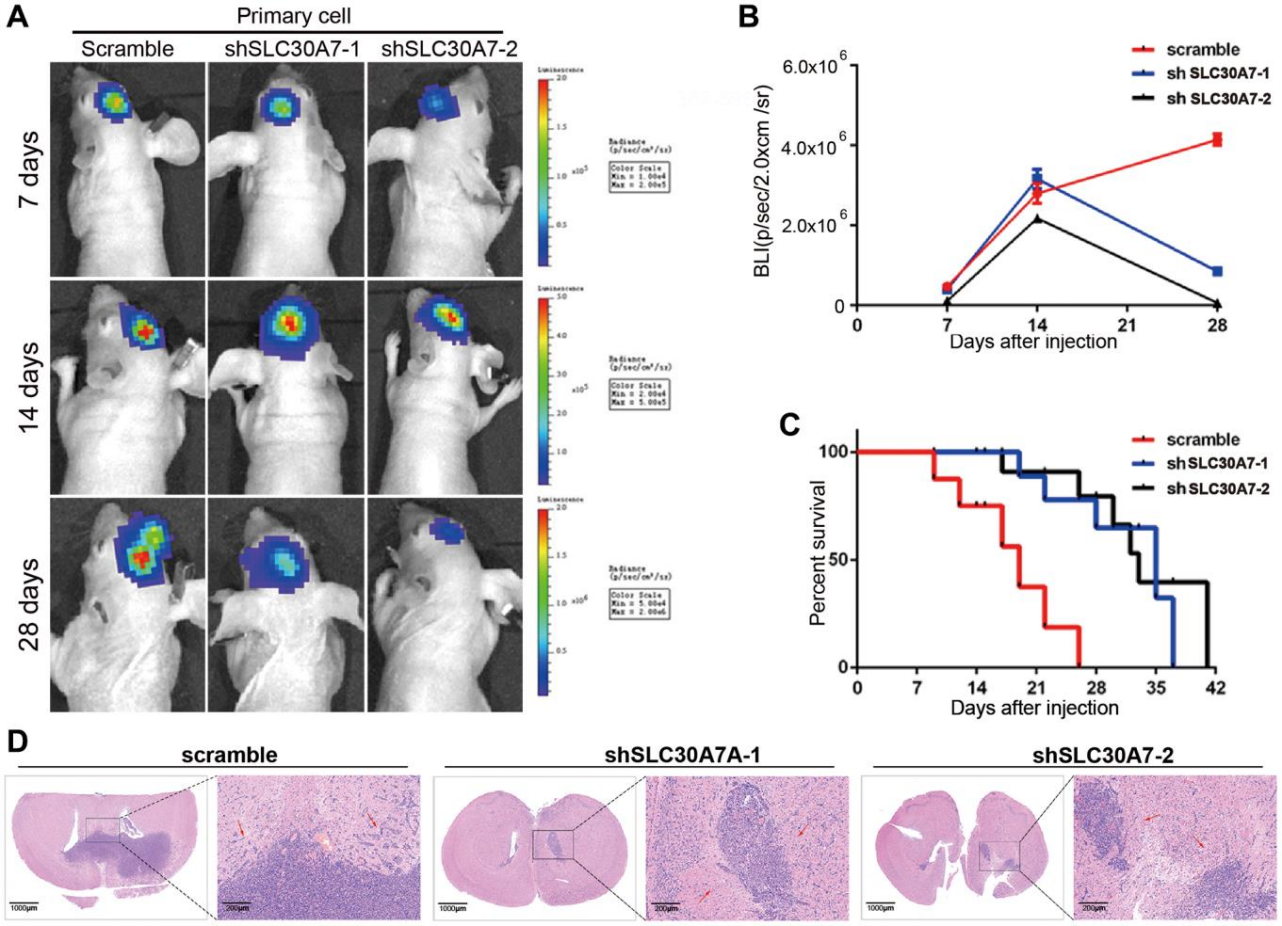
**Detection of tumorigenesis of SLC30A7 stably knocked down in nude mouse orthotopic implanted tumour model.** (**A**) Representative images of bioluminescence of mice on days 7, 14, and 28 after implantation. (**B**) Quantitative analysis of these bioluminescence images for the Scramble, shSLC30A7-1 and shSLC30A7-2 treatment groups. Data are shown as the mean ± S.D. *n* = 6, ^****^P < 0.0001 compared to the control, Student’s *t*-test. (**C**) The overall survival of mice in different groups. Data are shown as the mean ± S.D. *n* = 6, ^NS^*P* > 0.05, ^**^P < 0.01 compared to the control, ANOVA test. (**D**) Representative images of the HE (×4 magnification, scale bar = 1000 μm) and HE staining in local area enlargement of tumour (×40 magnification, scale bar = 200 μm). The three rows of HE samples are repeated data from different processing groups. The outlined sections of left images were defined as higher magnification sections right.

To confirm the effects of SLC30A7 in GBM tumorigenesis *in vivo*. Intracranial orthotopic xenotransplantation models were constructed to verify that silencing SLC30A7 significantly decreased transplant tumour size (Figure 8A, 8B, 8D). KM survival analysis demonstrated that the survival time of xenograft mice was noticeably prolonged after silencing SLC30A7 to control mice (Figure 8C). These data further confirm that SLC30A7 plays a role in promoting the malignant progression of GBM *in vivo*.

### SLC30A7 suppressed cuproptosis via activating the JAK2/STAT3/ATP7A pathway

**Figure 9:**
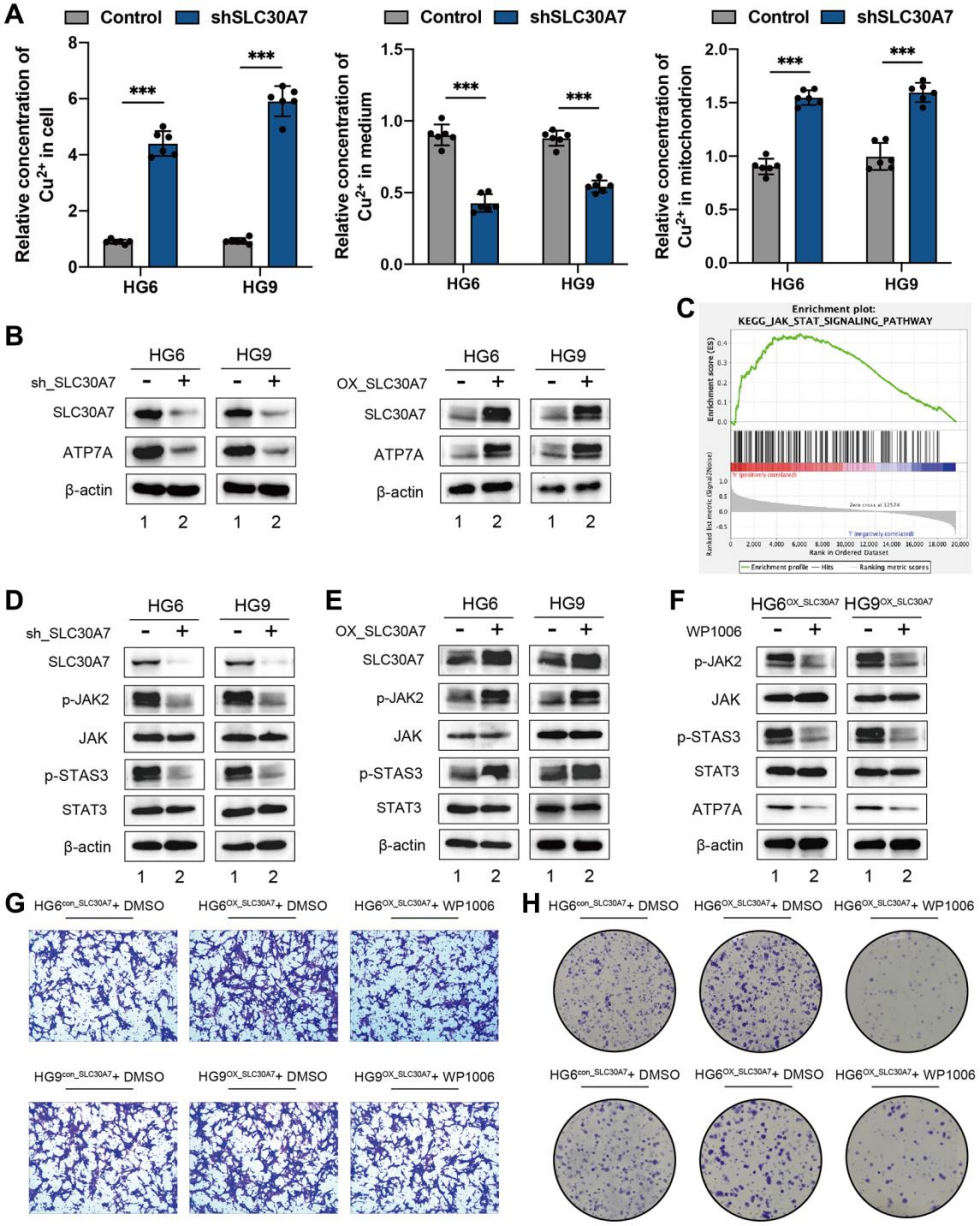
**SLC30A7 suppressed cuproptosis via activating the JAK2/STAT3/ATP7A pathway.** (**A**) Cu^2+^ levels in cytoplasm, mitochondria and medium were measured using Copper (Cu) Colorimetric Assay Kit (Elabscience, E-BC-K300-M). (**B**) Western blot showing the protein level of ATP7A following SLC30A7 knockdown in GBM cells. (**C**) GSEA analysis showed that the high expression SLC30A7 group was positively correlated with the JAK-STAT pathway. (**D**, **E**) Western blot analysis showed that SLC30A7 activated the JAK2/STAT3 pathway. (**F**) Western blot analysis demonstrated that JAK2/STAT3 pathway promoted APT7A protein levels in GBM cells. Conversely, inhibitor of JAK2/STAT3 signaling partially rescued APT7A protein levels in GBM cells. (**G**) Colony formation assay of the effect of WP1066 on the growth of GBM cells after overexpression of SLC30A7. (**H**) Transwell migration analysis showing the effect of WP1066 on GBM cells after overexpression of SLC30A7 on GBM cells.

To investigate the function of SLC30A7 as a novel cuproptosis regulator in GBM, the concentration of copper in the cell fraction and medium was analyzed. Knockdown of SLC30A7 led to an increase of copper in cytoplasm and mitochondria, but a decrease in medium (Figure 9A), suggesting that SLC30A7 affected copper homeostasis in GBM cells. Knockdown of SLC30A7 decreased ATP7A levels and SLC30A7 overexpression increased ATP7A levels (Figure 9B). The GSEA analysis showed that SLC30A7 was significantly enriched in the JAK-STAT signaling pathway (Figure 9C). Importantly, Knockdown of SLC30A7 decreased phosphorylated (p)-JAK2 and p-STAT3 levels (Figure 9D), and SLC30A7 overexpression increased p-JAK2 and p-STAT3 levels (Figure 9E). JAK2 inhibitor WP1006 reversed SLC30A7-induced APT7A in HG6 and HG9 cells (Figure 9F). In addition, Inhibition of JAK2/STAT3 signaling prevented SLC30A7-induced proliferation and migration in GBM cells overexpressing SLC30A7 (Figure 9G, 9H). These data demonstrate that SLC30A7 suppressed cuproptosis through the JAK2/STAT3/ATP7A pathway.

## DISCUSSION

The emergence of copper-dependent controlled cell death modalities has revealed that the interplay between individual cuproptosis regulators plays an indispensable role in tumour metabolism, the tumour microenvironment, and antitumour effects [30, 31]. However, swarming characteristics mediated by the combined actions of multiple cuproptosis regulators have not been explored. Thus, identifying the roles of distinct modes of cuproptosis regulation in the tumour microenvironment can help enhance our understanding of tumorigenesis and guide more effective therapeutic strategies.

Based on 13 cuproptosis regulators, we found three distinct modes of cuproptosis regulation. Cluster A was characterised by increased immune cell infiltration, corresponding to an immunoinflammatory phenotype; Cluster B was characterised by absence of immune and metabolic activity, corresponding to an immunometabolic deficient phenotype; Cluster C was characterised by high metabolic activity, corresponding to a metabolic activation phenotype [32]. The immunometabolic null phenotype is considered indolent. Tumours exhibiting immunoinflammatory and metabolic activation phenotypes are considered hot tumours [33, 34]. Altered cellular metabolism has been reported to be a hallmark of cancer [35]. Alterations in stromal cell metabolism have also been reported with tumour destruction [36]. Catabolites are transferred from supporting stromal cells to adjacent cancer cells as a result of cellular metabolic interactions within the tumour [37]. Thus, metabolic crosstalk between the tumour and stroma is crucial for the progressive malignant journey of tumour cells. Consistent with the appeal descriptions, we found that cluster C was a metabolically active phenotype exhibiting a marked stromal activation state, including high expression of angiogenic and EMT pathways. These results confirm the reliability of typing cuproptosis regulation patterns.

In this study, the DEGs identified in the different cuproptosis regulation patterns were significantly associated with immune and metabolic pathways. These DEGs are considered cuproptosis-related signature genes. Similar to the clustering results for the regulation patterns of cuproptosis, three genomic subtypes were identified based on cuproptosis signature genes, which were also associated with metabolic and immune pathways. This again illustrates that cuproptosis regulators play an important role in shaping the TME. Therefore, a comprehensive assessment of cuproptosis regulatory modes may enhance our understanding of copper ion-induced alterations in the TME. Considering the heterogeneity of individual tumour cuproptosis regulation patterns, we established a scoring system, a cuproptosis gene signature, to evaluate the cuproptosis regulation pattern in individual patients with GBM. The immunoinflammatory phenotype had a lower CupScore, whereas the metabolic activation phenotype had a higher CupScore. In addition, it was well validated that the high CupScore was significantly associated with advanced age, IDH wild-type, and mesenchymal subtypes in both TCGA and CGGA cohorts, and patients with low CupScore were more sensitive to TMZ adjuvant chemotherapy.

Our data also revealed a markedly negative correlation between the CupScore and tumour mutation burden. Integrated analysis demonstrated that the CupScore is an independent prognostic marker for GBM. This suggests that the CupScore is a reliable and robust tool for comprehensively assessing cuproptosis regulation patterns for individual tumours.

We identified seven novel regulators of cuproptosis that are risk factors for GBM. Importantly, we found that SLC30A7 overexpression predicted poor survival and SLC30A7 knockdown inhibited cell proliferation, migration, and invasion *in vitro*. Meanwhile, SLC30A7 knockdown inhibited the tumorigenicity of GBM cell *in vivo*. Mechanistically, SLC30A7 suppressed cuproptosis through the JAK2/STAT3/ATP7A pathway.

In short, in clinical practice, the CupScore provides a comprehensive assessment of cuproptosis regulation patterns and microenvironmental features of individual tumours. Patient prognosis can be predicted further by determining the tumour phenotype. We demonstrated that CupScore can be used to evaluate the clinical features of patients, including age, IDH status, and molecular types. The CupScore is effective for predicting patient survival. In addition, the CupScore can effectively predict patient response to TMZ treatment. Importantly, this study provides a new approach for the treatment of GBM, targeting cuproptosis regulators or cuproptosis-associated DEGs to reverse the malignant tumour phenotype and contribute to future new drug discovery. Interestingly, this is the first study to report SLC30A7 in GBM. SLC30A7 knockdown evidently inhibited GBM cell proliferation through the JAK2/STAT3/ATP7A pathway *in vitro* and *in vivo*.

## CONCLUSION

In conclusion, three distinct cuproptosis regulation patterns (immune activation, metabolic activation, and immunometabolic double deletion patterns) were identified by Cuproptosis regulators. Three regulated genomic phenotypes (cuproptosis gene clusters A, B and C) were determined based on clustering analysis of three distinct phenotypes related DEGs. Constructing CupScores were used to evaluate cuproptosis regulation patterns of individual samples. The results of the PPI network analysis illustrated novel cuproptosis-related genes. Furthermore, we revealed that SLC30A7 knockdown inhibited the tumorigenicity of GBM cell through the JAK2/STAT3/ATP7A pathway *in vitro* and *in vivo*.

## Supplementary Materials

Supplementary Figures

Supplementary Tables 1-3 and 5

Supplementary Tables 4 and 6-8
