# Supplementary material for: Investigation of cuproptosis regulator-mediated modification patterns and SLC30A7 function in GBM
Source: Aging (Albany NY). 2024 Feb 22;16(4):3554–82. doi: 10.18632/aging.205545 (PMC10929835; doi:10.18632/aging.205545)
Supplement: Supplementary Figures [file aging-16-205545-s001.pdf]

SUPPLEMENTARY FIGURES

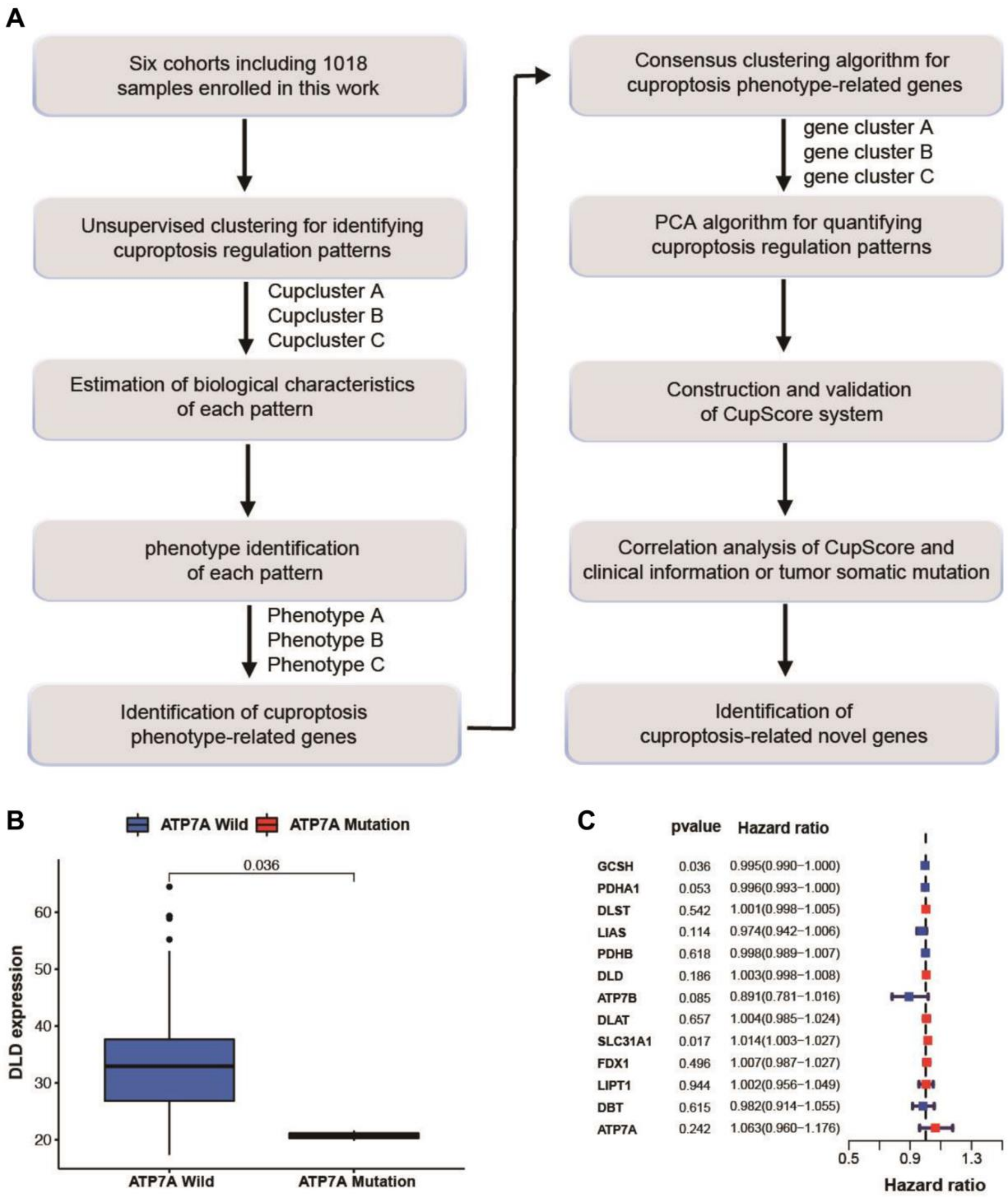

Supplementary Figure 1. Overview of study design and prognostic analysis of 13 cuproptosis regulators. (**A**) Overview of this work. (**B**) Expression differences of DLD between ATP7A wild and ATP7A mutation in TCGA and CGGA cohorts. (**C**) The prognostic analyses for 13 cuproptosis regulators in TCGA and CGGA cohorts using a univariate Cox regression model.

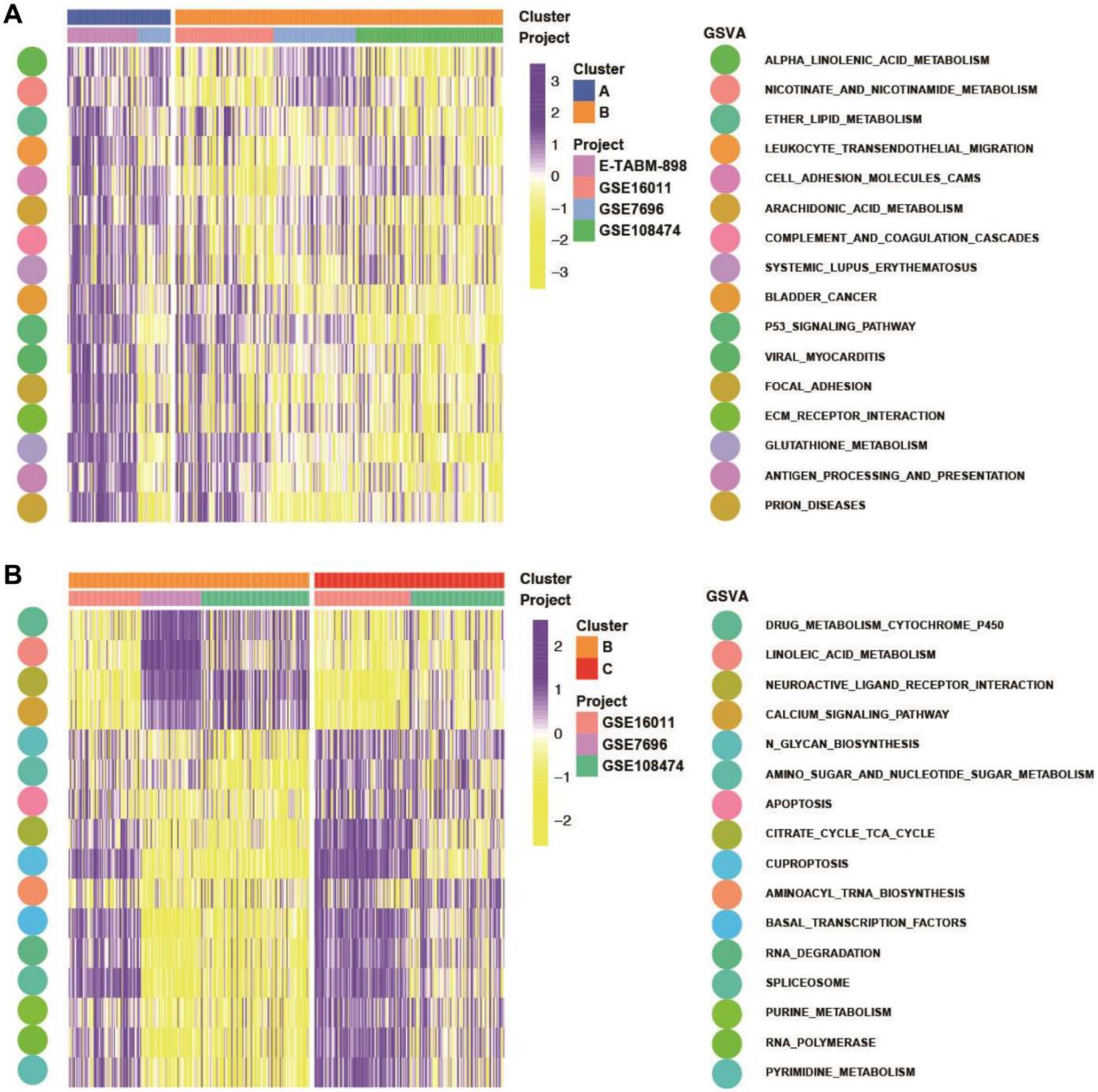

Supplementary Figure 2. Biological characteristics of cuproptosis regulation pattern. (**A**) GSVA enrichment analysis showing the activation biological pathways between cuproptosis regulation patterns A and B. Purple: activated pathways; yellow: inhibited pathways. The GBM cohorts were used as sample annotations (GSE7696, GSE16011, GSE108474, ArrayExpress-E-TABM-898). (**B**) GSVA enrichment analysis showing the activation biological pathways between cuproptosis regulation patterns B and C. Purple: activated pathways; yellow: inhibited pathways. The GBM cohorts were used as sample annotations (GSE7696, GSE16011, GSE108474, ArrayExpress-E-TABM-898).

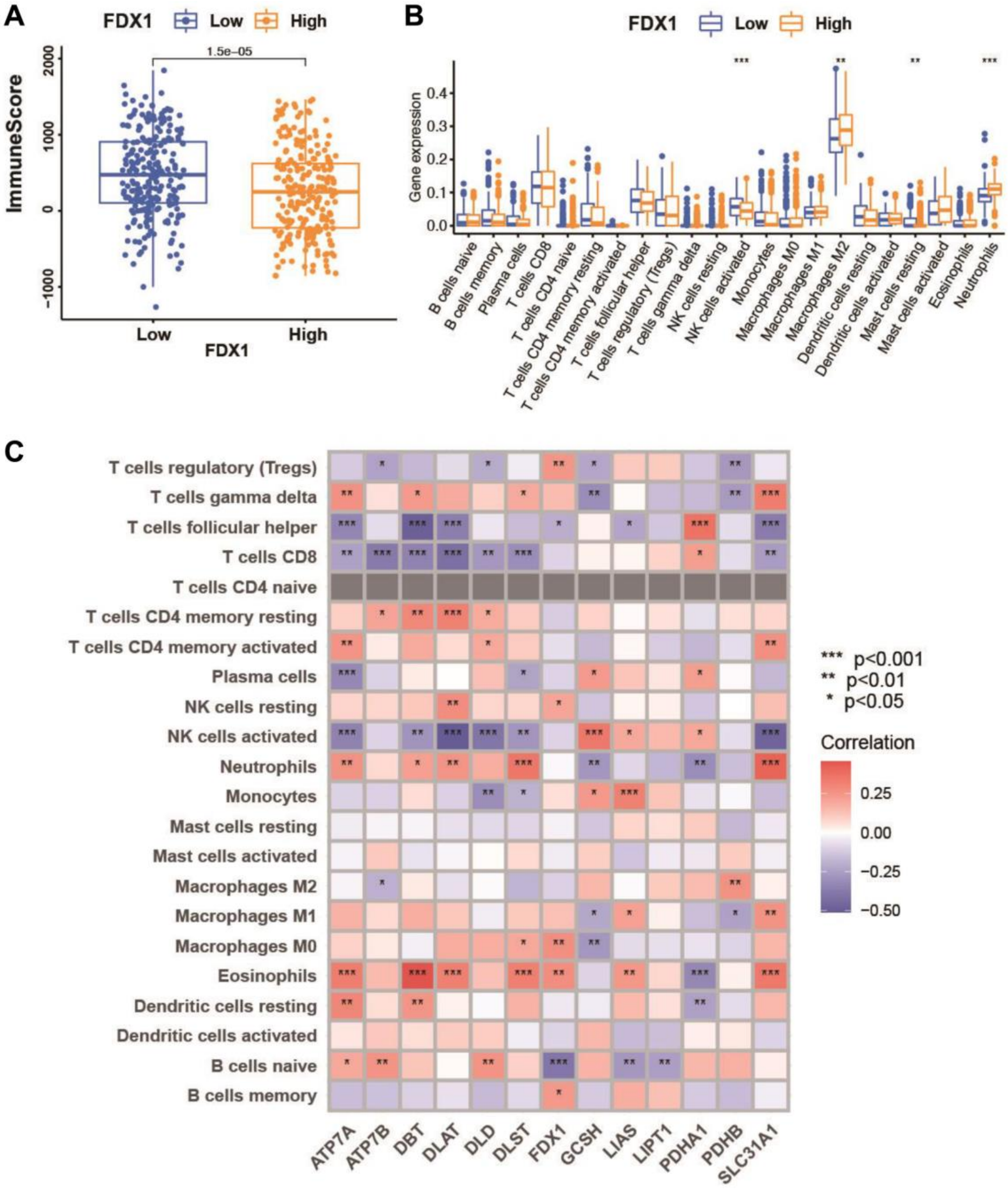

Supplementary Figure 3. Correlation analysis between TME infiltrating cells and cuproptosis regulators. (**A**) Expression differences of ImmuneScore in high- and low-FDX1 expression and mutation in four GBM cohorts. (**B**) Difference in the abundance of each TME infiltrating cell between FDX1 high expression and low expression groups. (**C**) The correlation between each cuproptosis regulator and each TME infiltration cell type. Red: positive correlation; Purple: negative correlation.

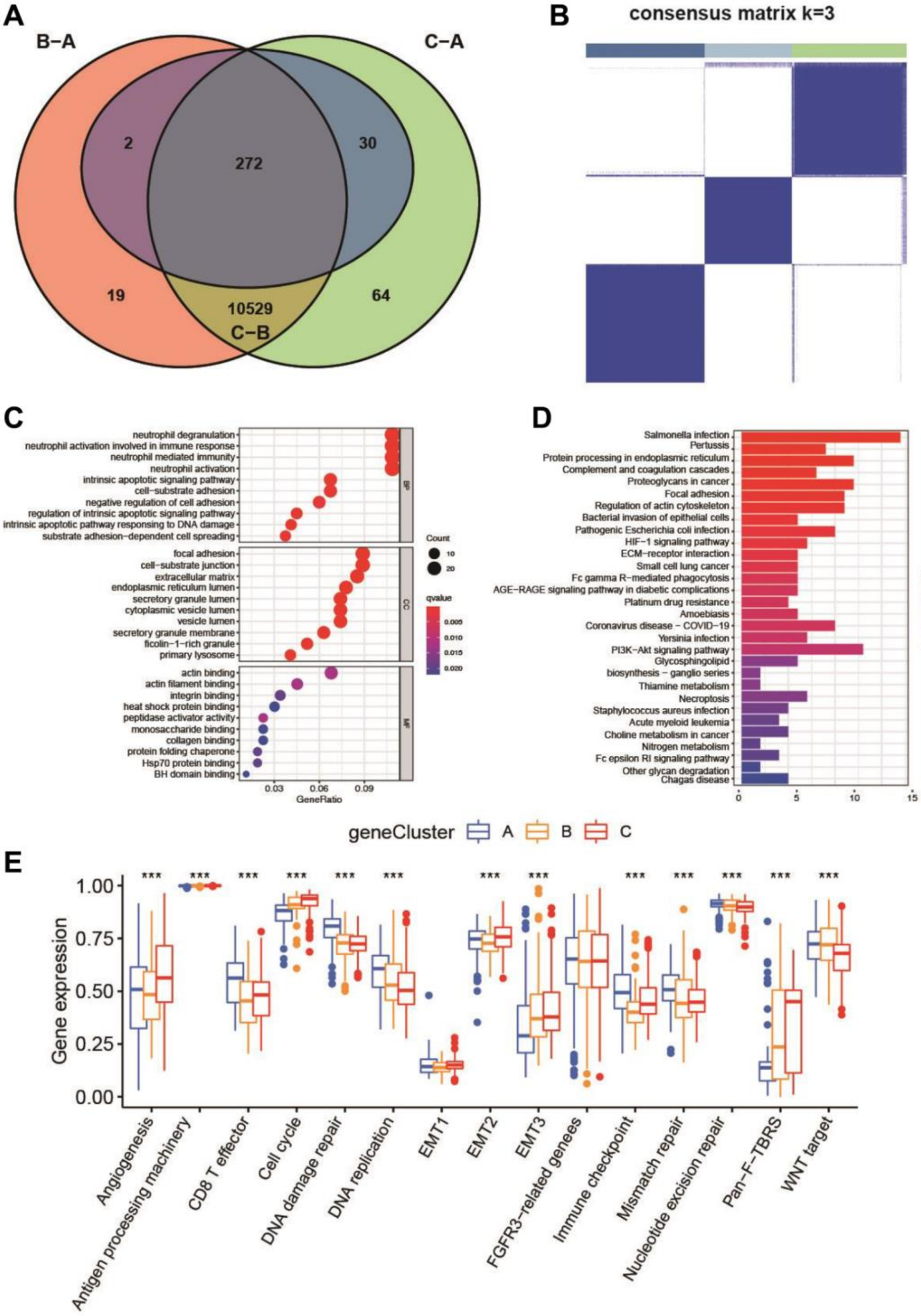

Supplementary Figure 4. Identification and functional annotation of cuproptosis pattern related DEGs. (**A**) Venn diagram indicating cuproptosis-related genes identified in three patterns. (**B**) Unsupervised clustering of 205 cuproptosis regulation pattern related DEGs and consensus matrices for k = 3, which was the optimal cluster number in four GBM cohorts. (**C**, **D**) Functional annotation for cuproptosis-related genes using GO and KEGG enrichment analysis. The color depth of the barplots represented the number of genes enriched. (**E**) Difference in the expression of known signatures including stromal-activation related signatures among three gene clusters. significant results are indicated as $^{*}p < 0.05$, $^{**}p < 0.01$, and $^{***}p < 0.001$.

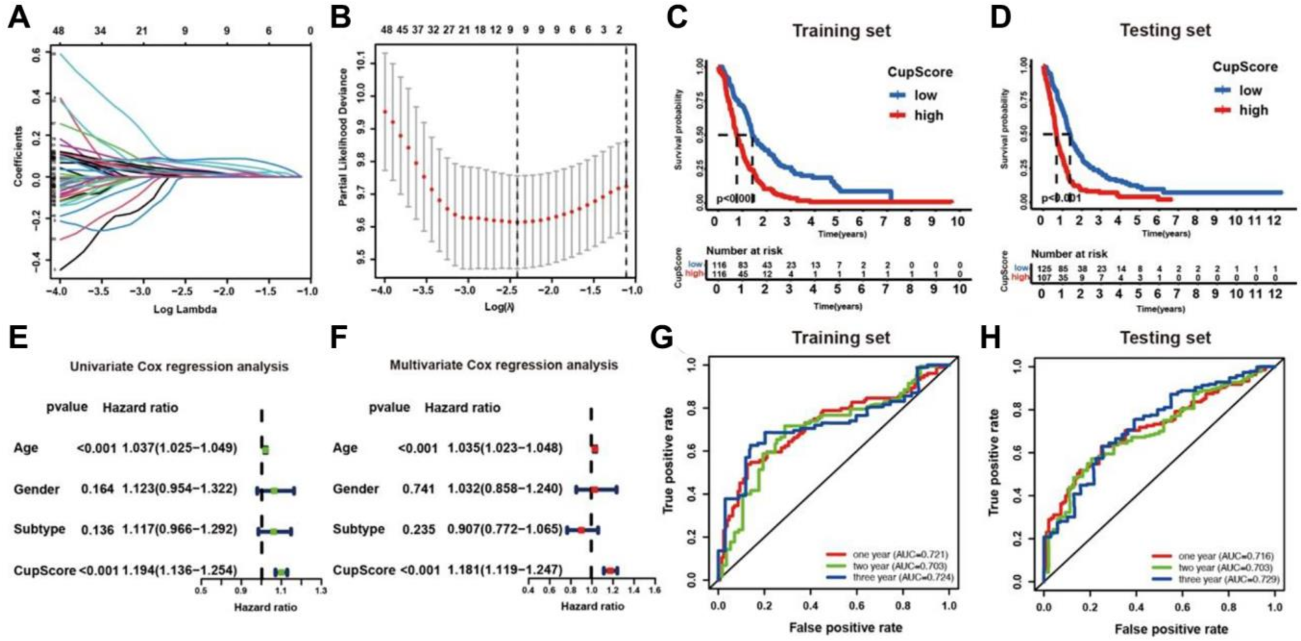

Supplementary Figure 5. Construction and validation of cuproptosis gene signature. (**A**) LASSO coefficient profiles of the cuproptosis regulation pattern related DEGs in the training set. (**B**) A coefficient profile plot was generated against the log (lambda) sequence. Selection of the optimal parameter (lambda) in the LASSO model. (**C**) Survival analyses for low (116 cases) and high (116 cases) CupScore patient groups in training cohort using Kaplan-Meier curves. (**D**) Survival analyses for low (125 cases) and high (107 cases) CupScore patient groups in testing cohort using Kaplan-Meier curves. (**E**, **F**) Forest plot showing univariate and multivariate Cox regression analyses of CupScore associated with age, gender, subtype and CupScore of two cohorts. (**G**, **H**) The predictive value of the quantification of cuproptosis gene signatures in training cohort (AUC, 0.721) and testing cohort (AUC, 0.716).
