# Supplementary material for: Investigation of cuproptosis regulator-mediated modification patterns and SLC30A7 function in GBM
Source: Aging (Albany NY). 2024 Feb 22;16(4):3554–82. doi: 10.18632/aging.205545 (PMC10929835; doi:10.18632/aging.205545)
Supplement: Supplementary Tables 1-3 and 5 [file aging-16-205545-s002.pdf]

SUPPLEMENTARY TABLES

Supplementary Table 1. Basic information of datasets included in this study for identifying distinct cuproptosis modification.

patterns

Accession number/Source	Platform	Number of patients	Sex	Histology	Survival data
GEO: GSE7696	Affymetrix Human Genome U133 Plus 2.0 Array	80	Female:21<br>Male:59	GBM	OS
GEO: GSE16011	Affymetrix Human Genome U133 Plus 2.0 Array	155	Female:50<br>Male:105	GBM	OS
GEO: GSE108474	Affymetrix Human Genome U133 Plus 2.0 Array	219	NA	GBM	OS
ArrayExpress: E-TABM-898	Affymetrix Human Genome U133 Plus 2.0 Array	48	NA	GBM	OS/RFS
CGGA-GBM	Illumina HumanHT-12 V3.0 expression beadchip	365	Female:146<br>Male:219	GBM	OS
TCGA-GBM	Illumina RNAseq	115	Female:45<br>Male:70	GBM	OS

Supplementary Table 2. survival analysis of the 13 cuproptosis regulators.

Id	HR	HR.95L	HR.95H	*p*-value	km
FDX1	1.006973402	0.987041029	1.02730829	0.495712101	0.047968317
LIPT1	1.001648786	0.956404591	1.049033328	0.944307596	0.164799545
LIAS	0.973771457	0.942201084	1.00639966	0.113970167	0.041093939
DLD	1.003393364	0.998367707	1.008444319	0.186067075	0.000934189
DBT	0.981688519	0.913508262	1.054957452	0.614810538	0.321149256
GCSH	0.994710163	0.989782823	0.999662032	0.036315368	0.001451297
DLST	1.001105558	0.997555792	1.004667955	0.542076987	0.001019463
DLAT	1.004374207	0.985211189	1.023909959	0.656989661	0.1576814
PDHA1	0.996353581	0.992678727	1.000042039	0.052663976	0.021418894
PDHB	0.997764372	0.989033005	1.006572822	0.617721571	0.022048795
SLC31A1	1.014496499	1.002571364	1.026563478	0.017049571	0.000886202
ATP7A	1.062507059	0.959832307	1.176165088	0.242276723	0.006947257
ATP7B	0.890913734	0.781113912	1.016147925	0.085204493	0.006653688

Supplementary Table 3. Spearman correlation analysis of the 13 cuproptosis regulators.

From	To	cor	*p*-value	Weight
FDX1	SLC31A1	0.5424231	2.55E-43	3.2545385
FDX1	ATP7A	0.1926444	5.46E-06	1.1558662
FDX1	ATP7B	−0.17246281	4.86E-05	1.0347769
FDX1	LIPT1	0.3857323	6.41E-21	2.3143938
FDX1	LIAS	0.478961	7.89E-33	2.873766
FDX1	DBT	0.4830635	1.92E-33	2.8983811
FDX1	GCSH	−0.20627506	1.09E-06	1.2376504
FDX1	DLAT	0.594214	1.05E-53	3.565284
FDX1	PDHA1	−0.44662004	2.84E-28	2.6797203
SLC31A1	ATP7A	0.5431786	1.86E-43	3.2590717
SLC31A1	LIAS	0.1985246	2.76E-06	1.1911478
SLC31A1	DLD	0.2576007	9.03E-10	1.5456041
SLC31A1	DBT	0.670527	6.02E-73	4.023162
SLC31A1	GCSH	−0.4165432	1.88E-24	2.4992592
SLC31A1	DLST	0.2832381	1.38E-11	1.6994285
SLC31A1	DLAT	0.709952	2.44E-85	4.2597123
SLC31A1	PDHA1	−0.52089866	1.64E-39	3.125392
ATP7A	ATP7B	0.19685	3.36E-06	1.1810998
ATP7A	DBT	0.4609091	3.16E-30	2.7654546
ATP7A	GCSH	−0.36054854	2.69E-18	2.1632912
ATP7A	DLST	0.4572562	1.02E-29	2.7435369
ATP7A	DLAT	0.3955124	5.32E-22	2.3730745
ATP7A	PDHA1	−0.34186315	1.70E-16	2.0511789
ATP7A	PDHB	−0.2156689	3.36E-07	1.2940134
ATP7B	LIPT1	−0.31381789	5.18E-14	1.8829074
ATP7B	LIAS	−0.21137111	5.80E-07	1.2682267
ATP7B	DBT	0.2095438	7.28E-07	1.2572626
ATP7B	GCSH	−0.20255898	1.71E-06	1.2153539
ATP7B	DLST	0.5434111	1.68E-43	3.2604664
LIPT1	LIAS	0.5354436	4.70E-42	3.2126618
LIPT1	DLST	−0.29883206	8.68E-13	1.7929924
LIAS	DBT	0.3483581	4.15E-17	2.0901488
LIAS	DLST	−0.19971439	2.40E-06	1.1982864
LIAS	PDHA1	−0.24828938	3.70E-09	1.4897363
DLD	DBT	0.3195885	1.68E-14	1.9175312
DLD	GCSH	0.2177158	2.58E-07	1.3062949
DLD	DLAT	0.4111505	8.31E-24	2.4669027
DLD	PDHA1	0.1898045	7.54E-06	1.1388272
DLD	PDHB	0.4052214	4.13E-23	2.4313284
DBT	GCSH	−0.19107973	6.53E-06	1.1464784
DBT	DLST	0.2474685	4.18E-09	1.4848112
DBT	DLAT	0.7333104	1.03E-93	4.3998624
DBT	PDHA1	−0.37823222	4.10E-20	2.2693933
GCSH	DLST	−0.41227933	6.10E-24	2.473676
GCSH	DLAT	−0.222563	1.37E-07	1.335378
GCSH	PDHA1	0.4654182	7.32E-31	2.7925091

GCSH	PDHB	0.5535259	2.15E-45	3.3211556
DLST	PDHA1	−0.42071574	5.84E-25	2.5242944
DLST	PDHB	−0.34297013	1.34E-16	2.0578208
DLAT	PDHA1	−0.34249597	1.49E-16	2.0549758
PDHA1	PDHB	0.337695	4.13E-16	2.0261702

Supplementary Table 5. The activation states of biological pathways in distinct cuproptosis regulation patterns by GSV.

Pathway: Cupcluster-A VS Cupcluster-B			
**Id**	**logFC**	**AveExpr**	**t**
KEGG_BLADDER_CANCER	−0.13155	−0.01951	−5.89681
KEGG_FOCAL_ADHESION	−0.15472	−0.02283	−5.87106
KEGG_ARACHIDONIC_ACID_METABOLISM	−0.15541	0.027795	−5.79329
KEGG_LEUKOCYTE_TRANSENDOTHELIAL_MIGRATI	−0.13634	0.005152	−5.75656
KEGG_ECM_RECEPTOR_INTERACTION	−0.23234	−0.0098	−5.69656
KEGG_VIRAL_MYOCARDITIS	−0.12326	−0.0121	−5.31573
KEGG_ANTIGEN_PROCESSING_AND_PRESENTATION	−0.14283	−0.0054	−5.12251
KEGG_CELL_ADHESION_MOLECULES_CAMS	−0.11796	0.042424	−5.09634
KEGG_COMPLEMENT_AND_COAGULATION_CASCA	−0.19742	0.02303	−4.95739
KEGG_ETHER_LIPID_METABOLISM	−0.12373	−0.01611	−4.83955
KEGG_P53_SIGNALING_PATHWAY	−0.16576	−0.03704	−4.7901
KEGG_ALPHA_LINOLENIC_ACID_METABOLISM	−0.14856	0.008494	−4.46508
KEGG_PRION_DISEASES	−0.14409	−0.03086	−4.37548
KEGG_NICOTINATE_AND_NICOTINAMIDE_METABO	−0.11648	−0.00757	−4.06478
KEGG_SYSTEMIC_LUPUS_ERYTHEMATOSUS	−0.17244	0.008308	−3.88144
KEGG_RNA_POLYMERASE	0.343526391	0.025566473	11.5533587
KEGG_BASAL_TRANSCRIPTION_FACTORS	0.392728826	0.024174141	11.24354121
KEGG_RNA_DEGRADATION	0.301024519	0.004463213	10.55191561
KEGG_PURINE_METABOLISM	0.135874337	0.005149463	10.44143427
KEGG_PYRIMIDINE_METABOLISM	0.239242189	0.009011862	10.41595569
KEGG_NEUROACTIVE_LIGAND_RECEPTOR_INTERA	−0.23329498	−0.01269365	−10.3388519
KEGG_N_GLYCAN_BIOSYNTHESIS	0.242639638	0.037772467	9.952752337
KEGG_CITRATE_CYCLE_TCA_CYCLE	0.250696153	0.012379116	9.207961649
KEGG_AMINOACYL_TRNA_BIOSYNTHESIS	0.235454972	0.017217915	9.112191971
KEGG_LINOLEIC_ACID_METABOLISM	−0.23944119	−0.04131769	−9.05620993
KEGG_CUPROPTOSIS	0.352442134	−0.00313381	9.012571505
KEGG_CALCIUM_SIGNALING_PATHWAY	−0.17580545	−0.01435262	−8.80908583
KEGG_SPLICEOSOME	0.292468432	0.016449469	8.459234927
KEGG_APOPTOSIS	0.133369138	0.015753211	8.443294698
KEGG_AMINO_SUGAR_AND_NUCLEOTIDE_SUGAR_	0.196199771	0.001322688	8.139285561
KEGG_DRUG_METABOLISM_CYTOCHROME_P450	−0.16903141	−0.02115796	−8.1349272

A enrichment analysis		
***P*-Value**	**adj.*P*-Val**	**B**
1.00E-08	6.84E-07	9.629578967
1.15E-08	6.84E-07	9.497786533
1.75E-08	7.59E-07	9.102453535
2.13E-08	7.59E-07	8.91722985

2.93E-08	8.70E-07	8.616618679
2.09E-07	5.30E-06	6.768080906
5.42E-07	1.07E-05	5.870368531
6.16E-07	1.10E-05	5.750889809
1.20E-06	1.94E-05	5.125151692
2.09E-06	3.10E-05	4.605952454
2.63E-06	3.60E-05	4.391213771
1.14E-05	0.000134905	3.027153084
1.68E-05	0.000186621	2.665744429
6.16E-05	0.000570227	1.462558314
0.000127883	0.001071823	0.789685106
7.11E-27	1.26E-24	50.26455005
1.05E-25	9.34E-24	47.6163656
3.73E-23	2.21E-21	41.84306186
9.35E-23	4.11E-21	40.93963993
1.15E-22	4.11E-21	40.73206604
2.18E-22	5.56E-21	40.10567513
5.11E-21	1.14E-19	37.01010973
1.82E-18	3.60E-17	31.24425758
3.79E-18	6.75E-17	30.52366346
5.82E-18	9.41E-17	30.10472874
8.11E-18	1.20E-16	29.77934674
3.76E-17	5.15E-16	28.2759355
4.98E-16	5.91E-15	25.7458195
5.59E-16	5.91E-15	25.63222723
4.97E-15	4.56E-14	23.4947501
5.12E-15	4.56E-14	23.46451168
