# Supplementary material for: Investigation of cuproptosis regulator-mediated modification patterns and SLC30A7 function in GBM
Source: Aging (Albany NY). 2024 Feb 22;16(4):3554–82. doi: 10.18632/aging.205545 (PMC10929835; doi:10.18632/aging.205545)
Supplement: Supplementary Tables 4 and 6-8 [file aging-16-205545-s003.docx]

| **Supplementary Table 4. ESTIMATE score of each sample.** | | | | | |
| --- | --- | --- | --- | --- | --- |
| ID | Cupcluster | geneCluster | StromalScore | ImmuneScore | ESTIMATEScore |
| FB006 | A | A | -361.424861 | -24.6009987 | -386.025859 |
| FB013 | A | A | -397.290792 | 161.4427429 | -235.848049 |
| FB017 | A | A | -18.4170046 | 398.8221112 | 380.4051066 |
| FB108 | A | A | 231.8609971 | 801.4519227 | 1033.31292 |
| FB206 | A | A | 1562.403189 | 1845.481097 | 3407.884285 |
| FB402 | A | A | 499.9679362 | 1245.716123 | 1745.684059 |
| FB403 | A | A | 826.097544 | 1221.84423 | 2047.941774 |
| FB404 | A | A | 634.5229349 | 1548.212568 | 2182.735503 |
| FB405 | A | A | 559.8290596 | 1276.410032 | 1836.239092 |
| FB406 | A | A | -437.834725 | -396.866681 | -834.701405 |
| FB407 | A | A | 716.0916356 | 1125.969027 | 1842.060662 |
| FB408 | A | A | 205.9906198 | 909.3320506 | 1115.32267 |
| FB409 | A | A | 410.2455704 | 555.6419482 | 965.8875186 |
| FB410 | A | A | 580.120982 | 861.2049111 | 1441.325893 |
| FB412 | A | A | 591.8561995 | 889.6548368 | 1481.511036 |
| FB414 | A | A | 883.742389 | 632.9260489 | 1516.668438 |
| FB416 | A | A | -378.097636 | -159.779941 | -537.877577 |
| FB417 | A | A | 394.9997042 | 506.662933 | 901.6626372 |
| FB418 | A | A | -517.18793 | -324.01215 | -841.20008 |
| FB419 | A | A | -377.179519 | -130.57911 | -507.758629 |
| FB420 | A | A | 277.4458659 | 801.067425 | 1078.513291 |
| FB425 | A | A | 797.5352992 | 1046.497294 | 1844.032593 |
| FB426 | A | A | 1016.344766 | 1270.883858 | 2287.228624 |
| FB427 | A | A | 208.1407483 | 312.6942648 | 520.8350131 |
| FB433 | A | A | 122.3471494 | 375.7936274 | 498.1407768 |
| FB434 | A | A | -123.489754 | 444.9573779 | 321.4676237 |
| FB435 | A | A | -71.5843756 | 571.0249862 | 499.4406106 |
| FB436 | A | A | 454.3893116 | 626.9841922 | 1081.373504 |
| FB438 | A | A | -205.968845 | 109.0897957 | -96.8790495 |
| FB439 | A | A | -74.7330529 | 11.49167394 | -63.241379 |
| FB502 | A | A | -43.0542693 | 694.4679697 | 651.4137004 |
| FB503 | A | A | -132.151964 | -122.706202 | -254.858166 |
| FB506 | A | A | 190.2776721 | 423.5416611 | 613.8193332 |
| FB508 | A | A | -200.718593 | 168.339718 | -32.3788748 |
| FB516 | A | A | 7.892277705 | 567.3786634 | 575.2709411 |
| FB553 | A | A | -279.32622 | 236.8644401 | -42.4617801 |
| FB554 | A | A | 21.34457043 | 301.7109157 | 323.0554861 |
| FB555 | A | A | 306.5542678 | 1114.881672 | 1421.435939 |
| FB556 | A | A | -236.043824 | 357.3590421 | 121.3152186 |
| FB557 | A | A | 707.9684338 | 1172.987204 | 1880.955638 |
| FB558 | A | A | -554.842701 | -206.863558 | -761.706259 |
| FB559 | A | A | -416.242287 | 3.685169025 | -412.557118 |
| FB561 | A | A | 515.5410679 | 926.2197381 | 1441.760806 |
| FB564 | A | A | -453.144413 | 174.114993 | -279.02942 |
| FB566 | A | A | 269.5290255 | 956.676344 | 1226.205369 |
| FB567 | A | A | 386.8915221 | 778.5278739 | 1165.419396 |
| FB568 | A | A | 81.38178417 | 763.5654172 | 844.9472014 |
| FB569 | A | A | 99.02972484 | 288.3455764 | 387.3753012 |

| GSM405213 | B | B | 819.3797225 | 894.2348138 | 1713.614536 |
| --- | --- | --- | --- | --- | --- |
| GSM405214 | B | C | 1024.882226 | 1215.48636 | 2240.368586 |
| GSM405215 | C | B | -255.836376 | 73.79292125 | -182.043454 |
| GSM405216 | B | A | -721.583326 | -576.708719 | -1298.29205 |
| GSM405217 | B | B | 616.9048128 | 1647.531322 | 2264.436135 |
| GSM405218 | B | C | 1248.678034 | 967.4027121 | 2216.080746 |
| GSM405219 | B | C | 369.9158205 | 905.8096135 | 1275.725434 |
| GSM405220 | C | C | 19.88296713 | 117.3727306 | 137.2556978 |
| GSM405221 | C | C | 133.1784617 | 546.8501812 | 680.0286429 |
| GSM405222 | B | B | -150.784411 | -90.6661057 | -241.450517 |
| GSM405223 | B | B | -495.940594 | -60.6439562 | -556.58455 |
| GSM405224 | B | B | 306.4270942 | 553.186124 | 859.6132183 |
| GSM405228 | B | B | 396.1251901 | 913.3568734 | 1309.482064 |
| GSM405229 | B | B | 45.33499449 | 270.5170361 | 315.8520306 |
| GSM405230 | B | C | -295.696054 | 258.4315393 | -37.2645148 |
| GSM405231 | C | B | -67.1363914 | 179.4609758 | 112.3245843 |
| GSM405232 | B | B | 25.96583364 | -358.789233 | -332.823399 |
| GSM405233 | B | C | 1126.977224 | 1340.274033 | 2467.251257 |
| GSM405234 | B | B | -330.861261 | -299.797358 | -630.65862 |
| GSM405235 | B | C | 1179.365685 | 1532.291319 | 2711.657005 |
| GSM405236 | B | C | 1014.745111 | 935.0351133 | 1949.780224 |
| GSM405237 | B | C | 193.2453662 | 1065.368148 | 1258.613514 |
| GSM405238 | B | B | -23.7269805 | 472.1926165 | 448.465636 |
| GSM405239 | C | C | 1264.326698 | 1449.590166 | 2713.916864 |
| GSM405240 | B | B | -48.6909553 | 634.5723336 | 585.8813784 |
| GSM405241 | C | C | 534.8804368 | 1078.305582 | 1613.186019 |
| GSM405242 | C | B | 13.70202739 | 523.5064054 | 537.2084327 |
| GSM405243 | B | B | 564.6745792 | 1100.543006 | 1665.217586 |
| GSM405244 | B | A | -86.3360455 | 357.0889227 | 270.7528772 |
| GSM405245 | B | B | 631.9940112 | 1254.108124 | 1886.102136 |
| GSM405246 | B | A | -1267.30116 | -1001.37675 | -2268.67791 |
| GSM405247 | C | B | -207.302396 | 153.2986836 | -54.0037125 |
| GSM405248 | B | B | 430.9580554 | 679.8336705 | 1110.791726 |
| GSM405249 | B | B | -168.886172 | 229.996248 | 61.11007642 |
| GSM405251 | B | A | -417.557084 | 23.2000674 | -394.357016 |
| GSM405252 | C | C | 258.4414684 | 798.8811078 | 1057.322576 |
| GSM405253 | B | B | 13.67636055 | -1.16451801 | 12.51184254 |
| GSM405254 | B | C | 117.9532625 | 595.154949 | 713.1082115 |
| GSM405255 | C | B | 415.9572729 | 610.7719726 | 1026.729246 |
| GSM405260 | C | C | 573.6251756 | 453.6742737 | 1027.299449 |
| GSM405262 | B | B | -312.530333 | -159.967138 | -472.49747 |
| GSM405263 | B | C | 266.3356333 | 533.2640921 | 799.5997254 |
| GSM405264 | C | C | 209.0753047 | 510.7336038 | 719.8089085 |
| GSM405266 | B | B | -111.333552 | 521.979018 | 410.645466 |
| GSM405267 | B | B | -516.315955 | 34.3743085 | -481.941646 |
| GSM405268 | B | B | 575.7797194 | 1323.787536 | 1899.567255 |
| GSM405269 | B | C | 436.756224 | 880.6894851 | 1317.445709 |
| GSM405270 | B | C | 759.8430783 | 1239.29247 | 1999.135548 |
| GSM405271 | B | B | 23.33959021 | 261.2136459 | 284.5532362 |
| GSM405274 | B | C | 37.61135847 | 671.0344352 | 708.6457937 |

| GSM405275 | B | B | -375.639976 | 492.8407804 | 117.200804 |
| --- | --- | --- | --- | --- | --- |
| GSM405276 | C | C | 696.1923877 | 1128.736662 | 1824.92905 |
| GSM405278 | B | B | 920.5409849 | 1496.914771 | 2417.455756 |
| GSM405280 | B | C | 135.9304075 | 755.4332771 | 891.3636846 |
| GSM405282 | C | C | 651.0849416 | 1221.956555 | 1873.041496 |
| GSM405290 | B | C | 607.4902794 | 437.6543858 | 1045.144665 |
| GSM405292 | B | B | -538.574924 | 292.4577307 | -246.117193 |
| GSM405293 | B | C | 760.7572506 | 768.4781898 | 1529.23544 |
| GSM405294 | B | B | 282.0311678 | -148.892833 | 133.1383349 |
| GSM405296 | B | B | -126.67732 | 160.774318 | 34.09699815 |
| GSM405297 | B | B | 80.09201832 | 644.2945882 | 724.3866065 |
| GSM405299 | B | C | -90.5459753 | 276.4696086 | 185.9236333 |
| GSM405301 | C | C | 751.3767689 | 913.5121267 | 1664.888896 |
| GSM405302 | C | B | 2.643070409 | 147.9211386 | 150.564209 |
| GSM405303 | B | B | -458.275618 | 154.6542387 | -303.621379 |
| GSM405304 | C | C | 420.7634609 | 483.290294 | 904.0537549 |
| GSM405305 | C | C | 624.9840333 | 1322.321253 | 1947.305287 |
| GSM405307 | C | C | 485.1698649 | 1151.820799 | 1636.990664 |
| GSM405308 | C | B | -171.564872 | 610.7691094 | 439.2042374 |
| GSM405309 | C | B | -128.016933 | 532.8980783 | 404.8811451 |
| GSM405312 | C | B | -721.046888 | -205.554412 | -926.601299 |
| GSM405313 | C | B | 586.1711691 | 1315.639446 | 1901.810615 |
| GSM405314 | C | C | 661.8921587 | 756.2580022 | 1418.150161 |
| GSM405315 | B | A | -579.366991 | -406.066705 | -985.433695 |
| GSM405317 | C | C | 377.1435872 | 1197.339899 | 1574.483486 |
| GSM405320 | C | C | 579.59048 | 1213.583868 | 1793.174348 |
| GSM405322 | C | C | 788.1109903 | 1182.21378 | 1970.32477 |
| GSM405323 | C | B | -420.520312 | -388.883653 | -809.403965 |
| GSM405324 | C | C | 559.6775827 | 1388.555782 | 1948.233365 |
| GSM405326 | C | C | 342.2123245 | 382.338121 | 724.5504455 |
| GSM405328 | C | C | 305.1913051 | 526.4230722 | 831.6143773 |
| GSM405330 | B | B | -329.685665 | 194.2098682 | -135.475797 |
| GSM405337 | B | B | -675.631789 | -114.42131 | -790.053099 |
| GSM405340 | B | B | -208.115629 | 532.3477133 | 324.2320845 |
| GSM405345 | C | C | 614.172885 | 1277.066841 | 1891.239726 |
| GSM405349 | C | B | -713.505886 | -581.921839 | -1295.42773 |
| GSM405350 | C | A | -292.960207 | 181.2260651 | -111.734142 |
| GSM405351 | C | C | 223.1819272 | 699.503469 | 922.6853962 |
| GSM405352 | B | B | -776.164643 | -596.488788 | -1372.65343 |
| GSM405353 | B | C | 857.3822572 | 1091.892072 | 1949.274329 |
| GSM405356 | B | B | -146.608324 | 660.4884977 | 513.8801739 |
| GSM405362 | B | C | 25.37645033 | 135.5660331 | 160.9424834 |
| GSM405363 | B | B | -613.042273 | 972.0241809 | 358.9819082 |
| GSM405365 | B | B | 103.6347623 | 607.1859265 | 710.8206888 |
| GSM405367 | C | B | 40.63973955 | 927.8425312 | 968.4822707 |
| GSM405368 | B | B | 15.86277916 | 575.3004903 | 591.1632695 |
| GSM405369 | C | B | -739.120439 | -700.780144 | -1439.90058 |
| GSM405370 | B | B | -284.999902 | -762.743376 | -1047.74328 |
| GSM405371 | B | C | 482.2398729 | 771.9663086 | 1254.206182 |
| GSM405372 | C | B | -281.564403 | 4.275335138 | -277.289067 |

| GSM405373 | C | C | 251.6816625 | 935.819182 | 1187.500844 |
| --- | --- | --- | --- | --- | --- |
| GSM405374 | C | C | 660.8557768 | 1318.870021 | 1979.725798 |
| GSM405375 | C | C | 314.7643973 | 724.8310547 | 1039.595452 |
| GSM405376 | B | B | 200.3911346 | 180.3138339 | 380.7049685 |
| GSM405379 | C | B | 563.3909561 | 1061.400558 | 1624.791514 |
| GSM405384 | C | C | 349.1623371 | 837.756726 | 1186.919063 |
| GSM405385 | B | B | -414.246336 | 163.7396905 | -250.506645 |
| GSM405389 | C | B | -37.0486901 | -268.776501 | -305.825191 |
| GSM405391 | C | C | -11.5175397 | 103.227268 | 91.70972837 |
| GSM405392 | B | C | -581.562967 | -157.868487 | -739.431453 |
| GSM405393 | C | C | 10.6905913 | 326.8426056 | 337.5331969 |
| GSM405396 | C | C | 883.0711449 | 378.2734035 | 1261.344548 |
| GSM405397 | C | B | 253.770088 | 89.32689301 | 343.096981 |
| GSM405405 | C | C | 372.1052817 | 24.76499686 | 396.8702786 |
| GSM405412 | C | C | 349.1799058 | 923.5408328 | 1272.720739 |
| GSM405415 | C | C | 876.0032955 | 479.1167158 | 1355.120011 |
| GSM405416 | C | C | 372.3804997 | 937.685397 | 1310.065897 |
| GSM405417 | C | C | 208.0444534 | 308.4845618 | 516.5290152 |
| GSM405418 | C | B | -497.377675 | -543.047596 | -1040.42527 |
| GSM405419 | C | C | 99.16733247 | 389.7033725 | 488.870705 |
| GSM405422 | C | C | -16.9900444 | 173.7491626 | 156.7591182 |
| GSM405426 | C | C | 101.6270364 | 275.7687053 | 377.3957417 |
| GSM405427 | C | C | -305.465108 | 314.4213334 | 8.956225667 |
| GSM405428 | C | C | -536.949189 | -3.27325571 | -540.222445 |
| GSM405430 | C | C | 1068.744658 | 1384.069409 | 2452.814067 |
| GSM405431 | C | B | 236.2965466 | -425.222974 | -188.926427 |
| GSM405432 | C | C | -167.36992 | 47.07828884 | -120.291631 |
| GSM405434 | C | C | -832.243856 | -413.493502 | -1245.73736 |
| GSM405436 | C | B | -457.670263 | -307.599085 | -765.269348 |
| GSM405438 | C | C | -83.6958598 | 249.6005531 | 165.9046933 |
| GSM405440 | C | C | 525.4223831 | 1017.31703 | 1542.739413 |
| GSM405442 | C | C | 593.767181 | 1122.076081 | 1715.843262 |
| GSM405443 | C | C | -292.223048 | 131.3683613 | -160.854687 |
| GSM405446 | C | C | -395.781351 | 16.26874117 | -379.51261 |
| GSM405447 | C | C | 823.2691551 | 1046.000802 | 1869.269957 |
| GSM405448 | C | C | 595.8969484 | 1380.352122 | 1976.24907 |
| GSM405452 | C | C | -244.71037 | 263.4523492 | 18.74197895 |
| GSM405453 | C | C | 104.8534279 | 897.0358803 | 1001.889308 |
| GSM405454 | C | C | 604.4593351 | 635.9286174 | 1240.387952 |
| GSM405455 | C | C | 526.9855177 | 746.5957449 | 1273.581263 |
| GSM405458 | B | B | -35.7427735 | 198.3076028 | 162.5648293 |
| GSM405459 | C | C | 560.798806 | 1204.794766 | 1765.593572 |
| GSM405461 | C | B | -201.852995 | -58.4984242 | -260.35142 |
| GSM405463 | C | C | 679.0084179 | 1234.946603 | 1913.955021 |
| GSM405464 | C | C | -151.594379 | 349.8212307 | 198.2268522 |
| GSM405465 | C | C | -230.016721 | 322.3729801 | 92.35625954 |
| GSM405466 | C | C | -177.917892 | 853.5457018 | 675.6278098 |
| GSM405470 | C | B | 63.18575917 | 959.523268 | 1022.709027 |
| GSM405471 | C | C | 197.1575408 | 787.3436893 | 984.5012301 |
| GSM405472 | C | C | 102.4890738 | 1113.448896 | 1215.93797 |

| GSM405473 | C | C | 524.5042447 | 756.7390539 | 1281.243299 |
| --- | --- | --- | --- | --- | --- |
| GSM405474 | C | C | 253.9697221 | 480.9346262 | 734.9043483 |
| GSM405475 | B | B | -1051.34742 | -1265.86464 | -2317.21207 |
| GSM405477 | C | C | 95.1382611 | 732.778187 | 827.9164481 |
| GSM405479 | C | C | 525.4808982 | 1265.261161 | 1790.74206 |
| GSM187153 | B | A | -146.161582 | -218.186128 | -364.347709 |
| GSM187154 | B | A | -413.200506 | -460.721134 | -873.921639 |
| GSM187155 | B | A | 121.8352656 | 259.6926401 | 381.5279057 |
| GSM187157 | B | A | 310.607918 | 225.4090347 | 536.0169527 |
| GSM187158 | B | A | 217.061699 | 264.2998574 | 481.3615564 |
| GSM187159 | A | A | -488.885107 | -571.503991 | -1060.3891 |
| GSM187160 | A | A | -479.268102 | -486.335537 | -965.603639 |
| GSM187161 | A | A | -285.458967 | -54.3754598 | -339.834427 |
| GSM187162 | B | A | -98.4545026 | 654.1488202 | 555.6943176 |
| GSM187163 | A | A | -501.037043 | 78.12354845 | -422.913495 |
| GSM187164 | B | A | 146.5232954 | 904.4983733 | 1051.021669 |
| GSM187165 | B | A | 194.8907319 | 136.0185942 | 330.9093262 |
| GSM187166 | B | A | -140.73625 | -71.5827336 | -212.318983 |
| GSM187167 | B | A | -99.6005464 | 101.5857117 | 1.985165297 |
| GSM187168 | B | A | 671.9257222 | 361.845766 | 1033.771488 |
| GSM187169 | B | A | 346.7469467 | 207.5162117 | 554.2631584 |
| GSM187171 | B | A | 276.8092213 | 568.6434318 | 845.4526531 |
| GSM187172 | A | A | -253.665606 | 10.95174604 | -242.713859 |
| GSM187174 | A | A | 1099.491082 | 1404.358258 | 2503.849339 |
| GSM187175 | B | A | -306.6978 | -685.88638 | -992.58418 |
| GSM187176 | B | A | 57.51054104 | 546.6759968 | 604.1865379 |
| GSM187177 | B | A | 22.08415594 | 102.5054351 | 124.589591 |
| GSM187178 | B | A | -60.4014791 | 64.01741967 | 3.615940626 |
| GSM187179 | B | A | 219.9314246 | 323.4587141 | 543.3901387 |
| GSM187181 | A | A | -434.180564 | -125.092782 | -559.273346 |
| GSM187182 | B | A | 243.0834133 | 461.0474645 | 704.1308777 |
| GSM187183 | B | A | -284.787537 | 323.0256582 | 38.2381215 |
| GSM187184 | A | A | 347.6549285 | 148.0468979 | 495.7018264 |
| GSM187185 | B | A | -62.7583949 | -156.037281 | -218.795676 |
| GSM187186 | B | A | 1076.779865 | -29.6318367 | 1047.148028 |
| GSM187187 | A | A | -404.909571 | -281.734869 | -686.64444 |
| GSM187188 | B | A | -181.643937 | 98.3850001 | -83.2589366 |
| GSM187189 | B | A | 240.9913402 | 1056.472914 | 1297.464254 |
| GSM187190 | B | A | -681.195142 | -655.297436 | -1336.49258 |
| GSM187191 | B | A | 1033.374397 | 504.3884451 | 1537.762842 |
| GSM187192 | B | A | 919.7499739 | 1230.634621 | 2150.384595 |
| GSM187193 | A | A | -132.404422 | -504.896988 | -637.30141 |
| GSM187194 | B | A | 627.7933975 | 911.7587844 | 1539.552182 |
| GSM187195 | A | A | -44.5155178 | 547.25687 | 502.7413522 |
| GSM187196 | B | A | -684.749592 | -374.470969 | -1059.22056 |
| GSM187197 | B | A | 852.7962009 | 1391.812431 | 2244.608632 |
| GSM187198 | B | A | -204.252331 | 158.6460532 | -45.6062773 |
| GSM187199 | A | A | 134.0202194 | 371.1788637 | 505.1990831 |
| GSM187200 | A | A | 653.9463519 | 1287.007972 | 1940.954324 |
| GSM187201 | A | A | 177.8399267 | -20.0785117 | 157.7614151 |

| GSM187202 | B | A | 509.489951 | 686.4173417 | 1195.907293 |
| --- | --- | --- | --- | --- | --- |
| GSM187203 | B | A | -466.501958 | -437.890356 | -904.392313 |
| GSM187204 | B | A | -208.49659 | 69.60458066 | -138.89201 |
| GSM187205 | B | A | -379.116573 | -116.155871 | -495.272443 |
| GSM187206 | A | A | 558.4127816 | 1050.171562 | 1608.584344 |
| GSM187207 | C | A | 184.1810659 | 337.8752329 | 522.0562988 |
| GSM187208 | B | A | -98.7437504 | 16.4347131 | -82.3090373 |
| GSM187209 | B | A | 231.7157 | 666.5866343 | 898.3023343 |
| GSM187210 | B | A | 101.1617831 | 428.3535265 | 529.5153097 |
| GSM187211 | B | A | -338.613624 | -134.376334 | -472.989957 |
| GSM187212 | A | A | 221.0668891 | 268.8504436 | 489.9173327 |
| GSM187213 | B | A | 416.7044124 | 316.1145412 | 732.8189536 |
| GSM187214 | A | A | 139.3253886 | -263.579052 | -124.253664 |
| GSM187215 | A | A | 535.886155 | 1050.063977 | 1585.950132 |
| GSM187216 | B | A | -340.474282 | -349.880212 | -690.354495 |
| GSM187217 | B | A | 32.76746984 | 113.8467805 | 146.6142504 |
| GSM187218 | A | A | -501.878653 | -205.901134 | -707.779787 |
| GSM187219 | B | A | 143.1969764 | 473.0989029 | 616.2958794 |
| GSM187220 | B | A | -93.6365275 | -126.057829 | -219.694357 |
| GSM187221 | A | A | 646.8420734 | 1040.910307 | 1687.752381 |
| GSM187222 | B | A | 495.5232531 | 432.9118368 | 928.4350899 |
| GSM187223 | B | A | -263.039094 | -259.718405 | -522.757499 |
| GSM187224 | A | A | 911.0532175 | -451.161399 | 459.8918187 |
| GSM187225 | C | A | -361.670155 | 194.0470414 | -167.623114 |
| GSM187226 | B | A | 898.5136913 | 1034.948249 | 1933.46194 |
| GSM187227 | B | A | 471.8849027 | 750.6690759 | 1222.553979 |
| GSM187228 | A | A | -635.18152 | -20.190819 | -655.372339 |
| GSM187229 | B | A | -250.691999 | -252.94597 | -503.637969 |
| GSM187230 | B | A | 611.0913313 | 1340.30675 | 1951.398081 |
| GSM187231 | B | A | -106.013657 | -96.9178216 | -202.931479 |
| GSM187232 | B | A | -314.079272 | 292.2939157 | -21.7853563 |
| GSM187233 | B | A | -95.3715864 | 313.1935112 | 217.8219248 |
| GSM187234 | B | A | -58.000977 | -316.25588 | -374.256857 |
| GSM187235 | B | A | 750.9409303 | 1440.656686 | 2191.597616 |
| GSM187236 | B | A | 190.0481142 | 10.92613988 | 200.9742541 |
| 900-00-53-32 | B | C | -603.289166 | -139.703241 | -742.992407 |
| 900-00-5317 | B | B | -731.645495 | -793.632416 | -1525.27791 |
| 900-00-5338 | C | C | 81.74696232 | 102.4940968 | 184.2410591 |
| 900-00-5342 | C | C | 880.184424 | 1364.144377 | 2244.328802 |
| 900-00-5346 | C | C | 436.3225771 | 987.0895969 | 1423.412174 |
| 900-00-5379 | C | C | 28.28852666 | 259.0805315 | 287.3690581 |
| 900-00-5381 | B | C | 446.6633833 | 1093.766237 | 1540.42962 |
| 900-00-5384 | B | C | 450.4182787 | 442.3333394 | 892.7516181 |
| 900-00-5396 | C | C | 785.7416029 | 1236.607989 | 2022.349592 |
| 900-00-5404 | B | C | 25.43755089 | 290.8829144 | 316.3204653 |
| 900-00-5404- | B | C | 805.0445496 | 863.0046066 | 1668.049156 |
| 900-00-5413 | C | C | 451.9539655 | 1311.963509 | 1763.917475 |
| 900-00-5414 | B | B | -711.78026 | -115.029684 | -826.809944 |
| 900-00-5445 | C | B | -329.864813 | 131.2062282 | -198.658585 |
| 900-00-5458 | B | B | -158.56769 | 314.1072596 | 155.5395695 |

| 900-00-5462 | C | C | -102.290364 | 313.8279238 | 211.5375594 |
| --- | --- | --- | --- | --- | --- |
| 900-00-5488 | C | C | -521.330489 | -353.162616 | -874.493104 |
| 900-00-5489 | C | C | -266.151774 | 104.753503 | -161.398271 |
| 900-00-5540 | B | C | 303.0143884 | 213.5532846 | 516.567673 |
| 900-00-5541 | C | C | -828.2332 | -591.643446 | -1419.87665 |
| 900-00-5542 | C | C | -263.576236 | -31.5618391 | -295.138075 |
| 900-00-5543 | B | C | -200.892528 | 562.9137534 | 362.0212254 |
| 900-00-5544 | C | C | -135.296578 | -48.8742252 | -184.170803 |
| 900-00-5546 | C | C | 206.9449277 | -65.4670786 | 141.477849 |
| 900-00-5548 | C | C | 46.50084081 | 292.9857224 | 339.4865633 |
| 900-00-5551 | C | C | 303.8621257 | 590.8614283 | 894.723554 |
| 900-00-5554 | C | B | -381.501598 | -418.583671 | -800.085269 |
| 900-0052-99 | C | C | 256.3189251 | 777.0150864 | 1033.334011 |
| 900-0053-03 | C | B | -589.515035 | -215.308563 | -804.823597 |
| E09139 | B | C | -127.410935 | 529.7775476 | 402.366613 |
| E09192 | C | B | -53.1708118 | 116.9124157 | 63.74160397 |
| E09278 | C | B | -996.936139 | -852.893621 | -1849.82976 |
| E09331 | B | B | 448.5577553 | 541.4904602 | 990.0482155 |
| E09348 | B | B | -126.246325 | 842.8773365 | 716.6310115 |
| E09430 | B | C | 615.5277996 | 1280.715711 | 1896.24351 |
| E09451 | B | C | 153.3092772 | 975.7008257 | 1129.010103 |
| E09454 | B | B | -611.371934 | -856.239108 | -1467.61104 |
| E09483 | B | C | -44.6550844 | 616.4728364 | 571.817752 |
| E09489 | C | C | 1054.237631 | 784.2902216 | 1838.527852 |
| E09535 | B | C | -71.733934 | 236.0674373 | 164.3335032 |
| E09569 | B | B | -490.859131 | -113.960038 | -604.819169 |
| E09601 | C | C | 660.3756169 | 647.1271491 | 1307.502766 |
| E09602 | B | C | 317.7602255 | 667.3879776 | 985.1482032 |
| E09605 | B | C | 70.57489407 | 570.5559881 | 641.1308822 |
| E09606 | C | C | 382.7779775 | 1319.697858 | 1702.475836 |
| E09610 | B | C | -472.531713 | 294.2746089 | -178.257104 |
| E09615 | B | C | 198.1061159 | 319.0235563 | 517.1296722 |
| E09623 | B | C | -507.579764 | -309.110355 | -816.690118 |
| E09647 | B | C | -241.368961 | -26.5436832 | -267.912644 |
| E09649 | B | C | 524.1454098 | 721.9054217 | 1246.050832 |
| E09654 | B | C | 352.9960781 | 686.7593587 | 1039.755437 |
| E09670 | B | B | -123.95874 | -234.157586 | -358.116327 |
| E09690 | C | B | -506.559232 | -518.796465 | -1025.3557 |
| E09698 | C | C | 165.6674025 | 978.4006863 | 1144.068089 |
| E09722 | B | B | -774.709952 | -254.824329 | -1029.53428 |
| E09730 | B | C | 177.2818352 | 537.7840088 | 715.065844 |
| E09740 | C | C | 956.9082658 | 1096.228026 | 2053.136291 |
| E09744 | C | C | 255.930307 | 1208.237756 | 1464.168063 |
| E09759 | B | C | 646.5157023 | 1291.403088 | 1937.91879 |
| E09782 | C | C | 463.9880218 | 316.3634346 | 780.3514564 |
| E09786 | B | B | -283.580081 | 407.9111486 | 124.3310678 |
| E09787 | B | B | 337.7449866 | 580.3230377 | 918.0680243 |
| E09791 | C | C | -252.253882 | 389.3504135 | 137.096532 |
| E09800 | C | C | 1081.545921 | 1401.986163 | 2483.532084 |
| E09802 | B | C | -582.204079 | -578.168613 | -1160.37269 |

| E09810 | C | C | -226.772689 | -106.339174 | -333.111863 |
| --- | --- | --- | --- | --- | --- |
| E09832 | B | C | -319.272712 | -261.514662 | -580.787374 |
| E09833 | C | C | -435.208158 | -282.859405 | -718.067563 |
| E09846 | B | A | -497.911752 | -310.005444 | -807.917195 |
| E09847 | B | B | -329.976329 | -313.020972 | -642.997302 |
| E09852 | B | A | -533.948324 | -393.273011 | -927.221335 |
| E09907 | C | C | -195.270064 | 579.7174162 | 384.4473525 |
| E09917 | B | C | 635.8367326 | 797.0017701 | 1432.838503 |
| E09930 | B | B | 527.8656984 | 65.63822047 | 593.5039189 |
| E09938 | B | C | 436.5064384 | 723.9936018 | 1160.50004 |
| E09951 | B | C | 155.2432748 | 272.1188683 | 427.362143 |
| E09956 | B | B | -622.804297 | -198.2372 | -821.041496 |
| E09967 | C | C | 259.0729771 | 869.4115403 | 1128.484517 |
| E10002 | C | C | 480.1010431 | 402.4393144 | 882.5403575 |
| E10013 | C | C | -625.125624 | -435.037374 | -1060.163 |
| E10016 | B | C | 500.4506048 | 999.7182913 | 1500.168896 |
| E10026 | C | C | 107.0451833 | 440.2373669 | 547.2825502 |
| E10031 | B | C | 335.1588251 | 271.7493615 | 606.9081865 |
| E10041 | C | C | 100.1971743 | 528.1903404 | 628.3875147 |
| E10077 | B | C | 105.594014 | 1272.181213 | 1377.775227 |
| E10102 | C | C | -18.7124645 | 550.1714906 | 531.4590262 |
| E10110 | B | B | -1207.49886 | -735.88626 | -1943.38512 |
| E10144 | B | B | -397.438565 | -326.296926 | -723.735491 |
| E10158 | B | B | -47.5042741 | 877.5551119 | 830.0508378 |
| E10184 | B | B | -290.2891 | -272.439283 | -562.728384 |
| E10193 | B | B | 262.5737333 | 681.8049201 | 944.3786534 |
| E10211 | B | B | -592.581173 | -728.057163 | -1320.63834 |
| E10226 | B | B | -157.683889 | -230.840422 | -388.524311 |
| E10227 | C | C | 309.3005122 | 579.8709032 | 889.1714154 |
| E10250 | C | C | 106.5623087 | 620.2102707 | 726.7725794 |
| E10252 | B | B | -651.304513 | -526.941645 | -1178.24616 |
| E10258 | B | B | -249.172184 | -164.673296 | -413.84548 |
| E10262 | C | B | -474.444309 | 424.6457241 | -49.7985851 |
| E10267 | B | C | -955.918869 | -664.472353 | -1620.39122 |
| E10271 | C | C | -596.426614 | -584.52045 | -1180.94706 |
| E10284 | C | C | -116.757916 | 169.9526419 | 53.19472591 |
| E10290 | B | C | 646.4132646 | 309.3863634 | 955.799628 |
| E10292 | C | C | -597.716535 | 551.3417917 | -46.3747428 |
| E10300 | C | C | 575.8136275 | 706.1807635 | 1281.994391 |
| E10305 | C | C | 190.7073201 | 624.1211317 | 814.8284518 |
| E10306 | C | C | -301.77087 | -88.6199071 | -390.390777 |
| E10312 | B | B | -238.516909 | -47.0205018 | -285.537411 |
| E10313 | C | C | 401.5605897 | 438.8113905 | 840.3719802 |
| E10318 | C | C | 401.4645006 | 961.4717062 | 1362.936207 |
| E10433 | B | B | 402.1455143 | 932.926563 | 1335.072077 |
| E10444 | C | C | -312.801286 | 63.95362662 | -248.847659 |
| E10462 | B | C | 393.4906431 | 915.1611781 | 1308.651821 |
| E10488 | B | C | 1189.345829 | 1438.209309 | 2627.555137 |
| E10514 | B | B | 124.4227745 | 622.0481674 | 746.4709419 |
| E10551 | B | C | 388.3654708 | 661.1303361 | 1049.495807 |

| E50057 | B | B | -212.800957 | -398.356042 | -611.156998 |
| --- | --- | --- | --- | --- | --- |
| E50074 | B | C | -589.073234 | -446.390807 | -1035.46404 |
| E50091 | B | B | -602.418293 | -126.722782 | -729.141075 |
| E50123 | C | C | -594.167766 | -235.805837 | -829.973602 |
| HF0024 | B | C | -44.4124529 | 175.3128987 | 130.9004457 |
| HF0031 | C | C | 428.122347 | 1189.515362 | 1617.637709 |
| HF0048 | B | B | 6.35475698 | 192.3361884 | 198.6909454 |
| HF0050 | B | C | 707.3537358 | 915.6377732 | 1622.991509 |
| HF0066 | B | C | -642.439478 | -447.9451 | -1090.38458 |
| HF0138 | C | C | -151.752153 | 132.0140751 | -19.7380777 |
| HF0142 | C | C | -616.399551 | -436.503119 | -1052.90267 |
| HF0180 | C | C | -275.146213 | 35.74981187 | -239.396401 |
| HF0268 | B | B | -152.037643 | 233.6510382 | 81.61339478 |
| HF0300.3 | C | C | 238.2495707 | 575.5035626 | 813.7531333 |
| HF0408 | C | C | -582.922705 | -376.441414 | -959.36412 |
| HF0442.5 | B | B | -174.88224 | 79.67355323 | -95.2086865 |
| HF0445 | C | C | -74.0589777 | 618.2572104 | 544.1982328 |
| HF0505 | B | A | -688.229347 | -719.851739 | -1408.08109 |
| HF0520 | C | C | 126.6550721 | 672.917257 | 799.5723291 |
| HF0543 | C | C | 884.6971622 | 888.1777157 | 1772.874878 |
| HF0583 | C | C | 38.15673367 | 127.8048248 | 165.9615585 |
| HF0627 | B | C | -32.4605339 | 264.4902161 | 232.0296822 |
| HF0654 | B | B | -399.208813 | 94.54827722 | -304.660536 |
| HF0790 | C | C | -226.702219 | 592.0029713 | 365.3007524 |
| HF0891 | C | C | -814.738861 | -584.656235 | -1399.3951 |
| HF0894 | C | C | -65.4469506 | 251.3627736 | 185.915823 |
| HF0963 | B | C | -434.740772 | -260.251915 | -694.992687 |
| HF0986 | B | B | -601.976368 | -365.339814 | -967.316182 |
| HF0990 | B | B | -384.294501 | -353.523437 | -737.817938 |
| HF0992 | B | B | -412.405241 | -223.926425 | -636.331665 |
| HF0996 | B | B | -1020.45794 | -740.248455 | -1760.7064 |
| HF1058 | B | A | -645.817475 | -822.873258 | -1468.69073 |
| HF1077 | B | C | 181.7187366 | 467.6325256 | 649.3512622 |
| HF1078 | B | C | 139.8140948 | 554.2696549 | 694.0837497 |
| HF1097 | B | C | 959.1162795 | 304.2798228 | 1263.396102 |
| HF1122 | C | C | -339.284923 | 64.57921394 | -274.705709 |
| HF1137 | C | C | 53.1585812 | 429.7448979 | 482.9034791 |
| HF1139 | B | C | -266.752708 | -316.216631 | -582.969339 |
| HF1178 | B | C | -8.64748927 | 244.363173 | 235.7156837 |
| HF1191 | C | C | -372.232038 | -60.4983848 | -432.730423 |
| HF1220 | C | C | 339.1726053 | 794.7293172 | 1133.901923 |
| HF1262 | C | C | 596.6205409 | 364.9508042 | 961.5713451 |
| HF1269 | C | C | 82.66043257 | 276.6045607 | 359.2649932 |
| HF1292 | B | C | 878.8214771 | 986.48486 | 1865.306337 |
| HF1297 | B | C | -395.891181 | 16.24910181 | -379.642079 |
| HF1318 | C | C | 170.5752704 | 419.8218356 | 590.3971059 |
| HF1338 | B | B | -460.12015 | -271.891148 | -732.011298 |
| HF1356 | B | C | 887.0168502 | 1105.764816 | 1992.781666 |
| HF1382 | C | C | -132.460106 | -469.701758 | -602.161863 |
| HF1397 | C | C | -438.505823 | -167.338589 | -605.844411 |

| HF1469 | B | B | -466.959304 | -317.450156 | -784.409461 |
| --- | --- | --- | --- | --- | --- |
| HF1492 | B | B | -671.711333 | -666.183934 | -1337.89527 |
| HF1509 | C | C | -502.264448 | 117.1631013 | -385.101347 |
| HF1517 | C | C | -131.086325 | -119.519812 | -250.606137 |
| HF1534 | B | B | -219.058106 | -120.692107 | -339.750213 |
| HF1538 | B | C | 156.4834939 | 205.1256264 | 361.6091203 |
| HF1540 | C | C | -76.7038113 | -334.296486 | -411.000297 |
| HF1585 | B | C | 165.0450092 | 556.5135408 | 721.55855 |
| HF1589 | C | C | -257.042246 | 88.50988729 | -168.532358 |
| HF1608 | C | C | -254.533105 | 393.6975831 | 139.1644781 |
| HF1618 | C | C | -164.495765 | 46.85000865 | -117.645757 |
| HF1628 | C | C | -447.314386 | -219.06233 | -666.376716 |
| HF1640 | B | A | -716.002738 | -801.714419 | -1517.71716 |
| HF1667 | B | B | -757.476013 | -567.808793 | -1325.28481 |
| HF1671 | B | B | -26.6303348 | 263.4170876 | 236.7867528 |
| MD545226 | C | B | -856.11735 | -560.262235 | -1416.37959 |
| MD602958 | C | C | 934.2694001 | 1463.455882 | 2397.725282 |
| MD607103 | C | C | 646.7889684 | 1265.66905 | 1912.458018 |
| MD607216 | C | C | -159.746538 | 260.3416032 | 100.5950652 |
| MD608660 | B | C | -559.787329 | -146.800228 | -706.587557 |
| MD621233 | B | A | -696.507921 | -117.661229 | -814.16915 |

| **Supplementary Table 6. Prognostic analysis of 205 cuproptosis phenotype-related genes using a univariate Cox regr.** | | | | |
| --- | --- | --- | --- | --- |
| Gene | HR | HR.95L | HR.95H | pvalue |
| GFM1 | 1.226818676 | 1.099050444 | 1.369440387 | 0.000269333 |
| MITD1 | 1.174436344 | 1.062763637 | 1.297843356 | 0.001610187 |
| RPAP3 | 1.220273258 | 1.097048535 | 1.357339057 | 0.00024702 |
| COPB1 | 1.272007118 | 1.116836804 | 1.44873638 | 0.000289281 |
| RIOK3 | 1.135198376 | 1.036432182 | 1.243376437 | 0.006324016 |
| ETFA | 1.20376939 | 1.067530668 | 1.357394956 | 0.002475466 |
| CAPN7 | 1.173399909 | 1.041612092 | 1.321861908 | 0.008521289 |
| DIS3 | 1.096602938 | 1.000000186 | 1.20253778 | 0.049999537 |
| SCO1 | 1.375351183 | 1.194555197 | 1.58351065 | 9.33E-06 |
| COPS8 | 1.147807204 | 1.041716415 | 1.264702521 | 0.005337764 |
| GCLM | 1.103316123 | 1.023762197 | 1.189051979 | 0.01002344 |
| EDEM3 | 1.143007521 | 1.029085459 | 1.269541009 | 0.012589491 |
| MRPL13 | 1.199941917 | 1.079798222 | 1.333453394 | 0.000708476 |
| ACP1 | 1.191043317 | 1.071547465 | 1.323864998 | 0.001191016 |
| PRMT6 | 1.176677277 | 1.073679631 | 1.289555444 | 0.000499429 |
| RANBP9 | 1.109161806 | 1.010670542 | 1.217251183 | 0.028986086 |
| IFRD1 | 1.32225396 | 1.191174743 | 1.467757392 | 1.57E-07 |
| GNAI3 | 1.240723556 | 1.098622942 | 1.401204073 | 0.000509842 |
| EXOC4 | 1.126386048 | 1.029938156 | 1.231865741 | 0.009164762 |
| DNAJC10 | 1.23221885 | 1.116042857 | 1.360488341 | 3.58E-05 |
| SH3GLB1 | 1.220220126 | 1.09139041 | 1.364257138 | 0.000472056 |
| SLC30A7 | 1.134557015 | 1.026364439 | 1.25415454 | 0.013553414 |
| ATP6AP2 | 1.09253926 | 1.010940579 | 1.180724227 | 0.025436424 |
| MGAT4B | 1.175739407 | 1.05798526 | 1.306599634 | 0.002639917 |
| SLC33A1 | 1.146116195 | 1.028231013 | 1.27751674 | 0.013790023 |
| KCTD20 | 1.155757313 | 1.053141797 | 1.26837143 | 0.002277497 |
| AGPAT5 | 1.221913267 | 1.09918486 | 1.358344793 | 0.000206395 |
| NAF1 | 1.08441932 | 1.009562032 | 1.164827147 | 0.026369142 |
| ARF4 | 1.225060536 | 1.106749706 | 1.356018717 | 8.95E-05 |
| PGK1 | 1.143942514 | 1.057767567 | 1.237138023 | 0.000764363 |
| LACTB | 1.092127573 | 1.004843812 | 1.186993065 | 0.038110449 |
| ABCD3 | 1.12606752 | 1.023777869 | 1.23857733 | 0.014541224 |
| ARPC1A | 1.210530173 | 1.090671249 | 1.343560949 | 0.000328786 |
| EGLN1 | 1.093515006 | 1.001849932 | 1.19356705 | 0.045355739 |
| FBXW8 | 1.100235942 | 1.00869199 | 1.200087975 | 0.031144224 |
| DSTN | 1.122654912 | 1.034516014 | 1.218303084 | 0.005547357 |
| SCOC | 1.090962942 | 1.005596132 | 1.183576688 | 0.036242098 |
| NEU1 | 1.144609071 | 1.022317264 | 1.28152969 | 0.019138298 |
| APLP2 | 1.143490431 | 1.032007659 | 1.267016145 | 0.010408774 |
| KDELR2 | 1.247262172 | 1.121110531 | 1.387608879 | 4.88E-05 |
| HNRNPC | 1.111876836 | 1.012532811 | 1.220967937 | 0.026365678 |
| COPB2 | 1.285132477 | 1.136407485 | 1.453321546 | 6.40E-05 |
| FNTA | 1.169038469 | 1.06034337 | 1.288875831 | 0.0017084 |
| SLC4A7 | 1.104123577 | 1.000152624 | 1.21890284 | 0.049647518 |
| SH3BGRL3 | 1.173211064 | 1.074086449 | 1.281483629 | 0.000389887 |
| CHPT1 | 1.223823883 | 1.120033346 | 1.337232416 | 7.93E-06 |
| PLS3 | 1.200597374 | 1.09994614 | 1.31045876 | 4.27E-05 |
| HSPA13 | 1.1311413 | 1.026763475 | 1.246129875 | 0.012608189 |

| ARPC5 | 1.129735408 | 1.029763527 | 1.239412795 | 0.009869174 |
| --- | --- | --- | --- | --- |
| CKAP4 | 1.114693932 | 1.031912557 | 1.204116136 | 0.005818161 |
| POLR2J | 1.233149584 | 1.106021631 | 1.374889835 | 0.000159861 |
| RPL22L1 | 1.15735721 | 1.057160401 | 1.267050591 | 0.001560973 |
| RECQL | 1.142477969 | 1.04305547 | 1.251377272 | 0.004138118 |
| GNS | 1.127857829 | 1.041794869 | 1.221030474 | 0.00296834 |
| HEXB | 1.279566944 | 1.147740653 | 1.426534435 | 8.83E-06 |
| FBXO6 | 1.196417217 | 1.093695871 | 1.308786286 | 9.02E-05 |
| RPS26 | 1.1944459 | 1.080435074 | 1.320487497 | 0.000517653 |
| MANEA | 1.119728256 | 1.000112075 | 1.253650865 | 0.049773169 |
| KBTBD2 | 1.107731621 | 1.013960889 | 1.210174236 | 0.023379001 |
| CHST2 | 1.056504328 | 1.004902932 | 1.110755438 | 0.031444661 |
| SLC16A4 | 1.202055209 | 1.116150211 | 1.294571924 | 1.15E-06 |
| NRCAM | 1.234092047 | 1.121113389 | 1.358455973 | 1.76E-05 |
| ERI1 | 1.158171137 | 1.038424703 | 1.291726187 | 0.008361961 |
| CHIC2 | 1.120076058 | 1.038391273 | 1.208186557 | 0.00333498 |
| DNAJB9 | 1.136663983 | 1.038878153 | 1.243654038 | 0.005254731 |
| GYS1 | 1.134768837 | 1.052975323 | 1.222915947 | 0.000925065 |
| KLF10 | 1.171249212 | 1.060482853 | 1.293585006 | 0.001817679 |
| CD164 | 1.106170734 | 1.026019423 | 1.192583362 | 0.008556377 |
| DYNLT1 | 1.201365228 | 1.076767196 | 1.340381112 | 0.001023801 |
| FHL1 | 1.098621344 | 1.0132956 | 1.191132041 | 0.022598237 |
| MRPS28 | 1.147872401 | 1.027823527 | 1.281942877 | 0.014409787 |
| FSCN1 | 1.124026814 | 1.033859764 | 1.222057693 | 0.006135048 |
| CBLN2 | 0.939099221 | 0.883820643 | 0.997835199 | 0.042358788 |
| PLEKHF2 | 1.14599401 | 1.044438371 | 1.257424379 | 0.00399789 |
| ARL4C | 1.259045235 | 1.158280473 | 1.368576041 | 6.22E-08 |
| BTN3A2 | 1.171564777 | 1.067235687 | 1.286092701 | 0.00087661 |
| IRF2 | 1.099001733 | 1.013808923 | 1.191353501 | 0.021842383 |
| NOP10 | 1.232378687 | 1.09094724 | 1.392145442 | 0.000780733 |
| FAM162A | 1.215941573 | 1.098200911 | 1.346305484 | 0.000168116 |
| RPL35 | 1.096499935 | 1.003885973 | 1.197658041 | 0.040745917 |
| RNF149 | 1.133632004 | 1.051211398 | 1.222514827 | 0.001126902 |
| SALL1 | 1.136539601 | 1.057509592 | 1.221475695 | 0.000500287 |
| POLR2E | 1.134754092 | 1.024375262 | 1.257026499 | 0.015468539 |
| DUSP6 | 1.131070341 | 1.048654644 | 1.21996324 | 0.001419215 |
| PPDPF | 1.0854988 | 1.016487245 | 1.159195701 | 0.014369179 |
| GTF2H2 | 1.10335876 | 1.024588444 | 1.188184935 | 0.009248052 |
| IKBIP | 1.220748479 | 1.102795472 | 1.351317527 | 0.000119447 |
| P4HB | 1.163638589 | 1.058621409 | 1.279073664 | 0.001686958 |
| PLOD3 | 1.345050579 | 1.19846542 | 1.509564673 | 4.78E-07 |
| SNAP91 | 0.930841 | 0.888934906 | 0.974722628 | 0.002293759 |
| PDE4B | 1.082534901 | 1.011111438 | 1.159003614 | 0.022770012 |
| HAUS1 | 1.156953634 | 1.045970586 | 1.279712575 | 0.004604244 |
| MAD2L1 | 1.090167064 | 1.005547297 | 1.181907834 | 0.03624622 |
| SCRG1 | 1.092863258 | 1.005530329 | 1.187781279 | 0.036640235 |
| HSPB1 | 1.211029038 | 1.107445105 | 1.324301606 | 2.70E-05 |
| RNF145 | 1.179851226 | 1.085338164 | 1.282594642 | 0.000103494 |
| PAICS | 1.181422256 | 1.058213759 | 1.318975996 | 0.003008294 |
| RHOA | 1.087658254 | 1.016971347 | 1.163258414 | 0.014253384 |

| SAE1 | 1.118521619 | 1.022520406 | 1.223536083 | 0.014429943 |
| --- | --- | --- | --- | --- |
| FJX1 | 1.113126245 | 1.024427022 | 1.209505422 | 0.011419784 |
| CEBPB | 1.144000632 | 1.057677637 | 1.23736893 | 0.000777086 |
| SMC4 | 1.147297083 | 1.052881794 | 1.250178893 | 0.00171246 |
| PRDX4 | 1.189430894 | 1.069602757 | 1.322683439 | 0.001365133 |
| IGFBP5 | 1.158970036 | 1.077280293 | 1.24685428 | 7.62E-05 |
| LSM4 | 1.181440829 | 1.057350849 | 1.320093925 | 0.003230218 |
| MIF | 1.160668783 | 1.057719139 | 1.273638696 | 0.001666066 |
| OSMR | 1.16010692 | 1.062887602 | 1.266218613 | 0.000881828 |
| SDC2 | 1.159510536 | 1.065874932 | 1.261371895 | 0.000571188 |
| SEC61A1 | 1.140663548 | 1.017497898 | 1.278738099 | 0.023976233 |
| PPIB | 1.194609615 | 1.059215163 | 1.347310899 | 0.003763902 |
| CASP1 | 1.170292633 | 1.080293587 | 1.267789482 | 0.000117331 |
| FAS | 1.117261409 | 1.032725605 | 1.208717059 | 0.005742513 |
| PLA2G4A | 1.280448627 | 1.178296508 | 1.391456797 | 5.62E-09 |
| EMILIN1 | 1.125640915 | 1.050450121 | 1.206213835 | 0.000792703 |
| S100A16 | 1.273717776 | 1.158384431 | 1.400534165 | 5.85E-07 |
| CMBL | 1.109019822 | 1.014572645 | 1.212259144 | 0.022694702 |
| SLC43A3 | 1.313341197 | 1.177592582 | 1.464738421 | 9.75E-07 |
| NUCB1 | 1.117766921 | 1.028039415 | 1.215325863 | 0.009116 |
| FLNA | 1.100053479 | 1.022358995 | 1.183652379 | 0.010720829 |
| GLIPR1 | 1.092784176 | 1.01030172 | 1.182000616 | 0.026696673 |
| CD99 | 1.307066829 | 1.180214617 | 1.447553411 | 2.73E-07 |
| CD58 | 1.265806388 | 1.167023536 | 1.372950726 | 1.30E-08 |
| IFI16 | 1.119069794 | 1.027366119 | 1.218959026 | 0.009912509 |
| PDGFC | 1.171565162 | 1.071623088 | 1.280828069 | 0.000500501 |
| FN1 | 1.206330063 | 1.102208361 | 1.32028777 | 4.64E-05 |
| FCER1G | 1.117133513 | 1.040608473 | 1.199286109 | 0.002217692 |
| FZD6 | 1.073093546 | 1.009530868 | 1.140658294 | 0.023546641 |
| HERPUD2 | 1.233911623 | 1.132912552 | 1.343914754 | 1.41E-06 |
| CDKN3 | 1.134803862 | 1.037666156 | 1.241034796 | 0.005609232 |
| EIF4EBP1 | 1.150019518 | 1.039830725 | 1.271884799 | 0.006528079 |
| OXTR | 1.107172955 | 1.040499355 | 1.178118897 | 0.001314516 |
| SHCBP1 | 1.179646689 | 1.086403343 | 1.280892882 | 8.41E-05 |
| GLUL | 1.101544296 | 1.021936028 | 1.187354006 | 0.011507079 |
| LUM | 1.051911096 | 1.001137107 | 1.105260154 | 0.044964356 |
| RNASE4 | 1.16742206 | 1.08603327 | 1.254910236 | 2.69E-05 |
| ARRDC3 | 1.239046523 | 1.121297315 | 1.369160763 | 2.59E-05 |
| NDUFA4L2 | 1.185550792 | 1.096745581 | 1.281546699 | 1.83E-05 |
| PTPN12 | 1.1512472 | 1.071526474 | 1.236899084 | 0.000119669 |
| PYGL | 1.272356362 | 1.155663394 | 1.400832387 | 9.22E-07 |
| COL4A2 | 1.111269019 | 1.038918468 | 1.188658082 | 0.002129794 |
| FERMT2 | 1.12446124 | 1.034087896 | 1.222732695 | 0.006067857 |
| PFN1 | 1.068568941 | 1.006249467 | 1.134748011 | 0.030528229 |
| GAP43 | 1.098315101 | 1.022634739 | 1.179596209 | 0.010040985 |
| LOXL1 | 1.08507122 | 1.023351947 | 1.150512838 | 0.006285119 |
| LSM7 | 1.148128973 | 1.053575214 | 1.251168518 | 0.001631896 |
| CD163 | 1.087221766 | 1.027362858 | 1.150568331 | 0.003800529 |
| LY75 | 1.255724642 | 1.163230644 | 1.355573277 | 5.44E-09 |
| C5AR1 | 1.098152746 | 1.024140573 | 1.177513602 | 0.00853835 |

| ANGPT2 | 1.186750894 | 1.089625945 | 1.292533176 | 8.49E-05 |
| --- | --- | --- | --- | --- |
| FZD7 | 1.181718029 | 1.10596108 | 1.262664233 | 7.84E-07 |
| CLIC1 | 1.298874652 | 1.178522081 | 1.43151782 | 1.36E-07 |
| SLC2A10 | 1.295165489 | 1.192256466 | 1.406957055 | 9.19E-10 |
| COL4A1 | 1.122312831 | 1.046494594 | 1.203624078 | 0.001223163 |
| MGST1 | 1.067262104 | 1.014551068 | 1.122711743 | 0.011769734 |
| BHLHE40 | 1.14475185 | 1.055171878 | 1.241936812 | 0.001147113 |
| NUPR1 | 1.182244225 | 1.089414991 | 1.282983452 | 6.00E-05 |
| MEST | 1.207062952 | 1.107237419 | 1.315888484 | 1.93E-05 |
| ENPEP | 1.164108257 | 1.075211332 | 1.260355051 | 0.000177421 |
| MYOF | 1.113818011 | 1.022915819 | 1.212798295 | 0.0130807 |
| NAMPT | 1.158924069 | 1.080048998 | 1.243559319 | 4.11E-05 |
| MDK | 1.189588546 | 1.100120112 | 1.286333095 | 1.35E-05 |
| CSRP2 | 1.274885771 | 1.174999747 | 1.383263045 | 5.41E-09 |
| ALOX5AP | 1.17985394 | 1.103890248 | 1.261045037 | 1.11E-06 |
| PROS1 | 1.105016321 | 1.020028114 | 1.1970857 | 0.014460608 |
| CFI | 1.095500974 | 1.028847701 | 1.166472339 | 0.004400332 |
| C1QC | 1.067520461 | 1.000199962 | 1.139372104 | 0.049300913 |
| LAMB1 | 1.128360462 | 1.058632979 | 1.202680586 | 0.000206673 |
| LGALS1 | 1.270348482 | 1.165534435 | 1.384588234 | 5.14E-08 |
| COL5A2 | 1.174380699 | 1.102012947 | 1.251500747 | 7.29E-07 |
| C1S | 1.147977792 | 1.079611565 | 1.2206733 | 1.06E-05 |
| CLEC5A | 1.237753707 | 1.15269224 | 1.329092178 | 4.31E-09 |
| GEM | 1.117032843 | 1.037579825 | 1.202570002 | 0.003283144 |
| PCOLCE2 | 1.19674444 | 1.125319819 | 1.272702419 | 1.06E-08 |
| CA12 | 1.132871232 | 1.069714366 | 1.199756934 | 2.02E-05 |
| NMB | 1.081603375 | 1.003471819 | 1.165818351 | 0.040309711 |
| C1R | 1.182683214 | 1.10154864 | 1.269793756 | 3.71E-06 |
| LY96 | 1.096845584 | 1.020898624 | 1.178442411 | 0.011572509 |
| PKIB | 1.108270725 | 1.041051011 | 1.179830754 | 0.001281169 |
| CTSC | 1.145203451 | 1.058773934 | 1.238688357 | 0.000708057 |
| S100A8 | 1.129639704 | 1.045204615 | 1.220895739 | 0.002102077 |
| CAV1 | 1.100583152 | 1.030574295 | 1.175347844 | 0.004262343 |
| PLOD2 | 1.214059986 | 1.124420055 | 1.310846106 | 7.18E-07 |
| CKS2 | 1.092431972 | 1.013345347 | 1.17769092 | 0.021126222 |
| GPNMB | 1.145872039 | 1.072276096 | 1.224519258 | 5.81E-05 |
| S100A11 | 1.168938008 | 1.076019682 | 1.269880179 | 0.000220971 |
| SNAI2 | 1.106086745 | 1.042494541 | 1.173558077 | 0.000845347 |
| S100A4 | 1.124373786 | 1.050267796 | 1.20370863 | 0.000752122 |
| DIRAS3 | 1.221591684 | 1.141589995 | 1.307199826 | 6.96E-09 |
| SEC61G | 1.190131783 | 1.115498358 | 1.269758624 | 1.38E-07 |
| EN1 | 1.187312303 | 1.105677241 | 1.274974697 | 2.31E-06 |
| GBP1 | 1.110311317 | 1.042737373 | 1.182264349 | 0.001089815 |
| BCL2A1 | 1.144012018 | 1.070174171 | 1.222944388 | 7.74E-05 |
| LPL | 1.103609535 | 1.032854381 | 1.179211734 | 0.003543572 |
| SLC16A3 | 1.078842962 | 1.009608323 | 1.152825418 | 0.024926745 |
| SERPINE1 | 1.078086198 | 1.018801665 | 1.140820526 | 0.009175585 |
| PBK | 1.09003284 | 1.025477564 | 1.158651963 | 0.005645965 |
| LIPG | 1.126979059 | 1.053497715 | 1.205585719 | 0.000511024 |
| FABP5 | 1.195470318 | 1.129297141 | 1.265521031 | 7.99E-10 |

| MOXD1 | 1.114202648 | 1.057379716 | 1.174079209 | 5.14E-05 |
| --- | --- | --- | --- | --- |
| SLC10A4 | 1.100577897 | 1.041938954 | 1.162516962 | 0.000602184 |
| CXCL14 | 1.116850489 | 1.064041689 | 1.172280211 | 7.76E-06 |
| ADM | 1.178843302 | 1.109023629 | 1.253058541 | 1.28E-07 |
| FABP7 | 1.138502434 | 1.069247693 | 1.212242777 | 5.10E-05 |
| IGFBP2 | 1.147702711 | 1.090259713 | 1.208172235 | 1.45E-07 |
| CHI3L2 | 1.10372466 | 1.049367721 | 1.160897273 | 0.000128097 |

ession model

| **Supplementary Table 7. The score of other known signatures in patients with GBM.** | | | | | | |
| --- | --- | --- | --- | --- | --- | --- |
| ID | Cupcluster | geneCluster | Alcoholism | Angiogenesis | Antigen proce | Base excision |
| FB006 | A | A | 0.32367803 | 0.673475197 | 0.999572984 | 0.829193652 |
| FB013 | A | A | 0.403245321 | 0.33193367 | 0.998576614 | 0.749555192 |
| FB017 | A | A | 0.447227955 | 0.539107537 | 0.998220767 | 0.762436837 |
| FB108 | A | A | 0.39093303 | 0.723507224 | 0.998505444 | 0.841648281 |
| FB206 | A | A | 0.437762437 | 0.917728276 | 0.999217138 | 0.80869689 |
| FB402 | A | A | 0.327307665 | 0.524446659 | 0.998505444 | 0.862002704 |
| FB403 | A | A | 0.528147463 | 0.632054658 | 0.999074799 | 0.912817593 |
| FB404 | A | A | 0.510355135 | 0.51498114 | 0.999359476 | 0.865062985 |
| FB405 | A | A | 0.396911252 | 0.713970536 | 0.99900363 | 0.889758736 |
| FB406 | A | A | 0.561241193 | 0.612127251 | 0.999430646 | 0.845135578 |
| FB407 | A | A | 0.380186464 | 0.706924774 | 0.999359476 | 0.743221123 |
| FB408 | A | A | 0.631414134 | 0.468721087 | 0.999430646 | 0.850259768 |
| FB409 | A | A | 0.397195929 | 0.789623514 | 0.99900363 | 0.887765995 |
| FB410 | A | A | 0.356415913 | 0.663297986 | 0.999715323 | 0.730980001 |
| FB412 | A | A | 0.514127108 | 0.764144901 | 0.999430646 | 0.830688207 |
| FB414 | A | A | 0.391146538 | 0.838516832 | 0.999145968 | 0.829051313 |
| FB416 | A | A | 0.390434844 | 0.592128674 | 0.997864921 | 0.802149313 |
| FB417 | A | A | 0.475339833 | 0.853818234 | 0.999217138 | 0.851967831 |
| FB418 | A | A | 0.426517686 | 0.804782578 | 0.999145968 | 0.85004626 |
| FB419 | A | A | 0.387516903 | 0.475766849 | 0.999572984 | 0.810618461 |
| FB420 | A | A | 0.439256992 | 0.528930325 | 0.998861291 | 0.836666429 |
| FB425 | A | A | 0.372500178 | 0.731407017 | 0.999359476 | 0.850829122 |
| FB426 | A | A | 0.400612056 | 0.761511636 | 0.999217138 | 0.78485517 |
| FB427 | A | A | 0.359191517 | 0.782435414 | 0.999644153 | 0.89758736 |
| FB433 | A | A | 0.411002776 | 0.598818589 | 0.997793751 | 0.857376699 |
| FB434 | A | A | 0.353569141 | 0.62251797 | 0.999715323 | 0.750693901 |
| FB435 | A | A | 0.585082912 | 0.540744431 | 0.99793609 | 0.853391218 |
| FB436 | A | A | 0.28026475 | 0.689061277 | 0.998861291 | 0.838445662 |
| FB438 | A | A | 0.370436268 | 0.653334282 | 0.999074799 | 0.909472635 |
| FB439 | A | A | 0.385025977 | 0.634474415 | 0.998718952 | 0.835385382 |
| FB502 | A | A | 0.309871184 | 0.422389865 | 0.99793609 | 0.857874884 |
| FB503 | A | A | 0.419044908 | 0.385025977 | 0.998149598 | 0.824709985 |
| FB506 | A | A | 0.32054658 | 0.54501459 | 0.999430646 | 0.844708562 |
| FB508 | A | A | 0.246459327 | 0.562237563 | 0.999644153 | 0.845491424 |
| FB516 | A | A | 0.595331293 | 0.493203331 | 0.998718952 | 0.877232937 |
| FB553 | A | A | 0.410291082 | 0.497758167 | 0.998505444 | 0.935805281 |
| FB554 | A | A | 0.450430574 | 0.911607715 | 0.999217138 | 0.875240196 |
| FB555 | A | A | 0.517329727 | 0.6215216 | 0.999786492 | 0.876023059 |
| FB556 | A | A | 0.436481389 | 0.374777596 | 0.999501815 | 0.747064266 |
| FB557 | A | A | 0.424880791 | 0.702085261 | 0.999572984 | 0.866771048 |
| FB558 | A | A | 0.462600527 | 0.542523664 | 0.998078429 | 0.828410789 |
| FB559 | A | A | 0.626788129 | 0.500249093 | 0.999857661 | 0.912746424 |
| FB561 | A | A | 0.416126966 | 0.661162907 | 0.999572984 | 0.779873319 |
| FB564 | A | A | 0.561027685 | 0.610276849 | 0.999074799 | 0.952316561 |
| FB566 | A | A | 0.435556188 | 0.652480251 | 0.999572984 | 0.759234218 |
| FB567 | A | A | 0.297558893 | 0.595260124 | 0.999217138 | 0.827841435 |
| FB568 | A | A | 0.316916945 | 0.559960145 | 0.99893246 | 0.951249021 |
| FB569 | A | A | 0.399473347 | 0.593125044 | 0.999430646 | 0.803074514 |

| GSM405213 | B | B | 0.574122838 | 0.728631414 | 0.999928831 | 0.789196498 |
| --- | --- | --- | --- | --- | --- | --- |
| GSM405214 | B | C | 0.522453918 | 0.608355277 | 0.998434275 | 0.773681588 |
| GSM405215 | C | B | 0.521030532 | 0.621592769 | 0.999430646 | 0.757241477 |
| GSM405216 | B | A | 0.431997723 | 0.492705146 | 0.997793751 | 0.71973525 |
| GSM405217 | B | B | 0.622019785 | 0.473774109 | 0.999928831 | 0.82321543 |
| GSM405218 | B | C | 0.442174934 | 0.764429578 | 0.999572984 | 0.869902498 |
| GSM405219 | B | C | 0.579175859 | 0.518966622 | 0.999715323 | 0.801081774 |
| GSM405220 | C | C | 0.686570351 | 0.426588855 | 0.998291937 | 0.855739805 |
| GSM405221 | C | C | 0.531350082 | 0.563732119 | 0.999501815 | 0.7973098 |
| GSM405222 | B | B | 0.456764643 | 0.496975304 | 0.999644153 | 0.80556544 |
| GSM405223 | B | B | 0.589851256 | 0.38267739 | 0.999644153 | 0.866272863 |
| GSM405224 | B | B | 0.59148815 | 0.439185823 | 0.999928831 | 0.788911821 |
| GSM405228 | B | B | 0.607857092 | 0.633406875 | 0.999644153 | 0.812255355 |
| GSM405229 | B | B | 0.557326881 | 0.472421892 | 0.999359476 | 0.706213081 |
| GSM405230 | B | C | 0.506227315 | 0.449505373 | 0.999857661 | 0.842217636 |
| GSM405231 | C | B | 0.545797452 | 0.689844139 | 0.999644153 | 0.901928688 |
| GSM405232 | B | B | 0.920717387 | 0.699451996 | 0.997437905 | 0.977439328 |
| GSM405233 | B | C | 0.464593267 | 0.80770052 | 0.999786492 | 0.720375774 |
| GSM405234 | B | B | 0.461888834 | 0.597466373 | 0.996797381 | 0.803643869 |
| GSM405235 | B | C | 0.528859156 | 0.537897659 | 0.999644153 | 0.792541456 |
| GSM405236 | B | C | 0.547363177 | 0.581097431 | 0.999572984 | 0.809622091 |
| GSM405237 | B | C | 0.505515622 | 0.547363177 | 0.999430646 | 0.749412853 |
| GSM405238 | B | B | 0.480606363 | 0.488363818 | 0.999501815 | 0.695181838 |
| GSM405239 | C | C | 0.615116362 | 0.815244467 | 0.99900363 | 0.877731122 |
| GSM405240 | B | B | 0.556259341 | 0.430431998 | 0.99893246 | 0.78072735 |
| GSM405241 | C | C | 0.502384172 | 0.524944844 | 0.999857661 | 0.777809409 |
| GSM405242 | C | B | 0.564301473 | 0.543520034 | 0.999572984 | 0.866486371 |
| GSM405243 | B | B | 0.532204114 | 0.55640168 | 0.999786492 | 0.831969255 |
| GSM405244 | B | A | 0.604227457 | 0.559817807 | 0.998647783 | 0.616183902 |
| GSM405245 | B | B | 0.521457548 | 0.641021991 | 1 | 0.718810049 |
| GSM405246 | B | A | 0.747064266 | 0.57810832 | 0.995231656 | 0.894313572 |
| GSM405247 | C | B | 0.706782435 | 0.509216426 | 0.999359476 | 0.921642588 |
| GSM405248 | B | B | 0.698882642 | 0.703366308 | 0.999928831 | 0.766422319 |
| GSM405249 | B | B | 0.707992314 | 0.652053235 | 0.998647783 | 0.815529144 |
| GSM405251 | B | A | 0.554693616 | 0.605508505 | 0.998149598 | 0.788271297 |
| GSM405252 | C | C | 0.556544018 | 0.491922283 | 0.999644153 | 0.819301117 |
| GSM405253 | B | B | 0.541384955 | 0.570777881 | 0.999217138 | 0.911323038 |
| GSM405254 | B | C | 0.481033378 | 0.651626219 | 0.999644153 | 0.873247456 |
| GSM405255 | C | B | 0.598605081 | 0.787132588 | 0.999715323 | 0.738595118 |
| GSM405260 | C | C | 0.582876664 | 0.652195573 | 0.999430646 | 0.855383958 |
| GSM405262 | B | B | 0.52224041 | 0.497971675 | 0.99900363 | 0.797594477 |
| GSM405263 | B | C | 0.548928902 | 0.613764145 | 0.999501815 | 0.774037435 |
| GSM405264 | C | C | 0.583588357 | 0.73944915 | 0.999786492 | 0.762009821 |
| GSM405266 | B | B | 0.665646573 | 0.620738737 | 0.999786492 | 0.832182763 |
| GSM405267 | B | B | 0.454416056 | 0.546366807 | 0.999074799 | 0.876663583 |
| GSM405268 | B | B | 0.482599103 | 0.584655896 | 1 | 0.822219059 |
| GSM405269 | B | C | 0.514411786 | 0.819941641 | 0.999501815 | 0.786420895 |
| GSM405270 | B | C | 0.485161198 | 0.719450573 | 0.999786492 | 0.817735392 |
| GSM405271 | B | B | 0.401323749 | 0.511280336 | 0.999074799 | 0.72386307 |
| GSM405274 | B | C | 0.5201765 | 0.522098071 | 0.999644153 | 0.838374493 |

| GSM405275 | B | B | 0.580528076 | 0.309942353 | 0.998363106 | 0.716461462 |
| --- | --- | --- | --- | --- | --- | --- |
| GSM405276 | C | C | 0.439826347 | 0.729770123 | 0.999572984 | 0.737527578 |
| GSM405278 | B | B | 0.475411003 | 0.605152658 | 0.999786492 | 0.716461462 |
| GSM405280 | B | C | 0.509145257 | 0.528716817 | 0.999857661 | 0.838374493 |
| GSM405282 | C | C | 0.505800299 | 0.681802007 | 0.999644153 | 0.813963419 |
| GSM405290 | B | C | 0.556757526 | 0.522382749 | 0.999359476 | 0.847840011 |
| GSM405292 | B | B | 0.517472066 | 0.552629706 | 0.999786492 | 0.874599673 |
| GSM405293 | B | C | 0.542523664 | 0.732688065 | 0.999430646 | 0.822219059 |
| GSM405294 | B | B | 0.641235499 | 0.722012668 | 0.999359476 | 0.824994662 |
| GSM405296 | B | B | 0.599174436 | 0.444310014 | 0.998291937 | 0.814532773 |
| GSM405297 | B | B | 0.700661875 | 0.504732759 | 0.999644153 | 0.850686784 |
| GSM405299 | B | C | 0.624154864 | 0.607501245 | 0.998790122 | 0.762294499 |
| GSM405301 | C | C | 0.58942424 | 0.860508149 | 0.999501815 | 0.845562593 |
| GSM405302 | C | B | 0.504803929 | 0.795174721 | 0.999501815 | 0.81901644 |
| GSM405303 | B | B | 0.458899722 | 0.54907124 | 0.998220767 | 0.82314426 |
| GSM405304 | C | C | 0.574692193 | 0.692192726 | 0.999786492 | 0.73944915 |
| GSM405305 | C | C | 0.463525728 | 0.727421536 | 0.999644153 | 0.76734752 |
| GSM405307 | C | C | 0.475766849 | 0.54188314 | 0.999430646 | 0.814959789 |
| GSM405308 | C | B | 0.561668209 | 0.692406234 | 0.998718952 | 0.817806562 |
| GSM405309 | C | B | 0.511707352 | 0.559248452 | 0.999715323 | 0.846203117 |
| GSM405312 | C | B | 0.478328945 | 0.398263469 | 0.999359476 | 0.879866202 |
| GSM405313 | C | B | 0.4529215 | 0.610846203 | 0.999857661 | 0.688136076 |
| GSM405314 | C | C | 0.504875098 | 0.61120205 | 0.999288307 | 0.796526938 |
| GSM405315 | B | A | 0.286883496 | 0.583801865 | 0.997010889 | 0.71147961 |
| GSM405317 | C | C | 0.444310014 | 0.505302114 | 0.999857661 | 0.821863213 |
| GSM405320 | C | C | 0.656892748 | 0.534837378 | 0.999644153 | 0.746281403 |
| GSM405322 | C | C | 0.483310796 | 0.749982208 | 0.999501815 | 0.749911038 |
| GSM405323 | C | B | 0.571204896 | 0.657390933 | 0.999145968 | 0.787417266 |
| GSM405324 | C | C | 0.592484521 | 0.476834389 | 0.999857661 | 0.860721657 |
| GSM405326 | C | C | 0.575902071 | 0.689274785 | 0.999217138 | 0.854529927 |
| GSM405328 | C | C | 0.58942424 | 0.724788271 | 0.999359476 | 0.79218561 |
| GSM405330 | B | B | 0.674685076 | 0.71667497 | 0.999074799 | 0.953739947 |
| GSM405337 | B | B | 0.638673404 | 0.415628781 | 0.998434275 | 0.88107608 |
| GSM405340 | B | B | 0.553127891 | 0.420041278 | 0.999359476 | 0.703010462 |
| GSM405345 | C | C | 0.459397908 | 0.54295068 | 0.999715323 | 0.686712689 |
| GSM405349 | C | B | 0.597110526 | 0.305956871 | 0.999572984 | 0.889118212 |
| GSM405350 | C | A | 0.638459896 | 0.753683012 | 0.998861291 | 0.652053235 |
| GSM405351 | C | C | 0.514340616 | 0.628638531 | 0.999572984 | 0.857020853 |
| GSM405352 | B | B | 0.501316632 | 0.635470785 | 0.99793609 | 0.798875525 |
| GSM405353 | B | C | 0.564870828 | 0.792754964 | 0.999501815 | 0.819301117 |
| GSM405356 | B | B | 0.62977724 | 0.38054231 | 0.999786492 | 0.645007473 |
| GSM405362 | B | C | 0.543591203 | 0.779232795 | 0.99893246 | 0.784072308 |
| GSM405363 | B | B | 0.627428653 | 0.317984485 | 0.999145968 | 0.832325101 |
| GSM405365 | B | B | 0.566151875 | 0.558750267 | 0.999501815 | 0.746423742 |
| GSM405367 | C | B | 0.604654473 | 0.478044267 | 0.999715323 | 0.888762366 |
| GSM405368 | B | B | 0.725642303 | 0.517472066 | 0.999217138 | 0.794534197 |
| GSM405369 | C | B | 0.828980144 | 0.555832325 | 0.997793751 | 0.866486371 |
| GSM405370 | B | B | 0.756387446 | 0.70841933 | 0.998434275 | 0.838588001 |
| GSM405371 | B | C | 0.479111807 | 0.667781653 | 0.999857661 | 0.809906768 |
| GSM405372 | C | B | 0.624368372 | 0.544302897 | 0.999145968 | 0.891110953 |

| GSM405373 | C | C | 0.485090029 | 0.687922568 | 0.999644153 | 0.748843499 |
| --- | --- | --- | --- | --- | --- | --- |
| GSM405374 | C | C | 0.526724077 | 0.573838161 | 0.999430646 | 0.793110811 |
| GSM405375 | C | C | 0.555049463 | 0.497117643 | 0.99893246 | 0.853818234 |
| GSM405376 | B | B | 0.536616611 | 0.773895096 | 0.999074799 | 0.745854388 |
| GSM405379 | C | B | 0.440822717 | 0.653476621 | 0.999928831 | 0.86869262 |
| GSM405384 | C | C | 0.466301331 | 0.964486513 | 0.999644153 | 0.823642445 |
| GSM405385 | B | B | 0.63803288 | 0.440822717 | 0.999359476 | 0.8107608 |
| GSM405389 | C | B | 0.64635969 | 0.640950822 | 0.998647783 | 0.814461604 |
| GSM405391 | C | C | 0.47569568 | 0.751619102 | 0.999359476 | 0.805351932 |
| GSM405392 | B | C | 0.579745214 | 0.527222262 | 0.999217138 | 0.830332361 |
| GSM405393 | C | C | 0.478186606 | 0.500889616 | 0.999928831 | 0.761725144 |
| GSM405396 | C | C | 0.50779304 | 0.867198064 | 0.999644153 | 0.894313572 |
| GSM405397 | C | B | 0.535264394 | 0.748629991 | 0.999572984 | 0.769980784 |
| GSM405405 | C | C | 0.506654331 | 0.694541314 | 0.999288307 | 0.786420895 |
| GSM405412 | C | C | 0.715393922 | 0.620596399 | 0.999644153 | 0.820866842 |
| GSM405415 | C | C | 0.826702726 | 0.84079425 | 0.997082058 | 0.810903139 |
| GSM405416 | C | C | 0.676037293 | 0.564870828 | 0.999644153 | 0.707849975 |
| GSM405417 | C | C | 0.576471426 | 0.828766636 | 0.999644153 | 0.878442815 |
| GSM405418 | C | B | 0.794890043 | 0.581239769 | 0.999644153 | 0.8852039 |
| GSM405419 | C | C | 0.508148886 | 0.728631414 | 0.999715323 | 0.745356202 |
| GSM405422 | C | C | 0.542238987 | 0.871041207 | 0.999572984 | 0.691409864 |
| GSM405426 | C | C | 0.702085261 | 0.607572415 | 0.999501815 | 0.901857519 |
| GSM405427 | C | C | 0.62671696 | 0.612554267 | 0.999572984 | 0.829051313 |
| GSM405428 | C | C | 0.498968045 | 0.549284748 | 0.999501815 | 0.653405452 |
| GSM405430 | C | C | 0.510426304 | 0.947761725 | 0.999786492 | 0.701231229 |
| GSM405431 | C | B | 0.682371361 | 0.490783574 | 0.998861291 | 0.926766778 |
| GSM405432 | C | C | 0.59974379 | 0.460394278 | 0.999145968 | 0.774749128 |
| GSM405434 | C | C | 0.549711764 | 0.418689061 | 0.999572984 | 0.802932176 |
| GSM405436 | C | B | 0.622731478 | 0.504803929 | 0.999145968 | 0.836879937 |
| GSM405438 | C | C | 0.600597822 | 0.84798235 | 0.999644153 | 0.835314213 |
| GSM405440 | C | C | 0.475766849 | 0.747064266 | 0.999430646 | 0.756173938 |
| GSM405442 | C | C | 0.509287595 | 0.801935805 | 0.999857661 | 0.72187033 |
| GSM405443 | C | C | 0.548573055 | 0.586506298 | 0.998790122 | 0.648637108 |
| GSM405446 | C | C | 0.525870045 | 0.424596114 | 0.999074799 | 0.761013451 |
| GSM405447 | C | C | 0.593694399 | 0.614475838 | 0.999430646 | 0.818233578 |
| GSM405448 | C | C | 0.500391431 | 0.763433208 | 0.999288307 | 0.798590848 |
| GSM405452 | C | C | 0.530211373 | 0.412070315 | 0.999715323 | 0.797452139 |
| GSM405453 | C | C | 0.476122696 | 0.483595474 | 0.999715323 | 0.816169668 |
| GSM405454 | C | C | 0.605223827 | 0.650843356 | 0.999430646 | 0.768059213 |
| GSM405455 | C | C | 0.579033521 | 0.834673689 | 0.999857661 | 0.814034588 |
| GSM405458 | B | B | 0.594904277 | 0.881716604 | 0.999572984 | 0.762436837 |
| GSM405459 | C | C | 0.531634759 | 0.556899865 | 0.999217138 | 0.72080279 |
| GSM405461 | C | B | 0.429079781 | 0.614119991 | 0.999857661 | 0.862073874 |
| GSM405463 | C | C | 0.655967547 | 0.553199061 | 0.999715323 | 0.862216212 |
| GSM405464 | C | C | 0.451142267 | 0.587645007 | 0.999430646 | 0.799516049 |
| GSM405465 | C | C | 0.532844637 | 0.458472707 | 0.998718952 | 0.852466017 |
| GSM405466 | C | C | 0.553768415 | 0.605793182 | 0.999644153 | 0.82001281 |
| GSM405470 | C | B | 0.590562949 | 0.680805637 | 0.999715323 | 0.667496975 |
| GSM405471 | C | C | 0.6484236 | 0.502099495 | 0.999430646 | 0.744146324 |
| GSM405472 | C | C | 0.573197637 | 0.496690627 | 0.999644153 | 0.714112875 |

| GSM405473 | C | C | 0.527293431 | 0.765568287 | 0.999715323 | 0.762863853 |
| --- | --- | --- | --- | --- | --- | --- |
| GSM405474 | C | C | 0.566365383 | 0.680520959 | 0.999715323 | 0.793466657 |
| GSM405475 | B | B | 0.621023415 | 0.542238987 | 0.996085688 | 0.755533414 |
| GSM405477 | C | C | 0.572343605 | 0.651555049 | 0.999644153 | 0.762365668 |
| GSM405479 | C | C | 0.561027685 | 0.644153441 | 0.999857661 | 0.741086044 |
| GSM187153 | B | A | 0.475838019 | 0.251441179 | 0.99893246 | 0.836025906 |
| GSM187154 | B | A | 0.725286456 | 0.236068607 | 0.99686855 | 0.932531492 |
| GSM187155 | B | A | 0.611344388 | 0.280122411 | 0.99900363 | 0.941000641 |
| GSM187157 | B | A | 0.46331222 | 0.716105615 | 0.997010889 | 0.868123265 |
| GSM187158 | B | A | 0.533983346 | 0.501174294 | 0.997153228 | 0.889047043 |
| GSM187159 | A | A | 0.614902854 | 0.226389581 | 0.996299196 | 0.868052096 |
| GSM187160 | A | A | 0.698597965 | 0.122767063 | 0.989893958 | 0.932033307 |
| GSM187161 | A | A | 0.61732261 | 0.213579105 | 0.996441534 | 0.840651911 |
| GSM187162 | B | A | 0.662728631 | 0.365952601 | 0.999501815 | 0.900078286 |
| GSM187163 | A | A | 0.542025479 | 0.056935449 | 0.996583873 | 0.926055085 |
| GSM187164 | B | A | 0.601024838 | 0.122838232 | 0.998363106 | 0.884563376 |
| GSM187165 | B | A | 0.483168458 | 0.539890399 | 0.998007259 | 0.896804498 |
| GSM187166 | B | A | 0.577965981 | 0.260337343 | 0.997580243 | 0.889616397 |
| GSM187167 | B | A | 0.464023913 | 0.328232866 | 0.999786492 | 0.81389225 |
| GSM187168 | B | A | 0.617180272 | 0.761938652 | 0.997295566 | 0.901501672 |
| GSM187169 | B | A | 0.491851114 | 0.825777525 | 0.999074799 | 0.945626646 |
| GSM187171 | B | A | 0.479182976 | 0.605864351 | 0.999430646 | 0.901288165 |
| GSM187172 | A | A | 0.603159917 | 0.332574194 | 0.989111095 | 0.806846488 |
| GSM187174 | A | A | 0.510283966 | 0.497544659 | 0.997010889 | 0.864778308 |
| GSM187175 | B | A | 0.527862786 | 0.696035869 | 0.99893246 | 0.929328873 |
| GSM187176 | B | A | 0.537399473 | 0.500889616 | 0.999501815 | 0.927905487 |
| GSM187177 | B | A | 0.75389652 | 0.50259768 | 0.999644153 | 0.9255569 |
| GSM187178 | B | A | 0.447299125 | 0.394064479 | 0.998220767 | 0.854672265 |
| GSM187179 | B | A | 0.569710341 | 0.559533129 | 0.99893246 | 0.891537969 |
| GSM187181 | A | A | 0.651982065 | 0.190733756 | 0.997010889 | 0.903423244 |
| GSM187182 | B | A | 0.563874457 | 0.62052523 | 0.998576614 | 0.918938154 |
| GSM187183 | B | A | 0.477617251 | 0.27606576 | 0.999288307 | 0.791545086 |
| GSM187184 | A | A | 0.551846844 | 0.493416839 | 0.998007259 | 0.78079852 |
| GSM187185 | B | A | 0.712191303 | 0.59049178 | 0.998861291 | 0.936516974 |
| GSM187186 | B | A | 0.661661092 | 0.37335421 | 0.998434275 | 0.908832112 |
| GSM187187 | A | A | 0.676891324 | 0.515550495 | 0.995302825 | 0.843000498 |
| GSM187188 | B | A | 0.518326098 | 0.750266885 | 0.998291937 | 0.749555192 |
| GSM187189 | B | A | 0.672407658 | 0.126467867 | 0.999572984 | 0.936801651 |
| GSM187190 | B | A | 0.64628852 | 0.380399972 | 0.991815529 | 0.825919863 |
| GSM187191 | B | A | 0.551989182 | 0.768557398 | 0.996299196 | 0.790619885 |
| GSM187192 | B | A | 0.551918013 | 0.522311579 | 0.998078429 | 0.786847911 |
| GSM187193 | A | A | 0.552416198 | 0.415628781 | 0.995587503 | 0.868479112 |
| GSM187194 | B | A | 0.591274642 | 0.715322753 | 0.999572984 | 0.8780158 |
| GSM187195 | A | A | 0.543875881 | 0.149882571 | 0.994021778 | 0.720375774 |
| GSM187196 | B | A | 0.551775674 | 0.189808555 | 0.997082058 | 0.906697032 |
| GSM187197 | B | A | 0.495765426 | 0.191872465 | 0.999501815 | 0.817735392 |
| GSM187198 | B | A | 0.482385595 | 0.33506512 | 0.997082058 | 0.871610562 |
| GSM187199 | A | A | 0.576257918 | 0.35570422 | 0.998505444 | 0.846416625 |
| GSM187200 | A | A | 0.551989182 | 0.593694399 | 0.997010889 | 0.763931393 |
| GSM187201 | A | A | 0.653191944 | 0.459326738 | 0.998647783 | 0.785068678 |

| GSM187202 | B | A | 0.648779446 | 0.547932531 | 0.999145968 | 0.922069604 |
| --- | --- | --- | --- | --- | --- | --- |
| GSM187203 | B | A | 0.566863568 | 0.434132802 | 0.997010889 | 0.801224112 |
| GSM187204 | B | A | 0.557611558 | 0.401394919 | 0.997651413 | 0.914454487 |
| GSM187205 | B | A | 0.455198918 | 0.312931464 | 0.998434275 | 0.952601238 |
| GSM187206 | A | A | 0.626147605 | 0.548146039 | 0.997722582 | 0.839655541 |
| GSM187207 | C | A | 0.520247669 | 0.551989182 | 0.999145968 | 0.825421678 |
| GSM187208 | B | A | 0.513344246 | 0.590207103 | 0.997010889 | 0.851754324 |
| GSM187209 | B | A | 0.491424098 | 0.686214504 | 0.99893246 | 0.877731122 |
| GSM187210 | B | A | 0.525870045 | 0.408156003 | 0.999786492 | 0.891110953 |
| GSM187211 | B | A | 0.617037933 | 0.361041919 | 0.99900363 | 0.945982492 |
| GSM187212 | A | A | 0.571062558 | 0.243399046 | 0.99900363 | 0.856095652 |
| GSM187213 | B | A | 0.61319479 | 0.343676607 | 0.99686855 | 0.876734752 |
| GSM187214 | A | A | 0.639740944 | 0.520247669 | 0.992384884 | 0.791758594 |
| GSM187215 | A | A | 0.65767561 | 0.299693972 | 0.999288307 | 0.880720233 |
| GSM187216 | B | A | 0.676393139 | 0.310938723 | 0.999145968 | 0.846630133 |
| GSM187217 | B | A | 0.554195431 | 0.496904135 | 0.999145968 | 0.897089175 |
| GSM187218 | A | A | 0.79005053 | 0.208170237 | 0.995587503 | 0.866201694 |
| GSM187219 | B | A | 0.593267383 | 0.335990321 | 0.998434275 | 0.769055583 |
| GSM187220 | B | A | 0.49021422 | 0.432567077 | 0.996441534 | 0.855668636 |
| GSM187221 | A | A | 0.584015373 | 0.583801865 | 0.999359476 | 0.869190805 |
| GSM187222 | B | A | 0.498114013 | 0.577610135 | 0.997153228 | 0.854031742 |
| GSM187223 | B | A | 0.53981923 | 0.661447584 | 0.996797381 | 0.899793609 |
| GSM187224 | A | A | 0.614618177 | 0.22539321 | 0.994448794 | 0.836879937 |
| GSM187225 | C | A | 0.638531065 | 0.31435485 | 0.997153228 | 0.869119636 |
| GSM187226 | B | A | 0.609280478 | 0.534410362 | 0.998363106 | 0.801010604 |
| GSM187227 | B | A | 0.556472849 | 0.499679738 | 0.999786492 | 0.832752117 |
| GSM187228 | A | A | 0.546295637 | 0.193651697 | 0.9928119 | 0.86250089 |
| GSM187229 | B | A | 0.596398833 | 0.635613124 | 0.997366735 | 0.874599673 |
| GSM187230 | B | A | 0.663440325 | 0.362038289 | 0.999501815 | 0.845847271 |
| GSM187231 | B | A | 0.531278913 | 0.729627784 | 0.999430646 | 0.923137143 |
| GSM187232 | B | A | 0.545085759 | 0.156572486 | 0.996014519 | 0.834389011 |
| GSM187233 | B | A | 0.495196071 | 0.585438759 | 0.998861291 | 0.932673831 |
| GSM187234 | B | A | 0.547363177 | 0.725499964 | 0.997793751 | 0.880435556 |
| GSM187235 | B | A | 0.535762579 | 0.386662871 | 0.999145968 | 0.885986763 |
| GSM187236 | B | A | 0.459397908 | 0.35164757 | 0.99793609 | 0.916304889 |
| 900-00-53-32 | B | C | 0.467653548 | 0.740089673 | 0.998434275 | 0.859084763 |
| 900-00-5317 | B | B | 0.402177781 | 0.391360046 | 0.992029037 | 0.731762864 |
| 900-00-5338 | C | C | 0.536189595 | 0.878941001 | 0.998007259 | 0.7300548 |
| 900-00-5342 | C | C | 0.518041421 | 0.738168102 | 0.999572984 | 0.82108035 |
| 900-00-5346 | C | C | 0.478115437 | 0.689417123 | 0.999572984 | 0.769055583 |
| 900-00-5379 | C | C | 0.408796527 | 0.631271796 | 0.999217138 | 0.847128318 |
| 900-00-5381 | B | C | 0.551562166 | 0.565582521 | 0.99900363 | 0.727279197 |
| 900-00-5384 | B | C | 0.6152587 | 0.656750409 | 0.998790122 | 0.732972742 |
| 900-00-5396 | C | C | 0.477901929 | 0.726496335 | 0.998861291 | 0.704433848 |
| 900-00-5404 | B | C | 0.475411003 | 0.437050744 | 0.999359476 | 0.765995303 |
| 900-00-5404- | B | C | 0.467724717 | 0.574905701 | 0.999288307 | 0.697672764 |
| 900-00-5413 | C | C | 0.565582521 | 0.697459256 | 0.999217138 | 0.784143477 |
| 900-00-5414 | B | B | 0.491993452 | 0.318197993 | 0.998505444 | 0.684150594 |
| 900-00-5445 | C | B | 0.456693474 | 0.334709273 | 0.999359476 | 0.726709843 |
| 900-00-5458 | B | B | 0.583090172 | 0.360330226 | 0.99900363 | 0.755462245 |

| 900-00-5462 | C | C | 0.496761796 | 0.520603516 | 0.999074799 | 0.79218561 |
| --- | --- | --- | --- | --- | --- | --- |
| 900-00-5488 | C | C | 0.495836595 | 0.678528219 | 0.999145968 | 0.819158779 |
| 900-00-5489 | C | C | 0.37335421 | 0.621735108 | 0.999288307 | 0.884848054 |
| 900-00-5540 | B | C | 0.576329087 | 0.616041563 | 0.99900363 | 0.899437762 |
| 900-00-5541 | C | C | 0.531990606 | 0.403458829 | 0.998647783 | 0.912319408 |
| 900-00-5542 | C | C | 0.493772685 | 0.345171162 | 0.999288307 | 0.820368657 |
| 900-00-5543 | B | C | 0.539178706 | 0.17265675 | 0.999644153 | 0.76421607 |
| 900-00-5544 | C | C | 0.444950537 | 0.680663298 | 0.999288307 | 0.876023059 |
| 900-00-5546 | C | C | 0.550067611 | 0.885488577 | 0.998861291 | 0.902071027 |
| 900-00-5548 | C | C | 0.466158992 | 0.707921144 | 0.99900363 | 0.830118853 |
| 900-00-5551 | C | C | 0.461888834 | 0.702868123 | 0.999288307 | 0.791260409 |
| 900-00-5554 | C | B | 0.583944203 | 0.43121486 | 0.99893246 | 0.903280905 |
| 900-0052-99 | C | C | 0.555618817 | 0.471283183 | 0.99900363 | 0.824852324 |
| 900-0053-03 | C | B | 0.806704149 | 0.399117501 | 0.999074799 | 0.913671625 |
| E09139 | B | C | 0.542167817 | 0.487652124 | 0.999288307 | 0.772187033 |
| E09192 | C | B | 0.612269589 | 0.535904918 | 0.999430646 | 0.719165896 |
| E09278 | C | B | 0.629990748 | 0.511280336 | 0.996797381 | 0.840296064 |
| E09331 | B | B | 0.509572272 | 0.554480108 | 0.998291937 | 0.831044054 |
| E09348 | B | B | 0.547292008 | 0.29990748 | 0.999288307 | 0.624510711 |
| E09430 | B | C | 0.565013166 | 0.368016511 | 0.999644153 | 0.686285674 |
| E09451 | B | C | 0.76934026 | 0.608212939 | 0.999430646 | 0.91936517 |
| E09454 | B | B | 0.408369511 | 0.517970251 | 0.997082058 | 0.631556473 |
| E09483 | B | C | 0.501814817 | 0.256921216 | 0.998861291 | 0.674542737 |
| E09489 | C | C | 0.488506156 | 0.851398477 | 0.999501815 | 0.698455626 |
| E09535 | B | C | 0.490997082 | 0.453206177 | 0.999359476 | 0.780086827 |
| E09569 | B | B | 0.590349441 | 0.321258273 | 0.998790122 | 0.763717885 |
| E09601 | C | C | 0.602448224 | 0.659383674 | 0.999288307 | 0.736033023 |
| E09602 | B | C | 0.555832325 | 0.33300121 | 0.999644153 | 0.693046758 |
| E09605 | B | C | 0.374777596 | 0.430004982 | 0.999715323 | 0.755106398 |
| E09606 | C | C | 0.479752331 | 0.336986691 | 0.999572984 | 0.725072949 |
| E09610 | B | C | 0.52537186 | 0.615756886 | 0.999786492 | 0.78905416 |
| E09615 | B | C | 0.582947833 | 0.212298057 | 0.999288307 | 0.864991816 |
| E09623 | B | C | 0.51604868 | 0.378336061 | 0.999217138 | 0.787559604 |
| E09647 | B | C | 0.436766066 | 0.420824141 | 0.999430646 | 0.820866842 |
| E09649 | B | C | 0.413849548 | 0.540175077 | 0.999430646 | 0.682015515 |
| E09654 | B | C | 0.368087681 | 0.549925272 | 0.999288307 | 0.676108462 |
| E09670 | B | B | 0.374208241 | 0.380257633 | 0.998363106 | 0.700448367 |
| E09690 | C | B | 0.722083837 | 0.585225251 | 0.997722582 | 0.81382108 |
| E09698 | C | C | 0.511707352 | 0.658458473 | 0.99893246 | 0.77254288 |
| E09722 | B | B | 0.589210732 | 0.24510711 | 0.998078429 | 0.892747847 |
| E09730 | B | C | 0.626503452 | 0.463454558 | 0.99900363 | 0.776030176 |
| E09740 | C | C | 0.561170023 | 0.50779304 | 0.999359476 | 0.714824568 |
| E09744 | C | C | 0.581097431 | 0.240481105 | 0.998861291 | 0.854316419 |
| E09759 | B | C | 0.553412569 | 0.511137997 | 0.999572984 | 0.699523166 |
| E09782 | C | C | 0.423528574 | 0.780229165 | 0.999074799 | 0.835243043 |
| E09786 | B | B | 0.512561384 | 0.292577041 | 0.999217138 | 0.701587076 |
| E09787 | B | B | 0.429933813 | 0.501814817 | 0.999359476 | 0.743932816 |
| E09791 | C | C | 0.58216497 | 0.276279268 | 0.999217138 | 0.695253007 |
| E09800 | C | C | 0.569639172 | 0.845277916 | 0.999430646 | 0.75802434 |
| E09802 | B | C | 0.594761939 | 0.331506654 | 0.999217138 | 0.826133371 |

| E09810 | C | C | 0.533271653 | 0.566080706 | 0.998861291 | 0.815529144 |
| --- | --- | --- | --- | --- | --- | --- |
| E09832 | B | C | 0.468934595 | 0.349868337 | 0.999359476 | 0.697174578 |
| E09833 | C | C | 0.531777098 | 0.449790051 | 0.999145968 | 0.736815885 |
| E09846 | B | A | 0.523094442 | 0.203188385 | 0.998291937 | 0.635541954 |
| E09847 | B | B | 0.522453918 | 0.332147178 | 0.997864921 | 0.633051028 |
| E09852 | B | A | 0.474912818 | 0.246530496 | 0.987403032 | 0.606362536 |
| E09907 | C | C | 0.515692833 | 0.258415771 | 0.999288307 | 0.80243399 |
| E09917 | B | C | 0.62664579 | 0.678670557 | 0.997437905 | 0.841150096 |
| E09930 | B | B | 0.406946125 | 0.761226959 | 0.998861291 | 0.671482457 |
| E09938 | B | C | 0.552914383 | 0.342822575 | 0.998861291 | 0.878727493 |
| E09951 | B | C | 0.536901288 | 0.826631556 | 0.999145968 | 0.858800085 |
| E09956 | B | B | 0.440822717 | 0.298484094 | 0.997153228 | 0.739520319 |
| E09967 | C | C | 0.479040638 | 0.567432923 | 0.999501815 | 0.832965625 |
| E10002 | C | C | 0.460607786 | 0.717457832 | 0.999572984 | 0.801722297 |
| E10013 | C | C | 0.469290442 | 0.393139278 | 0.99900363 | 0.872322255 |
| E10016 | B | C | 0.517756743 | 0.751263255 | 0.998861291 | 0.722724361 |
| E10026 | C | C | 0.530851897 | 0.526795246 | 0.999572984 | 0.698882642 |
| E10031 | B | C | 0.614760515 | 0.568713971 | 0.99893246 | 0.813251726 |
| E10041 | C | C | 0.489075511 | 0.386307024 | 0.999572984 | 0.796811615 |
| E10077 | B | C | 0.510639812 | 0.264251655 | 0.999217138 | 0.677745356 |
| E10102 | C | C | 0.543591203 | 0.358906839 | 0.99900363 | 0.695822361 |
| E10110 | B | B | 0.683011885 | 0.234502882 | 0.996299196 | 0.896804498 |
| E10144 | B | B | 0.443455982 | 0.465020283 | 0.99893246 | 0.736887054 |
| E10158 | B | B | 0.595758309 | 0.465304961 | 0.999715323 | 0.775531991 |
| E10184 | B | B | 0.405522739 | 0.523023272 | 0.998149598 | 0.701800584 |
| E10193 | B | B | 0.435485019 | 0.524375489 | 0.999430646 | 0.687922568 |
| E10211 | B | B | 0.408725358 | 0.395345527 | 0.998220767 | 0.707280621 |
| E10226 | B | B | 0.637677034 | 0.623514341 | 0.996441534 | 0.847128318 |
| E10227 | C | C | 0.522667426 | 0.771973525 | 0.999217138 | 0.806561811 |
| E10250 | C | C | 0.519535976 | 0.401750765 | 0.999644153 | 0.817877731 |
| E10252 | B | B | 0.431713045 | 0.255924845 | 0.997437905 | 0.714255213 |
| E10258 | B | B | 0.422461035 | 0.375773966 | 0.999145968 | 0.743434631 |
| E10262 | C | B | 0.564870828 | 0.459611416 | 0.999430646 | 0.922069604 |
| E10267 | B | C | 0.544445235 | 0.867767419 | 0.997793751 | 0.856878514 |
| E10271 | C | C | 0.51184969 | 0.216425877 | 0.999359476 | 0.868906128 |
| E10284 | C | C | 0.445591061 | 0.601309515 | 0.998861291 | 0.765141271 |
| E10290 | B | C | 0.594904277 | 0.759163049 | 0.997580243 | 0.752686642 |
| E10292 | C | C | 0.489787204 | 0.321115935 | 0.999288307 | 0.906483524 |
| E10300 | C | C | 0.60707423 | 0.652195573 | 0.997793751 | 0.783289446 |
| E10305 | C | C | 0.672407658 | 0.45398904 | 0.999074799 | 0.704718525 |
| E10306 | C | C | 0.48708277 | 0.455483595 | 0.998576614 | 0.869333144 |
| E10312 | B | B | 0.426802363 | 0.484093659 | 0.999572984 | 0.766706996 |
| E10313 | C | C | 0.426802363 | 0.551775674 | 0.998291937 | 0.843000498 |
| E10318 | C | C | 0.563162764 | 0.460323109 | 0.999644153 | 0.769269091 |
| E10433 | B | B | 0.452850331 | 0.633478044 | 0.999572984 | 0.752259626 |
| E10444 | C | C | 0.502526511 | 0.621450431 | 0.999074799 | 0.857732546 |
| E10462 | B | C | 0.4529215 | 0.772187033 | 0.999572984 | 0.687282044 |
| E10488 | B | C | 0.658173795 | 0.796811615 | 0.999501815 | 0.852679525 |
| E10514 | B | B | 0.614902854 | 0.260195004 | 0.994377624 | 0.819656964 |
| E10551 | B | C | 0.647996584 | 0.532417622 | 0.996441534 | 0.884634546 |

| E50057 | B | B | 0.54807487 | 0.437193082 | 0.998718952 | 0.649277631 |
| --- | --- | --- | --- | --- | --- | --- |
| E50074 | B | C | 0.580741584 | 0.146964629 | 0.998861291 | 0.682229023 |
| E50091 | B | B | 0.568927478 | 0.221834745 | 0.999145968 | 0.90385026 |
| E50123 | C | C | 0.515265817 | 0.367660665 | 0.998861291 | 0.889687567 |
| HF0024 | B | C | 0.518539606 | 0.47882713 | 0.999217138 | 0.835670059 |
| HF0031 | C | C | 0.603586933 | 0.71667497 | 0.999359476 | 0.760230589 |
| HF0048 | B | B | 0.544516405 | 0.434417479 | 0.998861291 | 0.5139136 |
| HF0050 | B | C | 0.468649918 | 0.649846986 | 0.999430646 | 0.684079425 |
| HF0066 | B | C | 0.520959362 | 0.827058572 | 0.999074799 | 0.882285958 |
| HF0138 | C | C | 0.483381966 | 0.477190236 | 0.998790122 | 0.862714398 |
| HF0142 | C | C | 0.535904918 | 0.848480535 | 0.99893246 | 0.888691196 |
| HF0180 | C | C | 0.466087823 | 0.343747776 | 0.999288307 | 0.916731905 |
| HF0268 | B | B | 0.523521458 | 0.383033236 | 0.998718952 | 0.671268949 |
| HF0300.3 | C | C | 0.508504733 | 0.52323678 | 0.999430646 | 0.770265462 |
| HF0408 | C | C | 0.523948473 | 0.478115437 | 0.998861291 | 0.736531208 |
| HF0442.5 | B | B | 0.57191659 | 0.459753754 | 0.999217138 | 0.821009181 |
| HF0445 | C | C | 0.519749484 | 0.325528432 | 0.998505444 | 0.843000498 |
| HF0505 | B | A | 0.40018504 | 0.178350295 | 0.992883069 | 0.702868123 |
| HF0520 | C | C | 0.415201765 | 0.327521173 | 0.999217138 | 0.770763647 |
| HF0543 | C | C | 0.487865632 | 0.783004768 | 0.999359476 | 0.873318625 |
| HF0583 | C | C | 0.572201267 | 0.166251512 | 0.999430646 | 0.73112234 |
| HF0627 | B | C | 0.601096007 | 0.831969255 | 0.998718952 | 0.915735535 |
| HF0654 | B | B | 0.518824283 | 0.334922781 | 0.998861291 | 0.783645292 |
| HF0790 | C | C | 0.51918013 | 0.270656893 | 0.999217138 | 0.740160843 |
| HF0891 | C | C | 0.486869262 | 0.493843855 | 0.997651413 | 0.891680307 |
| HF0894 | C | C | 0.428723934 | 0.904704292 | 0.999217138 | 0.823001922 |
| HF0963 | B | C | 0.512490214 | 0.315707067 | 0.999145968 | 0.85524162 |
| HF0986 | B | B | 0.533698669 | 0.300120988 | 0.99380827 | 0.766706996 |
| HF0990 | B | B | 0.53462387 | 0.351220554 | 0.999145968 | 0.848694043 |
| HF0992 | B | B | 0.678101203 | 0.42708704 | 0.998576614 | 0.878656323 |
| HF0996 | B | B | 0.611415558 | 0.268948829 | 0.99893246 | 0.87694826 |
| HF1058 | B | A | 0.574478685 | 0.218845634 | 0.992456053 | 0.724076578 |
| HF1077 | B | C | 0.482812611 | 0.548359547 | 0.999074799 | 0.788840652 |
| HF1078 | B | C | 0.618318981 | 0.305814533 | 0.998790122 | 0.919080492 |
| HF1097 | B | C | 0.61632624 | 0.939150238 | 0.999430646 | 0.594690769 |
| HF1122 | C | C | 0.508718241 | 0.320688919 | 0.998576614 | 0.862785567 |
| HF1137 | C | C | 0.503949897 | 0.461532987 | 0.999074799 | 0.828197281 |
| HF1139 | B | C | 0.448722511 | 0.622090954 | 0.998718952 | 0.799516049 |
| HF1178 | B | C | 0.475909188 | 0.513771262 | 0.997864921 | 0.829051313 |
| HF1191 | C | C | 0.385951178 | 0.742367091 | 0.999217138 | 0.829122482 |
| HF1220 | C | C | 0.465304961 | 0.576756103 | 0.998718952 | 0.762223329 |
| HF1262 | C | C | 0.4860864 | 0.760017081 | 0.998790122 | 0.756672123 |
| HF1269 | C | C | 0.492064622 | 0.441819088 | 0.99893246 | 0.808981567 |
| HF1292 | B | C | 0.498042844 | 0.804782578 | 0.999430646 | 0.784997509 |
| HF1297 | B | C | 0.45085759 | 0.598391574 | 0.999786492 | 0.842573482 |
| HF1318 | C | C | 0.569639172 | 0.57390933 | 0.999359476 | 0.846630133 |
| HF1338 | B | B | 0.663369155 | 0.2905843 | 0.99900363 | 0.782221906 |
| HF1356 | B | C | 0.582520817 | 0.668351007 | 0.999288307 | 0.740943705 |
| HF1382 | C | C | 0.392356416 | 0.705216711 | 0.99900363 | 0.891253292 |
| HF1397 | C | C | 0.550992812 | 0.180343036 | 0.999074799 | 0.846558964 |

| HF1469 | B | B | 0.433990463 | 0.310369369 | 0.998078429 | 0.774962636 |
| --- | --- | --- | --- | --- | --- | --- |
| HF1492 | B | B | 0.453206177 | 0.420397125 | 0.994733471 | 0.717315494 |
| HF1509 | C | C | 0.44260195 | 0.470784998 | 0.999217138 | 0.65461533 |
| HF1517 | C | C | 0.434132802 | 0.917372429 | 0.999572984 | 0.861860366 |
| HF1534 | B | B | 0.654686499 | 0.205323465 | 0.997509074 | 0.724717102 |
| HF1538 | B | C | 0.548786563 | 0.277702655 | 0.998790122 | 0.781510213 |
| HF1540 | C | C | 0.493559177 | 0.750978578 | 0.998790122 | 0.806277133 |
| HF1585 | B | C | 0.54601096 | 0.293288734 | 0.998220767 | 0.862216212 |
| HF1589 | C | C | 0.515408156 | 0.500320262 | 0.998790122 | 0.801722297 |
| HF1608 | C | C | 0.531777098 | 0.526225891 | 0.999359476 | 0.922211942 |
| HF1618 | C | C | 0.425948331 | 0.687922568 | 0.999145968 | 0.800868266 |
| HF1628 | C | C | 0.484663013 | 0.730695324 | 0.999644153 | 0.896377482 |
| HF1640 | B | A | 0.37748203 | 0.235499253 | 0.991459683 | 0.716105615 |
| HF1667 | B | B | 0.495978934 | 0.249519607 | 0.993452423 | 0.75489289 |
| HF1671 | B | B | 0.566934738 | 0.382890897 | 0.996797381 | 0.812326525 |
| MD545226 | C | B | 0.6484236 | 0.491281759 | 0.997651413 | 0.8107608 |
| MD602958 | C | C | 0.524944844 | 0.471212013 | 0.999288307 | 0.765639456 |
| MD607103 | C | C | 0.554907124 | 0.541954309 | 0.998647783 | 0.814319266 |
| MD607216 | C | C | 0.419471924 | 0.365952601 | 0.999145968 | 0.847057149 |
| MD608660 | B | C | 0.434915664 | 0.410718098 | 0.997651413 | 0.787061419 |
| MD621233 | B | A | 0.850473276 | 0.145683581 | 0.997082058 | 0.642872393 |

| CD8 T effecto | Cell cycle | Cytokine-cyto | DNA damage | DNA replicati | EMT1 | EMT2 |
| --- | --- | --- | --- | --- | --- | --- |
| 0.365312077 | 0.88933172 | 0.861575689 | 0.768557398 | 0.482599103 | 0.201409152 | 0.738168102 |
| 0.417692691 | 0.873247456 | 0.806205964 | 0.754608213 | 0.5336275 | 0.231513771 | 0.781083197 |
| 0.424880791 | 0.850615615 | 0.862145043 | 0.771190663 | 0.520959362 | 0.252793395 | 0.780015657 |
| 0.479111807 | 0.931890969 | 0.860223472 | 0.790264038 | 0.548288378 | 0.252579888 | 0.75909188 |
| 0.785495694 | 0.894954096 | 0.940644794 | 0.636823002 | 0.4932745 | 0.141342253 | 0.826346879 |
| 0.416838659 | 0.937655683 | 0.910611344 | 0.758878372 | 0.483026119 | 0.223969824 | 0.773254573 |
| 0.730908832 | 0.895950466 | 0.925201053 | 0.777026546 | 0.519678315 | 0.2161412 | 0.785638033 |
| 0.766137641 | 0.809550922 | 0.882712974 | 0.845135578 | 0.555049463 | 0.191018433 | 0.761511636 |
| 0.40232012 | 0.914240979 | 0.861362181 | 0.788057789 | 0.529215002 | 0.228311152 | 0.857803715 |
| 0.331648993 | 0.836025906 | 0.876663583 | 0.74969753 | 0.563518611 | 0.18511138 | 0.674115721 |
| 0.483239627 | 0.89758736 | 0.891964985 | 0.677104832 | 0.383104405 | 0.224539179 | 0.825919863 |
| 0.513628923 | 0.931677461 | 0.902426874 | 0.677745356 | 0.511137997 | 0.281047612 | 0.850829122 |
| 0.424667283 | 0.896804498 | 0.936303466 | 0.791260409 | 0.515479325 | 0.227243613 | 0.816312006 |
| 0.449291865 | 0.89039926 | 0.914739164 | 0.731051171 | 0.472493061 | 0.195502099 | 0.776172514 |
| 0.49220696 | 0.888904704 | 0.897373852 | 0.765995303 | 0.506440823 | 0.19336702 | 0.749270515 |
| 0.425663654 | 0.901359334 | 0.892320831 | 0.737527578 | 0.48089104 | 0.22439684 | 0.824496477 |
| 0.487011601 | 0.862714398 | 0.83865917 | 0.802861006 | 0.47569568 | 0.17272792 | 0.791545086 |
| 0.414988257 | 0.86250089 | 0.841861789 | 0.788698313 | 0.574834531 | 0.2026902 | 0.754608213 |
| 0.380613479 | 0.923635328 | 0.879510355 | 0.7838588 | 0.640594975 | 0.195288592 | 0.617180272 |
| 0.424667283 | 0.868763789 | 0.908618604 | 0.753967689 | 0.620382891 | 0.171802719 | 0.651056864 |
| 0.463525728 | 0.900789979 | 0.89659099 | 0.749057007 | 0.491779944 | 0.168101914 | 0.727208028 |
| 0.551135151 | 0.910397836 | 0.893744217 | 0.783147107 | 0.522453918 | 0.169240623 | 0.757526155 |
| 0.629848409 | 0.905344815 | 0.893317202 | 0.732261049 | 0.471496691 | 0.199131734 | 0.794747705 |
| 0.443384812 | 0.894882926 | 0.883211159 | 0.780229165 | 0.549355918 | 0.193082343 | 0.722937869 |
| 0.571347235 | 0.89139563 | 0.832396271 | 0.818589424 | 0.49427087 | 0.222048253 | 0.819158779 |
| 0.430574336 | 0.930182905 | 0.864280122 | 0.73944915 | 0.474129955 | 0.214931322 | 0.868194435 |
| 0.488292648 | 0.930823429 | 0.718952388 | 0.857946054 | 0.440965056 | 0.260479681 | 0.822076721 |
| 0.425948331 | 0.93381254 | 0.886769625 | 0.757597324 | 0.497544659 | 0.192868835 | 0.802789837 |
| 0.39292577 | 0.946480678 | 0.866130525 | 0.761013451 | 0.562735748 | 0.188954523 | 0.772115864 |
| 0.526724077 | 0.935449434 | 0.708490499 | 0.803216853 | 0.515621664 | 0.223685147 | 0.815173297 |
| 0.418902569 | 0.963561312 | 0.835314213 | 0.827770265 | 0.535264394 | 0.234929898 | 0.770194292 |
| 0.377553199 | 0.88826418 | 0.893317202 | 0.72592698 | 0.774464451 | 0.175645862 | 0.642658885 |
| 0.398832823 | 0.922923635 | 0.880791403 | 0.734823144 | 0.531421251 | 0.211444025 | 0.735178991 |
| 0.398192299 | 0.878300477 | 0.892961355 | 0.751476763 | 0.519535976 | 0.208739591 | 0.740730197 |
| 0.367589495 | 0.936730482 | 0.848622874 | 0.718098356 | 0.531848267 | 0.209308946 | 0.788271297 |
| 0.403957014 | 0.904419614 | 0.776528361 | 0.873603302 | 0.530994235 | 0.277489147 | 0.750124546 |
| 0.439684008 | 0.946551847 | 0.71354352 | 0.762436837 | 0.569212156 | 0.23265248 | 0.766493488 |
| 0.642730055 | 0.861931535 | 0.844495054 | 0.79837734 | 0.579033521 | 0.227385951 | 0.767916874 |
| 0.426731194 | 0.916518397 | 0.878798662 | 0.726140488 | 0.526154722 | 0.17891965 | 0.755746922 |
| 0.431499537 | 0.898227884 | 0.90278272 | 0.752900149 | 0.552558537 | 0.211942211 | 0.775104975 |
| 0.433705786 | 0.844210376 | 0.819087609 | 0.829264821 | 0.602732902 | 0.232865988 | 0.772756387 |
| 0.471923706 | 0.83552772 | 0.822503736 | 0.727421536 | 0.647427229 | 0.493772685 | 0.801010604 |
| 0.594477261 | 0.926482101 | 0.889118212 | 0.698099779 | 0.500106754 | 0.201907338 | 0.837022276 |
| 0.349156644 | 0.944772614 | 0.74564088 | 0.844637392 | 0.819372287 | 0.198064195 | 0.764998932 |
| 0.412070315 | 0.934595402 | 0.817521885 | 0.693117928 | 0.633051028 | 0.20368657 | 0.829691837 |
| 0.530211373 | 0.904419614 | 0.833748488 | 0.761369298 | 0.510426304 | 0.259127464 | 0.847626503 |
| 0.525514198 | 0.863141413 | 0.883068821 | 0.624226034 | 0.632196997 | 0.191374279 | 0.721514483 |
| 0.454629564 | 0.908903281 | 0.848267027 | 0.708063483 | 0.42815458 | 0.193936375 | 0.780371504 |

| 0.513344246 | 0.895238773 | 0.890826276 | 0.695324176 | 0.458543876 | 0.167603729 | 0.786136218 |
| --- | --- | --- | --- | --- | --- | --- |
| 0.522026902 | 0.918155291 | 0.911821223 | 0.794036012 | 0.384456622 | 0.181125899 | 0.885346239 |
| 0.392214077 | 0.875453704 | 0.842573482 | 0.758593694 | 0.340046972 | 0.149028539 | 0.673973383 |
| 0.35677176 | 0.90591417 | 0.868834958 | 0.638459896 | 0.454487225 | 0.115507793 | 0.769909615 |
| 0.746139065 | 0.895381112 | 0.917514768 | 0.779802149 | 0.563660949 | 0.142480962 | 0.786207387 |
| 0.564870828 | 0.905985339 | 0.910184329 | 0.7300548 | 0.40438403 | 0.144117856 | 0.760586435 |
| 0.468080564 | 0.88620027 | 0.870116006 | 0.713685859 | 0.446872109 | 0.151092449 | 0.764144901 |
| 0.355419543 | 0.882712974 | 0.922354281 | 0.751547933 | 0.465589638 | 0.188456338 | 0.709842716 |
| 0.543235357 | 0.898227884 | 0.882855313 | 0.730908832 | 0.364600384 | 0.162194862 | 0.751903779 |
| 0.410931606 | 0.728916091 | 0.883567006 | 0.568713971 | 0.392854601 | 0.173226105 | 0.674613906 |
| 0.299480464 | 0.890755106 | 0.796455768 | 0.792399118 | 0.410219913 | 0.151661803 | 0.720731621 |
| 0.363604014 | 0.895025265 | 0.865062985 | 0.726923351 | 0.41569995 | 0.14269447 | 0.719165896 |
| 0.393068109 | 0.873247456 | 0.870044837 | 0.822219059 | 0.465162622 | 0.096647925 | 0.74770479 |
| 0.431072522 | 0.887694826 | 0.904277276 | 0.688918938 | 0.445875738 | 0.119493275 | 0.703010462 |
| 0.352857448 | 0.965554053 | 0.814034588 | 0.730695324 | 0.484520675 | 0.171162195 | 0.572770621 |
| 0.312362109 | 0.890541598 | 0.833890826 | 0.795032382 | 0.516119849 | 0.137214433 | 0.787417266 |
| 0.423528574 | 0.905629493 | 0.77247171 | 0.75902071 | 0.860508149 | 0.199772258 | 0.634545584 |
| 0.465803146 | 0.854458757 | 0.885773255 | 0.673119351 | 0.416696321 | 0.118852751 | 0.825350509 |
| 0.399473347 | 0.74870116 | 0.893174863 | 0.741014874 | 0.620809907 | 0.124546296 | 0.607857092 |
| 0.485730553 | 0.898014376 | 0.902142196 | 0.650202833 | 0.392498755 | 0.122482386 | 0.789694684 |
| 0.442459611 | 0.911536545 | 0.916376059 | 0.712831827 | 0.385452993 | 0.155362608 | 0.765354779 |
| 0.377553199 | 0.838730339 | 0.77873461 | 0.755319906 | 0.436694897 | 0.216852893 | 0.810547292 |
| 0.415201765 | 0.923350651 | 0.919863355 | 0.785139848 | 0.566507722 | 0.194221052 | 0.795032382 |
| 0.470429151 | 0.870258345 | 0.91516618 | 0.772827557 | 0.485090029 | 0.213792613 | 0.745498541 |
| 0.334993951 | 0.963347804 | 0.848836382 | 0.717742509 | 0.466158992 | 0.193580528 | 0.735748345 |
| 0.619101843 | 0.893174863 | 0.893673048 | 0.706497758 | 0.543306526 | 0.121414846 | 0.621806277 |
| 0.388513273 | 0.879225678 | 0.861077503 | 0.764571917 | 0.65255142 | 0.146252936 | 0.801935805 |
| 0.536118426 | 0.8852039 | 0.900291794 | 0.705501388 | 0.493843855 | 0.147605153 | 0.817166038 |
| 0.348871966 | 0.931819799 | 0.895167604 | 0.787203758 | 0.548715394 | 0.195929115 | 0.803928546 |
| 0.489644865 | 0.898156715 | 0.930823429 | 0.706213081 | 0.538894029 | 0.154935592 | 0.705216711 |
| 0.347662088 | 0.920076863 | 0.861148673 | 0.873959149 | 0.730481816 | 0.174934168 | 0.560102484 |
| 0.431357199 | 0.926197424 | 0.846060779 | 0.879652694 | 0.493915024 | 0.180556544 | 0.68044979 |
| 0.385880009 | 0.849690413 | 0.859867625 | 0.78079852 | 0.462671696 | 0.14269447 | 0.691267525 |
| 0.336274998 | 0.896235143 | 0.873318625 | 0.860792826 | 0.645149811 | 0.144616042 | 0.789338837 |
| 0.359476194 | 0.913956302 | 0.866771048 | 0.81182834 | 0.592982706 | 0.158280549 | 0.752900149 |
| 0.41569995 | 0.905700662 | 0.87595189 | 0.660237706 | 0.377980215 | 0.160202121 | 0.683011885 |
| 0.314283681 | 0.895452281 | 0.835954736 | 0.7973098 | 0.673759875 | 0.222617607 | 0.729556615 |
| 0.405807416 | 0.881431927 | 0.832752117 | 0.731620525 | 0.459824923 | 0.173083766 | 0.756529784 |
| 0.423172728 | 0.617963134 | 0.891182122 | 0.742367091 | 0.447654971 | 0.134509999 | 0.733542097 |
| 0.322539321 | 0.915735535 | 0.893317202 | 0.756245107 | 0.515550495 | 0.107038645 | 0.717244324 |
| 0.302540744 | 0.873532133 | 0.783360615 | 0.787417266 | 0.589637748 | 0.21407729 | 0.667852822 |
| 0.356202406 | 0.90385026 | 0.845064408 | 0.670272578 | 0.419614262 | 0.193794036 | 0.827414419 |
| 0.611273219 | 0.767774536 | 0.861219842 | 0.737242901 | 0.391502384 | 0.130026333 | 0.694043129 |
| 0.399900363 | 0.931962138 | 0.895879297 | 0.709913885 | 0.489075511 | 0.181125899 | 0.697815102 |
| 0.642374208 | 0.907052879 | 0.799017863 | 0.770336631 | 0.472279553 | 0.199914597 | 0.696534055 |
| 0.428225749 | 0.893103694 | 0.862216212 | 0.679524589 | 0.532346452 | 0.143263825 | 0.821151519 |
| 0.406519109 | 0.905202477 | 0.817593054 | 0.71453989 | 0.329015728 | 0.187673475 | 0.795032382 |
| 0.560671838 | 0.922923635 | 0.85936944 | 0.640310298 | 0.322325813 | 0.156358978 | 0.816027329 |
| 0.398548146 | 0.925841577 | 0.910469006 | 0.638886912 | 0.459611416 | 0.184328518 | 0.722653192 |
| 0.329727422 | 0.909828482 | 0.733470927 | 0.759661234 | 0.415344104 | 0.147605153 | 0.786420895 |

| 0.358408654 | 0.913031101 | 0.876236567 | 0.636823002 | 0.442744289 | 0.152871682 | 0.767703366 |
| --- | --- | --- | --- | --- | --- | --- |
| 0.307878443 | 0.912248239 | 0.918511138 | 0.659668351 | 0.460323109 | 0.134011814 | 0.799017863 |
| 0.604085118 | 0.910469006 | 0.86143335 | 0.645434489 | 0.412995516 | 0.139064835 | 0.75596043 |
| 0.39292577 | 0.920930895 | 0.829691837 | 0.734538467 | 0.451782791 | 0.17272792 | 0.865703509 |
| 0.437050744 | 0.884776884 | 0.869546651 | 0.739662657 | 0.461746495 | 0.183403316 | 0.760444097 |
| 0.292292364 | 0.879368017 | 0.860579318 | 0.652124404 | 0.519891823 | 0.189096861 | 0.712333642 |
| 0.37534695 | 0.901003487 | 0.745925557 | 0.760301758 | 0.531207743 | 0.147035798 | 0.705572557 |
| 0.462173511 | 0.907764572 | 0.873745641 | 0.722368515 | 0.500106754 | 0.167105544 | 0.724147747 |
| 0.314497189 | 0.928546011 | 0.779802149 | 0.789979361 | 0.419044908 | 0.173368444 | 0.789908192 |
| 0.368230019 | 0.909543805 | 0.812753541 | 0.685858658 | 0.65973952 | 0.130738026 | 0.649704647 |
| 0.377553199 | 0.874030318 | 0.812041848 | 0.738737456 | 0.455768273 | 0.144402534 | 0.785780372 |
| 0.333855242 | 0.927336133 | 0.815315636 | 0.741086044 | 0.44566223 | 0.1623372 | 0.723009039 |
| 0.425877162 | 0.894028895 | 0.891182122 | 0.702014092 | 0.463169881 | 0.154366237 | 0.725357626 |
| 0.320190734 | 0.906554694 | 0.829122482 | 0.714397552 | 0.526937585 | 0.149028539 | 0.69496833 |
| 0.324176215 | 0.903280905 | 0.843498683 | 0.7363177 | 0.463668066 | 0.160486798 | 0.756316276 |
| 0.446231585 | 0.757383816 | 0.946836524 | 0.686000996 | 0.398192299 | 0.157070671 | 0.77873461 |
| 0.385025977 | 0.832752117 | 0.853960572 | 0.685716319 | 0.482029749 | 0.170236994 | 0.810831969 |
| 0.39292577 | 0.879652694 | 0.918866985 | 0.735463668 | 0.405238061 | 0.174649491 | 0.733043911 |
| 0.338054231 | 0.924560529 | 0.839797879 | 0.725286456 | 0.505230944 | 0.134865846 | 0.639313928 |
| 0.423172728 | 0.907906911 | 0.88214362 | 0.726353996 | 0.392000569 | 0.169382962 | 0.828624297 |
| 0.302540744 | 0.906341186 | 0.756458615 | 0.755604583 | 0.487225109 | 0.108960216 | 0.676464309 |
| 0.466870685 | 0.89758736 | 0.930040567 | 0.652622589 | 0.443954167 | 0.152373497 | 0.80976443 |
| 0.429862643 | 0.889687567 | 0.888193011 | 0.738808626 | 0.382819728 | 0.142196285 | 0.683367732 |
| 0.442459611 | 0.804070885 | 0.900932318 | 0.575830902 | 0.406874956 | 0.115365454 | 0.726567504 |
| 0.337911892 | 0.901501672 | 0.845206747 | 0.740374351 | 0.482741442 | 0.153156359 | 0.772685218 |
| 0.383531421 | 0.855028112 | 0.917941784 | 0.720019927 | 0.355988898 | 0.14475838 | 0.734396128 |
| 0.484520675 | 0.911607715 | 0.871397054 | 0.719023557 | 0.381396342 | 0.187815814 | 0.798021493 |
| 0.365952601 | 0.857234361 | 0.893388371 | 0.72905843 | 0.399402178 | 0.186605936 | 0.645363319 |
| 0.592911537 | 0.788057789 | 0.820653334 | 0.82933599 | 0.486157569 | 0.20888193 | 0.651412711 |
| 0.274571205 | 0.905273646 | 0.867553911 | 0.741299552 | 0.445591061 | 0.178279126 | 0.770692477 |
| 0.38054231 | 0.887552487 | 0.823784784 | 0.738452779 | 0.473062415 | 0.179417835 | 0.770763647 |
| 0.334638104 | 0.925983916 | 0.876165397 | 0.799587218 | 0.739662657 | 0.130595687 | 0.737812255 |
| 0.275709914 | 0.931036937 | 0.832325101 | 0.78485517 | 0.74564088 | 0.151946481 | 0.704433848 |
| 0.298768771 | 0.89758736 | 0.906127678 | 0.726353996 | 0.555689986 | 0.125400327 | 0.721229806 |
| 0.64422461 | 0.872606932 | 0.909686143 | 0.731193509 | 0.440253363 | 0.275140559 | 0.882641805 |
| 0.276279268 | 0.883424667 | 0.825350509 | 0.72592698 | 0.549000071 | 0.143619671 | 0.579602875 |
| 0.367589495 | 0.84904989 | 0.93480891 | 0.80250516 | 0.627143976 | 0.209451285 | 0.802932176 |
| 0.341968543 | 0.789837022 | 0.903280905 | 0.788627144 | 0.601665362 | 0.121770692 | 0.842431144 |
| 0.295921998 | 0.932673831 | 0.902640381 | 0.747918298 | 0.531705928 | 0.135577539 | 0.682086684 |
| 0.412070315 | 0.904277276 | 0.871468223 | 0.740232012 | 0.527720447 | 0.181125899 | 0.747135435 |
| 0.301117358 | 0.929115365 | 0.920859725 | 0.671411287 | 0.581737955 | 0.135221692 | 0.743861647 |
| 0.381823358 | 0.927691979 | 0.859084763 | 0.783218276 | 0.434630987 | 0.155433777 | 0.816098498 |
| 0.408938866 | 0.952814746 | 0.815030959 | 0.762792684 | 0.787346096 | 0.153939221 | 0.698597965 |
| 0.527222262 | 0.910896022 | 0.900505302 | 0.748629991 | 0.472279553 | 0.085759021 | 0.853391218 |
| 0.434702156 | 0.932745 | 0.854031742 | 0.748629991 | 0.661234076 | 0.140701729 | 0.657960288 |
| 0.4322824 | 0.892392001 | 0.92655327 | 0.709202192 | 0.688278414 | 0.125613835 | 0.749982208 |
| 0.22539321 | 0.942637535 | 0.809408583 | 0.753540673 | 0.591061134 | 0.137356772 | 0.652337912 |
| 0.362180628 | 0.883994022 | 0.881716604 | 0.8583019 | 0.715109245 | 0.119849121 | 0.679097573 |
| 0.475197495 | 0.900861149 | 0.834958366 | 0.736958224 | 0.475055156 | 0.125400327 | 0.722083837 |
| 0.304533485 | 0.948758096 | 0.857661376 | 0.780513842 | 0.652978436 | 0.138210804 | 0.66080706 |

| 0.391004199 | 0.909401466 | 0.828481959 | 0.718169525 | 0.440395701 | 0.161411999 | 0.787417266 |
| --- | --- | --- | --- | --- | --- | --- |
| 0.398690485 | 0.894598249 | 0.890683937 | 0.724147747 | 0.402106612 | 0.160344459 | 0.825564017 |
| 0.370365099 | 0.915023842 | 0.902996228 | 0.760017081 | 0.456195289 | 0.168884777 | 0.706213081 |
| 0.4932745 | 0.907337556 | 0.876378905 | 0.736460038 | 0.456693474 | 0.121841862 | 0.754679382 |
| 0.423457405 | 0.842431144 | 0.846131948 | 0.69183688 | 0.505800299 | 0.139776528 | 0.74457334 |
| 0.280549427 | 0.948473418 | 0.823927123 | 0.763717885 | 0.41776386 | 0.139207174 | 0.690199986 |
| 0.370578607 | 0.930609921 | 0.939933101 | 0.741299552 | 0.425805992 | 0.211372856 | 0.7363177 |
| 0.30816312 | 0.813963419 | 0.898441392 | 0.53775532 | 0.471425521 | 0.208810761 | 0.607928261 |
| 0.250800655 | 0.906554694 | 0.861362181 | 0.758309017 | 0.520603516 | 0.149170878 | 0.747491282 |
| 0.358622162 | 0.927691979 | 0.832823287 | 0.751263255 | 0.613764145 | 0.178563803 | 0.598462743 |
| 0.347235072 | 0.860792826 | 0.907622233 | 0.720589282 | 0.665504235 | 0.134723507 | 0.613621806 |
| 0.343534268 | 0.898939577 | 0.910682514 | 0.754964059 | 0.570493203 | 0.136289232 | 0.798163832 |
| 0.374279411 | 0.909401466 | 0.926339762 | 0.716959647 | 0.479254146 | 0.13031101 | 0.725428795 |
| 0.273076649 | 0.919009323 | 0.895381112 | 0.716247954 | 0.505942638 | 0.122553555 | 0.721300975 |
| 0.51704505 | 0.802576329 | 0.927051455 | 0.794818874 | 0.640310298 | 0.158422888 | 0.735677176 |
| 0.42189168 | 0.830830546 | 0.949540958 | 0.841577112 | 0.458472707 | 0.171233364 | 0.725499964 |
| 0.400469717 | 0.840367234 | 0.867482741 | 0.685858658 | 0.455127749 | 0.137356772 | 0.727421536 |
| 0.359974379 | 0.694612483 | 0.915949043 | 0.767916874 | 0.602519394 | 0.105757597 | 0.654330653 |
| 0.282542168 | 0.949540958 | 0.962636111 | 0.774108604 | 0.681303822 | 0.130453349 | 0.714753398 |
| 0.25542666 | 0.91103836 | 0.947904064 | 0.756743292 | 0.664863711 | 0.159561597 | 0.733043911 |
| 0.303892961 | 0.92448936 | 0.861860366 | 0.668635684 | 0.407159633 | 0.120276137 | 0.825065832 |
| 0.238701872 | 0.923564159 | 0.862002704 | 0.771831186 | 0.410148744 | 0.199202904 | 0.758095509 |
| 0.380186464 | 0.902426874 | 0.8511138 | 0.769126753 | 0.632552843 | 0.174080137 | 0.700163689 |
| 0.325599601 | 0.979645577 | 0.812113017 | 0.737385239 | 0.295281475 | 0.172870258 | 0.780300334 |
| 0.550636965 | 0.921642588 | 0.924845207 | 0.639313928 | 0.365952601 | 0.174435983 | 0.80044125 |
| 0.356842929 | 0.919934524 | 0.892036154 | 0.841861789 | 0.7569568 | 0.155860793 | 0.645078642 |
| 0.259412141 | 0.904134937 | 0.910255498 | 0.739093303 | 0.363034659 | 0.156074301 | 0.745285033 |
| 0.270799231 | 0.932531492 | 0.849405736 | 0.800298911 | 0.479467654 | 0.212226888 | 0.59767988 |
| 0.270443385 | 0.90484663 | 0.849761583 | 0.753611843 | 0.443455982 | 0.141911608 | 0.743648139 |
| 0.248309729 | 0.894598249 | 0.843783361 | 0.772258202 | 0.504946267 | 0.175717031 | 0.659526012 |
| 0.390790691 | 0.970749413 | 0.878656323 | 0.720731621 | 0.414845918 | 0.130524518 | 0.804711408 |
| 0.335634474 | 0.912817593 | 0.899224255 | 0.686143335 | 0.345811686 | 0.16959647 | 0.775959006 |
| 0.306953242 | 0.944060921 | 0.87901217 | 0.781083197 | 0.516262188 | 0.135648708 | 0.785780372 |
| 0.356131236 | 0.920148032 | 0.825991033 | 0.657533272 | 0.371503808 | 0.171446872 | 0.78286243 |
| 0.346523379 | 0.920646217 | 0.905060138 | 0.721372144 | 0.367304818 | 0.146964629 | 0.836310583 |
| 0.388655612 | 0.882357128 | 0.874457334 | 0.7228667 | 0.336986691 | 0.180485375 | 0.757454985 |
| 0.353782649 | 0.929257704 | 0.857661376 | 0.701444737 | 0.501174294 | 0.104547719 | 0.660735891 |
| 0.282257491 | 0.945840154 | 0.87488435 | 0.67112661 | 0.457903352 | 0.212369226 | 0.73218988 |
| 0.361041919 | 0.886057932 | 0.928546011 | 0.674400399 | 0.317415131 | 0.217778094 | 0.828624297 |
| 0.470286812 | 0.887196641 | 0.880862572 | 0.703650986 | 0.466799516 | 0.137356772 | 0.752971319 |
| 0.514198278 | 0.907622233 | 0.894171233 | 0.614191161 | 0.407301971 | 0.20368657 | 0.73218988 |
| 0.397551776 | 0.841719451 | 0.883211159 | 0.642587716 | 0.306455057 | 0.146395274 | 0.798733186 |
| 0.350366522 | 0.871112376 | 0.900861149 | 0.653690129 | 0.408084834 | 0.177425094 | 0.697743933 |
| 0.505586791 | 0.923137143 | 0.88207245 | 0.679026404 | 0.489431357 | 0.11586364 | 0.781012028 |
| 0.311081062 | 0.891964985 | 0.850615615 | 0.671767134 | 0.328659882 | 0.132019073 | 0.781937229 |
| 0.268948829 | 0.905558323 | 0.876663583 | 0.726781012 | 0.520461177 | 0.121628354 | 0.720731621 |
| 0.446018077 | 0.944416767 | 0.861575689 | 0.761369298 | 0.4798235 | 0.115436624 | 0.701729414 |
| 0.470713828 | 0.917657106 | 0.853035371 | 0.702583446 | 0.585652267 | 0.132659597 | 0.756458615 |
| 0.301686713 | 0.842431144 | 0.902071027 | 0.793110811 | 0.45712049 | 0.1820511 | 0.734040282 |
| 0.651839727 | 0.889972244 | 0.89965127 | 0.661162907 | 0.435769696 | 0.130524518 | 0.681730838 |

| 0.416055797 | 0.906554694 | 0.882641805 | 0.697103409 | 0.406661448 | 0.164614618 | 0.771546509 |
| --- | --- | --- | --- | --- | --- | --- |
| 0.338979432 | 0.917087752 | 0.814176927 | 0.727279197 | 0.418333215 | 0.132873105 | 0.719308234 |
| 0.368870543 | 0.953597609 | 0.917585937 | 0.68770906 | 0.524375489 | 0.152800512 | 0.757383816 |
| 0.326097787 | 0.916660736 | 0.867126895 | 0.717742509 | 0.483026119 | 0.097501957 | 0.759163049 |
| 0.461034802 | 0.915522027 | 0.917585937 | 0.594975447 | 0.386235855 | 0.147747491 | 0.821222689 |
| 0.521671055 | 0.662586293 | 0.900078286 | 0.843783361 | 0.615472208 | 0.128175931 | 0.732332218 |
| 0.596042986 | 0.883282329 | 0.762579176 | 0.839513202 | 0.669205039 | 0.165041634 | 0.369439898 |
| 0.519322468 | 0.876592413 | 0.911750053 | 0.864920646 | 0.69696107 | 0.139990036 | 0.581453277 |
| 0.598249235 | 0.881574265 | 0.879154509 | 0.830759377 | 0.651555049 | 0.145541243 | 0.687566721 |
| 0.599174436 | 0.824425308 | 0.755889261 | 0.845349085 | 0.686072166 | 0.138353142 | 0.581097431 |
| 0.502811188 | 0.903992598 | 0.783716461 | 0.792541456 | 0.788128959 | 0.185538396 | 0.566863568 |
| 0.57704078 | 0.777738239 | 0.841719451 | 0.935520604 | 0.783147107 | 0.170094655 | 0.588285531 |
| 0.660451213 | 0.738737456 | 0.728133229 | 0.824709985 | 0.621663939 | 0.141057576 | 0.709344531 |
| 0.617749626 | 0.636253647 | 0.922710127 | 0.836879937 | 0.64529215 | 0.13337129 | 0.674898584 |
| 0.548003701 | 0.904206106 | 0.736887054 | 0.855312789 | 0.764287239 | 0.155220269 | 0.697815102 |
| 0.715322753 | 0.825706355 | 0.819229948 | 0.829620668 | 0.612056081 | 0.136573909 | 0.703935663 |
| 0.561881717 | 0.859298271 | 0.862714398 | 0.812326525 | 0.65667924 | 0.125400327 | 0.689844139 |
| 0.581737955 | 0.85310654 | 0.786492065 | 0.827699096 | 0.716390293 | 0.131449719 | 0.558109743 |
| 0.593765568 | 0.737598747 | 0.903992598 | 0.808341043 | 0.617464949 | 0.134154153 | 0.739947335 |
| 0.605935521 | 0.923137143 | 0.924702868 | 0.831186392 | 0.624724219 | 0.139064835 | 0.76934026 |
| 0.551562166 | 0.857519038 | 0.873674472 | 0.863924276 | 0.777738239 | 0.134723507 | 0.768201551 |
| 0.643157071 | 0.857946054 | 0.852821863 | 0.869902498 | 0.726709843 | 0.150024909 | 0.764002562 |
| 0.627570991 | 0.884349868 | 0.777382393 | 0.79111807 | 0.578179489 | 0.1686001 | 0.657035086 |
| 0.754252366 | 0.825421678 | 0.758664864 | 0.806988826 | 0.557469219 | 0.177638602 | 0.796384599 |
| 0.521742225 | 0.793324319 | 0.886413778 | 0.917799445 | 0.637250018 | 0.160913814 | 0.669987901 |
| 0.671624795 | 0.885061561 | 0.838943847 | 0.863141413 | 0.677958864 | 0.115436624 | 0.744359832 |
| 0.570920219 | 0.773325742 | 0.908903281 | 0.879581524 | 0.701017721 | 0.12924347 | 0.723436054 |
| 0.503167034 | 0.874955519 | 0.805494271 | 0.893317202 | 0.658316134 | 0.156928333 | 0.809479752 |
| 0.707921144 | 0.827627927 | 0.712048964 | 0.909757313 | 0.621165753 | 0.164258772 | 0.800156572 |
| 0.575261547 | 0.929755889 | 0.819443456 | 0.921571418 | 0.816098498 | 0.155504946 | 0.734823144 |
| 0.549569426 | 0.881147249 | 0.926410932 | 0.874030318 | 0.729556615 | 0.150380756 | 0.730837663 |
| 0.652124404 | 0.893103694 | 0.884848054 | 0.928759519 | 0.7766707 | 0.141413423 | 0.773396911 |
| 0.678670557 | 0.842431144 | 0.766066472 | 0.807344673 | 0.622304462 | 0.120916661 | 0.730197139 |
| 0.652622589 | 0.810974308 | 0.891182122 | 0.850188599 | 0.620881076 | 0.198491211 | 0.725855811 |
| 0.654686499 | 0.920859725 | 0.948402249 | 0.815600313 | 0.696605224 | 0.120418476 | 0.859013593 |
| 0.544302897 | 0.911394207 | 0.541669632 | 0.894455911 | 0.455341257 | 0.257704078 | 0.689203615 |
| 0.621450431 | 0.932958508 | 0.930182905 | 0.839299694 | 0.553910754 | 0.146039428 | 0.649206462 |
| 0.666856451 | 0.728773753 | 0.859725286 | 0.859440609 | 0.708205822 | 0.162621877 | 0.750053377 |
| 0.601950039 | 0.76627998 | 0.860009964 | 0.831898086 | 0.676962494 | 0.137854957 | 0.564230304 |
| 0.736744716 | 0.912390577 | 0.920432709 | 0.826346879 | 0.538182336 | 0.165539819 | 0.791687424 |
| 0.767205181 | 0.838872678 | 0.870898868 | 0.719308234 | 0.59148815 | 0.12411928 | 0.840865419 |
| 0.643370579 | 0.868977297 | 0.721443314 | 0.804426731 | 0.69802861 | 0.149455555 | 0.602234716 |
| 0.670201409 | 0.860863995 | 0.817521885 | 0.844993239 | 0.668635684 | 0.155433777 | 0.779873319 |
| 0.787773112 | 0.834602519 | 0.774037435 | 0.750124546 | 0.595046616 | 0.165753327 | 0.732688065 |
| 0.50359405 | 0.827058572 | 0.822076721 | 0.890612768 | 0.677104832 | 0.136787417 | 0.608284108 |
| 0.736815885 | 0.817450715 | 0.848124689 | 0.821365027 | 0.623158494 | 0.122838232 | 0.816383176 |
| 0.556757526 | 0.846416625 | 0.744359832 | 0.828054943 | 0.659027827 | 0.136360401 | 0.794391858 |
| 0.644438118 | 0.82008398 | 0.866272863 | 0.801224112 | 0.701800584 | 0.130168671 | 0.711550779 |
| 0.707565298 | 0.938509715 | 0.89452708 | 0.77973098 | 0.583303679 | 0.136146893 | 0.747847128 |
| 0.58223614 | 0.879296847 | 0.876805921 | 0.813465234 | 0.605223827 | 0.157284179 | 0.72905843 |

| 0.634118568 | 0.853035371 | 0.857234361 | 0.790477546 | 0.740374351 | 0.109743079 | 0.82214789 |
| --- | --- | --- | --- | --- | --- | --- |
| 0.6287097 | 0.902996228 | 0.908120418 | 0.816667853 | 0.658529642 | 0.130738026 | 0.777524731 |
| 0.584157711 | 0.83759163 | 0.684364102 | 0.843712191 | 0.78079852 | 0.155718454 | 0.676464309 |
| 0.511707352 | 0.890470429 | 0.50160131 | 0.892107323 | 0.71048324 | 0.139349512 | 0.651982065 |
| 0.649704647 | 0.830332361 | 0.822076721 | 0.73112234 | 0.616112732 | 0.171233364 | 0.745427372 |
| 0.618532489 | 0.708134652 | 0.900647641 | 0.814746281 | 0.609849833 | 0.132019073 | 0.789338837 |
| 0.607785923 | 0.899010747 | 0.746850758 | 0.845135578 | 0.679239912 | 0.153583375 | 0.738879795 |
| 0.476265035 | 0.897800868 | 0.892819016 | 0.834602519 | 0.666358266 | 0.136645079 | 0.723791901 |
| 0.598249235 | 0.791189239 | 0.8645648 | 0.864066614 | 0.60906697 | 0.133086613 | 0.79424952 |
| 0.55327023 | 0.797594477 | 0.894100064 | 0.853035371 | 0.697815102 | 0.129385809 | 0.704505017 |
| 0.585652267 | 0.862145043 | 0.897302683 | 0.833392641 | 0.57604441 | 0.173154936 | 0.788057789 |
| 0.597466373 | 0.816667853 | 0.831044054 | 0.850900292 | 0.635470785 | 0.129385809 | 0.777738239 |
| 0.509074087 | 0.88833535 | 0.844210376 | 0.846558964 | 0.705786065 | 0.13963419 | 0.726638673 |
| 0.814461604 | 0.90591417 | 0.890470429 | 0.843996869 | 0.756600954 | 0.13130738 | 0.767916874 |
| 0.693544943 | 0.920076863 | 0.855668636 | 0.878656323 | 0.704433848 | 0.14162693 | 0.601451854 |
| 0.631342965 | 0.887552487 | 0.922852466 | 0.866913387 | 0.781439043 | 0.143975518 | 0.667212298 |
| 0.662443954 | 0.820653334 | 0.821863213 | 0.912604085 | 0.668849192 | 0.174008967 | 0.513771262 |
| 0.621806277 | 0.73944915 | 0.870471853 | 0.785282186 | 0.646786706 | 0.128104761 | 0.712689488 |
| 0.520247669 | 0.882428297 | 0.833606149 | 0.858088392 | 0.635470785 | 0.141199915 | 0.657533272 |
| 0.664365526 | 0.844210376 | 0.858728916 | 0.784641663 | 0.489929542 | 0.179844851 | 0.792327948 |
| 0.605793182 | 0.812753541 | 0.848907551 | 0.827627927 | 0.609707494 | 0.132588428 | 0.788200128 |
| 0.524944844 | 0.889545228 | 0.828624297 | 0.84591844 | 0.737100562 | 0.167247883 | 0.651555049 |
| 0.655540531 | 0.902284535 | 0.879296847 | 0.831684578 | 0.671340118 | 0.1551491 | 0.83040353 |
| 0.691125187 | 0.750622731 | 0.456124119 | 0.899153085 | 0.786349726 | 0.136929756 | 0.677745356 |
| 0.757526155 | 0.799444879 | 0.88833535 | 0.769838446 | 0.595402462 | 0.139491851 | 0.747277774 |
| 0.627286314 | 0.781652551 | 0.97203046 | 0.848551705 | 0.644011102 | 0.142196285 | 0.849832752 |
| 0.582947833 | 0.894028895 | 0.742295922 | 0.767845705 | 0.646502028 | 0.146608782 | 0.697316917 |
| 0.68564515 | 0.811543662 | 0.884563376 | 0.767134012 | 0.680022774 | 0.139064835 | 0.714468721 |
| 0.568215785 | 0.837306953 | 0.851469646 | 0.819301117 | 0.542167817 | 0.19955875 | 0.770692477 |
| 0.595402462 | 0.864351292 | 0.873105117 | 0.90171518 | 0.65148388 | 0.150594264 | 0.717173155 |
| 0.556045833 | 0.870187175 | 0.855028112 | 0.815244467 | 0.687566721 | 0.140559391 | 0.758380186 |
| 0.713970536 | 0.887125471 | 0.816240837 | 0.906341186 | 0.707280621 | 0.169738809 | 0.709344531 |
| 0.53462387 | 0.928830688 | 0.885275069 | 0.876663583 | 0.640452637 | 0.137214433 | 0.633335706 |
| 0.61013451 | 0.874457334 | 0.840936588 | 0.82527934 | 0.597395203 | 0.145256565 | 0.830617038 |
| 0.627642161 | 0.828268451 | 0.788769483 | 0.907479895 | 0.708775176 | 0.11892392 | 0.734609636 |
| 0.500747278 | 0.960216355 | 0.725072949 | 0.820724504 | 0.596327664 | 0.183759163 | 0.700234859 |
| 0.590918796 | 0.873176286 | 0.858657747 | 0.559960145 | 0.550992812 | 0.235001068 | 0.683225393 |
| 0.482812611 | 0.951462529 | 0.897800868 | 0.77873461 | 0.533271653 | 0.173510782 | 0.818589424 |
| 0.536189595 | 0.959006476 | 0.927976656 | 0.722653192 | 0.577538965 | 0.163760586 | 0.81389225 |
| 0.563305103 | 0.947761725 | 0.870258345 | 0.720375774 | 0.521884563 | 0.215927692 | 0.828837805 |
| 0.455697103 | 0.934737741 | 0.855526297 | 0.758878372 | 0.606433706 | 0.192726496 | 0.675894954 |
| 0.49014305 | 0.954949826 | 0.836239414 | 0.703437478 | 0.545370436 | 0.174151306 | 0.735392499 |
| 0.591203473 | 0.943918582 | 0.905202477 | 0.726567504 | 0.602661732 | 0.165112803 | 0.737883425 |
| 0.413066686 | 0.958365953 | 0.918795815 | 0.705359049 | 0.596470002 | 0.19955875 | 0.705430218 |
| 0.548715394 | 0.939719593 | 0.846416625 | 0.713258843 | 0.539249875 | 0.163902925 | 0.754323536 |
| 0.561098854 | 0.948544588 | 0.874101487 | 0.659383674 | 0.507223685 | 0.179773682 | 0.799017863 |
| 0.543662373 | 0.941142979 | 0.898156715 | 0.681303822 | 0.602732902 | 0.174151306 | 0.799516049 |
| 0.530709558 | 0.974450217 | 0.789979361 | 0.748131806 | 0.650914526 | 0.177567433 | 0.839157355 |
| 0.522098071 | 0.934737741 | 0.842288805 | 0.681232652 | 0.543804711 | 0.169667639 | 0.763504377 |
| 0.62671696 | 0.92448936 | 0.808341043 | 0.697672764 | 0.643157071 | 0.145754751 | 0.819087609 |

| 0.489716034 | 0.949540958 | 0.784285816 | 0.721514483 | 0.571276066 | 0.171091026 | 0.852608355 |
| --- | --- | --- | --- | --- | --- | --- |
| 0.433065262 | 0.935022418 | 0.820510996 | 0.769696107 | 0.55227386 | 0.166678528 | 0.66600242 |
| 0.507508362 | 0.947477048 | 0.715251584 | 0.720589282 | 0.607287738 | 0.176144047 | 0.677460679 |
| 0.515550495 | 0.951604868 | 0.908476265 | 0.746708419 | 0.64016796 | 0.173226105 | 0.721941499 |
| 0.367803003 | 0.950893175 | 0.71453989 | 0.676677817 | 0.733755605 | 0.159703936 | 0.676891324 |
| 0.547647854 | 0.941569995 | 0.894740588 | 0.688136076 | 0.628923208 | 0.152159989 | 0.675539108 |
| 0.567859939 | 0.956871397 | 0.844993239 | 0.697245748 | 0.550352288 | 0.173510782 | 0.719379404 |
| 0.503095865 | 0.96897018 | 0.80969326 | 0.738025763 | 0.628496192 | 0.160700306 | 0.728916091 |
| 0.49740232 | 0.950466159 | 0.919080492 | 0.760728774 | 0.79631343 | 0.198064195 | 0.667496975 |
| 0.468009394 | 0.964700021 | 0.886840794 | 0.705572557 | 0.711408441 | 0.173226105 | 0.748416483 |
| 0.525514198 | 0.959148815 | 0.842075297 | 0.765568287 | 0.637321187 | 0.170735179 | 0.726567504 |
| 0.563162764 | 0.949896804 | 0.846274287 | 0.809266244 | 0.669845563 | 0.162194862 | 0.657319764 |
| 0.533058145 | 0.954949826 | 0.865490001 | 0.755391075 | 0.595402462 | 0.168101914 | 0.596114156 |
| 0.519251299 | 0.964130667 | 0.802291652 | 0.798092662 | 0.813678742 | 0.183687994 | 0.672763504 |
| 0.57704078 | 0.953668778 | 0.787773112 | 0.710910255 | 0.563305103 | 0.169881147 | 0.769553768 |
| 0.540530923 | 0.956800228 | 0.953099424 | 0.761653975 | 0.63596897 | 0.181410576 | 0.815813821 |
| 0.506511992 | 0.954949826 | 0.88726781 | 0.844281546 | 0.733897943 | 0.166393851 | 0.579318198 |
| 0.564372643 | 0.892249662 | 0.819372287 | 0.755675753 | 0.490498897 | 0.203900078 | 0.765924134 |
| 0.675396769 | 0.9659099 | 0.792470287 | 0.735677176 | 0.494982564 | 0.172941428 | 0.860009964 |
| 0.787701943 | 0.940146609 | 0.849263398 | 0.621450431 | 0.424311437 | 0.190662586 | 0.872606932 |
| 0.554978293 | 0.953739947 | 0.809906768 | 0.770336631 | 0.618105473 | 0.192157142 | 0.845277916 |
| 0.584228881 | 0.894171233 | 0.904348445 | 0.513557754 | 0.523877304 | 0.19756601 | 0.739520319 |
| 0.561312362 | 0.867696249 | 0.841007757 | 0.747989467 | 0.449505373 | 0.171589211 | 0.844139207 |
| 0.513486585 | 0.932389154 | 0.899580101 | 0.702725785 | 0.479254146 | 0.174435983 | 0.848480535 |
| 0.594050246 | 0.945413138 | 0.71453989 | 0.710839086 | 0.50985695 | 0.165610989 | 0.774322112 |
| 0.556188172 | 0.960358693 | 0.90278272 | 0.73624653 | 0.670984272 | 0.166038004 | 0.758237848 |
| 0.576969611 | 0.95865063 | 0.932745 | 0.68564515 | 0.453704363 | 0.19543093 | 0.730624155 |
| 0.591843997 | 0.891964985 | 0.890968614 | 0.626147605 | 0.469646289 | 0.184186179 | 0.790477546 |
| 0.55227386 | 0.950110312 | 0.892392001 | 0.712191303 | 0.55433777 | 0.164543449 | 0.755818091 |
| 0.699523166 | 0.95039499 | 0.888904704 | 0.698384457 | 0.464806775 | 0.171375703 | 0.794890043 |
| 0.491495267 | 0.959362323 | 0.761440467 | 0.813109387 | 0.571560743 | 0.19962992 | 0.707422959 |
| 0.658387303 | 0.93281617 | 0.899864778 | 0.770692477 | 0.598106896 | 0.199701089 | 0.692050388 |
| 0.557896235 | 0.97722582 | 0.854885773 | 0.698242118 | 0.630133087 | 0.136645079 | 0.67119778 |
| 0.549640595 | 0.947334709 | 0.860650488 | 0.776314853 | 0.486513415 | 0.199274073 | 0.710910255 |
| 0.614618177 | 0.938367376 | 0.860650488 | 0.664009679 | 0.564941997 | 0.175645862 | 0.795815244 |
| 0.643299409 | 0.959860508 | 0.81588499 | 0.624937727 | 0.566365383 | 0.192441819 | 0.862145043 |
| 0.61120205 | 0.875169027 | 0.830190022 | 0.650558679 | 0.459326738 | 0.176357555 | 0.719308234 |
| 0.52330795 | 0.967475625 | 0.844708562 | 0.798235001 | 0.624012526 | 0.16753256 | 0.737812255 |
| 0.456195289 | 0.962636111 | 0.871183546 | 0.858586577 | 0.494413209 | 0.185751904 | 0.885132731 |
| 0.467226532 | 0.963276635 | 0.879439186 | 0.735677176 | 0.632624013 | 0.18511138 | 0.69902498 |
| 0.579958722 | 0.966621593 | 0.82627571 | 0.74357697 | 0.668422176 | 0.176642232 | 0.850473276 |
| 0.596612341 | 0.947975233 | 0.945128461 | 0.686000996 | 0.50366522 | 0.165041634 | 0.867767419 |
| 0.56665006 | 0.944701445 | 0.870827699 | 0.743932816 | 0.539534553 | 0.173510782 | 0.799871895 |
| 0.613621806 | 0.945341969 | 0.864280122 | 0.635114938 | 0.440111024 | 0.171019856 | 0.862572059 |
| 0.523094442 | 0.94420326 | 0.89659099 | 0.728346737 | 0.594904277 | 0.184257348 | 0.8179489 |
| 0.455697103 | 0.964771191 | 0.914383318 | 0.714397552 | 0.594121415 | 0.180271867 | 0.751049747 |
| 0.527293431 | 0.952245392 | 0.855597466 | 0.591416981 | 0.524090812 | 0.181837592 | 0.802647498 |
| 0.579816383 | 0.934381895 | 0.832111593 | 0.71041207 | 0.494413209 | 0.174364814 | 0.792470287 |
| 0.748487652 | 0.939790762 | 0.910469006 | 0.680236282 | 0.525798876 | 0.204682941 | 0.837022276 |
| 0.495694257 | 0.962137926 | 0.882784143 | 0.747206605 | 0.672763504 | 0.174507153 | 0.638317557 |

| 0.443740659 | 0.960145185 | 0.842288805 | 0.80250516 | 0.585652267 | 0.189950893 | 0.790691054 |
| --- | --- | --- | --- | --- | --- | --- |
| 0.479040638 | 0.938438545 | 0.848124689 | 0.735606007 | 0.443740659 | 0.204896449 | 0.755889261 |
| 0.411358622 | 0.949683297 | 0.82321543 | 0.747491282 | 0.4798235 | 0.202049676 | 0.788911821 |
| 0.679524589 | 0.917799445 | 0.767774536 | 0.547149669 | 0.474201124 | 0.248523237 | 0.836737599 |
| 0.509643442 | 0.924702868 | 0.849975091 | 0.684008256 | 0.431570707 | 0.196996655 | 0.738452779 |
| 0.648850616 | 0.922710127 | 0.7973098 | 0.721443314 | 0.650060494 | 0.176144047 | 0.702939293 |
| 0.456835812 | 0.956942566 | 0.922638958 | 0.725784642 | 0.614618177 | 0.164258772 | 0.79211444 |
| 0.758593694 | 0.971461106 | 0.846416625 | 0.796740446 | 0.449505373 | 0.213721443 | 0.81901644 |
| 0.62052523 | 0.919436339 | 0.865205323 | 0.617820796 | 0.55640168 | 0.221123052 | 0.76727635 |
| 0.67219415 | 0.918653477 | 0.89765853 | 0.702441107 | 0.531421251 | 0.176571063 | 0.927691979 |
| 0.505871468 | 0.975588926 | 0.888193011 | 0.769909615 | 0.603515764 | 0.173581951 | 0.749839869 |
| 0.50359405 | 0.932958508 | 0.775104975 | 0.638815743 | 0.628211515 | 0.20781439 | 0.796882784 |
| 0.584228881 | 0.917514768 | 0.843925699 | 0.714468721 | 0.583801865 | 0.248380898 | 0.795388229 |
| 0.493630347 | 0.964415344 | 0.873105117 | 0.700804213 | 0.641804854 | 0.206675681 | 0.702298769 |
| 0.504732759 | 0.953597609 | 0.796669276 | 0.723720732 | 0.695324176 | 0.173012597 | 0.641591346 |
| 0.578962351 | 0.961070386 | 0.847341826 | 0.69076934 | 0.581666785 | 0.183118639 | 0.809906768 |
| 0.574549854 | 0.917087752 | 0.83865917 | 0.681019145 | 0.512561384 | 0.155504946 | 0.821436197 |
| 0.687780229 | 0.957013736 | 0.920005694 | 0.752259626 | 0.641947192 | 0.159846274 | 0.75802434 |
| 0.563305103 | 0.953668778 | 0.902569212 | 0.717956017 | 0.485303537 | 0.170165824 | 0.772685218 |
| 0.670059071 | 0.946765355 | 0.775247313 | 0.665575404 | 0.571133727 | 0.162123692 | 0.843996869 |
| 0.700163689 | 0.957156074 | 0.87282044 | 0.686356843 | 0.497188812 | 0.166678528 | 0.752757811 |
| 0.567859939 | 0.963205466 | 0.736887054 | 0.852039001 | 0.801864636 | 0.15102128 | 0.606860722 |
| 0.520817024 | 0.92762081 | 0.847057149 | 0.584442388 | 0.636040139 | 0.169881147 | 0.765497118 |
| 0.5408156 | 0.952031884 | 0.910397836 | 0.770051954 | 0.835100705 | 0.165326311 | 0.781723721 |
| 0.632979859 | 0.877588784 | 0.913742794 | 0.610917372 | 0.58842787 | 0.190093232 | 0.722937869 |
| 0.571276066 | 0.908689773 | 0.861291011 | 0.590420611 | 0.534766209 | 0.199772258 | 0.714326382 |
| 0.590776457 | 0.896519821 | 0.843214006 | 0.52843214 | 0.476407373 | 0.192512988 | 0.720162266 |
| 0.568856309 | 0.946623016 | 0.869333144 | 0.775389652 | 0.588356701 | 0.130666856 | 0.795815244 |
| 0.479040638 | 0.94213935 | 0.876521244 | 0.708846345 | 0.59874742 | 0.155433777 | 0.762935022 |
| 0.501103124 | 0.969753042 | 0.887623657 | 0.720660451 | 0.57597324 | 0.135648708 | 0.795388229 |
| 0.616824425 | 0.88826418 | 0.710981425 | 0.629492563 | 0.443384812 | 0.187886983 | 0.707280621 |
| 0.543093018 | 0.932246815 | 0.836381752 | 0.635897801 | 0.534054516 | 0.190520248 | 0.778307594 |
| 0.621735108 | 0.968685503 | 0.733613266 | 0.764429578 | 0.868977297 | 0.137356772 | 0.732830404 |
| 0.492420468 | 0.968685503 | 0.81382108 | 0.810049107 | 0.633406875 | 0.158992243 | 0.621379261 |
| 0.435271511 | 0.965767561 | 0.827200911 | 0.803786207 | 0.677674187 | 0.211942211 | 0.714895737 |
| 0.456266458 | 0.964700021 | 0.825991033 | 0.784001139 | 0.511280336 | 0.197423671 | 0.653690129 |
| 0.553697246 | 0.961924418 | 0.891609138 | 0.733897943 | 0.681873176 | 0.182976301 | 0.724290086 |
| 0.501743648 | 0.978933884 | 0.803359192 | 0.78485517 | 0.82940716 | 0.197850687 | 0.715038076 |
| 0.527008754 | 0.952814746 | 0.866415202 | 0.815671482 | 0.512703722 | 0.206035158 | 0.733826774 |
| 0.495907765 | 0.890043413 | 0.857803715 | 0.759447726 | 0.603729272 | 0.233079496 | 0.784570493 |
| 0.39506085 | 0.940573625 | 0.785282186 | 0.770550139 | 0.730980001 | 0.194149883 | 0.603159917 |
| 0.636823002 | 0.904277276 | 0.897018006 | 0.618247812 | 0.630275425 | 0.151661803 | 0.729627784 |
| 0.514055939 | 0.962066757 | 0.935164757 | 0.721016298 | 0.514696463 | 0.197708348 | 0.832609779 |
| 0.637178848 | 0.961355064 | 0.911180699 | 0.69390079 | 0.596968187 | 0.229521031 | 0.76934026 |
| 0.571133727 | 0.952103053 | 0.859013593 | 0.700519536 | 0.627001637 | 0.178207957 | 0.819799303 |
| 0.426588855 | 0.973311508 | 0.781367874 | 0.738310441 | 0.636467155 | 0.213365597 | 0.625791759 |
| 0.734751975 | 0.939292577 | 0.847626503 | 0.629563732 | 0.516618034 | 0.178777311 | 0.79424952 |
| 0.624795388 | 0.939506085 | 0.946480678 | 0.619528859 | 0.440182193 | 0.138210804 | 0.850615615 |
| 0.521671055 | 0.867838588 | 0.77247171 | 0.711621949 | 0.52330795 | 0.167959576 | 0.68457761 |
| 0.549640595 | 0.867411572 | 0.928830688 | 0.799444879 | 0.641093161 | 0.175717031 | 0.695466515 |

| 0.629919579 | 0.905344815 | 0.836310583 | 0.580670415 | 0.497758167 | 0.236424454 | 0.750266885 |
| --- | --- | --- | --- | --- | --- | --- |
| 0.469005765 | 0.970393566 | 0.739591488 | 0.791972102 | 0.63390506 | 0.199843428 | 0.654401822 |
| 0.510782151 | 0.960287524 | 0.829264821 | 0.852394847 | 0.741726568 | 0.189737385 | 0.724503594 |
| 0.57191659 | 0.953597609 | 0.885417408 | 0.747348943 | 0.869261974 | 0.171091026 | 0.62358551 |
| 0.517827913 | 0.948971603 | 0.8107608 | 0.65767561 | 0.621806277 | 0.167319052 | 0.808412213 |
| 0.479894669 | 0.961070386 | 0.895665789 | 0.719308234 | 0.51192086 | 0.204611771 | 0.821151519 |
| 0.689132446 | 0.9255569 | 0.878300477 | 0.686000996 | 0.538111166 | 0.139918867 | 0.851683154 |
| 0.524375489 | 0.937157498 | 0.864991816 | 0.603017579 | 0.497615828 | 0.130880364 | 0.88933172 |
| 0.556757526 | 0.975660095 | 0.881645434 | 0.80037008 | 0.552060352 | 0.196569639 | 0.713757028 |
| 0.514340616 | 0.95445164 | 0.800797096 | 0.748843499 | 0.579033521 | 0.160130952 | 0.765568287 |
| 0.511778521 | 0.977724005 | 0.888193011 | 0.806134795 | 0.626218774 | 0.17479183 | 0.688420753 |
| 0.48081987 | 0.950964344 | 0.807771689 | 0.780300334 | 0.622802647 | 0.194078713 | 0.690555832 |
| 0.574478685 | 0.906697032 | 0.849619244 | 0.693758451 | 0.448722511 | 0.150309587 | 0.80770052 |
| 0.613906484 | 0.958721799 | 0.871183546 | 0.72386307 | 0.58636396 | 0.177140417 | 0.750693901 |
| 0.454629564 | 0.964913529 | 0.879439186 | 0.735107822 | 0.528147463 | 0.191231941 | 0.817877731 |
| 0.554907124 | 0.959219984 | 0.815315636 | 0.727848552 | 0.596398833 | 0.144473703 | 0.722155007 |
| 0.555476479 | 0.942210519 | 0.849192228 | 0.808483382 | 0.563305103 | 0.154366237 | 0.792612625 |
| 0.672763504 | 0.892392001 | 0.715892107 | 0.688491922 | 0.584798235 | 0.168315422 | 0.794747705 |
| 0.583944203 | 0.951249021 | 0.902498043 | 0.57597324 | 0.39712476 | 0.171019856 | 0.754110028 |
| 0.555405309 | 0.951249021 | 0.926197424 | 0.714468721 | 0.591915166 | 0.16852893 | 0.853675895 |
| 0.506227315 | 0.941569995 | 0.803216853 | 0.789267668 | 0.475553341 | 0.203900078 | 0.830047683 |
| 0.528147463 | 0.923065974 | 0.807131165 | 0.821934382 | 0.450644082 | 0.298982279 | 0.815600313 |
| 0.512632553 | 0.964984699 | 0.75389652 | 0.70108889 | 0.603658103 | 0.148815031 | 0.780656181 |
| 0.545299267 | 0.918653477 | 0.871895239 | 0.792327948 | 0.415059426 | 0.221123052 | 0.769553768 |
| 0.455697103 | 0.978791545 | 0.888975874 | 0.697459256 | 0.838801509 | 0.177425094 | 0.647213721 |
| 0.539321045 | 0.947121201 | 0.814817451 | 0.589922425 | 0.598391574 | 0.18098356 | 0.748843499 |
| 0.472991246 | 0.957369582 | 0.825065832 | 0.766137641 | 0.636680663 | 0.177211586 | 0.751263255 |
| 0.621450431 | 0.934666572 | 0.491851114 | 0.808341043 | 0.887979503 | 0.171589211 | 0.6484236 |
| 0.538253505 | 0.973311508 | 0.617180272 | 0.749412853 | 0.621379261 | 0.192228311 | 0.717457832 |
| 0.56252224 | 0.967048609 | 0.730980001 | 0.800654758 | 0.751619102 | 0.150309587 | 0.750338054 |
| 0.443740659 | 0.950537328 | 0.751974948 | 0.776101345 | 0.727279197 | 0.181125899 | 0.591416981 |
| 0.623016155 | 0.92349299 | 0.719379404 | 0.70422034 | 0.458188029 | 0.199914597 | 0.746993097 |
| 0.491708775 | 0.937015159 | 0.81901644 | 0.753967689 | 0.643939933 | 0.180058359 | 0.824994662 |
| 0.585438759 | 0.950466159 | 0.782506583 | 0.667568145 | 0.808341043 | 0.190093232 | 0.740801366 |
| 0.667212298 | 0.967689132 | 0.957298413 | 0.677958864 | 0.58529642 | 0.188883353 | 0.909614974 |
| 0.554764785 | 0.981495979 | 0.917514768 | 0.780513842 | 0.744146324 | 0.160771475 | 0.695395346 |
| 0.541029108 | 0.954593979 | 0.77353925 | 0.736388869 | 0.555974664 | 0.167674899 | 0.760728774 |
| 0.588000854 | 0.945982492 | 0.807487012 | 0.783502954 | 0.613692976 | 0.171873888 | 0.773895096 |
| 0.538538182 | 0.958152445 | 0.860650488 | 0.78905416 | 0.517472066 | 0.187886983 | 0.770692477 |
| 0.539961569 | 0.945413138 | 0.828410789 | 0.778165255 | 0.480464024 | 0.182833962 | 0.765141271 |
| 0.619742367 | 0.869190805 | 0.860508149 | 0.74144189 | 0.556828695 | 0.203473062 | 0.813678742 |
| 0.645576827 | 0.944843783 | 0.885346239 | 0.749839869 | 0.605366166 | 0.184257348 | 0.873389794 |
| 0.534339193 | 0.946338339 | 0.896235143 | 0.711764287 | 0.770194292 | 0.131876735 | 0.763290869 |
| 0.617749626 | 0.941569995 | 0.917443598 | 0.775887837 | 0.455625934 | 0.167888407 | 0.877588784 |
| 0.511209167 | 0.97103409 | 0.739093303 | 0.741868906 | 0.56985268 | 0.166038004 | 0.755604583 |
| 0.551135151 | 0.939577254 | 0.860935165 | 0.772187033 | 0.689203615 | 0.147747491 | 0.71041207 |
| 0.616397409 | 0.958081275 | 0.760017081 | 0.734609636 | 0.609351648 | 0.150096079 | 0.733826774 |
| 0.551846844 | 0.934097217 | 0.910753683 | 0.678670557 | 0.562806918 | 0.152088819 | 0.784499324 |
| 0.504732759 | 0.957511921 | 0.900007117 | 0.770550139 | 0.581453277 | 0.147320475 | 0.71866771 |
| 0.51498114 | 0.916731905 | 0.89765853 | 0.734609636 | 0.501316632 | 0.150594264 | 0.697459256 |

| 0.646786706 | 0.930823429 | 0.827983773 | 0.606149029 | 0.530211373 | 0.180912391 | 0.790548715 |
| --- | --- | --- | --- | --- | --- | --- |
| 0.558821436 | 0.909116789 | 0.804924916 | 0.604796812 | 0.555262971 | 0.214290798 | 0.750409224 |
| 0.523521458 | 0.928973027 | 0.74144189 | 0.837306953 | 0.502882357 | 0.186392428 | 0.815742652 |
| 0.511565013 | 0.929257704 | 0.817308377 | 0.81588499 | 0.550352288 | 0.174862999 | 0.723222546 |
| 0.473062415 | 0.970251228 | 0.921927265 | 0.744786848 | 0.484734183 | 0.219486158 | 0.754181197 |
| 0.541598463 | 0.961853249 | 0.838588001 | 0.781581382 | 0.579033521 | 0.164116433 | 0.866913387 |
| 0.491993452 | 0.956729058 | 0.858800085 | 0.782150737 | 0.534268024 | 0.193794036 | 0.671980642 |
| 0.549355918 | 0.957725429 | 0.721372144 | 0.748487652 | 0.589210732 | 0.185040211 | 0.783360615 |
| 0.500106754 | 0.951676037 | 0.809835599 | 0.751690271 | 0.482812611 | 0.183687994 | 0.806917657 |
| 0.400113871 | 0.961853249 | 0.78286243 | 0.762508007 | 0.684079425 | 0.203828909 | 0.795103551 |
| 0.470144474 | 0.975588926 | 0.802932176 | 0.711550779 | 0.480535193 | 0.204469433 | 0.778592271 |
| 0.459469077 | 0.947904064 | 0.804497901 | 0.800014234 | 0.64116433 | 0.20788556 | 0.701800584 |
| 0.657248594 | 0.890470429 | 0.749270515 | 0.592911537 | 0.498256352 | 0.210661163 | 0.775104975 |
| 0.550708135 | 0.933100847 | 0.738452779 | 0.633193367 | 0.639669774 | 0.196356131 | 0.791616255 |
| 0.599672621 | 0.893317202 | 0.691338695 | 0.821365027 | 0.603231087 | 0.174008967 | 0.814817451 |
| 0.527578108 | 0.938082699 | 0.875524874 | 0.848622874 | 0.829051313 | 0.170521671 | 0.711194933 |
| 0.577396627 | 0.939719593 | 0.8914668 | 0.721229806 | 0.608924632 | 0.162479539 | 0.841292435 |
| 0.600241976 | 0.945555476 | 0.888050673 | 0.699309658 | 0.521315209 | 0.211657533 | 0.845847271 |
| 0.54601096 | 0.964059498 | 0.909116789 | 0.641876023 | 0.665148388 | 0.16546865 | 0.764714255 |
| 0.470927336 | 0.964059498 | 0.745925557 | 0.793608996 | 0.446373924 | 0.196854316 | 0.815457975 |
| 0.643085901 | 0.944487937 | 0.848409366 | 0.687637891 | 0.33712903 | 0.289374422 | 0.787061419 |

| EMT3 | Fanconi anem | FGFR3-relate | Homologous r | Immune check | MicroRNAs i | Mismatch rep |
| --- | --- | --- | --- | --- | --- | --- |
| 0.346309871 | 0.812540033 | 0.707209451 | 0.812540033 | 0.419543093 | 0.5605295 | 0.575546224 |
| 0.377909046 | 0.801722297 | 0.654401822 | 0.801722297 | 0.287168173 | 0.565084336 | 0.545299267 |
| 0.510568643 | 0.714753398 | 0.905985339 | 0.714753398 | 0.572272436 | 0.56672123 | 0.616610917 |
| 0.543520034 | 0.670984272 | 0.728987261 | 0.670984272 | 0.592128674 | 0.512276706 | 0.496690627 |
| 0.719948758 | 0.643726425 | 0.475980357 | 0.643726425 | 0.43121486 | 0.508220056 | 0.4798235 |
| 0.827556758 | 0.613052452 | 0.208668422 | 0.613052452 | 0.738310441 | 0.564799658 | 0.512205537 |
| 0.409010035 | 0.671624795 | 0.64322824 | 0.671624795 | 0.510995659 | 0.695751192 | 0.558963775 |
| 0.352074585 | 0.670486086 | 0.41050459 | 0.670486086 | 0.422674543 | 0.563732119 | 0.554408939 |
| 0.546651484 | 0.76941143 | 0.717884848 | 0.76941143 | 0.649348801 | 0.602661732 | 0.622375632 |
| 0.536403103 | 0.850757953 | 0.77254288 | 0.850757953 | 0.387018718 | 0.648637108 | 0.580599246 |
| 0.347590919 | 0.756814462 | 0.731051171 | 0.756814462 | 0.348231443 | 0.490712405 | 0.38886912 |
| 0.547220838 | 0.66287097 | 0.637605864 | 0.66287097 | 0.588854886 | 0.600455484 | 0.60287524 |
| 0.530211373 | 0.852679525 | 0.750409224 | 0.852679525 | 0.671268949 | 0.648210092 | 0.467155363 |
| 0.606077859 | 0.792470287 | 0.845562593 | 0.792470287 | 0.490498897 | 0.572414775 | 0.367233649 |
| 0.578962351 | 0.804782578 | 0.583730695 | 0.804782578 | 0.309657676 | 0.472848908 | 0.499679738 |
| 0.549498256 | 0.776813038 | 0.774962636 | 0.776813038 | 0.649348801 | 0.607145399 | 0.502811188 |
| 0.440822717 | 0.908760942 | 0.903707921 | 0.908760942 | 0.547861362 | 0.537185965 | 0.529926696 |
| 0.868123265 | 0.810831969 | 0.710056224 | 0.810831969 | 0.662159277 | 0.573482314 | 0.609351648 |
| 0.289445591 | 0.888193011 | 0.557825066 | 0.888193011 | 0.396128389 | 0.702725785 | 0.704576187 |
| 0.288947406 | 0.928759519 | 0.797238631 | 0.928759519 | 0.373852395 | 0.551135151 | 0.485517045 |
| 0.279339549 | 0.619884706 | 0.431855384 | 0.619884706 | 0.425734823 | 0.54188314 | 0.586506298 |
| 0.418760231 | 0.605935521 | 0.424524945 | 0.605935521 | 0.74044552 | 0.537114796 | 0.492633976 |
| 0.444025336 | 0.579816383 | 0.659312504 | 0.579816383 | 0.647071383 | 0.520390008 | 0.394420326 |
| 0.62878087 | 0.86655754 | 0.75909188 | 0.86655754 | 0.408156003 | 0.622660309 | 0.69183688 |
| 0.619244182 | 0.601167177 | 0.786420895 | 0.601167177 | 0.38680521 | 0.526439399 | 0.632552843 |
| 0.43327877 | 0.856665006 | 0.961924418 | 0.856665006 | 0.620169383 | 0.527222262 | 0.46117714 |
| 0.806063625 | 0.789552345 | 0.730837663 | 0.789552345 | 0.433919294 | 0.531919436 | 0.550423457 |
| 0.674685076 | 0.851825493 | 0.581666785 | 0.851825493 | 0.411002776 | 0.570279695 | 0.613977653 |
| 0.800583588 | 0.901430503 | 0.759305388 | 0.901430503 | 0.414988257 | 0.583374849 | 0.548715394 |
| 0.493060992 | 0.7704078 | 0.818447086 | 0.7704078 | 0.397765284 | 0.526012383 | 0.497331151 |
| 0.299409295 | 0.638459896 | 0.569283325 | 0.638459896 | 0.534410362 | 0.527151092 | 0.499181553 |
| 0.454131379 | 0.641804854 | 0.303181268 | 0.641804854 | 0.311579247 | 0.652978436 | 0.627215145 |
| 0.393922141 | 0.846558964 | 0.788982991 | 0.846558964 | 0.453490855 | 0.608924632 | 0.566151875 |
| 0.525158352 | 0.885986763 | 0.830545869 | 0.885986763 | 0.490356558 | 0.560600669 | 0.585652267 |
| 0.278912533 | 0.546153299 | 0.221692406 | 0.546153299 | 0.80869689 | 0.670059071 | 0.656750409 |
| 0.567290584 | 0.514269447 | 0.579887552 | 0.514269447 | 0.434203971 | 0.593765568 | 0.598178066 |
| 0.2771333 | 0.790619885 | 0.324176215 | 0.790619885 | 0.488221479 | 0.598106896 | 0.574905701 |
| 0.26055085 | 0.722582023 | 0.460963632 | 0.722582023 | 0.449363035 | 0.658316134 | 0.626788129 |
| 0.339975802 | 0.704647356 | 0.571489574 | 0.704647356 | 0.324887908 | 0.519607145 | 0.447227955 |
| 0.716176785 | 0.822930752 | 0.666500605 | 0.822930752 | 0.513486585 | 0.607430076 | 0.63184115 |
| 0.14582592 | 0.898299053 | 0.132730766 | 0.898299053 | 0.527079923 | 0.468934595 | 0.469361611 |
| 0.126539036 | 0.724076578 | 0.190876094 | 0.724076578 | 0.489075511 | 0.702441107 | 0.729343107 |
| 0.557611558 | 0.655754039 | 0.691908049 | 0.655754039 | 0.546936161 | 0.583873034 | 0.407088463 |
| 0.281830475 | 0.562878087 | 0.542096648 | 0.562878087 | 0.335420966 | 0.590705288 | 0.625507081 |
| 0.354992527 | 0.462671696 | 0.655398192 | 0.462671696 | 0.229307523 | 0.475268664 | 0.420610633 |
| 0.325457263 | 0.76314853 | 0.572628283 | 0.76314853 | 0.478471283 | 0.484520675 | 0.485659384 |
| 0.274428866 | 0.791972102 | 0.272222618 | 0.791972102 | 0.532844637 | 0.631983489 | 0.72592698 |
| 0.571489574 | 0.727350367 | 0.737456409 | 0.727350367 | 0.419258416 | 0.492491638 | 0.54394705 |

| 0.54907124 | 0.854956943 | 0.655113515 | 0.854956943 | 0.508647071 | 0.642730055 | 0.423742082 |
| --- | --- | --- | --- | --- | --- | --- |
| 0.41157213 | 0.456622304 | 0.315707067 | 0.456622304 | 0.521386378 | 0.678528219 | 0.510283966 |
| 0.6959647 | 0.834744858 | 0.646003843 | 0.834744858 | 0.499110384 | 0.563233934 | 0.457476336 |
| 0.480321685 | 0.650772187 | 0.842359974 | 0.650772187 | 0.324460892 | 0.519535976 | 0.325670771 |
| 0.179346666 | 0.653974806 | 0.191089602 | 0.653974806 | 0.526439399 | 0.501885987 | 0.316062914 |
| 0.704006832 | 0.688420753 | 0.564159135 | 0.688420753 | 0.506369653 | 0.671482457 | 0.573553484 |
| 0.37748203 | 0.897872038 | 0.752615472 | 0.897872038 | 0.470642659 | 0.639598605 | 0.357056437 |
| 0.547576685 | 0.516902712 | 0.189381539 | 0.516902712 | 0.363390506 | 0.586577468 | 0.484663013 |
| 0.317770977 | 0.782008398 | 0.591915166 | 0.782008398 | 0.646146182 | 0.573553484 | 0.431997723 |
| 0.348658458 | 0.762650345 | 0.705145541 | 0.762650345 | 0.510568643 | 0.556686357 | 0.369866913 |
| 0.52330795 | 0.768770906 | 0.655825208 | 0.768770906 | 0.418689061 | 0.616183902 | 0.527578108 |
| 0.523521458 | 0.767845705 | 0.574407515 | 0.767845705 | 0.463596897 | 0.610490357 | 0.403174151 |
| 0.844993239 | 0.795459398 | 0.658814319 | 0.795459398 | 0.42089531 | 0.674685076 | 0.456835812 |
| 0.963276635 | 0.740516689 | 0.765141271 | 0.740516689 | 0.365525585 | 0.600668992 | 0.438901146 |
| 0.518681944 | 0.824709985 | 0.456764643 | 0.824709985 | 0.528574479 | 0.66593125 | 0.537114796 |
| 0.476976728 | 0.91317344 | 0.691979219 | 0.91317344 | 0.415486442 | 0.711550779 | 0.661020568 |
| 0.273361327 | 0.823784784 | 0.395559035 | 0.823784784 | 0.326595972 | 0.871325884 | 0.890470429 |
| 0.498896876 | 0.756529784 | 0.507223685 | 0.756529784 | 0.534054516 | 0.580599246 | 0.40331649 |
| 0.30609921 | 0.740303181 | 0.64941997 | 0.740303181 | 0.358266316 | 0.684719949 | 0.625364743 |
| 0.26581738 | 0.768486229 | 0.189452708 | 0.768486229 | 0.639527436 | 0.57291296 | 0.399758024 |
| 0.562451071 | 0.696534055 | 0.341185681 | 0.696534055 | 0.417479183 | 0.646929044 | 0.426233008 |
| 0.41363604 | 0.780656181 | 0.610703864 | 0.780656181 | 0.602661732 | 0.578748843 | 0.288235713 |
| 0.398192299 | 0.725001779 | 0.481460394 | 0.725001779 | 0.345882855 | 0.553839584 | 0.358622162 |
| 0.447085617 | 0.63810405 | 0.286954665 | 0.63810405 | 0.618674827 | 0.572770621 | 0.633335706 |
| 0.260408512 | 0.870614191 | 0.589993595 | 0.870614191 | 0.411856807 | 0.612767775 | 0.322325813 |
| 0.2905843 | 0.805707779 | 0.317343961 | 0.805707779 | 0.533129315 | 0.6287097 | 0.397907622 |
| 0.256636538 | 0.696320547 | 0.383317913 | 0.696320547 | 0.369582236 | 0.638815743 | 0.630987118 |
| 0.321258273 | 0.667069959 | 0.59362323 | 0.667069959 | 0.426304178 | 0.648992954 | 0.542238987 |
| 0.373211871 | 0.366664294 | 0.54907124 | 0.366664294 | 0.373069532 | 0.491139421 | 0.22745712 |
| 0.47050032 | 0.493630347 | 0.489004341 | 0.493630347 | 0.494413209 | 0.601238346 | 0.411500961 |
| 0.310582877 | 0.867411572 | 0.253220411 | 0.867411572 | 0.227030105 | 0.63803288 | 0.491424098 |
| 0.351291723 | 0.765497118 | 0.734965483 | 0.765497118 | 0.624083695 | 0.740374351 | 0.743648139 |
| 0.465091453 | 0.846487794 | 0.591701658 | 0.846487794 | 0.453846701 | 0.60187887 | 0.413280194 |
| 0.420610633 | 0.550636965 | 0.489431357 | 0.550636965 | 0.433421109 | 0.587289161 | 0.452850331 |
| 0.397409437 | 0.60493915 | 0.588712547 | 0.60493915 | 0.439684008 | 0.611059711 | 0.398619315 |
| 0.338410078 | 0.580243399 | 0.478257775 | 0.580243399 | 0.590634119 | 0.664792541 | 0.360472564 |
| 0.298982279 | 0.827983773 | 0.621806277 | 0.827983773 | 0.323179845 | 0.745142694 | 0.714184044 |
| 0.601807701 | 0.853675895 | 0.661234076 | 0.853675895 | 0.711906626 | 0.687282044 | 0.612340759 |
| 0.59362323 | 0.82627571 | 0.616682087 | 0.82627571 | 0.618247812 | 0.64223187 | 0.572414775 |
| 0.526297061 | 0.735178991 | 0.570279695 | 0.735178991 | 0.455198918 | 0.599672621 | 0.684364102 |
| 0.277773824 | 0.889474059 | 0.641804854 | 0.889474059 | 0.333285887 | 0.758949541 | 0.519962992 |
| 0.407301971 | 0.84385453 | 0.438118283 | 0.84385453 | 0.619742367 | 0.676535478 | 0.52224041 |
| 0.354280834 | 0.889758736 | 0.239484734 | 0.889758736 | 0.573624653 | 0.682584869 | 0.451711622 |
| 0.735819515 | 0.8645648 | 0.576756103 | 0.8645648 | 0.677958864 | 0.70735179 | 0.60081133 |
| 0.475909188 | 0.900505302 | 0.776172514 | 0.900505302 | 0.36089958 | 0.732332218 | 0.65973952 |
| 0.480677532 | 0.794534197 | 0.575403886 | 0.794534197 | 0.775318483 | 0.606433706 | 0.45085759 |
| 0.246530496 | 0.73425379 | 0.696534055 | 0.73425379 | 0.425450146 | 0.60294641 | 0.366877802 |
| 0.477688421 | 0.799516049 | 0.752686642 | 0.799516049 | 0.487367447 | 0.600028468 | 0.435058003 |
| 0.45605295 | 0.563732119 | 0.56565369 | 0.563732119 | 0.337698384 | 0.563447441 | 0.259269803 |
| 0.243897232 | 0.801010604 | 0.573482314 | 0.801010604 | 0.545299267 | 0.59155932 | 0.433776955 |

| 0.241548644 | 0.48395132 | 0.570279695 | 0.48395132 | 0.504519251 | 0.512063198 | 0.23891538 |
| --- | --- | --- | --- | --- | --- | --- |
| 0.775318483 | 0.701871753 | 0.557469219 | 0.701871753 | 0.527222262 | 0.578250658 | 0.490498897 |
| 0.203828909 | 0.648494769 | 0.571489574 | 0.648494769 | 0.317415131 | 0.574905701 | 0.228026475 |
| 0.236282115 | 0.924987545 | 0.536403103 | 0.924987545 | 0.497544659 | 0.608212939 | 0.446160416 |
| 0.415272934 | 0.793537826 | 0.687139705 | 0.793537826 | 0.6825137 | 0.602448224 | 0.399402178 |
| 0.37741086 | 0.650060494 | 0.509074087 | 0.650060494 | 0.665860081 | 0.593196214 | 0.278272009 |
| 0.442886627 | 0.916020212 | 0.738879795 | 0.916020212 | 0.384385453 | 0.688990107 | 0.62664579 |
| 0.475339833 | 0.71453989 | 0.608141769 | 0.71453989 | 0.541171447 | 0.629350224 | 0.446231585 |
| 0.341826205 | 0.850686784 | 0.608568785 | 0.850686784 | 0.363390506 | 0.579674045 | 0.390150167 |
| 0.643014732 | 0.796811615 | 0.403814675 | 0.796811615 | 0.278983702 | 0.655469362 | 0.409721728 |
| 0.378264892 | 0.855099281 | 0.791972102 | 0.855099281 | 0.447512633 | 0.730339478 | 0.438616469 |
| 0.355419543 | 0.806988826 | 0.661020568 | 0.806988826 | 0.531207743 | 0.634474415 | 0.390221337 |
| 0.473062415 | 0.752615472 | 0.611629066 | 0.752615472 | 0.585652267 | 0.627855669 | 0.438972315 |
| 0.617037933 | 0.879225678 | 0.654971176 | 0.879225678 | 0.333570564 | 0.623941356 | 0.566294214 |
| 0.49014305 | 0.855312789 | 0.839442033 | 0.855312789 | 0.457547506 | 0.727279197 | 0.527008754 |
| 0.452423315 | 0.890683937 | 0.650701018 | 0.890683937 | 0.665717743 | 0.696178208 | 0.525941214 |
| 0.489217849 | 0.827485588 | 0.894455911 | 0.827485588 | 0.457618675 | 0.656963917 | 0.536972457 |
| 0.343747776 | 0.802932176 | 0.301117358 | 0.802932176 | 0.590562949 | 0.672905843 | 0.410860437 |
| 0.427015871 | 0.660095367 | 0.132446089 | 0.660095367 | 0.28745285 | 0.646573198 | 0.464095082 |
| 0.718169525 | 0.865490001 | 0.454700733 | 0.865490001 | 0.368230019 | 0.562308732 | 0.382179204 |
| 0.857590207 | 0.887338979 | 0.660593552 | 0.887338979 | 0.747277774 | 0.681232652 | 0.583944203 |
| 0.425378977 | 0.374919935 | 0.564230304 | 0.374919935 | 0.520959362 | 0.553768415 | 0.184969041 |
| 0.354423173 | 0.752473134 | 0.330154437 | 0.752473134 | 0.479965839 | 0.583944203 | 0.47775959 |
| 0.400612056 | 0.536901288 | 0.824140631 | 0.536901288 | 0.381681019 | 0.506084976 | 0.244608925 |
| 0.319407871 | 0.838089816 | 0.509714611 | 0.838089816 | 0.586719806 | 0.591630489 | 0.510212796 |
| 0.431855384 | 0.705430218 | 0.354209665 | 0.705430218 | 0.343890115 | 0.644793965 | 0.306668565 |
| 0.398049961 | 0.817664223 | 0.528503309 | 0.817664223 | 0.551704505 | 0.536545442 | 0.306241549 |
| 0.510070458 | 0.888904704 | 0.652480251 | 0.888904704 | 0.369297559 | 0.606362536 | 0.57191659 |
| 0.443028966 | 0.789552345 | 0.422247527 | 0.789552345 | 0.455697103 | 0.679026404 | 0.57803715 |
| 0.360187887 | 0.779161626 | 0.734396128 | 0.779161626 | 0.463739236 | 0.698384457 | 0.572557113 |
| 0.629563732 | 0.820510996 | 0.70322397 | 0.820510996 | 0.610703864 | 0.640594975 | 0.506867839 |
| 0.531990606 | 0.672122981 | 0.320333072 | 0.672122981 | 0.308234289 | 0.801864636 | 0.683011885 |
| 0.283467369 | 0.780513842 | 0.57704078 | 0.780513842 | 0.32680948 | 0.613123621 | 0.651555049 |
| 0.317201623 | 0.43747776 | 0.543306526 | 0.43747776 | 0.517187389 | 0.535762579 | 0.265248025 |
| 0.31442602 | 0.882001281 | 0.362394136 | 0.882001281 | 0.749911038 | 0.501672479 | 0.326097787 |
| 0.729485446 | 0.805494271 | 0.329158067 | 0.805494271 | 0.242900861 | 0.756885631 | 0.633264536 |
| 0.603373425 | 0.490356558 | 0.706141912 | 0.490356558 | 0.407728987 | 0.597822219 | 0.506511992 |
| 0.419543093 | 0.779303964 | 0.668849192 | 0.779303964 | 0.530567219 | 0.744075155 | 0.691125187 |
| 0.624226034 | 0.662942139 | 0.821151519 | 0.662942139 | 0.330154437 | 0.651341542 | 0.542452494 |
| 0.609280478 | 0.827983773 | 0.412710839 | 0.827983773 | 0.559248452 | 0.60287524 | 0.425521315 |
| 0.284534909 | 0.397978792 | 0.401893104 | 0.397978792 | 0.349868337 | 0.465874315 | 0.280905274 |
| 0.394206818 | 0.772044694 | 0.665860081 | 0.772044694 | 0.328161697 | 0.616255071 | 0.411287453 |
| 0.18404384 | 0.861148673 | 0.403814675 | 0.861148673 | 0.403174151 | 0.716959647 | 0.729627784 |
| 0.451213437 | 0.875453704 | 0.717030816 | 0.875453704 | 0.491495267 | 0.580528076 | 0.334424596 |
| 0.330225607 | 0.683296563 | 0.164970465 | 0.683296563 | 0.495836595 | 0.688207245 | 0.693758451 |
| 0.384171945 | 0.727065689 | 0.540459754 | 0.727065689 | 0.407301971 | 0.697957441 | 0.434275141 |
| 0.456408797 | 0.899864778 | 0.463596897 | 0.899864778 | 0.398903993 | 0.669560885 | 0.6080706 |
| 0.531990606 | 0.669418547 | 0.423813252 | 0.669418547 | 0.298341755 | 0.546793823 | 0.543804711 |
| 0.465589638 | 0.800156572 | 0.724645933 | 0.800156572 | 0.289303252 | 0.511493844 | 0.455910611 |
| 0.301402035 | 0.79218561 | 0.202476692 | 0.79218561 | 0.25435912 | 0.734538467 | 0.595260124 |

| 0.428439257 | 0.755248737 | 0.672977012 | 0.755248737 | 0.492491638 | 0.546651484 | 0.338338908 |
| --- | --- | --- | --- | --- | --- | --- |
| 0.458828553 | 0.714824568 | 0.401181411 | 0.714824568 | 0.545583944 | 0.5874315 | 0.42089531 |
| 0.315849406 | 0.594833108 | 0.601807701 | 0.594833108 | 0.657889118 | 0.689132446 | 0.415984627 |
| 0.434488648 | 0.775959006 | 0.788911821 | 0.775959006 | 0.295352644 | 0.672265319 | 0.34638104 |
| 0.500960786 | 0.822859583 | 0.606362536 | 0.822859583 | 0.373211871 | 0.663369155 | 0.470784998 |
| 0.554408939 | 0.786065049 | 0.352288093 | 0.786065049 | 0.70422034 | 0.620667568 | 0.476905558 |
| 0.287595189 | 0.630702441 | 0.515835172 | 0.630702441 | 0.307949612 | 0.61426233 | 0.453063839 |
| 0.98548146 | 0.520105331 | 0.323606861 | 0.520105331 | 0.378051384 | 0.59049178 | 0.391644723 |
| 0.553341399 | 0.889545228 | 0.784143477 | 0.889545228 | 0.737100562 | 0.583801865 | 0.483026119 |
| 0.23265248 | 0.890897445 | 0.399829194 | 0.890897445 | 0.389936659 | 0.710198562 | 0.568856309 |
| 0.777524731 | 0.737741086 | 0.578392997 | 0.737741086 | 0.373211871 | 0.719521742 | 0.506583161 |
| 0.635257277 | 0.862856736 | 0.731335848 | 0.862856736 | 0.337342538 | 0.710127393 | 0.670201409 |
| 0.57597324 | 0.814959789 | 0.562379902 | 0.814959789 | 0.326168956 | 0.641021991 | 0.462315849 |
| 0.794463028 | 0.871112376 | 0.663796171 | 0.871112376 | 0.614760515 | 0.622162124 | 0.544587574 |
| 0.506227315 | 0.737029393 | 0.692762081 | 0.737029393 | 0.425450146 | 0.722582023 | 0.506013807 |
| 0.548217209 | 0.454416056 | 0.47057149 | 0.454416056 | 0.442673119 | 0.599103267 | 0.481531564 |
| 0.354565511 | 0.904704292 | 0.582734325 | 0.904704292 | 0.344032453 | 0.620952245 | 0.41676749 |
| 0.755533414 | 0.906625863 | 0.658814319 | 0.906625863 | 0.648708277 | 0.698669134 | 0.645220981 |
| 0.579816383 | 0.583730695 | 0.087253576 | 0.583730695 | 0.391004199 | 0.670272578 | 0.526795246 |
| 0.38267739 | 0.602590563 | 0.257846417 | 0.602590563 | 0.429862643 | 0.587146822 | 0.479325315 |
| 0.574621023 | 0.757383816 | 0.961995588 | 0.757383816 | 0.687495552 | 0.538965198 | 0.295210305 |
| 0.387516903 | 0.767134012 | 0.741228382 | 0.767134012 | 0.421535834 | 0.680805637 | 0.456337627 |
| 0.467938225 | 0.864351292 | 0.641520176 | 0.864351292 | 0.406305601 | 0.761369298 | 0.551562166 |
| 0.436196712 | 0.595260124 | 0.775033805 | 0.595260124 | 0.55433777 | 0.512988399 | 0.295494983 |
| 0.356842929 | 0.821934382 | 0.533841008 | 0.821934382 | 0.482670273 | 0.627428653 | 0.44779731 |
| 0.667069959 | 0.829478329 | 0.275354067 | 0.829478329 | 0.286314141 | 0.739306811 | 0.793395488 |
| 0.336986691 | 0.663226817 | 0.646502028 | 0.663226817 | 0.734396128 | 0.593409722 | 0.471852537 |
| 0.869119636 | 0.740303181 | 0.604725642 | 0.740303181 | 0.378691908 | 0.722012668 | 0.650060494 |
| 0.388228596 | 0.793822504 | 0.778592271 | 0.793822504 | 0.397409437 | 0.688634261 | 0.474201124 |
| 0.334139919 | 0.81389225 | 0.701729414 | 0.81389225 | 0.601024838 | 0.726852181 | 0.632339335 |
| 0.605081489 | 0.774179774 | 0.695039499 | 0.774179774 | 0.643441748 | 0.616610917 | 0.416411643 |
| 0.684292933 | 0.797096292 | 0.650558679 | 0.797096292 | 0.550209949 | 0.488862003 | 0.36403103 |
| 0.368728204 | 0.590064764 | 0.788484805 | 0.590064764 | 0.571347235 | 0.608355277 | 0.353213294 |
| 0.368585866 | 0.757882001 | 0.696035869 | 0.757882001 | 0.454060209 | 0.591132304 | 0.458615045 |
| 0.687424383 | 0.46537613 | 0.466016654 | 0.46537613 | 0.675539108 | 0.563162764 | 0.350793538 |
| 0.347875596 | 0.736673546 | 0.420112448 | 0.736673546 | 0.505302114 | 0.609422817 | 0.416411643 |
| 0.477617251 | 0.705359049 | 0.598391574 | 0.705359049 | 0.328588713 | 0.625293573 | 0.441036225 |
| 0.717600171 | 0.882570636 | 0.608426447 | 0.882570636 | 0.341683866 | 0.601807701 | 0.471852537 |
| 0.664650203 | 0.778165255 | 0.270514554 | 0.778165255 | 0.578891182 | 0.636467155 | 0.467582378 |
| 0.530282542 | 0.770550139 | 0.893246032 | 0.770550139 | 0.41057576 | 0.66287097 | 0.597964558 |
| 0.784001139 | 0.931890969 | 0.787132588 | 0.931890969 | 0.487794463 | 0.609209309 | 0.486513415 |
| 0.369795744 | 0.690627002 | 0.663369155 | 0.690627002 | 0.616397409 | 0.580812754 | 0.335634474 |
| 0.537470643 | 0.599459113 | 0.596398833 | 0.599459113 | 0.447868479 | 0.633122198 | 0.49640595 |
| 0.284677247 | 0.770478969 | 0.389936659 | 0.770478969 | 0.562806918 | 0.69283325 | 0.537826489 |
| 0.401039072 | 0.664863711 | 0.847128318 | 0.664863711 | 0.378193723 | 0.577325457 | 0.389011458 |
| 0.445519892 | 0.789125329 | 0.901074657 | 0.789125329 | 0.434061633 | 0.715038076 | 0.692762081 |
| 0.417408014 | 0.816169668 | 0.692762081 | 0.816169668 | 0.486655754 | 0.645434489 | 0.515835172 |
| 0.373496548 | 0.803643869 | 0.52743577 | 0.803643869 | 0.312219771 | 0.583019002 | 0.486513415 |
| 0.343961284 | 0.572272436 | 0.583374849 | 0.572272436 | 0.581737955 | 0.62458188 | 0.440893887 |
| 0.53675895 | 0.543875881 | 0.49533841 | 0.543875881 | 0.345882855 | 0.562379902 | 0.424169098 |

| 0.479609992 | 0.746067896 | 0.824709985 | 0.746067896 | 0.421251157 | 0.730552986 | 0.393068109 |
| --- | --- | --- | --- | --- | --- | --- |
| 0.283823215 | 0.855455128 | 0.652124404 | 0.855455128 | 0.402889474 | 0.597324034 | 0.339121771 |
| 0.371147961 | 0.976514127 | 0.736602377 | 0.976514127 | 0.279695395 | 0.550708135 | 0.456337627 |
| 0.4394705 | 0.82727208 | 0.634047399 | 0.82727208 | 0.514838802 | 0.595829478 | 0.454344886 |
| 0.43847413 | 0.754323536 | 0.582805494 | 0.754323536 | 0.573411145 | 0.584371219 | 0.39498968 |
| 0.130595687 | 0.814461604 | 0.732688065 | 0.814461604 | 0.602590563 | 0.422745712 | 0.392569924 |
| 0.212938581 | 0.722083837 | 0.500960786 | 0.722083837 | 0.541242616 | 0.634616753 | 0.538324674 |
| 0.23058857 | 0.850900292 | 0.406590278 | 0.850900292 | 0.448295495 | 0.547932531 | 0.581951463 |
| 0.222831115 | 0.725428795 | 0.59362323 | 0.725428795 | 0.419614262 | 0.505800299 | 0.51085332 |
| 0.222546438 | 0.85310654 | 0.633478044 | 0.85310654 | 0.581026261 | 0.48395132 | 0.571418404 |
| 0.23372002 | 0.855882144 | 0.498256352 | 0.855882144 | 0.420681802 | 0.516119849 | 0.563803288 |
| 0.199772258 | 0.913244609 | 0.124688634 | 0.913244609 | 0.385808839 | 0.54494342 | 0.694043129 |
| 0.398832823 | 0.904348445 | 0.791829763 | 0.904348445 | 0.51291723 | 0.425948331 | 0.531065405 |
| 0.36189595 | 0.89246317 | 0.148815031 | 0.89246317 | 0.50779304 | 0.647142552 | 0.629350224 |
| 0.165824496 | 0.860792826 | 0.307878443 | 0.860792826 | 0.498683368 | 0.555405309 | 0.623229663 |
| 0.167390221 | 0.780585012 | 0.629350224 | 0.780585012 | 0.509429934 | 0.523663796 | 0.492705146 |
| 0.214788983 | 0.858657747 | 0.64016796 | 0.858657747 | 0.565440182 | 0.436339051 | 0.516119849 |
| 0.492633976 | 0.902996228 | 0.602163547 | 0.902996228 | 0.497473489 | 0.541242616 | 0.542452494 |
| 0.220126681 | 0.84179062 | 0.822361398 | 0.84179062 | 0.605722013 | 0.473418262 | 0.504234574 |
| 0.203259554 | 0.662301616 | 0.688563092 | 0.662301616 | 0.457262828 | 0.521386378 | 0.451996299 |
| 0.301188528 | 0.898227884 | 0.607643584 | 0.898227884 | 0.428510426 | 0.51910896 | 0.591630489 |
| 0.620169383 | 0.91723009 | 0.633122198 | 0.91723009 | 0.778948118 | 0.480250516 | 0.57803715 |
| 0.292007686 | 0.913031101 | 0.870329514 | 0.913031101 | 0.555618817 | 0.404953384 | 0.440039855 |
| 0.211728703 | 0.791402747 | 0.292292364 | 0.791402747 | 0.609209309 | 0.428296918 | 0.459469077 |
| 0.431570707 | 0.95551918 | 0.664721372 | 0.95551918 | 0.676108462 | 0.502384172 | 0.649989325 |
| 0.204255925 | 0.943420397 | 0.683865917 | 0.943420397 | 0.571276066 | 0.561241193 | 0.608426447 |
| 0.23884421 | 0.913813963 | 0.664792541 | 0.913813963 | 0.655327023 | 0.639527436 | 0.553199061 |
| 0.152515835 | 0.910611344 | 0.753967689 | 0.910611344 | 0.658600811 | 0.378122554 | 0.412141485 |
| 0.308305459 | 0.919649847 | 0.768557398 | 0.919649847 | 0.586150452 | 0.415344104 | 0.551704505 |
| 0.260052665 | 0.767062843 | 0.537826489 | 0.767062843 | 0.48395132 | 0.412283823 | 0.528574479 |
| 0.869973667 | 0.80869689 | 0.606931891 | 0.80869689 | 0.646217351 | 0.495480749 | 0.474414632 |
| 0.468294072 | 0.566365383 | 0.596185325 | 0.566365383 | 0.505088606 | 0.312077432 | 0.495409579 |
| 0.370151591 | 0.897516191 | 0.848195858 | 0.897516191 | 0.547576685 | 0.429791474 | 0.502811188 |
| 0.23784784 | 0.883567006 | 0.775887837 | 0.883567006 | 0.470215643 | 0.48295495 | 0.619386521 |
| 0.273717173 | 0.772329372 | 0.413280194 | 0.772329372 | 0.398192299 | 0.416696321 | 0.563162764 |
| 0.120204968 | 0.967760302 | 0.837022276 | 0.967760302 | 0.60700306 | 0.509928119 | 0.456195289 |
| 0.747775959 | 0.867482741 | 0.663226817 | 0.867482741 | 0.510141627 | 0.410646929 | 0.410219913 |
| 0.130382179 | 0.774108604 | 0.339691125 | 0.774108604 | 0.571631912 | 0.588854886 | 0.636680663 |
| 0.318055654 | 0.905060138 | 0.782079567 | 0.905060138 | 0.437975945 | 0.467582378 | 0.433705786 |
| 0.145968258 | 0.844566223 | 0.775959006 | 0.844566223 | 0.411714469 | 0.298697602 | 0.409935236 |
| 0.255141983 | 0.885417408 | 0.609921002 | 0.885417408 | 0.598533912 | 0.408298342 | 0.472777738 |
| 0.414205395 | 0.927834318 | 0.74564088 | 0.927834318 | 0.538039997 | 0.468934595 | 0.623514341 |
| 0.39605722 | 0.905843 | 0.70108889 | 0.905843 | 0.819585795 | 0.406092093 | 0.599886129 |
| 0.233933528 | 0.899295424 | 0.853462387 | 0.899295424 | 0.677958864 | 0.372642517 | 0.430930183 |
| 0.223258131 | 0.946551847 | 0.754038858 | 0.946551847 | 0.362678813 | 0.509999288 | 0.672834674 |
| 0.204184756 | 0.790406377 | 0.550708135 | 0.790406377 | 0.643441748 | 0.327734681 | 0.492562807 |
| 0.337413707 | 0.925841577 | 0.734823144 | 0.925841577 | 0.462742865 | 0.467582378 | 0.56565369 |
| 0.244608925 | 0.84385453 | 0.662443954 | 0.84385453 | 0.636894171 | 0.487794463 | 0.599672621 |
| 0.404028183 | 0.734324959 | 0.498256352 | 0.734324959 | 0.663084478 | 0.408938866 | 0.527791616 |
| 0.288378051 | 0.893744217 | 0.735107822 | 0.893744217 | 0.52224041 | 0.49227813 | 0.485659384 |

| 0.305245178 | 0.79318198 | 0.710554409 | 0.79318198 | 0.749199345 | 0.40018504 | 0.654971176 |
| --- | --- | --- | --- | --- | --- | --- |
| 0.161981354 | 0.860223472 | 0.860863995 | 0.860223472 | 0.368087681 | 0.428795104 | 0.490427728 |
| 0.285531279 | 0.841434773 | 0.517614405 | 0.841434773 | 0.505159775 | 0.599459113 | 0.606647214 |
| 0.28752402 | 0.864422461 | 0.628425023 | 0.864422461 | 0.561810547 | 0.581880293 | 0.585936944 |
| 0.21300975 | 0.767561028 | 0.611415558 | 0.767561028 | 0.62251797 | 0.416340474 | 0.528218632 |
| 0.253078073 | 0.898014376 | 0.797736816 | 0.898014376 | 0.500960786 | 0.402462458 | 0.5273646 |
| 0.519820653 | 0.91103836 | 0.683937086 | 0.91103836 | 0.577610135 | 0.445519892 | 0.499822077 |
| 0.486726923 | 0.875097858 | 0.650629848 | 0.875097858 | 0.494840225 | 0.456479966 | 0.478400114 |
| 0.271083909 | 0.937228667 | 0.694327806 | 0.937228667 | 0.779446303 | 0.457476336 | 0.525087182 |
| 0.366450786 | 0.945697815 | 0.745783218 | 0.945697815 | 0.589566579 | 0.52330795 | 0.619030674 |
| 0.181268237 | 0.889474059 | 0.706640097 | 0.889474059 | 0.793680165 | 0.385097146 | 0.547505516 |
| 0.218987972 | 0.93174863 | 0.776813038 | 0.93174863 | 0.565226674 | 0.446302754 | 0.514127108 |
| 0.306455057 | 0.888620027 | 0.836381752 | 0.888620027 | 0.538039997 | 0.466657177 | 0.419756601 |
| 0.184613195 | 0.812468863 | 0.149384385 | 0.812468863 | 0.532275283 | 0.395416696 | 0.440395701 |
| 0.169027116 | 0.856309159 | 0.185680734 | 0.856309159 | 0.452565654 | 0.414845918 | 0.496690627 |
| 0.180770052 | 0.823500107 | 0.29471212 | 0.823500107 | 0.437264252 | 0.474983987 | 0.594192584 |
| 0.362607644 | 0.905060138 | 0.497544659 | 0.905060138 | 0.492420468 | 0.300476834 | 0.437121913 |
| 0.226176073 | 0.902853889 | 0.751547933 | 0.902853889 | 0.429506797 | 0.552985553 | 0.4322824 |
| 0.234075866 | 0.929898228 | 0.700661875 | 0.929898228 | 0.630417764 | 0.507010177 | 0.464450929 |
| 0.273574835 | 0.923920006 | 0.549498256 | 0.923920006 | 0.535833748 | 0.381752188 | 0.530282542 |
| 0.42815458 | 0.753113657 | 0.59148815 | 0.753113657 | 0.762508007 | 0.407586649 | 0.486584585 |
| 0.252437549 | 0.779659811 | 0.670486086 | 0.779659811 | 0.497758167 | 0.37022276 | 0.559960145 |
| 0.271155078 | 0.741726568 | 0.429435627 | 0.741726568 | 0.449149527 | 0.475197495 | 0.709913885 |
| 0.167603729 | 0.771902356 | 0.699309658 | 0.771902356 | 0.50259768 | 0.539890399 | 0.598035727 |
| 0.223898655 | 0.704433848 | 0.249377269 | 0.704433848 | 0.633549214 | 0.4125685 | 0.453063839 |
| 0.175717031 | 0.931321614 | 0.456408797 | 0.931321614 | 0.55220269 | 0.44160558 | 0.426161839 |
| 0.184186179 | 0.807842858 | 0.840509572 | 0.807842858 | 0.493915024 | 0.429364458 | 0.494840225 |
| 0.241335136 | 0.923350651 | 0.555049463 | 0.923350651 | 0.494911394 | 0.509074087 | 0.546224468 |
| 0.116788841 | 0.876805921 | 0.202049676 | 0.876805921 | 0.776884208 | 0.403529998 | 0.488007971 |
| 0.259483311 | 0.920646217 | 0.622944986 | 0.920646217 | 0.484734183 | 0.493701516 | 0.671838303 |
| 0.199843428 | 0.805494271 | 0.800725927 | 0.805494271 | 0.512134368 | 0.534695039 | 0.40232012 |
| 0.184969041 | 0.884776884 | 0.659953028 | 0.884776884 | 0.437121913 | 0.543520034 | 0.551633336 |
| 0.890826276 | 0.926268593 | 0.627286314 | 0.926268593 | 0.685075795 | 0.520674685 | 0.442317273 |
| 0.376556829 | 0.82001281 | 0.504234574 | 0.82001281 | 0.586008113 | 0.486584585 | 0.515265817 |
| 0.262472422 | 0.907266387 | 0.706711266 | 0.907266387 | 0.556117002 | 0.452779162 | 0.480037008 |
| 0.496263611 | 0.891182122 | 0.8717529 | 0.891182122 | 0.49021422 | 0.544302897 | 0.623087325 |
| 0.329158067 | 0.634047399 | 0.872962778 | 0.634047399 | 0.406874956 | 0.340402818 | 0.399829194 |
| 0.586079283 | 0.785353356 | 0.988328233 | 0.785353356 | 0.479396484 | 0.39918867 | 0.43327877 |
| 0.327663511 | 0.643797594 | 0.631627642 | 0.643797594 | 0.467155363 | 0.424667283 | 0.440111024 |
| 0.349725998 | 0.84079425 | 0.815315636 | 0.84079425 | 0.487153939 | 0.516119849 | 0.486442246 |
| 0.341114511 | 0.811614832 | 0.804284393 | 0.811614832 | 0.469930966 | 0.540459754 | 0.557825066 |
| 0.359618532 | 0.784356985 | 0.713116504 | 0.784356985 | 0.380257633 | 0.469005765 | 0.412853178 |
| 0.289160914 | 0.785780372 | 0.647284891 | 0.785780372 | 0.405736247 | 0.503309373 | 0.435698527 |
| 0.739876165 | 0.842217636 | 0.366664294 | 0.842217636 | 0.399829194 | 0.416055797 | 0.458472707 |
| 0.354565511 | 0.796811615 | 0.928474842 | 0.796811615 | 0.393352786 | 0.447441463 | 0.420397125 |
| 0.201978507 | 0.716034446 | 0.822005551 | 0.716034446 | 0.425236638 | 0.469432781 | 0.348871966 |
| 0.565297844 | 0.802006975 | 0.358693331 | 0.802006975 | 0.43334994 | 0.477474913 | 0.422389865 |
| 0.355277204 | 0.898299053 | 0.869261974 | 0.898299053 | 0.409650559 | 0.452067469 | 0.369012882 |
| 0.266956089 | 0.739235642 | 0.948117572 | 0.739235642 | 0.449220696 | 0.482314426 | 0.366237278 |
| 0.26574621 | 0.826133371 | 0.818589424 | 0.826133371 | 0.454416056 | 0.665006049 | 0.563945627 |

| 0.336488506 | 0.804640239 | 0.871895239 | 0.804640239 | 0.536189595 | 0.590278272 | 0.462102341 |
| --- | --- | --- | --- | --- | --- | --- |
| 0.451996299 | 0.921998434 | 0.904134937 | 0.921998434 | 0.393281617 | 0.533271653 | 0.525229521 |
| 0.215714184 | 0.868977297 | 0.711977795 | 0.868977297 | 0.452423315 | 0.568002277 | 0.51498114 |
| 0.390506014 | 0.717386663 | 0.586577468 | 0.717386663 | 0.384029606 | 0.424169098 | 0.470357982 |
| 0.274215358 | 0.871397054 | 0.563945627 | 0.871397054 | 0.421607003 | 0.664294356 | 0.636538325 |
| 0.252224041 | 0.742438261 | 0.736104192 | 0.742438261 | 0.352999786 | 0.585011743 | 0.455768273 |
| 0.266742581 | 0.77873461 | 0.827983773 | 0.77873461 | 0.721514483 | 0.449078357 | 0.469859796 |
| 0.749484022 | 0.874457334 | 0.890185752 | 0.874457334 | 0.479325315 | 0.535406733 | 0.571489574 |
| 0.323606861 | 0.775104975 | 0.279980073 | 0.775104975 | 0.394847342 | 0.574122838 | 0.67319052 |
| 0.455981781 | 0.672265319 | 0.716888478 | 0.672265319 | 0.409294712 | 0.52323678 | 0.459326738 |
| 0.365667924 | 0.787061419 | 0.844779731 | 0.787061419 | 0.414205395 | 0.483097288 | 0.546366807 |
| 0.301544374 | 0.828980144 | 0.53875169 | 0.828980144 | 0.384100776 | 0.483595474 | 0.516902712 |
| 0.44160558 | 0.754252366 | 0.83346381 | 0.754252366 | 0.372927194 | 0.577467796 | 0.483453135 |
| 0.333357056 | 0.90691054 | 0.86869262 | 0.90691054 | 0.406732617 | 0.709131023 | 0.62045406 |
| 0.334353427 | 0.81695253 | 0.666215928 | 0.81695253 | 0.37954594 | 0.481531564 | 0.49427087 |
| 0.430645506 | 0.816383176 | 0.606433706 | 0.816383176 | 0.434203971 | 0.415059426 | 0.422176358 |
| 0.389438474 | 0.776314853 | 0.774891467 | 0.776314853 | 0.440893887 | 0.387659241 | 0.475766849 |
| 0.490854743 | 0.758237848 | 0.711550779 | 0.758237848 | 0.410789268 | 0.440751548 | 0.408298342 |
| 0.348373781 | 0.813749911 | 0.79211444 | 0.813749911 | 0.396128389 | 0.431713045 | 0.419970109 |
| 0.522952103 | 0.86449363 | 0.767703366 | 0.86449363 | 0.39918867 | 0.33819657 | 0.435485019 |
| 0.340901003 | 0.782435414 | 0.624297203 | 0.782435414 | 0.462671696 | 0.612625436 | 0.513771262 |
| 0.437121913 | 0.645149811 | 0.891182122 | 0.645149811 | 0.460678955 | 0.349298982 | 0.377837876 |
| 0.362038289 | 0.722510853 | 0.772827557 | 0.722510853 | 0.478613622 | 0.398832823 | 0.346309871 |
| 0.565582521 | 0.668849192 | 0.865703509 | 0.668849192 | 0.351932247 | 0.538182336 | 0.307664935 |
| 0.288733898 | 0.821507366 | 0.744217493 | 0.821507366 | 0.4457334 | 0.44260195 | 0.419116077 |
| 0.400113871 | 0.92242545 | 0.774464451 | 0.92242545 | 0.447868479 | 0.412354993 | 0.536403103 |
| 0.330581453 | 0.583944203 | 0.382463882 | 0.583944203 | 0.455270088 | 0.419044908 | 0.365667924 |
| 0.443527151 | 0.764287239 | 0.712618319 | 0.764287239 | 0.388086257 | 0.434488648 | 0.363176998 |
| 0.225962565 | 0.590847627 | 0.624724219 | 0.590847627 | 0.335492136 | 0.400825564 | 0.455910611 |
| 0.29471212 | 0.669632055 | 0.624795388 | 0.669632055 | 0.501743648 | 0.383175575 | 0.368087681 |
| 0.248309729 | 0.685431642 | 0.789979361 | 0.685431642 | 0.529997865 | 0.551704505 | 0.524588997 |
| 0.291082485 | 0.875524874 | 0.367233649 | 0.875524874 | 0.450999929 | 0.507366024 | 0.502170664 |
| 0.689203615 | 0.84698598 | 0.816240837 | 0.84698598 | 0.410718098 | 0.52537186 | 0.480037008 |
| 0.256280692 | 0.891964985 | 0.681944346 | 0.891964985 | 0.461034802 | 0.429293289 | 0.41783503 |
| 0.34125685 | 0.732830404 | 0.728844922 | 0.732830404 | 0.46324105 | 0.328161697 | 0.459896093 |
| 0.430431998 | 0.838516832 | 0.765710626 | 0.838516832 | 0.476905558 | 0.366308448 | 0.442174934 |
| 0.29158067 | 0.648494769 | 0.887694826 | 0.648494769 | 0.423813252 | 0.278770194 | 0.3919294 |
| 0.296491353 | 0.67119778 | 0.490000712 | 0.67119778 | 0.416340474 | 0.3919294 | 0.486228738 |
| 0.304533485 | 0.797665647 | 0.57184542 | 0.797665647 | 0.555049463 | 0.337840723 | 0.437548929 |
| 0.326240125 | 0.75389652 | 0.556757526 | 0.75389652 | 0.386520532 | 0.512205537 | 0.537399473 |
| 0.444025336 | 0.844281546 | 0.76934026 | 0.844281546 | 0.4394705 | 0.457974521 | 0.511992029 |
| 0.405309231 | 0.70834816 | 0.501031955 | 0.70834816 | 0.403672336 | 0.443954167 | 0.396768913 |
| 0.235214575 | 0.785780372 | 0.48089104 | 0.785780372 | 0.608212939 | 0.534481532 | 0.438260622 |
| 0.362892321 | 0.74044552 | 0.703935663 | 0.74044552 | 0.400469717 | 0.362536474 | 0.435556188 |
| 0.375631628 | 0.867055726 | 0.966550423 | 0.867055726 | 0.47256423 | 0.420610633 | 0.478115437 |
| 0.315280051 | 0.669774393 | 0.487225109 | 0.669774393 | 0.471069675 | 0.366521956 | 0.384954807 |
| 0.469219273 | 0.662159277 | 0.834389011 | 0.662159277 | 0.447583802 | 0.377553199 | 0.384029606 |
| 0.40637677 | 0.717600171 | 0.883709345 | 0.717600171 | 0.457405167 | 0.594548431 | 0.388086257 |
| 0.526439399 | 0.778948118 | 0.728346737 | 0.778948118 | 0.392498755 | 0.47156786 | 0.405166892 |
| 0.379403601 | 0.732261049 | 0.509287595 | 0.732261049 | 0.451213437 | 0.560671838 | 0.539249875 |

| 0.591416981 | 0.885061561 | 0.753754181 | 0.885061561 | 0.408084834 | 0.581382108 | 0.472421892 |
| --- | --- | --- | --- | --- | --- | --- |
| 0.334211088 | 0.730552986 | 0.874386165 | 0.730552986 | 0.497046474 | 0.380044125 | 0.440324532 |
| 0.306597395 | 0.832609779 | 0.862785567 | 0.832609779 | 0.437548929 | 0.403814675 | 0.436908405 |
| 0.403102982 | 0.804142054 | 0.877588784 | 0.804142054 | 0.561098854 | 0.312433279 | 0.512134368 |
| 0.542452494 | 0.838374493 | 0.951035513 | 0.838374493 | 0.396341897 | 0.407017294 | 0.343961284 |
| 0.308234289 | 0.810618461 | 0.921286741 | 0.810618461 | 0.482172087 | 0.38260622 | 0.404597538 |
| 0.367518326 | 0.709842716 | 0.45505658 | 0.709842716 | 0.447085617 | 0.515265817 | 0.436766066 |
| 0.279054872 | 0.766066472 | 0.526297061 | 0.766066472 | 0.465945484 | 0.404882215 | 0.502384172 |
| 0.42815458 | 0.662016938 | 0.790904562 | 0.662016938 | 0.515265817 | 0.347377411 | 0.348373781 |
| 0.298768771 | 0.752757811 | 0.573838161 | 0.752757811 | 0.450715252 | 0.475838019 | 0.511209167 |
| 0.556117002 | 0.882997651 | 0.696035869 | 0.882997651 | 0.434061633 | 0.581951463 | 0.515336987 |
| 0.347519749 | 0.640452637 | 0.869973667 | 0.640452637 | 0.41982777 | 0.390221337 | 0.393850971 |
| 0.295921998 | 0.776243684 | 0.792612625 | 0.776243684 | 0.440751548 | 0.52323678 | 0.525870045 |
| 0.385097146 | 0.821009181 | 0.605508505 | 0.821009181 | 0.442957797 | 0.505159775 | 0.451213437 |
| 0.349085474 | 0.854031742 | 0.820582165 | 0.854031742 | 0.412497331 | 0.532773468 | 0.58323251 |
| 0.226318412 | 0.686499182 | 0.687993737 | 0.686499182 | 0.418475553 | 0.401750765 | 0.38879795 |
| 0.355348374 | 0.673475197 | 0.9524589 | 0.673475197 | 0.394420326 | 0.47363177 | 0.397551776 |
| 0.431072522 | 0.734680806 | 0.792826133 | 0.734680806 | 0.410006405 | 0.551419828 | 0.588997224 |
| 0.263112946 | 0.805494271 | 0.561454701 | 0.805494271 | 0.422674543 | 0.548644225 | 0.379261263 |
| 0.289160914 | 0.823428937 | 0.762792684 | 0.823428937 | 0.510710981 | 0.439256992 | 0.406092093 |
| 0.438829977 | 0.72905843 | 0.90897445 | 0.72905843 | 0.439968686 | 0.397622945 | 0.418048537 |
| 0.237919009 | 0.806205964 | 0.730695324 | 0.806205964 | 0.454202548 | 0.651839727 | 0.762935022 |
| 0.422105188 | 0.697316917 | 0.897943207 | 0.697316917 | 0.476834389 | 0.391288876 | 0.454344886 |
| 0.289872607 | 0.632196997 | 0.247811544 | 0.632196997 | 0.4322824 | 0.48089104 | 0.378620739 |
| 0.344601808 | 0.651341542 | 0.906270016 | 0.651341542 | 0.403245321 | 0.357198776 | 0.346025194 |
| 0.355134866 | 0.636894171 | 0.811543662 | 0.636894171 | 0.572272436 | 0.34332076 | 0.427371717 |
| 0.273147819 | 0.589281902 | 0.925912746 | 0.589281902 | 0.426588855 | 0.29570849 | 0.347590919 |
| 0.370792114 | 0.682940716 | 0.635399616 | 0.682940716 | 0.384314284 | 0.538965198 | 0.500960786 |
| 0.557753897 | 0.873603302 | 0.683723578 | 0.873603302 | 0.408013665 | 0.59767988 | 0.466301331 |
| 0.663155647 | 0.838872678 | 0.628496192 | 0.838872678 | 0.463739236 | 0.547149669 | 0.493345669 |
| 0.258842787 | 0.666642944 | 0.932175646 | 0.666642944 | 0.560031314 | 0.316774607 | 0.440822717 |
| 0.358408654 | 0.602163547 | 0.889616397 | 0.602163547 | 0.476905558 | 0.34538467 | 0.335990321 |
| 0.317984485 | 0.822432567 | 0.459540246 | 0.822432567 | 0.374919935 | 0.70834816 | 0.609992171 |
| 0.726781012 | 0.887481318 | 0.772044694 | 0.887481318 | 0.409792897 | 0.632481674 | 0.482599103 |
| 0.267952459 | 0.763504377 | 0.920646217 | 0.763504377 | 0.414988257 | 0.59355206 | 0.596042986 |
| 0.89659099 | 0.857519038 | 0.648210092 | 0.857519038 | 0.551490997 | 0.499110384 | 0.425165469 |
| 0.634616753 | 0.649206462 | 0.441249733 | 0.649206462 | 0.411002776 | 0.540317415 | 0.461461818 |
| 0.216568216 | 0.780300334 | 0.205394634 | 0.780300334 | 0.543235357 | 0.344032453 | 0.578606505 |
| 0.431357199 | 0.765995303 | 0.5811686 | 0.765995303 | 0.432780585 | 0.469717458 | 0.402533627 |
| 0.269162337 | 0.773895096 | 0.822361398 | 0.773895096 | 0.529713188 | 0.527008754 | 0.369368728 |
| 0.266884919 | 0.839228525 | 0.769482599 | 0.839228525 | 0.471069675 | 0.551490997 | 0.591843997 |
| 0.259981496 | 0.73318625 | 0.862073874 | 0.73318625 | 0.441890257 | 0.381325173 | 0.451355775 |
| 0.247099851 | 0.793466657 | 0.714753398 | 0.793466657 | 0.524731336 | 0.476122696 | 0.50153014 |
| 0.257917586 | 0.666714113 | 0.607358907 | 0.666714113 | 0.396199559 | 0.394420326 | 0.341826205 |
| 0.240409935 | 0.639598605 | 0.750907409 | 0.639598605 | 0.372856024 | 0.4260195 | 0.366806633 |
| 0.768628567 | 0.883282329 | 0.821293858 | 0.883282329 | 0.414134225 | 0.509002918 | 0.677745356 |
| 0.30296776 | 0.715820938 | 0.847768842 | 0.715820938 | 0.441320902 | 0.357625792 | 0.368087681 |
| 0.307807274 | 0.776457192 | 0.118496904 | 0.776457192 | 0.431926553 | 0.58736033 | 0.603586933 |
| 0.256778877 | 0.805707779 | 0.692477404 | 0.805707779 | 0.380399972 | 0.426588855 | 0.446373924 |
| 0.554764785 | 0.804640239 | 0.575830902 | 0.804640239 | 0.406661448 | 0.602021208 | 0.613123621 |

| 0.272934311 | 0.70834816 | 0.905131307 | 0.70834816 | 0.488292648 | 0.318696178 | 0.452636823 |
| --- | --- | --- | --- | --- | --- | --- |
| 0.521884563 | 0.764358409 | 0.743363462 | 0.764358409 | 0.551063981 | 0.411358622 | 0.471852537 |
| 0.170806348 | 0.686285674 | 0.598035727 | 0.686285674 | 0.419258416 | 0.535762579 | 0.533841008 |
| 0.287879866 | 0.871254715 | 0.297914739 | 0.871254715 | 0.411643299 | 0.603586933 | 0.617607288 |
| 0.380257633 | 0.779090456 | 0.82001281 | 0.779090456 | 0.437050744 | 0.489075511 | 0.455625934 |
| 0.546509145 | 0.83659526 | 0.59155932 | 0.83659526 | 0.393637464 | 0.472848908 | 0.410291082 |
| 0.518468436 | 0.709059853 | 0.769980784 | 0.709059853 | 0.449718881 | 0.394206818 | 0.584727066 |
| 0.401466088 | 0.71767134 | 0.73838161 | 0.71767134 | 0.373994733 | 0.389367305 | 0.432424738 |
| 0.614475838 | 0.880435556 | 0.669489716 | 0.880435556 | 0.394206818 | 0.495053733 | 0.484947691 |
| 0.434559818 | 0.704362679 | 0.925841577 | 0.704362679 | 0.396341897 | 0.490641235 | 0.507579532 |
| 0.698811472 | 0.905771831 | 0.649348801 | 0.905771831 | 0.400042702 | 0.552558537 | 0.644580457 |
| 0.336702014 | 0.74870116 | 0.266457903 | 0.74870116 | 0.357696961 | 0.66287097 | 0.672834674 |
| 0.37435058 | 0.787701943 | 0.958081275 | 0.787701943 | 0.484734183 | 0.890541598 | 0.448651342 |
| 0.269802861 | 0.638673404 | 0.405238061 | 0.638673404 | 0.458828553 | 0.408867696 | 0.368941712 |
| 0.314995374 | 0.815102128 | 0.689844139 | 0.815102128 | 0.380115294 | 0.588214362 | 0.503309373 |
| 0.607358907 | 0.848480535 | 0.739591488 | 0.848480535 | 0.351291723 | 0.554622447 | 0.466443669 |
| 0.375987474 | 0.837378123 | 0.450430574 | 0.837378123 | 0.347875596 | 0.485872892 | 0.544729912 |
| 0.327307665 | 0.724076578 | 0.850544445 | 0.724076578 | 0.479182976 | 0.356415913 | 0.361113088 |
| 0.325457263 | 0.720945128 | 0.602377055 | 0.720945128 | 0.426375347 | 0.390221337 | 0.45299267 |
| 0.35577539 | 0.672905843 | 0.634047399 | 0.672905843 | 0.446018077 | 0.424311437 | 0.448366664 |
| 0.772115864 | 0.679239912 | 0.497971675 | 0.679239912 | 0.464806775 | 0.473774109 | 0.442815458 |
| 0.415272934 | 0.766920504 | 0.590349441 | 0.766920504 | 0.41157213 | 0.594121415 | 0.656465732 |
| 0.747562451 | 0.745498541 | 0.421749342 | 0.745498541 | 0.354992527 | 0.476905558 | 0.472991246 |
| 0.540744431 | 0.732616896 | 0.59668351 | 0.732616896 | 0.512347876 | 0.430503167 | 0.438687638 |
| 0.348658458 | 0.858088392 | 0.279268379 | 0.858088392 | 0.336559675 | 0.571276066 | 0.691623372 |
| 0.438901146 | 0.820297488 | 0.64429578 | 0.820297488 | 0.413991887 | 0.464166252 | 0.50978578 |
| 0.328731051 | 0.736815885 | 0.601380685 | 0.736815885 | 0.477403744 | 0.420539463 | 0.52530069 |
| 0.453063839 | 0.911536545 | 0.821222689 | 0.911536545 | 0.551135151 | 0.535620241 | 0.576969611 |
| 0.362180628 | 0.773325742 | 0.941569995 | 0.773325742 | 0.414561241 | 0.539463383 | 0.565938367 |
| 0.492064622 | 0.7632197 | 0.514340616 | 0.7632197 | 0.401750765 | 0.64429578 | 0.577467796 |
| 0.258629279 | 0.895950466 | 0.156430147 | 0.895950466 | 0.474343463 | 0.503167034 | 0.581524447 |
| 0.38680521 | 0.745071525 | 0.868906128 | 0.745071525 | 0.414845918 | 0.385951178 | 0.38780158 |
| 0.453917871 | 0.886556117 | 0.832182763 | 0.886556117 | 0.39292577 | 0.548644225 | 0.479609992 |
| 0.310582877 | 0.848622874 | 0.290086115 | 0.848622874 | 0.417479183 | 0.572699452 | 0.662159277 |
| 0.259625649 | 0.587075653 | 0.521528717 | 0.587075653 | 0.454558394 | 0.370009252 | 0.386164686 |
| 0.308803644 | 0.926197424 | 0.562237563 | 0.926197424 | 0.46331222 | 0.53675895 | 0.467938225 |
| 0.356415913 | 0.877090599 | 0.66080706 | 0.877090599 | 0.497900505 | 0.551277489 | 0.497117643 |
| 0.347946765 | 0.867269234 | 0.855953313 | 0.867269234 | 0.385452993 | 0.481033378 | 0.492064622 |
| 0.528787987 | 0.69489716 | 0.869404313 | 0.69489716 | 0.430645506 | 0.473489431 | 0.441890257 |
| 0.45911323 | 0.896733329 | 0.877019429 | 0.896733329 | 0.452636823 | 0.564230304 | 0.550423457 |
| 0.361041919 | 0.853604726 | 0.571204896 | 0.853604726 | 0.445448723 | 0.388726781 | 0.474770479 |
| 0.468649918 | 0.703793324 | 0.86556117 | 0.703793324 | 0.520745854 | 0.370507437 | 0.411358622 |
| 0.349370152 | 0.682300192 | 0.725642303 | 0.682300192 | 0.373994733 | 0.451640453 | 0.477973098 |
| 0.427869902 | 0.785139848 | 0.532773468 | 0.785139848 | 0.459611416 | 0.33919294 | 0.457689844 |
| 0.689488293 | 0.864351292 | 0.769980784 | 0.864351292 | 0.575759732 | 0.552629706 | 0.463383389 |
| 0.36709131 | 0.816525514 | 0.465589638 | 0.816525514 | 0.466087823 | 0.53569141 | 0.591416981 |
| 0.503522881 | 0.864351292 | 0.689203615 | 0.864351292 | 0.370009252 | 0.426731194 | 0.431286029 |
| 0.509358765 | 0.808554551 | 0.54088677 | 0.808554551 | 0.394420326 | 0.428012241 | 0.473062415 |
| 0.435769696 | 0.881645434 | 0.810404953 | 0.881645434 | 0.384456622 | 0.561312362 | 0.576542595 |
| 0.543591203 | 0.84492207 | 0.499822077 | 0.84492207 | 0.391004199 | 0.508575902 | 0.568358124 |

| 0.337627215 | 0.82001281 | 0.885061561 | 0.82001281 | 0.516404526 | 0.385951178 | 0.423457405 |
| --- | --- | --- | --- | --- | --- | --- |
| 0.415628781 | 0.678457049 | 0.885630916 | 0.678457049 | 0.429506797 | 0.338837093 | 0.340687496 |
| 0.338837093 | 0.932958508 | 0.78592271 | 0.932958508 | 0.480606363 | 0.579745214 | 0.423599744 |
| 0.427727564 | 0.859867625 | 0.789837022 | 0.859867625 | 0.45299267 | 0.502740019 | 0.500676108 |
| 0.268521813 | 0.53882286 | 0.632339335 | 0.53882286 | 0.464237421 | 0.897018006 | 0.319479041 |
| 0.294569782 | 0.763077361 | 0.869475482 | 0.763077361 | 0.431570707 | 0.456906982 | 0.484663013 |
| 0.603231087 | 0.754964059 | 0.540673262 | 0.754964059 | 0.401821934 | 0.477617251 | 0.418190876 |
| 0.387588072 | 0.812753541 | 0.851184969 | 0.812753541 | 0.451782791 | 0.578891182 | 0.509358765 |
| 0.300832681 | 0.867198064 | 0.797523308 | 0.867198064 | 0.475411003 | 0.489431357 | 0.418760231 |
| 0.439755178 | 0.919080492 | 0.809052736 | 0.919080492 | 0.443171305 | 0.653334282 | 0.654757668 |
| 0.464450929 | 0.84492207 | 0.843498683 | 0.84492207 | 0.47875596 | 0.536474272 | 0.517329727 |
| 0.408796527 | 0.866415202 | 0.828126112 | 0.866415202 | 0.428225749 | 0.594406092 | 0.570635542 |
| 0.304533485 | 0.717956017 | 0.885346239 | 0.717956017 | 0.525016013 | 0.294569782 | 0.451853961 |
| 0.350295353 | 0.706924774 | 0.876521244 | 0.706924774 | 0.438545299 | 0.379617109 | 0.427941072 |
| 0.443242474 | 0.949825635 | 0.693616113 | 0.949825635 | 0.455625934 | 0.490854743 | 0.555974664 |
| 0.379972956 | 0.908547434 | 0.693331435 | 0.908547434 | 0.493559177 | 0.602021208 | 0.710625578 |
| 0.380898157 | 0.809052736 | 0.637392356 | 0.809052736 | 0.42808341 | 0.46331222 | 0.413707209 |
| 0.371076792 | 0.74770479 | 0.674400399 | 0.74770479 | 0.651910896 | 0.487153939 | 0.50366522 |
| 0.335776813 | 0.869404313 | 0.497900505 | 0.869404313 | 0.461960003 | 0.555761156 | 0.604796812 |
| 0.614475838 | 0.791260409 | 0.841434773 | 0.791260409 | 0.318696178 | 0.51597751 | 0.452636823 |
| 0.593409722 | 0.945626646 | 0.646929044 | 0.945626646 | 0.493060992 | 0.420254786 | 0.499822077 |

| Nucleotide ex | Oocyte meios | P53 signaling | Pan-F-TBRS | Progesterone- | Proteasome | RNA degrada |
| --- | --- | --- | --- | --- | --- | --- |
| 0.939292577 | 0.716247954 | 0.352501601 | 0.11992029 | 0.370792114 | 0.0878941 | 0.734467298 |
| 0.908049249 | 0.544658743 | 0.315920575 | 0.053804 | 0.158351719 | 0.10034873 | 0.658671981 |
| 0.946053662 | 0.786065049 | 0.26375347 | 0.106896306 | 0.319906057 | 0.097644296 | 0.726709843 |
| 0.903138567 | 0.630560102 | 0.259412141 | 0.156999502 | 0.200341613 | 0.079069105 | 0.522667426 |
| 0.911394207 | 0.662016938 | 0.240765782 | 0.227599459 | 0.143406163 | 0.114226745 | 0.676891324 |
| 0.910397836 | 0.682442531 | 0.288662729 | 0.05166892 | 0.172799089 | 0.074514269 | 0.705999573 |
| 0.875097858 | 0.687210875 | 0.253718597 | 0.119635613 | 0.280051242 | 0.133086613 | 0.721300975 |
| 0.905487154 | 0.746708419 | 0.26781012 | 0.088321116 | 0.264678671 | 0.116148317 | 0.71041207 |
| 0.907052879 | 0.795672906 | 0.317699808 | 0.068678386 | 0.344103623 | 0.061490285 | 0.68870543 |
| 0.917016582 | 0.681944346 | 0.250587147 | 0.124973312 | 0.324532062 | 0.034730624 | 0.724432425 |
| 0.922496619 | 0.503522881 | 0.212511565 | 0.09202192 | 0.128816454 | 0.082841079 | 0.661305245 |
| 0.890612768 | 0.735036652 | 0.305245178 | 0.046331222 | 0.263895808 | 0.107892677 | 0.719094726 |
| 0.908903281 | 0.691908049 | 0.268165967 | 0.075652978 | 0.258985126 | 0.08170237 | 0.722012668 |
| 0.902284535 | 0.504732759 | 0.248665575 | 0.040922354 | 0.085474343 | 0.102697317 | 0.683438901 |
| 0.919436339 | 0.610917372 | 0.264465163 | 0.102483809 | 0.141342253 | 0.159134581 | 0.637605864 |
| 0.914098641 | 0.714753398 | 0.301544374 | 0.09614974 | 0.175432354 | 0.08476265 | 0.6215216 |
| 0.934239556 | 0.532915807 | 0.3174863 | 0.045690698 | 0.161483168 | 0.068749555 | 0.661518753 |
| 0.910896022 | 0.706497758 | 0.272364956 | 0.094157 | 0.332147178 | 0.087040068 | 0.701658245 |
| 0.946053662 | 0.770621308 | 0.31542239 | 0.059355206 | 0.351718739 | 0.096576756 | 0.741086044 |
| 0.929471212 | 0.616041563 | 0.240196427 | 0.060920931 | 0.212369226 | 0.07131165 | 0.745071525 |
| 0.92662444 | 0.778805779 | 0.258344602 | 0.085189666 | 0.229449861 | 0.087324746 | 0.729556615 |
| 0.908120418 | 0.757383816 | 0.313073803 | 0.140701729 | 0.274357697 | 0.095580386 | 0.675610277 |
| 0.920219201 | 0.532061775 | 0.260408512 | 0.091523735 | 0.127891253 | 0.088321116 | 0.601950039 |
| 0.930111736 | 0.763290869 | 0.257348231 | 0.100206391 | 0.315849406 | 0.101700947 | 0.716105615 |
| 0.910255498 | 0.820155149 | 0.271155078 | 0.078072735 | 0.3040353 | 0.13963419 | 0.689417123 |
| 0.943278059 | 0.376699167 | 0.307380258 | 0.032666714 | 0.115436624 | 0.10340901 | 0.632125827 |
| 0.876023059 | 0.783075938 | 0.273788342 | 0.087965269 | 0.189879724 | 0.089602164 | 0.581097431 |
| 0.888050673 | 0.769980784 | 0.34744858 | 0.156003131 | 0.195359761 | 0.047683439 | 0.739093303 |
| 0.942495196 | 0.756245107 | 0.29677603 | 0.1354352 | 0.284321401 | 0.120276137 | 0.759803573 |
| 0.900362963 | 0.573126468 | 0.32161412 | 0.176286385 | 0.194292221 | 0.092448936 | 0.64941997 |
| 0.903707921 | 0.55013878 | 0.401608426 | 0.117500534 | 0.115934809 | 0.115151946 | 0.71767134 |
| 0.934737741 | 0.757099139 | 0.325243755 | 0.175290015 | 0.365240908 | 0.089459825 | 0.723222546 |
| 0.909757313 | 0.708988684 | 0.364173368 | 0.120347306 | 0.368372358 | 0.092448936 | 0.691054018 |
| 0.934310725 | 0.732759234 | 0.295139136 | 0.064977582 | 0.349156644 | 0.104690058 | 0.700590705 |
| 0.925770408 | 0.746993097 | 0.316561099 | 0.087822931 | 0.34951249 | 0.088321116 | 0.750053377 |
| 0.946124831 | 0.700377197 | 0.272507295 | 0.096078571 | 0.200839798 | 0.114724931 | 0.704505017 |
| 0.919934524 | 0.830332361 | 0.273574835 | 0.087751761 | 0.275781083 | 0.080492492 | 0.675539108 |
| 0.907337556 | 0.863070244 | 0.412853178 | 0.079424952 | 0.343890115 | 0.128318269 | 0.773681588 |
| 0.920646217 | 0.558038574 | 0.289801438 | 0.082912248 | 0.264536332 | 0.104974735 | 0.735890684 |
| 0.913956302 | 0.757739663 | 0.281474628 | 0.083339264 | 0.367518326 | 0.105117074 | 0.704078002 |
| 0.881147249 | 0.514909971 | 0.320261903 | 0.049249164 | 0.234147036 | 0.137214433 | 0.726282827 |
| 0.826133371 | 0.803003345 | 0.292221194 | 0.05586791 | 0.322183474 | 0.11586364 | 0.828481959 |
| 0.902925059 | 0.509287595 | 0.263397623 | 0.066329799 | 0.183545655 | 0.078784428 | 0.742224753 |
| 0.9255569 | 0.794961213 | 0.3515764 | 0.050387873 | 0.371788485 | 0.083339264 | 0.762365668 |
| 0.928118995 | 0.30609921 | 0.263824639 | 0.183616824 | 0.062344317 | 0.042843926 | 0.657960288 |
| 0.90897445 | 0.720162266 | 0.218418618 | 0.107963846 | 0.19749484 | 0.048466301 | 0.633478044 |
| 0.963063127 | 0.910469006 | 0.264678671 | 0.146110597 | 0.374279411 | 0.149882571 | 0.738879795 |
| 0.942851043 | 0.378264892 | 0.255711337 | 0.047256423 | 0.241619814 | 0.043555619 | 0.673902213 |

| 0.926837947 | 0.689132446 | 0.28332503 | 0.213721443 | 0.198277703 | 0.109529571 | 0.704149171 |
| --- | --- | --- | --- | --- | --- | --- |
| 0.926980286 | 0.791758594 | 0.231086755 | 0.134011814 | 0.191089602 | 0.139064835 | 0.734538467 |
| 0.958437122 | 0.741940075 | 0.2161412 | 0.109743079 | 0.216497046 | 0.031243328 | 0.736104192 |
| 0.890114583 | 0.420824141 | 0.23891538 | 0.06511992 | 0.196640808 | 0.071453989 | 0.632125827 |
| 0.944416767 | 0.533698669 | 0.207031528 | 0.07344673 | 0.181197068 | 0.14269447 | 0.679951605 |
| 0.927549641 | 0.775104975 | 0.193936375 | 0.036936873 | 0.27613693 | 0.141769269 | 0.76627998 |
| 0.940146609 | 0.694256637 | 0.324389723 | 0.186677105 | 0.237919009 | 0.059782222 | 0.678955235 |
| 0.909614974 | 0.761369298 | 0.310369369 | 0.119706782 | 0.238488364 | 0.069959433 | 0.78179489 |
| 0.924987545 | 0.764643086 | 0.323251014 | 0.116717671 | 0.289872607 | 0.07551064 | 0.727777382 |
| 0.946551847 | 0.623941356 | 0.202761369 | 0.072806206 | 0.168457761 | 0.046900576 | 0.704149171 |
| 0.939933101 | 0.695395346 | 0.249733115 | 0.089388656 | 0.248523237 | 0.082200555 | 0.75282898 |
| 0.931819799 | 0.678030033 | 0.23058857 | 0.179417835 | 0.236993808 | 0 | 0.691338695 |
| 0.956871397 | 0.642374208 | 0.286100633 | 0.196783147 | 0.326524803 | 0.060280407 | 0.718098356 |
| 0.923920006 | 0.684435272 | 0.310725215 | 0.161269661 | 0.196142623 | 0.107607999 | 0.755675753 |
| 0.901928688 | 0.763860223 | 0.286242972 | 0.091381396 | 0.332787702 | 0.088605793 | 0.722439684 |
| 0.934524233 | 0.834673689 | 0.304248808 | 0.120418476 | 0.292505871 | 0.056152587 | 0.735534837 |
| 0.886698456 | 0.926410932 | 0.273646004 | 0.116005978 | 0.45704932 | 0.053661661 | 0.828553128 |
| 0.935520604 | 0.689630631 | 0.288022205 | 0.535122055 | 0.130595687 | 0.037577397 | 0.707992314 |
| 0.936018789 | 0.891964985 | 0.233293004 | 0.05786065 | 0.153868052 | 0.062273148 | 0.736887054 |
| 0.931606291 | 0.643797594 | 0.276635115 | 0.259483311 | 0.226958935 | 0.116219486 | 0.70422034 |
| 0.939363746 | 0.71866771 | 0.250658316 | 0.272436126 | 0.284677247 | 0.065902783 | 0.720945128 |
| 0.91523735 | 0.591416981 | 0.25435912 | 0.096932603 | 0.217422248 | 0.080848338 | 0.696747562 |
| 0.832894456 | 0.557326881 | 0.224610348 | 0.026403815 | 0.241477475 | 0.124546296 | 0.617536118 |
| 0.940431286 | 0.866272863 | 0.211088179 | 0.256565369 | 0.247313358 | 0.116931179 | 0.738666287 |
| 0.899081916 | 0.578820013 | 0.22539321 | 0.18197993 | 0.167319052 | 0.086541883 | 0.693260266 |
| 0.928190164 | 0.767774536 | 0.224966195 | 0.219628496 | 0.28546011 | 0.105899936 | 0.692335065 |
| 0.952316561 | 0.775247313 | 0.290797808 | 0.130168671 | 0.237634332 | 0.063625365 | 0.797879155 |
| 0.937442175 | 0.724717102 | 0.222404099 | 0.057789481 | 0.269731692 | 0.134225322 | 0.705359049 |
| 0.906768202 | 0.281901644 | 0.268948829 | 0.082912248 | 0.037577397 | 0.034659455 | 0.629136716 |
| 0.931321614 | 0.499395061 | 0.282399829 | 0.089886841 | 0.179702512 | 0.105971105 | 0.709202192 |
| 0.95658672 | 0.694470144 | 0.221478898 | 0.064906412 | 0.273788342 | 0.097857804 | 0.862002704 |
| 0.959362323 | 0.81588499 | 0.317842146 | 0.090242687 | 0.238274856 | 0.663369155 | 0.801010604 |
| 0.850330937 | 0.499750907 | 0.238559533 | 0.120062629 | 0.217351078 | 0.063767703 | 0.707565298 |
| 0.943776244 | 0.721016298 | 0.30923066 | 0.110027756 | 0.047896947 | 0.051811259 | 0.777168885 |
| 0.939933101 | 0.635613124 | 0.267738951 | 0.064835243 | 0.114297915 | 0.10646929 | 0.76314853 |
| 0.943918582 | 0.636894171 | 0.203401893 | 0.097857804 | 0.228595829 | 0.057291296 | 0.731051171 |
| 0.938865561 | 0.891751477 | 0.176286385 | 0.086257206 | 0.234289374 | 0.076435841 | 0.729627784 |
| 0.942922212 | 0.791687424 | 0.241833321 | 0.109173724 | 0.228666999 | 0.08170237 | 0.721656822 |
| 0.94107181 | 0.776030176 | 0.256636538 | 0.063910042 | 0.250017792 | 0.043769127 | 0.779375133 |
| 0.923635328 | 0.832680948 | 0.218703295 | 0.331008469 | 0.256707708 | 0.021279624 | 0.713187673 |
| 0.925201053 | 0.814390435 | 0.287239342 | 0.142552132 | 0.22546438 | 0.064835243 | 0.722155007 |
| 0.926197424 | 0.770550139 | 0.206746851 | 0.080990677 | 0.25642303 | 0.072236851 | 0.731264679 |
| 0.943562736 | 0.698882642 | 0.218916803 | 0.05273646 | 0.274927051 | 0.046971746 | 0.785566864 |
| 0.923848836 | 0.793680165 | 0.259198634 | 0.096790264 | 0.231940787 | 0.117714042 | 0.745213864 |
| 0.935164757 | 0.745996726 | 0.290655469 | 0.052949968 | 0.253433919 | 0.044979005 | 0.777738239 |
| 0.934666572 | 0.730481816 | 0.227243613 | 0.240623443 | 0.203615401 | 0.050316703 | 0.649277631 |
| 0.922496619 | 0.616112732 | 0.2636823 | 0.120418476 | 0.17059284 | 0.085332005 | 0.696035869 |
| 0.938153868 | 0.730766493 | 0.220411359 | 0.05579674 | 0.210305316 | 0.109102555 | 0.686356843 |
| 0.936161127 | 0.659953028 | 0.260906697 | 0.113088036 | 0.276208099 | 0.025407444 | 0.70108889 |
| 0.912817593 | 0.762436837 | 0.225677888 | 0.103836026 | 0.221621237 | 0.048395132 | 0.756173938 |

| 0.934524233 | 0.423315067 | 0.24097929 | 0.089815671 | 0.150878941 | 0.043342111 | 0.762721514 |
| --- | --- | --- | --- | --- | --- | --- |
| 0.92036154 | 0.645647997 | 0.216568216 | 0.097857804 | 0.31229094 | 0.077503381 | 0.711621949 |
| 0.935378265 | 0.371432638 | 0.268094798 | 0.254714967 | 0.104263042 | 0.090740873 | 0.682300192 |
| 0.940075439 | 0.650345171 | 0.194719237 | 0.145968258 | 0.295423813 | 0.113230375 | 0.667212298 |
| 0.932389154 | 0.636395986 | 0.231584941 | 0.076578179 | 0.193722867 | 0.054942709 | 0.673902213 |
| 0.869973667 | 0.436125543 | 0.28546011 | 0.313144972 | 0.159561597 | 0.06924774 | 0.720162266 |
| 0.955020995 | 0.861575689 | 0.288164543 | 0.107323322 | 0.255213152 | 0.076079994 | 0.639456266 |
| 0.913742794 | 0.64941997 | 0.326524803 | 0.126254359 | 0.223044623 | 0.053946338 | 0.740018504 |
| 0.907124048 | 0.710767917 | 0.317059284 | 0.342893744 | 0.139349512 | 0.016653619 | 0.755035229 |
| 0.895879297 | 0.719664081 | 0.298626432 | 0.160130952 | 0.224539179 | 0.11273219 | 0.710198562 |
| 0.890470429 | 0.636324817 | 0.277560316 | 0.163547078 | 0.206319835 | 0.071881005 | 0.749341684 |
| 0.935235926 | 0.597608711 | 0.294071596 | 0.122695894 | 0.252152872 | 0.026261476 | 0.740730197 |
| 0.946907693 | 0.696818732 | 0.20994947 | 0.129172301 | 0.259269803 | 0.071596328 | 0.749839869 |
| 0.929044196 | 0.747277774 | 0.270087538 | 0.096861433 | 0.293929258 | 0.08063483 | 0.725571134 |
| 0.91310227 | 0.636395986 | 0.333855242 | 0.087040068 | 0.247811544 | 0.104832396 | 0.709059853 |
| 0.949398619 | 0.706568927 | 0.301829051 | 0.076791687 | 0.123549925 | 0.065831613 | 0.798092662 |
| 0.906412355 | 0.650274002 | 0.261903067 | 0.082200555 | 0.315778236 | 0.094726354 | 0.655967547 |
| 0.950679667 | 0.721087467 | 0.244608925 | 0.09928119 | 0.15507793 | 0.07864209 | 0.720589282 |
| 0.959647 | 0.780086827 | 0.275354067 | 0.127748915 | 0.274784713 | 0.054231016 | 0.876877091 |
| 0.960358693 | 0.734467298 | 0.262472422 | 0.092235428 | 0.296491353 | 0.060066899 | 0.715180414 |
| 0.938652053 | 0.711906626 | 0.233150665 | 0.11999146 | 0.26062202 | 0.084406804 | 0.7435058 |
| 0.935093588 | 0.374564088 | 0.249661946 | 0.824354138 | 0.038716106 | 0.048537471 | 0.696818732 |
| 0.928190164 | 0.600028468 | 0.20681802 | 0.184613195 | 0.250871824 | 0.096434417 | 0.7569568 |
| 0.927193794 | 0.258985126 | 0.20368657 | 0.121770692 | 0.042630418 | 0.15201765 | 0.614475838 |
| 0.943989752 | 0.720233435 | 0.26161839 | 0.124403957 | 0.282684506 | 0.046331222 | 0.760372927 |
| 0.93694399 | 0.58323251 | 0.24510711 | 0.142409793 | 0.177638602 | 0.031741513 | 0.705501388 |
| 0.934239556 | 0.623799018 | 0.311436908 | 0.104832396 | 0.181481745 | 0.133869475 | 0.69909615 |
| 0.925201053 | 0.679880436 | 0.222973454 | 0.105401751 | 0.248309729 | 0.073589068 | 0.720162266 |
| 0.954878656 | 0.70841933 | 0.228453491 | 0.158138211 | 0.191516618 | 0.080848338 | 0.770194292 |
| 0.931321614 | 0.813536403 | 0.32054658 | 0.11792755 | 0.304746993 | 0.062700164 | 0.71767134 |
| 0.920148032 | 0.708632838 | 0.362678813 | 0.126539036 | 0.16546865 | 0.038360259 | 0.748629991 |
| 0.909330297 | 0.857661376 | 0.264963348 | 0.094299338 | 0.224681517 | 0.085545513 | 0.794890043 |
| 0.931962138 | 0.870329514 | 0.35058003 | 0.136289232 | 0.263824639 | 0.070599957 | 0.736460038 |
| 0.932887339 | 0.431784215 | 0.260266173 | 0.078855597 | 0.16340474 | 0.089388656 | 0.762792684 |
| 0.891039784 | 0.486726923 | 0.231940787 | 0.099210021 | 0.079709629 | 0.192014803 | 0.736815885 |
| 0.898583731 | 0.845633763 | 0.35371148 | 0.257704078 | 0.262472422 | 0.12618319 | 0.718525372 |
| 0.896875667 | 0.425592485 | 0.3309373 | 0.1013451 | 0.122340047 | 0.089317486 | 0.695751192 |
| 0.924204683 | 0.875809551 | 0.328588713 | 0.078215074 | 0.22126539 | 0.07963846 | 0.760017081 |
| 0.91004199 | 0.71354352 | 0.330866131 | 0.096932603 | 0.223542808 | 0.066472137 | 0.727350367 |
| 0.919151662 | 0.669560885 | 0.306312718 | 0.26681375 | 0.28752402 | 0.139990036 | 0.752757811 |
| 0.903992598 | 0.352928617 | 0.298341755 | 0.163902925 | 0.161269661 | 0.068464878 | 0.57191659 |
| 0.932887339 | 0.526225891 | 0.425663654 | 0.15408156 | 0.196427301 | 0.078428582 | 0.732545726 |
| 0.889901075 | 0.797096292 | 0.310013522 | 0.276563946 | 0.332431855 | 0.108889047 | 0.765070102 |
| 0.940431286 | 0.496619458 | 0.278272009 | 0.072735037 | 0.133798306 | 0.020140915 | 0.72699452 |
| 0.942922212 | 0.805209594 | 0.305672194 | 0.105259412 | 0.309586506 | 0.055725571 | 0.735890684 |
| 0.929613551 | 0.678812896 | 0.325884279 | 0.161483168 | 0.166607359 | 0.076364672 | 0.696035869 |
| 0.909187958 | 0.679738097 | 0.244608925 | 0.106326952 | 0.258131094 | 0.091239058 | 0.708276991 |
| 0.872393424 | 0.637178848 | 0.296206676 | 0.151590634 | 0.316845776 | 0.065404598 | 0.707209451 |
| 0.913386948 | 0.603586933 | 0.302184898 | 0.219130311 | 0.262330083 | 0.057647143 | 0.709344531 |
| 0.929755889 | 0.803572699 | 0.276919792 | 0.094726354 | 0.274215358 | 0.055013878 | 0.76934026 |

| 0.917016582 | 0.558750267 | 0.318980855 | 0.140701729 | 0.272934311 | 0.04341328 | 0.68144616 |
| --- | --- | --- | --- | --- | --- | --- |
| 0.903921429 | 0.682798377 | 0.267383104 | 0.135791047 | 0.251725856 | 0.110525941 | 0.720233435 |
| 0.880364387 | 0.704505017 | 0.322183474 | 0.128460608 | 0.222475269 | 0.084976158 | 0.74870116 |
| 0.906697032 | 0.649633478 | 0.269375845 | 0.593979076 | 0.189096861 | 0.086755391 | 0.694541314 |
| 0.936516974 | 0.690982848 | 0.245676464 | 0.157924703 | 0.265176856 | 0.099494698 | 0.671980642 |
| 0.913600455 | 0.706711266 | 0.336630845 | 0.208312576 | 0.244893602 | 0.054373354 | 0.697601594 |
| 0.862785567 | 0.56665006 | 0.334922781 | 0.140559391 | 0.203900078 | 0.059212867 | 0.736531208 |
| 0.906198847 | 0.533912177 | 0.281972813 | 0.367020141 | 0.242331507 | 0.22752829 | 0.74457334 |
| 0.902355704 | 0.614760515 | 0.317059284 | 0.152159989 | 0.314924205 | 0.122126539 | 0.690413494 |
| 0.928403672 | 0.772258202 | 0.336986691 | 0.174151306 | 0.276350438 | 0.077645719 | 0.763931393 |
| 0.891751477 | 0.726140488 | 0.297487723 | 0.130595687 | 0.270799231 | 0.043270942 | 0.697672764 |
| 0.899935948 | 0.803857377 | 0.291438332 | 0.466443669 | 0.227599459 | 0.066899153 | 0.714966906 |
| 0.938438545 | 0.641306669 | 0.277062131 | 0.278556686 | 0.116290655 | 0.08063483 | 0.678030033 |
| 0.892392001 | 0.734396128 | 0.296633692 | 0.311650416 | 0.193224682 | 0.052878799 | 0.681019145 |
| 0.933741371 | 0.512276706 | 0.341185681 | 0.06412355 | 0.03622518 | 0.10753683 | 0.706711266 |
| 0.925201053 | 0.679951605 | 0.281047612 | 0.107109814 | 0.029392926 | 0.10241264 | 0.726496335 |
| 0.937086328 | 0.554195431 | 0.383531421 | 0.181837592 | 0.079353783 | 0.061490285 | 0.700448367 |
| 0.959789339 | 0.828837805 | 0.346665718 | 0.123194079 | 0.086114867 | 0.121272507 | 0.680663298 |
| 0.864635969 | 0.65041634 | 0.349085474 | 0.113301544 | 0.216497046 | 0.059497545 | 0.59148815 |
| 0.932104477 | 0.621735108 | 0.31335848 | 0.115934809 | 0.21713757 | 0.126894883 | 0.691267525 |
| 0.90071881 | 0.440466871 | 0.298057078 | 0.099708206 | 0.097074941 | 0.08276991 | 0.640381468 |
| 0.932317984 | 0.653049605 | 0.294356274 | 0.244253078 | 0.123763433 | 0.069888264 | 0.768415059 |
| 0.945982492 | 0.789979361 | 0.297203046 | 0.078713259 | 0.19336702 | 0.089886841 | 0.713828197 |
| 0.881503096 | 0.43847413 | 0.409650559 | 0.074727777 | 0.037008042 | 0.113515052 | 0.712048964 |
| 0.935947619 | 0.621806277 | 0.291082485 | 0.274357697 | 0.103836026 | 0.107465661 | 0.641876023 |
| 0.901003487 | 0.872393424 | 0.271582094 | 0.20788556 | 0.179631343 | 0.103337841 | 0.783360615 |
| 0.897872038 | 0.689630631 | 0.405451569 | 0.127606576 | 0.17066401 | 0.065262259 | 0.687210875 |
| 0.88627144 | 0.772827557 | 0.261689559 | 0.099850544 | 0.280905274 | 0.089317486 | 0.614902854 |
| 0.90897445 | 0.595260124 | 0.326168956 | 0.051597751 | 0.074158423 | 0.078286243 | 0.734965483 |
| 0.898370223 | 0.72699452 | 0.3712903 | 0.077788058 | 0.199701089 | 0.093302968 | 0.711906626 |
| 0.926339762 | 0.598391574 | 0.326951818 | 0.099850544 | 0.125969682 | 0.104191872 | 0.690342324 |
| 0.901501672 | 0.517827913 | 0.314141342 | 0.122268878 | 0.078997936 | 0.066756814 | 0.65148388 |
| 0.928973027 | 0.574763362 | 0.414276564 | 0.212155718 | 0.045904206 | 0.075581809 | 0.63803288 |
| 0.90691054 | 0.750693901 | 0.270514554 | 0.08376628 | 0.228666999 | 0.123336417 | 0.750409224 |
| 0.889616397 | 0.494697886 | 0.336915522 | 0.429649135 | 0.180627713 | 0.13237492 | 0.788769483 |
| 0.933100847 | 0.641947192 | 0.223542808 | 0.060849762 | 0.220340189 | 0.086755391 | 0.685929827 |
| 0.92142908 | 0.574763362 | 0.295139136 | 0.14682229 | 0.247028681 | 0.107323322 | 0.728204398 |
| 0.899437762 | 0.629065547 | 0.31955021 | 0.142623301 | 0.233435343 | 0.117144687 | 0.71247598 |
| 0.918155291 | 0.519535976 | 0.525443029 | 0.08063483 | 0.181552914 | 0.093587645 | 0.670201409 |
| 0.868977297 | 0.565297844 | 0.285673618 | 0.074158423 | 0.202405523 | 0.088392285 | 0.715678599 |
| 0.903992598 | 0.535549071 | 0.372215501 | 0.099138851 | 0.067610846 | 0.099067682 | 0.727208028 |
| 0.927691979 | 0.391217707 | 0.31022703 | 0.136858587 | 0.069745926 | 0.090740873 | 0.605366166 |
| 0.900576471 | 0.589210732 | 0.238488364 | 0.095865063 | 0.251939364 | 0.145114227 | 0.695537684 |
| 0.92662444 | 0.811045477 | 0.272151448 | 0.053163476 | 0.227812967 | 0.080990677 | 0.70009252 |
| 0.919507508 | 0.469361611 | 0.251583517 | 0.129670486 | 0.123692264 | 0.098925343 | 0.625222404 |
| 0.93075226 | 0.8313999 | 0.300192157 | 0.123122909 | 0.229948046 | 0.135292862 | 0.74564088 |
| 0.938296207 | 0.689274785 | 0.257917586 | 0.079353783 | 0.251868194 | 0.10440538 | 0.743221123 |
| 0.907195217 | 0.608141769 | 0.312789125 | 0.195857946 | 0.236139777 | 0.092448936 | 0.734040282 |
| 0.845420255 | 0.619884706 | 0.22546438 | 0.10646929 | 0.191374279 | 0.074016084 | 0.707067113 |
| 0.939150238 | 0.625720589 | 0.262828268 | 0.120418476 | 0.340829834 | 0.054231016 | 0.749270515 |

| 0.928474842 | 0.577254288 | 0.275425237 | 0.204825279 | 0.153654544 | 0.092377767 | 0.738239271 |
| --- | --- | --- | --- | --- | --- | --- |
| 0.927549641 | 0.651199203 | 0.254928475 | 0.082983418 | 0.231513771 | 0.084122127 | 0.708276991 |
| 0.850829122 | 0.568500463 | 0.318625009 | 0.10654046 | 0.290228453 | 0.10340901 | 0.737812255 |
| 0.912817593 | 0.505871468 | 0.302469575 | 0.086328375 | 0.154223899 | 0.076435841 | 0.696818732 |
| 0.909614974 | 0.510070458 | 0.346808056 | 0.187104121 | 0.175218846 | 0.062201979 | 0.676748986 |
| 0.907551064 | 0.609778663 | 0.42708704 | 0.20681802 | 0.252935734 | 0.166749698 | 0.692192726 |
| 0.918084122 | 0.82933599 | 0.437833606 | 0.173226105 | 0.345740517 | 0.194648068 | 0.736175361 |
| 0.933029678 | 0.84179062 | 0.397480606 | 0.155433777 | 0.307736104 | 0.156999502 | 0.731051171 |
| 0.947405879 | 0.786776742 | 0.384883638 | 0.17998719 | 0.286741157 | 0.162693047 | 0.689345954 |
| 0.932887339 | 0.741299552 | 0.456124119 | 0.180770052 | 0.275354067 | 0.212938581 | 0.663297986 |
| 0.949967974 | 0.865632339 | 0.385025977 | 0.106398121 | 0.385595331 | 0.157640026 | 0.709273361 |
| 0.913529286 | 0.849975091 | 0.439755178 | 0.18717529 | 0.309372998 | 0.202192015 | 0.793324319 |
| 0.939933101 | 0.544089389 | 0.374208241 | 0.17991602 | 0.257561739 | 0.208027898 | 0.551277489 |
| 0.839797879 | 0.884848054 | 0.382036866 | 0.177638602 | 0.426660024 | 0.177709772 | 0.581595616 |
| 0.926695609 | 0.755391075 | 0.397622945 | 0.216639385 | 0.271368586 | 0.182833962 | 0.704718525 |
| 0.950110312 | 0.510355135 | 0.388228596 | 0.258344602 | 0.19856238 | 0.18098356 | 0.733470927 |
| 0.920930895 | 0.727208028 | 0.358906839 | 0.141982777 | 0.323464522 | 0.181481745 | 0.628994378 |
| 0.930965768 | 0.811258985 | 0.433065262 | 0.165895666 | 0.244680094 | 0.185396057 | 0.74870116 |
| 0.929257704 | 0.63184115 | 0.432851754 | 0.162835385 | 0.221905914 | 0.15507793 | 0.615970394 |
| 0.871610562 | 0.594050246 | 0.413778379 | 0.152871682 | 0.248950253 | 0.152800512 | 0.668920362 |
| 0.939363746 | 0.854102911 | 0.419471924 | 0.570777881 | 0.351007046 | 0.18411501 | 0.71354352 |
| 0.900149456 | 0.801081774 | 0.421749342 | 0.173368444 | 0.319977226 | 0.18511138 | 0.683367732 |
| 0.898085546 | 0.331364316 | 0.339691125 | 0.252508718 | 0.190164401 | 0.177353925 | 0.460963632 |
| 0.876877091 | 0.721372144 | 0.390078998 | 0.358123977 | 0.178777311 | 0.160202121 | 0.536830119 |
| 0.903352075 | 0.823784784 | 0.404953384 | 0.112234005 | 0.333784072 | 0.160557967 | 0.763433208 |
| 0.92968472 | 0.83453135 | 0.332076009 | 0.184257348 | 0.298697602 | 0.237207316 | 0.765852964 |
| 0.799943065 | 0.731762864 | 0.436694897 | 0.206889189 | 0.365525585 | 0.189025692 | 0.772258202 |
| 0.91004199 | 0.574122838 | 0.453206177 | 0.212369226 | 0.251654686 | 0.182905131 | 0.653476621 |
| 0.904277276 | 0.67425806 | 0.433421109 | 0.22126539 | 0.278485517 | 0.140772899 | 0.630987118 |
| 0.929969397 | 0.701800584 | 0.427371717 | 0.197210163 | 0.275567575 | 0.176072877 | 0.501031955 |
| 0.912888762 | 0.814532773 | 0.481745072 | 0.14895737 | 0.256992385 | 0.141271084 | 0.719592912 |
| 0.948473418 | 0.199772258 | 0.354850189 | 0.191374279 | 0.226531919 | 0.192512988 | 0.477688421 |
| 0.906839371 | 0.51704505 | 0.323820369 | 0.166820867 | 0.234147036 | 0.161198491 | 0.524019643 |
| 0.927478471 | 0.81175717 | 0.380969326 | 0.131165042 | 0.347946765 | 0.173795459 | 0.762579176 |
| 0.834673689 | 0.807487012 | 0.435840865 | 0.180343036 | 0.338054231 | 0.223613978 | 0.72179916 |
| 0.8511138 | 0.572628283 | 0.380613479 | 0.166180343 | 0.269162337 | 0.165824496 | 0.707209451 |
| 0.94726354 | 0.562237563 | 0.411216284 | 0.172585581 | 0.223115792 | 0.176215216 | 0.555974664 |
| 0.93174863 | 0.783929969 | 0.427300548 | 0.155504946 | 0.364742723 | 0.216425877 | 0.795744075 |
| 0.920148032 | 0.479325315 | 0.370934453 | 0.182762793 | 0.247740374 | 0.193794036 | 0.677176002 |
| 0.880293218 | 0.413920717 | 0.400398548 | 0.106825137 | 0.180271867 | 0.158778735 | 0.669987901 |
| 0.898227884 | 0.639171589 | 0.342822575 | 0.187815814 | 0.237634332 | 0.173154936 | 0.639385097 |
| 0.872749271 | 0.732688065 | 0.451213437 | 0.150451925 | 0.272934311 | 0.154650915 | 0.461105971 |
| 0.903636752 | 0.747491282 | 0.379688278 | 0.176642232 | 0.296847199 | 0.199202904 | 0.64835243 |
| 0.863924276 | 0.192228311 | 0.273930681 | 0.211017009 | 0.254786136 | 0.207031528 | 0.492847484 |
| 0.955590349 | 0.868977297 | 0.372357839 | 0.143619671 | 0.296420184 | 0.174151306 | 0.788342467 |
| 0.907266387 | 0.355206035 | 0.300192157 | 0.189879724 | 0.206960359 | 0.183260978 | 0.68151733 |
| 0.925272223 | 0.832965625 | 0.351932247 | 0.148388015 | 0.290157284 | 0.142409793 | 0.658458473 |
| 0.901430503 | 0.612767775 | 0.392783432 | 0.178350295 | 0.266956089 | 0.148032169 | 0.600597822 |
| 0.869831329 | 0.285815956 | 0.417692691 | 0.198206533 | 0.237990179 | 0.195715607 | 0.564159135 |
| 0.844281546 | 0.635186108 | 0.423101559 | 0.181695253 | 0.226745427 | 0.165183973 | 0.646786706 |

| 0.920575048 | 0.815457975 | 0.347163903 | 0.24297203 | 0.369012882 | 0.199843428 | 0.732545726 |
| --- | --- | --- | --- | --- | --- | --- |
| 0.933954879 | 0.584228881 | 0.433065262 | 0.148601523 | 0.232438972 | 0.166607359 | 0.565226674 |
| 0.917799445 | 0.795317059 | 0.391146538 | 0.183118639 | 0.297131877 | 0.20368657 | 0.706853605 |
| 0.920432709 | 0.850473276 | 0.356914099 | 0.15507793 | 0.328659882 | 0.195146253 | 0.740587859 |
| 0.873389794 | 0.739306811 | 0.421607003 | 0.100633407 | 0.246672835 | 0.235570422 | 0.59668351 |
| 0.945555476 | 0.667781653 | 0.351789908 | 0.198989396 | 0.282755676 | 0.185040211 | 0.630488933 |
| 0.938865561 | 0.818233578 | 0.432424738 | 0.182833962 | 0.324460892 | 0.202974877 | 0.60913814 |
| 0.902426874 | 0.558750267 | 0.414205395 | 0.174222475 | 0.263966977 | 0.180485375 | 0.66493488 |
| 0.941142979 | 0.704149171 | 0.372357839 | 0.201480322 | 0.294783289 | 0.184684364 | 0.702298769 |
| 0.947548217 | 0.803715038 | 0.381538681 | 0.201836168 | 0.326026617 | 0.189168031 | 0.716888478 |
| 0.876734752 | 0.520674685 | 0.390007829 | 0.201836168 | 0.247526866 | 0.183687994 | 0.709202192 |
| 0.935022418 | 0.740872536 | 0.429293289 | 0.200910967 | 0.234360544 | 0.176144047 | 0.597537542 |
| 0.932246815 | 0.503095865 | 0.436481389 | 0.15102128 | 0.161483168 | 0.170236994 | 0.585082912 |
| 0.926980286 | 0.529997865 | 0.383033236 | 0.225179703 | 0.278485517 | 0.196925486 | 0.418973739 |
| 0.962707281 | 0.728844922 | 0.407800157 | 0.188741015 | 0.320688919 | 0.217849263 | 0.727065689 |
| 0.929969397 | 0.739093303 | 0.393210448 | 0.171518041 | 0.349868337 | 0.149313216 | 0.662515123 |
| 0.930609921 | 0.376129813 | 0.29371575 | 0.175574692 | 0.25236638 | 0.229521031 | 0.632695182 |
| 0.924062344 | 0.527222262 | 0.382819728 | 0.175290015 | 0.214433136 | 0.163120063 | 0.651056864 |
| 0.880293218 | 0.65255142 | 0.458259199 | 0.834744858 | 0.211871041 | 0.157568856 | 0.537043627 |
| 0.93281617 | 0.582663156 | 0.37121913 | 0.210732332 | 0.255568999 | 0.173368444 | 0.617963134 |
| 0.883353498 | 0.780157996 | 0.385880009 | 0.181908761 | 0.258913956 | 0.177567433 | 0.561597039 |
| 0.92762081 | 0.748487652 | 0.371859654 | 0.150451925 | 0.3443883 | 0.226176073 | 0.624439542 |
| 0.88214362 | 0.788484805 | 0.459824923 | 0.174649491 | 0.36922639 | 0.212867412 | 0.694541314 |
| 0.819301117 | 0.747206605 | 0.353782649 | 0.200412782 | 0.47256423 | 0.165183973 | 0.613052452 |
| 0.951960715 | 0.317130453 | 0.265319194 | 0.164329941 | 0.219984343 | 0.181908761 | 0.623158494 |
| 0.904775461 | 0.507010177 | 0.405807416 | 0.193509359 | 0.250373639 | 0.165397481 | 0.674827414 |
| 0.947121201 | 0.675610277 | 0.408725358 | 0.179275496 | 0.21713757 | 0.139491851 | 0.566507722 |
| 0.939861932 | 0.771688848 | 0.350437691 | 0.154793253 | 0.354352003 | 0.15095011 | 0.650629848 |
| 0.919863355 | 0.71147961 | 0.367589495 | 0.165397481 | 0.169169454 | 0.188527507 | 0.73318625 |
| 0.938723223 | 0.829905345 | 0.392142908 | 0.157711195 | 0.347163903 | 0.166678528 | 0.718454203 |
| 0.932246815 | 0.397978792 | 0.390434844 | 0.14269447 | 0.166251512 | 0.182691623 | 0.630346595 |
| 0.909187958 | 0.842644652 | 0.469005765 | 0.171873888 | 0.344957654 | 0.171019856 | 0.712902996 |
| 0.885488577 | 0.673475197 | 0.496690627 | 0.170735179 | 0.272791972 | 0.224254501 | 0.733043911 |
| 0.915095011 | 0.822717244 | 0.393637464 | 0.18717529 | 0.279980073 | 0.173581951 | 0.6152587 |
| 0.915664366 | 0.780086827 | 0.37954594 | 0.20162266 | 0.289232083 | 0.184542025 | 0.660095367 |
| 0.897943207 | 0.626147605 | 0.404668707 | 0.495907765 | 0.392071739 | 0.195786777 | 0.63077361 |
| 0.907551064 | 0.15301402 | 0.18304747 | 0.518326098 | 0.130951534 | 0.151804142 | 0.537612981 |
| 0.85317771 | 0.334211088 | 0.29264821 | 0.492705146 | 0.265888549 | 0.165112803 | 0.535264394 |
| 0.838801509 | 0.394206818 | 0.253078073 | 0.505159775 | 0.319621379 | 0.145754751 | 0.557042203 |
| 0.874813181 | 0.581310939 | 0.304960501 | 0.477617251 | 0.350081845 | 0.141769269 | 0.58842787 |
| 0.887694826 | 0.630133087 | 0.276563946 | 0.465874315 | 0.365739093 | 0.161411999 | 0.625364743 |
| 0.876378905 | 0.294569782 | 0.344601808 | 0.452352146 | 0.183545655 | 0.168457761 | 0.595829478 |
| 0.883495837 | 0.568642801 | 0.269802861 | 0.519607145 | 0.287808697 | 0.141128745 | 0.569710341 |
| 0.906839371 | 0.295850829 | 0.304319977 | 0.482314426 | 0.197708348 | 0.165041634 | 0.583588357 |
| 0.868336773 | 0.378264892 | 0.313571988 | 0.510782151 | 0.226247242 | 0.159134581 | 0.509501103 |
| 0.883994022 | 0.211515195 | 0.261689559 | 0.470784998 | 0.16134083 | 0.1686001 | 0.53882286 |
| 0.83453135 | 0.410006405 | 0.279126041 | 0.457333998 | 0.215856523 | 0.147462814 | 0.644153441 |
| 0.843285175 | 0.241335136 | 0.259625649 | 0.545299267 | 0.131165042 | 0.155362608 | 0.512134368 |
| 0.857092022 | 0.239342396 | 0.244822433 | 0.533058145 | 0.177780941 | 0.160629137 | 0.524446659 |
| 0.816525514 | 0.292434702 | 0.25542666 | 0.589139563 | 0.274642374 | 0.139136005 | 0.596612341 |

| 0.885773255 | 0.554124262 | 0.306597395 | 0.50779304 | 0.280193581 | 0.168386592 | 0.602234716 |
| --- | --- | --- | --- | --- | --- | --- |
| 0.941997011 | 0.570564373 | 0.326026617 | 0.432068892 | 0.372571347 | 0.163760586 | 0.576898441 |
| 0.909828482 | 0.621948616 | 0.265319194 | 0.488506156 | 0.416696321 | 0.180556544 | 0.554907124 |
| 0.881360757 | 0.548217209 | 0.285246602 | 0.481389225 | 0.291936517 | 0.17785211 | 0.675539108 |
| 0.925058715 | 0.793537826 | 0.286669988 | 0.498683368 | 0.451996299 | 0.170521671 | 0.641306669 |
| 0.914383318 | 0.564870828 | 0.258273432 | 0.456195289 | 0.29784357 | 0.124475126 | 0.643299409 |
| 0.843996869 | 0.56152587 | 0.261689559 | 0.524233151 | 0.286385311 | 0.178848481 | 0.616682087 |
| 0.872749271 | 0.637321187 | 0.29371575 | 0.510995659 | 0.334353427 | 0.183759163 | 0.589353071 |
| 0.939861932 | 0.835100705 | 0.306597395 | 0.500889616 | 0.393637464 | 0.194861576 | 0.704078002 |
| 0.903209736 | 0.611842573 | 0.259696819 | 0.508718241 | 0.350793538 | 0.153298698 | 0.603800441 |
| 0.882997651 | 0.605650843 | 0.27094157 | 0.5201765 | 0.322895168 | 0.166820867 | 0.569710341 |
| 0.894171233 | 0.489289019 | 0.241762152 | 0.517472066 | 0.375773966 | 0.166892036 | 0.719308234 |
| 0.89139563 | 0.560244822 | 0.264607501 | 0.469717458 | 0.283823215 | 0.147462814 | 0.575617394 |
| 0.881147249 | 0.764144901 | 0.282826845 | 0.606718383 | 0.432638246 | 0.173226105 | 0.649206462 |
| 0.90904562 | 0.582876664 | 0.303110099 | 0.517329727 | 0.309871184 | 0.152729343 | 0.541384955 |
| 0.907195217 | 0.183901502 | 0.255141983 | 0.533912177 | 0.212582734 | 0.160700306 | 0.557967404 |
| 0.915877873 | 0.337342538 | 0.249946623 | 0.626147605 | 0.234289374 | 0.167105544 | 0.688064906 |
| 0.869048466 | 0.544801082 | 0.298768771 | 0.477830759 | 0.22439684 | 0.158280549 | 0.560244822 |
| 0.883851683 | 0.19023557 | 0.312575617 | 0.573268806 | 0.163974094 | 0.147747491 | 0.528716817 |
| 0.906981709 | 0.134794677 | 0.273574835 | 0.436766066 | 0.175432354 | 0.159988613 | 0.565938367 |
| 0.877090599 | 0.711621949 | 0.288876237 | 0.494555548 | 0.251441179 | 0.15201765 | 0.619742367 |
| 0.920219201 | 0.10547292 | 0.233933528 | 0.4322824 | 0.143833179 | 0.177140417 | 0.425663654 |
| 0.874670842 | 0.121343677 | 0.21720874 | 0.540673262 | 0.173653121 | 0.180271867 | 0.647213721 |
| 0.901145826 | 0.316561099 | 0.215643015 | 0.485303537 | 0.199772258 | 0.164685787 | 0.52330795 |
| 0.895879297 | 0.26375347 | 0.272791972 | 0.54601096 | 0.251156501 | 0.162550708 | 0.503878727 |
| 0.874243826 | 0.375275781 | 0.233221835 | 0.607287738 | 0.239982919 | 0.196569639 | 0.505942638 |
| 0.897373852 | 0.266173226 | 0.247455697 | 0.456693474 | 0.167319052 | 0.152159989 | 0.629990748 |
| 0.843641022 | 0.195857946 | 0.281189951 | 0.548928902 | 0.146181766 | 0.154010391 | 0.495053733 |
| 0.912604085 | 0.257917586 | 0.258842787 | 0.42395559 | 0.271439755 | 0.17472066 | 0.556615188 |
| 0.89452708 | 0.1820511 | 0.249021422 | 0.469930966 | 0.157568856 | 0.147605153 | 0.544587574 |
| 0.938367376 | 0.776030176 | 0.359618532 | 0.503238204 | 0.352572771 | 0.174934168 | 0.641235499 |
| 0.867269234 | 0.40125258 | 0.23991175 | 0.600953669 | 0.253505089 | 0.183687994 | 0.544445235 |
| 0.91723009 | 0.449434204 | 0.354067326 | 0.496121273 | 0.255568999 | 0.149455555 | 0.612554267 |
| 0.886342609 | 0.424240268 | 0.274500036 | 0.369582236 | 0.236282115 | 0.132588428 | 0.561027685 |
| 0.900861149 | 0.181339406 | 0.260123835 | 0.490854743 | 0.190662586 | 0.142409793 | 0.484876521 |
| 0.919222831 | 0.242545015 | 0.272222618 | 0.548430717 | 0.225108533 | 0.184328518 | 0.560814177 |
| 0.902640381 | 0.134937015 | 0.214504306 | 0.402177781 | 0.118639243 | 0.130097502 | 0.474414632 |
| 0.889474059 | 0.310796385 | 0.260337343 | 0.528076294 | 0.312860295 | 0.172229735 | 0.600384314 |
| 0.844423884 | 0.488363818 | 0.295637321 | 0.420325955 | 0.264821009 | 0.175574692 | 0.647854245 |
| 0.901928688 | 0.660735891 | 0.267027258 | 0.558963775 | 0.415059426 | 0.156003131 | 0.704576187 |
| 0.876734752 | 0.297985908 | 0.318980855 | 0.605366166 | 0.23372002 | 0.150665433 | 0.576898441 |
| 0.89039926 | 0.645790335 | 0.271439755 | 0.516618034 | 0.316062914 | 0.180485375 | 0.583303679 |
| 0.875311366 | 0.508860579 | 0.295067967 | 0.525514198 | 0.290726639 | 0.186321258 | 0.614902854 |
| 0.88413636 | 0.186677105 | 0.27400185 | 0.517187389 | 0.136075724 | 0.135008184 | 0.544872251 |
| 0.885844424 | 0.625364743 | 0.329798591 | 0.448081987 | 0.376841506 | 0.149028539 | 0.56565369 |
| 0.857590207 | 0.22745712 | 0.263966977 | 0.504092235 | 0.1820511 | 0.155718454 | 0.566080706 |
| 0.901003487 | 0.181410576 | 0.272364956 | 0.474414632 | 0.17891965 | 0.17265675 | 0.550708135 |
| 0.902996228 | 0.273290157 | 0.273717173 | 0.499110384 | 0.180556544 | 0.170094655 | 0.590420611 |
| 0.885702085 | 0.448366664 | 0.315707067 | 0.522026902 | 0.253291581 | 0.171731549 | 0.632979859 |
| 0.878371646 | 0.628852039 | 0.25443029 | 0.491993452 | 0.393210448 | 0.150024909 | 0.663653832 |

| 0.88726781 | 0.617963134 | 0.311365739 | 0.480464024 | 0.21927265 | 0.149882571 | 0.614191161 |
| --- | --- | --- | --- | --- | --- | --- |
| 0.880435556 | 0.296633692 | 0.285887125 | 0.451711622 | 0.238346025 | 0.137356772 | 0.549498256 |
| 0.866699879 | 0.478471283 | 0.276563946 | 0.418546723 | 0.282969184 | 0.155860793 | 0.611557896 |
| 0.853889403 | 0.082129386 | 0.25129884 | 0.548644225 | 0.152800512 | 0.166607359 | 0.393210448 |
| 0.866415202 | 0.191658957 | 0.216070031 | 0.459967262 | 0.152088819 | 0.151305957 | 0.5139136 |
| 0.928901858 | 0.276421607 | 0.316276422 | 0.552700875 | 0.170236994 | 0.134937015 | 0.459540246 |
| 0.907693403 | 0.549000071 | 0.261476052 | 0.4860864 | 0.324105046 | 0.150024909 | 0.620098214 |
| 0.721016298 | 0.529784357 | 0.341114511 | 0.480464024 | 0.231584941 | 0.201266814 | 0.664721372 |
| 0.895879297 | 0.141769269 | 0.238132517 | 0.395203188 | 0.142125116 | 0.159775105 | 0.46744004 |
| 0.87488435 | 0.808483382 | 0.324105046 | 0.494840225 | 0.328090527 | 0.17066401 | 0.584513558 |
| 0.852181339 | 0.59155932 | 0.352786279 | 0.492135791 | 0.420112448 | 0.145897089 | 0.62671696 |
| 0.888620027 | 0.153369867 | 0.231584941 | 0.500889616 | 0.129456978 | 0.154579745 | 0.596185325 |
| 0.851184969 | 0.54601096 | 0.251797025 | 0.397907622 | 0.356273575 | 0.165397481 | 0.577183119 |
| 0.886556117 | 0.664863711 | 0.283965554 | 0.484378336 | 0.337342538 | 0.157640026 | 0.610988542 |
| 0.903209736 | 0.782648922 | 0.249590776 | 0.526225891 | 0.419685432 | 0.149384385 | 0.576115579 |
| 0.883709345 | 0.235641591 | 0.354992527 | 0.48402249 | 0.130097502 | 0.182833962 | 0.607358907 |
| 0.894598249 | 0.199772258 | 0.231940787 | 0.504519251 | 0.13137855 | 0.164899295 | 0.500391431 |
| 0.895167604 | 0.664436695 | 0.317699808 | 0.574478685 | 0.380684649 | 0.228453491 | 0.634545584 |
| 0.914739164 | 0.213579105 | 0.257704078 | 0.518539606 | 0.177638602 | 0.18610775 | 0.602732902 |
| 0.870044837 | 0.209522454 | 0.274784713 | 0.547434346 | 0.166963205 | 0.173083766 | 0.545512775 |
| 0.885630916 | 0.175361184 | 0.287666358 | 0.528787987 | 0.168457761 | 0.209380115 | 0.60493915 |
| 0.795530567 | 0.752544303 | 0.229236353 | 0.588854886 | 0.464735606 | 0.223898655 | 0.62465305 |
| 0.908262757 | 0.150238417 | 0.259269803 | 0.506796669 | 0.171091026 | 0.154366237 | 0.500676108 |
| 0.868194435 | 0.237349655 | 0.275994591 | 0.553056722 | 0.177425094 | 0.184613195 | 0.514909971 |
| 0.916162551 | 0.099067682 | 0.214148459 | 0.458259199 | 0.162266031 | 0.154223899 | 0.446800939 |
| 0.894669419 | 0.114013237 | 0.196000285 | 0.485801722 | 0.146181766 | 0.147747491 | 0.469290442 |
| 0.883424667 | 0.116788841 | 0.1686001 | 0.493701516 | 0.158778735 | 0.125257989 | 0.460963632 |
| 0.883068821 | 0.512703722 | 0.276635115 | 0.59561597 | 0.267169596 | 0.148672692 | 0.656181055 |
| 0.903992598 | 0.63803288 | 0.24097929 | 0.546366807 | 0.258415771 | 0.171091026 | 0.677176002 |
| 0.887694826 | 0.584513558 | 0.396911252 | 0.510283966 | 0.276279268 | 0.140274714 | 0.633762722 |
| 0.886556117 | 0.102199132 | 0.187388798 | 0.510212796 | 0.187104121 | 0.152373497 | 0.462814035 |
| 0.884776884 | 0.141057576 | 0.208099068 | 0.489644865 | 0.133228952 | 0.147035798 | 0.520532346 |
| 0.91310227 | 0.734823144 | 0.260195004 | 0.62458188 | 0.383887268 | 0.196071454 | 0.687282044 |
| 0.877873461 | 0.606931891 | 0.407017294 | 0.638673404 | 0.290086115 | 0.16959647 | 0.611059711 |
| 0.782933599 | 0.665290727 | 0.337200199 | 0.382036866 | 0.385880009 | 0.187388798 | 0.589210732 |
| 0.852608355 | 0.560885346 | 0.282613337 | 0.496477119 | 0.275638745 | 0.176286385 | 0.579816383 |
| 0.86349726 | 0.514482955 | 0.304106469 | 0.436552559 | 0.205323465 | 0.166607359 | 0.633335706 |
| 0.895238773 | 0.58636396 | 0.289160914 | 0.490071881 | 0.469148103 | 0.184328518 | 0.669205039 |
| 0.896092805 | 0.319336702 | 0.341826205 | 0.415344104 | 0.211444025 | 0.166892036 | 0.63084478 |
| 0.870258345 | 0.224681517 | 0.26474984 | 0.389438474 | 0.12205537 | 0.210091808 | 0.592769198 |
| 0.883282329 | 0.656038716 | 0.332076009 | 0.390434844 | 0.392498755 | 0.161625507 | 0.627072806 |
| 0.896519821 | 0.334424596 | 0.271510924 | 0.514838802 | 0.260906697 | 0.147533983 | 0.482029749 |
| 0.880791403 | 0.61120205 | 0.319906057 | 0.364742723 | 0.37541812 | 0.164187602 | 0.591772828 |
| 0.902925059 | 0.254145612 | 0.267169596 | 0.50985695 | 0.121913031 | 0.153156359 | 0.510924489 |
| 0.907337556 | 0.216070031 | 0.350081845 | 0.505800299 | 0.14895737 | 0.146181766 | 0.587787346 |
| 0.858942424 | 0.66593125 | 0.305529856 | 0.416055797 | 0.40224895 | 0.155006761 | 0.638388727 |
| 0.874742011 | 0.191160771 | 0.226674258 | 0.504163405 | 0.134367661 | 0.177567433 | 0.522596256 |
| 0.889972244 | 0.877873461 | 0.30503167 | 0.687424383 | 0.380471141 | 0.234858729 | 0.589922425 |
| 0.868977297 | 0.651910896 | 0.264465163 | 0.557042203 | 0.192655327 | 0.192157142 | 0.550209949 |
| 0.890683937 | 0.849761583 | 0.296562522 | 0.4932745 | 0.248452067 | 0.17272792 | 0.704505017 |

| 0.826702726 | 0.116788841 | 0.223827486 | 0.536403103 | 0.162266031 | 0.178706142 | 0.426304178 |
| --- | --- | --- | --- | --- | --- | --- |
| 0.80456907 | 0.381823358 | 0.382962067 | 0.487794463 | 0.235214575 | 0.154152729 | 0.553839584 |
| 0.822076721 | 0.572272436 | 0.311863924 | 0.492705146 | 0.288164543 | 0.163831756 | 0.538111166 |
| 0.892676678 | 0.814461604 | 0.281403459 | 0.518254928 | 0.456622304 | 0.183260978 | 0.542594833 |
| 0.848267027 | 0.305601025 | 0.286314141 | 0.499252722 | 0.266173226 | 0.151448295 | 0.508504733 |
| 0.932673831 | 0.432353569 | 0.298626432 | 0.528218632 | 0.318126824 | 0.168813608 | 0.632624013 |
| 0.907266387 | 0.179417835 | 0.225179703 | 0.57191659 | 0.247028681 | 0.151234788 | 0.480606363 |
| 0.899010747 | 0.153583375 | 0.238630702 | 0.678314711 | 0.207600882 | 0.141555761 | 0.509145257 |
| 0.889189382 | 0.777524731 | 0.307451427 | 0.522382749 | 0.43534268 | 0.186534766 | 0.718098356 |
| 0.87075653 | 0.36296349 | 0.244680094 | 0.633122198 | 0.312006263 | 0.193651697 | 0.556686357 |
| 0.90797808 | 0.796099922 | 0.328446374 | 0.54287951 | 0.415059426 | 0.187531137 | 0.732545726 |
| 0.877731122 | 0.71254715 | 0.257419401 | 0.488648495 | 0.394135649 | 0.140132375 | 0.666215928 |
| 0.860223472 | 0.214006121 | 0.245889972 | 0.517258558 | 0.154295068 | 0.18197993 | 0.485161198 |
| 0.915095011 | 0.38367376 | 0.310369369 | 0.500818447 | 0.178563803 | 0.146039428 | 0.641804854 |
| 0.90897445 | 0.416055797 | 0.292150025 | 0.565724859 | 0.366664294 | 0.154650915 | 0.532631129 |
| 0.898797239 | 0.471354352 | 0.230446232 | 0.538111166 | 0.265176856 | 0.167319052 | 0.570350865 |
| 0.938580884 | 0.686712689 | 0.220055512 | 0.564514981 | 0.391644723 | 0.154935592 | 0.667710483 |
| 0.897943207 | 0.105330581 | 0.18511138 | 0.600384314 | 0.168173084 | 0.15920575 | 0.414347733 |
| 0.930609921 | 0.314924205 | 0.27812967 | 0.517685574 | 0.23165611 | 0.151946481 | 0.631342965 |
| 0.842502313 | 0.593979076 | 0.287951036 | 0.427158209 | 0.40018504 | 0.156714825 | 0.654401822 |
| 0.894740588 | 0.288876237 | 0.261049036 | 0.513771262 | 0.225037364 | 0.152658174 | 0.543520034 |
| 0.859227101 | 0.63184115 | 0.288591559 | 0.551633336 | 0.33819657 | 0.204042417 | 0.668920362 |
| 0.874243826 | 0.479040638 | 0.248736745 | 0.67945342 | 0.301900221 | 0.18304747 | 0.625151235 |
| 0.867126895 | 0.320902427 | 0.298697602 | 0.445946908 | 0.240338766 | 0.160771475 | 0.593907907 |
| 0.91317344 | 0.673332859 | 0.260764358 | 0.572841791 | 0.443455982 | 0.172087396 | 0.693971959 |
| 0.896519821 | 0.636040139 | 0.238346025 | 0.54088677 | 0.393922141 | 0.163618248 | 0.545726283 |
| 0.879225678 | 0.535477902 | 0.23065974 | 0.540032738 | 0.401537257 | 0.154366237 | 0.665788912 |
| 0.846274287 | 0.691196356 | 0.405166892 | 0.631058288 | 0.335563305 | 0.161554338 | 0.639527436 |
| 0.895167604 | 0.660949399 | 0.416696321 | 0.55846559 | 0.324816739 | 0.175361184 | 0.540246246 |
| 0.852679525 | 0.740232012 | 0.279268379 | 0.665788912 | 0.35776813 | 0.222190591 | 0.787773112 |
| 0.922567789 | 0.499181553 | 0.28439257 | 0.468934595 | 0.432567077 | 0.17891965 | 0.57704078 |
| 0.903565583 | 0.105330581 | 0.223827486 | 0.648637108 | 0.20162266 | 0.175076507 | 0.51811259 |
| 0.916874244 | 0.376129813 | 0.323251014 | 0.472066045 | 0.262543591 | 0.144900719 | 0.555618817 |
| 0.859725286 | 0.790975731 | 0.278272009 | 0.531990606 | 0.322396982 | 0.189025692 | 0.588926055 |
| 0.826702726 | 0.193153512 | 0.331079638 | 0.654045975 | 0.156501317 | 0.180698883 | 0.675966123 |
| 0.763860223 | 0.570564373 | 0.284819586 | 0.579816383 | 0.381965696 | 0.176499893 | 0.639669774 |
| 0.868052096 | 0.609921002 | 0.265888549 | 0.545726283 | 0.371930823 | 0.181339406 | 0.630702441 |
| 0.845776101 | 0.467297701 | 0.360401395 | 0.558679098 | 0.26062202 | 0.155789624 | 0.610490357 |
| 0.88826418 | 0.385310654 | 0.307736104 | 0.448081987 | 0.21820511 | 0.140559391 | 0.551490997 |
| 0.900078286 | 0.763931393 | 0.264180485 | 0.52537186 | 0.410718098 | 0.218632126 | 0.568073447 |
| 0.867980927 | 0.322325813 | 0.29165184 | 0.555903494 | 0.192940004 | 0.160130952 | 0.441178564 |
| 0.901288165 | 0.40224895 | 0.27606576 | 0.559177283 | 0.312717956 | 0.155006761 | 0.593338552 |
| 0.895167604 | 0.518397267 | 0.267311935 | 0.543804711 | 0.287381681 | 0.172443242 | 0.562878087 |
| 0.862358551 | 0.322396982 | 0.264393993 | 0.552060352 | 0.276421607 | 0.185538396 | 0.595971817 |
| 0.868906128 | 0.588712547 | 0.309444168 | 0.542096648 | 0.307878443 | 0.191872465 | 0.618817166 |
| 0.895025265 | 0.684933457 | 0.277062131 | 0.557326881 | 0.400042702 | 0.181552914 | 0.610276849 |
| 0.89552345 | 0.282898014 | 0.274428866 | 0.628140346 | 0.223186962 | 0.177994449 | 0.570706711 |
| 0.906981709 | 0.331293146 | 0.296349014 | 0.54188314 | 0.219984343 | 0.179702512 | 0.627926838 |
| 0.9255569 | 0.747775959 | 0.286812327 | 0.486513415 | 0.356273575 | 0.148672692 | 0.647213721 |
| 0.872891609 | 0.56878514 | 0.27300548 | 0.555618817 | 0.306739734 | 0.163831756 | 0.591345812 |

| 0.892107323 | 0.233435343 | 0.234645221 | 0.597252865 | 0.176926909 | 0.168742438 | 0.547220838 |
| --- | --- | --- | --- | --- | --- | --- |
| 0.899864778 | 0.128602946 | 0.232011956 | 0.51184969 | 0.152159989 | 0.161554338 | 0.500960786 |
| 0.904206106 | 0.333072379 | 0.263966977 | 0.516902712 | 0.239200057 | 0.156857163 | 0.639954452 |
| 0.903423244 | 0.608284108 | 0.292505871 | 0.539036368 | 0.429222119 | 0.143334994 | 0.625720589 |
| 0.889545228 | 0.154010391 | 0.253006903 | 0.460109601 | 0.145612412 | 0.132801936 | 0.69489716 |
| 0.838801509 | 0.344032453 | 0.444238844 | 0.703437478 | 0.224823856 | 0.14788983 | 0.5677176 |
| 0.879368017 | 0.346096363 | 0.325955448 | 0.477901929 | 0.28752402 | 0.177069248 | 0.605437335 |
| 0.857519038 | 0.339477617 | 0.350081845 | 0.477901929 | 0.232225464 | 0.172799089 | 0.543377696 |
| 0.900007117 | 0.500747278 | 0.276635115 | 0.516262188 | 0.252010533 | 0.162550708 | 0.628140346 |
| 0.923137143 | 0.67219415 | 0.27200911 | 0.462742865 | 0.373638887 | 0.156003131 | 0.630702441 |
| 0.917657106 | 0.563732119 | 0.34026048 | 0.531207743 | 0.2636823 | 0.164899295 | 0.627784499 |
| 0.90278272 | 0.754465874 | 0.303892961 | 0.465091453 | 0.449291865 | 0.160202121 | 0.702369938 |
| 0.892036154 | 0.120703153 | 0.213721443 | 0.430930183 | 0.161554338 | 0.128460608 | 0.528289801 |
| 0.902071027 | 0.138068465 | 0.255782507 | 0.485232368 | 0.182478115 | 0.142125116 | 0.543021849 |
| 0.913529286 | 0.688847769 | 0.28126112 | 0.563518611 | 0.267240766 | 0.167105544 | 0.59255569 |
| 0.860294641 | 0.733826774 | 0.28752402 | 0.632125827 | 0.423101559 | 0.151661803 | 0.673902213 |
| 0.910967191 | 0.459682585 | 0.25848694 | 0.51704505 | 0.262116575 | 0.166963205 | 0.638744573 |
| 0.88107608 | 0.619315351 | 0.262970607 | 0.517116219 | 0.434488648 | 0.164899295 | 0.556686357 |
| 0.88107608 | 0.605793182 | 0.277773824 | 0.549427087 | 0.434488648 | 0.23685147 | 0.616468579 |
| 0.918866985 | 0.615756886 | 0.32474557 | 0.498042844 | 0.241192798 | 0.164614618 | 0.594192584 |
| 0.912319408 | 0.152373497 | 0.206391004 | 0.640310298 | 0.128816454 | 0.152942851 | 0.645576827 |

| Spliceosome | Systemic lupu | Viral carcinog | WNT target |
| --- | --- | --- | --- |
| 0.502455341 | 0.948758096 | 0.641306669 | 0.65461533 |
| 0.447939648 | 0.96690627 | 0.485588214 | 0.75489289 |
| 0.352928617 | 0.956657889 | 0.680236282 | 0.827699096 |
| 0.430360828 | 0.934239556 | 0.423172728 | 0.668208668 |
| 0.356985268 | 0.929613551 | 0.595331293 | 0.641235499 |
| 0.403743506 | 0.920646217 | 0.526795246 | 0.634830261 |
| 0.388086257 | 0.958935307 | 0.643726425 | 0.641947192 |
| 0.39093303 | 0.946409508 | 0.686072166 | 0.870329514 |
| 0.424524945 | 0.937798021 | 0.759732403 | 0.612696605 |
| 0.440466871 | 0.962351434 | 0.676820155 | 0.769838446 |
| 0.365596755 | 0.932958508 | 0.42915095 | 0.669418547 |
| 0.364600384 | 0.953526439 | 0.602804071 | 0.487652124 |
| 0.35983204 | 0.954380471 | 0.646430859 | 0.626788129 |
| 0.419044908 | 0.936801651 | 0.353996157 | 0.6152587 |
| 0.40637677 | 0.954095794 | 0.546153299 | 0.69283325 |
| 0.395843712 | 0.930325244 | 0.58010106 | 0.62671696 |
| 0.365739093 | 0.955875027 | 0.378122554 | 0.712262472 |
| 0.338410078 | 0.956159704 | 0.669133869 | 0.655825208 |
| 0.413564871 | 0.959789339 | 0.752117287 | 0.573339976 |
| 0.354992527 | 0.927691979 | 0.585225251 | 0.608426447 |
| 0.407728987 | 0.960999217 | 0.684221764 | 0.550209949 |
| 0.430431998 | 0.929400043 | 0.624083695 | 0.584655896 |
| 0.465803146 | 0.917585937 | 0.414988257 | 0.660878229 |
| 0.39705359 | 0.964486513 | 0.801935805 | 0.650060494 |
| 0.398975162 | 0.959860508 | 0.675467938 | 0.789125329 |
| 0.404739876 | 0.991673191 | 0.25642303 | 0.53775532 |
| 0.41057576 | 0.947690556 | 0.514055939 | 0.801366451 |
| 0.42815458 | 0.941000641 | 0.591203473 | 0.583090172 |
| 0.375062273 | 0.961497402 | 0.718240695 | 0.701515906 |
| 0.401679596 | 0.926766778 | 0.384883638 | 0.723507224 |
| 0.482029749 | 0.899793609 | 0.364956231 | 0.67838588 |
| 0.450003558 | 0.969681873 | 0.780300334 | 0.764998932 |
| 0.477830759 | 0.962707281 | 0.695893531 | 0.741726568 |
| 0.41263967 | 0.961070386 | 0.627357483 | 0.642303039 |
| 0.408013665 | 0.934951249 | 0.661661092 | 0.709059853 |
| 0.343036083 | 0.967546794 | 0.511351505 | 0.680805637 |
| 0.406092093 | 0.957725429 | 0.728631414 | 0.661732261 |
| 0.437904775 | 0.962209095 | 0.796171091 | 0.524517828 |
| 0.437975945 | 0.956942566 | 0.543093018 | 0.689132446 |
| 0.471923706 | 0.967617963 | 0.761226959 | 0.695253007 |
| 0.412426162 | 0.93068109 | 0.558038574 | 0.592128674 |
| 0.444808199 | 0.957440752 | 0.777097715 | 0.787346096 |
| 0.398192299 | 0.94313572 | 0.451569283 | 0.784784001 |
| 0.433776955 | 0.969610704 | 0.781581382 | 0.757312647 |
| 0.381823358 | 0.91829763 | 0.26055085 | 0.492918653 |
| 0.336203829 | 0.946124831 | 0.548857733 | 0.734396128 |
| 0.332503025 | 0.967190947 | 0.780585012 | 0.591061134 |
| 0.374208241 | 0.928973027 | 0.434702156 | 0.739662657 |

| 0.440324532 | 0.943776244 | 0.633691552 | 0.600526653 |
| --- | --- | --- | --- |
| 0.41363604 | 0.878656323 | 0.643512917 | 0.639527436 |
| 0.395630204 | 0.962280265 | 0.629350224 | 0.654971176 |
| 0.536331934 | 0.941783503 | 0.298128247 | 0.682656039 |
| 0.42295922 | 0.952103053 | 0.49014305 | 0.620596399 |
| 0.462102341 | 0.962991958 | 0.679026404 | 0.552487367 |
| 0.396128389 | 0.962351434 | 0.486798093 | 0.753398335 |
| 0.481318056 | 0.963418974 | 0.649846986 | 0.635186108 |
| 0.439897516 | 0.946196 | 0.698882642 | 0.698170949 |
| 0.291509501 | 0.958294783 | 0.485517045 | 0.750338054 |
| 0.41776386 | 0.960928048 | 0.658600811 | 0.651128034 |
| 0.345882855 | 0.968044979 | 0.59355206 | 0.6215216 |
| 0.45704932 | 0.977652836 | 0.578250658 | 0.564799658 |
| 0.505515622 | 0.967546794 | 0.641235499 | 0.678528219 |
| 0.474912818 | 0.973525016 | 0.714184044 | 0.591203473 |
| 0.444167675 | 0.959077646 | 0.777595901 | 0.700733044 |
| 0.469076934 | 0.987331863 | 0.864422461 | 0.67938225 |
| 0.472848908 | 0.956302043 | 0.510212796 | 0.562664579 |
| 0.433848125 | 0.980570778 | 0.747420112 | 0.673688705 |
| 0.444594691 | 0.939648424 | 0.4457334 | 0.44160558 |
| 0.436837236 | 0.959077646 | 0.602163547 | 0.673973383 |
| 0.475411003 | 0.932317984 | 0.497829336 | 0.603159917 |
| 0.448936019 | 0.95238773 | 0.429222119 | 0.563803288 |
| 0.363532845 | 0.933314355 | 0.791616255 | 0.531421251 |
| 0.482599103 | 0.873532133 | 0.532844637 | 0.721941499 |
| 0.459896093 | 0.969041349 | 0.605650843 | 0.473133585 |
| 0.490498897 | 0.972457476 | 0.728987261 | 0.59767988 |
| 0.484876521 | 0.968756672 | 0.631414134 | 0.669632055 |
| 0.479111807 | 0.950822006 | 0.206106327 | 0.602305886 |
| 0.4591844 | 0.955234503 | 0.456551135 | 0.813251726 |
| 0.463169881 | 0.964059498 | 0.426446516 | 0.720731621 |
| 0.426304178 | 0.940573625 | 0.781439043 | 0.637890542 |
| 0.430076151 | 0.951818376 | 0.509002918 | 0.488221479 |
| 0.496761796 | 0.948188741 | 0.417336844 | 0.693046758 |
| 0.449790051 | 0.967689132 | 0.540103907 | 0.673759875 |
| 0.393068109 | 0.962351434 | 0.549427087 | 0.730908832 |
| 0.406803786 | 0.972884492 | 0.840509572 | 0.68151733 |
| 0.396697744 | 0.957796598 | 0.724859441 | 0.621663939 |
| 0.400327379 | 0.956302043 | 0.516760373 | 0.584869404 |
| 0.343605437 | 0.980997794 | 0.77659953 | 0.590634119 |
| 0.473418262 | 0.979859085 | 0.697672764 | 0.741584229 |
| 0.415130596 | 0.978079852 | 0.673617536 | 0.667354637 |
| 0.392000569 | 0.95238773 | 0.583659526 | 0.671268949 |
| 0.399544516 | 0.977510497 | 0.741584229 | 0.559177283 |
| 0.452779162 | 0.976656466 | 0.807415842 | 0.625151235 |
| 0.403601167 | 0.951818376 | 0.638531065 | 0.508362394 |
| 0.417621522 | 0.96377482 | 0.500889616 | 0.559319621 |
| 0.414134225 | 0.963205466 | 0.602234716 | 0.629563732 |
| 0.438331791 | 0.957013736 | 0.524660167 | 0.848195858 |
| 0.412853178 | 0.966977439 | 0.581951463 | 0.610561526 |

| 0.462458188 | 0.964628852 | 0.422816881 | 0.740303181 |
| --- | --- | --- | --- |
| 0.411287453 | 0.953526439 | 0.568642801 | 0.554551277 |
| 0.451142267 | 0.932673831 | 0.261760729 | 0.452565654 |
| 0.437548929 | 0.949042773 | 0.646502028 | 0.495765426 |
| 0.455768273 | 0.945982492 | 0.50772187 | 0.515906341 |
| 0.533413992 | 0.945484307 | 0.382819728 | 0.40431286 |
| 0.513557754 | 0.976514127 | 0.74144189 | 0.610205679 |
| 0.526581738 | 0.955803857 | 0.65041634 | 0.625507081 |
| 0.506440823 | 0.968044979 | 0.610490357 | 0.668564515 |
| 0.487225109 | 0.980072593 | 0.668279838 | 0.671624795 |
| 0.536830119 | 0.98142481 | 0.58949541 | 0.693829621 |
| 0.488719664 | 0.963703651 | 0.490783574 | 0.664365526 |
| 0.428581596 | 0.954024625 | 0.574905701 | 0.65874315 |
| 0.428866273 | 0.984769767 | 0.708632838 | 0.735178991 |
| 0.442957797 | 0.964130667 | 0.554693616 | 0.686855028 |
| 0.499608569 | 0.960643371 | 0.596541171 | 0.6349726 |
| 0.461105971 | 0.955875027 | 0.567148246 | 0.582022632 |
| 0.39506085 | 0.957084905 | 0.45192513 | 0.471425521 |
| 0.468649918 | 0.920432709 | 0.593836738 | 0.775531991 |
| 0.398476977 | 0.959931677 | 0.688136076 | 0.731905202 |
| 0.461746495 | 0.963347804 | 0.72592698 | 0.684506441 |
| 0.474983987 | 0.920290371 | 0.245747634 | 0.780940858 |
| 0.405238061 | 0.974877233 | 0.631200626 | 0.743719308 |
| 0.427229379 | 0.923991175 | 0.225677888 | 0.81595616 |
| 0.450145897 | 0.973525016 | 0.681659668 | 0.540032738 |
| 0.42196285 | 0.957938937 | 0.398049961 | 0.649775817 |
| 0.469076934 | 0.94833108 | 0.454131379 | 0.618532489 |
| 0.391146538 | 0.961283894 | 0.674969753 | 0.575190378 |
| 0.39093303 | 0.944274429 | 0.704149171 | 0.466657177 |
| 0.430503167 | 0.97210163 | 0.715180414 | 0.551562166 |
| 0.465874315 | 0.966692762 | 0.656394563 | 0.645861505 |
| 0.456764643 | 0.976656466 | 0.809337414 | 0.781083197 |
| 0.520105331 | 0.968258487 | 0.705216711 | 0.544658743 |
| 0.465660807 | 0.962351434 | 0.494057362 | 0.669489716 |
| 0.436552559 | 0.946124831 | 0.3847413 | 0.59355206 |
| 0.479609992 | 0.961853249 | 0.787132588 | 0.515479325 |
| 0.488363818 | 0.966835101 | 0.339335279 | 0.700661875 |
| 0.404526368 | 0.961995588 | 0.76627998 | 0.584157711 |
| 0.443740659 | 0.967760302 | 0.615187531 | 0.658814319 |
| 0.451355775 | 0.968329656 | 0.512774891 | 0.517472066 |
| 0.571631912 | 0.966052238 | 0.289018575 | 0.705999573 |
| 0.475197495 | 0.944345598 | 0.474129955 | 0.733897943 |
| 0.571062558 | 0.955946196 | 0.763717885 | 0.585936944 |
| 0.432567077 | 0.952103053 | 0.494769056 | 0.769482599 |
| 0.548928902 | 0.973311508 | 0.694683652 | 0.717173155 |
| 0.45092876 | 0.95658672 | 0.543591203 | 0.863212583 |
| 0.518895452 | 0.970393566 | 0.683438901 | 0.728489076 |
| 0.509429934 | 0.970820582 | 0.601594193 | 0.708704007 |
| 0.513486585 | 0.957511921 | 0.602377055 | 0.561668209 |
| 0.542238987 | 0.973026831 | 0.668422176 | 0.696462885 |

| 0.413493702 | 0.952245392 | 0.541954309 | 0.601451854 |
| --- | --- | --- | --- |
| 0.461960003 | 0.943562736 | 0.606362536 | 0.606931891 |
| 0.543021849 | 0.947192371 | 0.652836097 | 0.522382749 |
| 0.448224326 | 0.952743577 | 0.498327521 | 0.643014732 |
| 0.4529215 | 0.957084905 | 0.70215643 | 0.782435414 |
| 0.527933955 | 0.942424027 | 0.637250018 | 0.692263896 |
| 0.466230162 | 0.969397196 | 0.6618746 | 0.568073447 |
| 0.371574977 | 0.957084905 | 0.52843214 | 0.641093161 |
| 0.475055156 | 0.963134296 | 0.579674045 | 0.633478044 |
| 0.560244822 | 0.966408085 | 0.743363462 | 0.649348801 |
| 0.519749484 | 0.969610704 | 0.627357483 | 0.584157711 |
| 0.464735606 | 0.974806064 | 0.759163049 | 0.625435912 |
| 0.513059569 | 0.957369582 | 0.498896876 | 0.604369796 |
| 0.500177923 | 0.970180058 | 0.558679098 | 0.687780229 |
| 0.46843641 | 0.953882286 | 0.547434346 | 0.475766849 |
| 0.383246744 | 0.932175646 | 0.477973098 | 0.793893673 |
| 0.53775532 | 0.941498826 | 0.464806775 | 0.560743008 |
| 0.466870685 | 0.946907693 | 0.79318198 | 0.583374849 |
| 0.528787987 | 0.935805281 | 0.583303679 | 0.54088677 |
| 0.502313003 | 0.93480891 | 0.505088606 | 0.646217351 |
| 0.465945484 | 0.941356487 | 0.329727422 | 0.70429151 |
| 0.487225109 | 0.974379048 | 0.478257775 | 0.585154082 |
| 0.465803146 | 0.959006476 | 0.638958081 | 0.689345954 |
| 0.583944203 | 0.928261334 | 0.379332432 | 0.535976087 |
| 0.527506939 | 0.930325244 | 0.470429151 | 0.539249875 |
| 0.488007971 | 0.967831471 | 0.811543662 | 0.551419828 |
| 0.486157569 | 0.950466159 | 0.549925272 | 0.715038076 |
| 0.513415415 | 0.977652836 | 0.779090456 | 0.637250018 |
| 0.558109743 | 0.960856879 | 0.468294072 | 0.763362038 |
| 0.471140844 | 0.950466159 | 0.677887695 | 0.529926696 |
| 0.453775532 | 0.938723223 | 0.457262828 | 0.585011743 |
| 0.461675326 | 0.930823429 | 0.471354352 | 0.615899224 |
| 0.55839442 | 0.920290371 | 0.488862003 | 0.670272578 |
| 0.433634617 | 0.94825991 | 0.559034944 | 0.669347377 |
| 0.506298484 | 0.947832894 | 0.430431998 | 0.429862643 |
| 0.387872749 | 0.92662444 | 0.553910754 | 0.539178706 |
| 0.473916447 | 0.973169169 | 0.584727066 | 0.542452494 |
| 0.444238844 | 0.952031884 | 0.626147605 | 0.539321045 |
| 0.373496548 | 0.95039499 | 0.500889616 | 0.628496192 |
| 0.401323749 | 0.955092164 | 0.600740161 | 0.50772187 |
| 0.480677532 | 0.972884492 | 0.566080706 | 0.627499822 |
| 0.469005765 | 0.931321614 | 0.379830617 | 0.477973098 |
| 0.451996299 | 0.962422603 | 0.495480749 | 0.730624155 |
| 0.403601167 | 0.958792968 | 0.775247313 | 0.470429151 |
| 0.379047755 | 0.946338339 | 0.394206818 | 0.608924632 |
| 0.432424738 | 0.974521386 | 0.853747064 | 0.606860722 |
| 0.404170522 | 0.943491566 | 0.649989325 | 0.57084905 |
| 0.483310796 | 0.960216355 | 0.664223187 | 0.723293716 |
| 0.394420326 | 0.946338339 | 0.506156145 | 0.493630347 |
| 0.483453135 | 0.958152445 | 0.637748203 | 0.617678457 |

| 0.42295922 | 0.950822006 | 0.438829977 | 0.587502669 |
| --- | --- | --- | --- |
| 0.464450929 | 0.954309302 | 0.534766209 | 0.649846986 |
| 0.462315849 | 0.966763931 | 0.904348445 | 0.674115721 |
| 0.396341897 | 0.967333286 | 0.470073304 | 0.638317557 |
| 0.426446516 | 0.957084905 | 0.462315849 | 0.819656964 |
| 0.525229521 | 0.945911323 | 0.512063198 | 0.770194292 |
| 0.526724077 | 0.929827059 | 0.605152658 | 0.862073874 |
| 0.468721087 | 0.97416554 | 0.733328589 | 0.747562451 |
| 0.481673902 | 0.960216355 | 0.617464949 | 0.797736816 |
| 0.451071098 | 0.864066614 | 0.594406092 | 0.733542097 |
| 0.46850758 | 0.925699238 | 0.668493346 | 0.771048324 |
| 0.552914383 | 0.896448651 | 0.664792541 | 0.832396271 |
| 0.500462601 | 0.913031101 | 0.4188314 | 0.792968472 |
| 0.460251939 | 0.951320191 | 0.669916732 | 0.739520319 |
| 0.512347876 | 0.948615757 | 0.644580457 | 0.790691054 |
| 0.455554765 | 0.910682514 | 0.435200342 | 0.58842787 |
| 0.448081987 | 0.940288947 | 0.571774251 | 0.77247171 |
| 0.476478542 | 0.925485731 | 0.625151235 | 0.884349868 |
| 0.339335279 | 0.903067397 | 0.421607003 | 0.812255355 |
| 0.430645506 | 0.935591773 | 0.453135008 | 0.834958366 |
| 0.418617892 | 0.944914953 | 0.76627998 | 0.64009679 |
| 0.496263611 | 0.890968614 | 0.612554267 | 0.780015657 |
| 0.548430717 | 0.905700662 | 0.312077432 | 0.736815885 |
| 0.488221479 | 0.852608355 | 0.40431286 | 0.874670842 |
| 0.45299267 | 0.913742794 | 0.665788912 | 0.776243684 |
| 0.422176358 | 0.939363746 | 0.610490357 | 0.787274927 |
| 0.42915095 | 0.959362323 | 0.673688705 | 0.696534055 |
| 0.588926055 | 0.945341969 | 0.491851114 | 0.786847911 |
| 0.518397267 | 0.914098641 | 0.483595474 | 0.774677959 |
| 0.559390791 | 0.922994805 | 0.532346452 | 0.785353356 |
| 0.556472849 | 0.917585937 | 0.624083695 | 0.622233293 |
| 0.51398477 | 0.91730126 | 0.296989538 | 0.746779589 |
| 0.406163262 | 0.863568429 | 0.387445733 | 0.729200769 |
| 0.457405167 | 0.966408085 | 0.702939293 | 0.84278699 |
| 0.438047114 | 0.958294783 | 0.744359832 | 0.799587218 |
| 0.593267383 | 0.895167604 | 0.499039214 | 0.704149171 |
| 0.557469219 | 0.885061561 | 0.423528574 | 0.726923351 |
| 0.454131379 | 0.877019429 | 0.665148388 | 0.618745997 |
| 0.407301971 | 0.897872038 | 0.388940289 | 0.751476763 |
| 0.433634617 | 0.898299053 | 0.296704861 | 0.766208811 |
| 0.381182834 | 0.894740588 | 0.401821934 | 0.720304605 |
| 0.441747918 | 0.882214789 | 0.617180272 | 0.79417835 |
| 0.481460394 | 0.888477688 | 0.503380542 | 0.760799943 |
| 0.504448082 | 0.897018006 | 0.264963348 | 0.769482599 |
| 0.478400114 | 0.951604868 | 0.702441107 | 0.783360615 |
| 0.431499537 | 0.899366593 | 0.335420966 | 0.694114298 |
| 0.482741442 | 0.955092164 | 0.571204896 | 0.933172016 |
| 0.486299907 | 0.913813963 | 0.473489431 | 0.699523166 |
| 0.420397125 | 0.815315636 | 0.282470999 | 0.76215216 |
| 0.504732759 | 0.933172016 | 0.47363177 | 0.884421038 |

| 0.425236638 | 0.951035513 | 0.670913102 | 0.702796954 |
| --- | --- | --- | --- |
| 0.539107537 | 0.941641164 | 0.391502384 | 0.793253149 |
| 0.499537399 | 0.95865063 | 0.694256637 | 0.735321329 |
| 0.465233791 | 0.939790762 | 0.708704007 | 0.63084478 |
| 0.486442246 | 0.918368799 | 0.451284606 | 0.789267668 |
| 0.412283823 | 0.942779873 | 0.466799516 | 0.693260266 |
| 0.55633051 | 0.932104477 | 0.616041563 | 0.752544303 |
| 0.514127108 | 0.901430503 | 0.533129315 | 0.633051028 |
| 0.440751548 | 0.959219984 | 0.621592769 | 0.726852181 |
| 0.481175717 | 0.951177852 | 0.649562309 | 0.743434631 |
| 0.453846701 | 0.875453704 | 0.395274358 | 0.567788769 |
| 0.464806775 | 0.909543805 | 0.502313003 | 0.805423102 |
| 0.509287595 | 0.85417408 | 0.360259056 | 0.768415059 |
| 0.473774109 | 0.882784143 | 0.362322966 | 0.837449292 |
| 0.476834389 | 0.933385524 | 0.625435912 | 0.810120276 |
| 0.50160131 | 0.969112519 | 0.727563874 | 0.703081631 |
| 0.503736389 | 0.910896022 | 0.310796385 | 0.832965625 |
| 0.468649918 | 0.924204683 | 0.36196712 | 0.696818732 |
| 0.431641876 | 0.896306313 | 0.487367447 | 0.627286314 |
| 0.418404384 | 0.936232297 | 0.454202548 | 0.787417266 |
| 0.48081987 | 0.884634546 | 0.566009537 | 0.774962636 |
| 0.522453918 | 0.949612127 | 0.575688563 | 0.783787631 |
| 0.548644225 | 0.906127678 | 0.677887695 | 0.839157355 |
| 0.494911394 | 0.952957085 | 0.658529642 | 0.617963134 |
| 0.490285389 | 0.890541598 | 0.224610348 | 0.716247954 |
| 0.471710199 | 0.923991175 | 0.47057149 | 0.686428012 |
| 0.440822717 | 0.923421821 | 0.381752188 | 0.696534055 |
| 0.399259839 | 0.955803857 | 0.734965483 | 0.785139848 |
| 0.505444452 | 0.920290371 | 0.408013665 | 0.712760658 |
| 0.423884421 | 0.956373212 | 0.705857234 | 0.720375774 |
| 0.470998505 | 0.933029678 | 0.39299694 | 0.54188314 |
| 0.499252722 | 0.911963561 | 0.685075795 | 0.554693616 |
| 0.478115437 | 0.939719593 | 0.530638389 | 0.710839086 |
| 0.450217066 | 0.908689773 | 0.602163547 | 0.668208668 |
| 0.559177283 | 0.933527863 | 0.567788769 | 0.815030959 |
| 0.588570209 | 0.960928048 | 0.518468436 | 0.724503594 |
| 0.455412426 | 0.920788556 | 0.070172941 | 0.855668636 |
| 0.653334282 | 0.939221408 | 0.270656893 | 0.678172372 |
| 0.555689986 | 0.942993381 | 0.410006405 | 0.520532346 |
| 0.586008113 | 0.958864138 | 0.442174934 | 0.787844282 |
| 0.663084478 | 0.956800228 | 0.49740232 | 0.644509288 |
| 0.641733684 | 0.925129884 | 0.243683724 | 0.699451996 |
| 0.573482314 | 0.96797381 | 0.333784072 | 0.622944986 |
| 0.571418404 | 0.931392783 | 0.270087538 | 0.710839086 |
| 0.523521458 | 0.951249021 | 0.299195787 | 0.905415985 |
| 0.528147463 | 0.924987545 | 0.143833179 | 0.837235784 |
| 0.66493488 | 0.937584514 | 0.394064479 | 0.687495552 |
| 0.658885489 | 0.941000641 | 0.202263184 | 0.769980784 |
| 0.520603516 | 0.967404455 | 0.220909544 | 0.726282827 |
| 0.468934595 | 0.976087111 | 0.33613266 | 0.62465305 |

| 0.589637748 | 0.940573625 | 0.403174151 | 0.633051028 |
| --- | --- | --- | --- |
| 0.586790976 | 0.97829336 | 0.4726354 | 0.717600171 |
| 0.622873817 | 0.970891751 | 0.663511494 | 0.710340901 |
| 0.604583304 | 0.972528646 | 0.482314426 | 0.764073731 |
| 0.711835457 | 0.979787915 | 0.712333642 | 0.747562451 |
| 0.590349441 | 0.974307878 | 0.593765568 | 0.870614191 |
| 0.569568002 | 0.946409508 | 0.421678172 | 0.821436197 |
| 0.67425806 | 0.960999217 | 0.596612341 | 0.744431001 |
| 0.559177283 | 0.965767561 | 0.647640737 | 0.707992314 |
| 0.637748203 | 0.968400826 | 0.606220198 | 0.843498683 |
| 0.56358978 | 0.970820582 | 0.535406733 | 0.698811472 |
| 0.444594691 | 0.976158281 | 0.594334923 | 0.824496477 |
| 0.604725642 | 0.952031884 | 0.390577183 | 0.710127393 |
| 0.656536901 | 0.988257064 | 0.681659668 | 0.71560743 |
| 0.562095225 | 0.960643371 | 0.490570066 | 0.711337271 |
| 0.461604156 | 0.935947619 | 0.180058359 | 0.769909615 |
| 0.531990606 | 0.95445164 | 0.197210163 | 0.767489858 |
| 0.614831685 | 0.906839371 | 0.319621379 | 0.73425379 |
| 0.532773468 | 0.907622233 | 0.192512988 | 0.680165113 |
| 0.425094299 | 0.939150238 | 0.084620312 | 0.725642303 |
| 0.721656822 | 0.969753042 | 0.479325315 | 0.635470785 |
| 0.44260195 | 0.912959932 | 0.089175148 | 0.821222689 |
| 0.481389225 | 0.921856096 | 0.177780941 | 0.667923991 |
| 0.578392997 | 0.946196 | 0.237634332 | 0.6753256 |
| 0.453135008 | 0.957013736 | 0.322823998 | 0.721941499 |
| 0.467297701 | 0.945128461 | 0.476905558 | 0.721229806 |
| 0.565155505 | 0.940573625 | 0.213507935 | 0.73112234 |
| 0.519322468 | 0.944914953 | 0.134296491 | 0.64016796 |
| 0.430787844 | 0.942210519 | 0.280478258 | 0.748487652 |
| 0.473987617 | 0.923279482 | 0.14269447 | 0.606860722 |
| 0.631698811 | 0.971674614 | 0.687210875 | 0.665006049 |
| 0.532275283 | 0.972243968 | 0.348302612 | 0.697388086 |
| 0.592626859 | 0.956657889 | 0.372357839 | 0.691338695 |
| 0.461390648 | 0.96377482 | 0.351291723 | 0.802861006 |
| 0.463454558 | 0.940288947 | 0.171233364 | 0.706995943 |
| 0.534766209 | 0.96277845 | 0.315778236 | 0.648210092 |
| 0.410362252 | 0.923350651 | 0.101914454 | 0.806561811 |
| 0.475126326 | 0.960501032 | 0.246815173 | 0.800939435 |
| 0.54501459 | 0.931108106 | 0.244253078 | 0.727848552 |
| 0.549711764 | 0.962707281 | 0.457191659 | 0.803999715 |
| 0.589281902 | 0.94833108 | 0.248665575 | 0.781510213 |
| 0.665504235 | 0.949754466 | 0.459397908 | 0.671411287 |
| 0.627642161 | 0.948615757 | 0.411643299 | 0.589353071 |
| 0.446018077 | 0.927549641 | 0.116077148 | 0.673973383 |
| 0.548573055 | 0.949398619 | 0.591843997 | 0.74564088 |
| 0.462102341 | 0.937798021 | 0.110312433 | 0.934524233 |
| 0.512846061 | 0.942851043 | 0.11892392 | 0.737456409 |
| 0.475482172 | 0.946409508 | 0.209451285 | 0.722012668 |
| 0.590776457 | 0.94519963 | 0.344530638 | 0.716532631 |
| 0.705216711 | 0.976941143 | 0.601167177 | 0.704006832 |

| 0.698526795 | 0.959504662 | 0.420824141 | 0.716888478 |
| --- | --- | --- | --- |
| 0.481673902 | 0.950608498 | 0.190093232 | 0.818945271 |
| 0.506511992 | 0.960856879 | 0.286669988 | 0.770265462 |
| 0.462671696 | 0.938580884 | 0.078072735 | 0.823927123 |
| 0.465091453 | 0.948686926 | 0.127250729 | 0.886769625 |
| 0.491851114 | 0.884563376 | 0.137214433 | 0.814176927 |
| 0.525229521 | 0.938865561 | 0.521386378 | 0.717884848 |
| 0.722155007 | 0.930325244 | 0.303679453 | 0.783004768 |
| 0.494982564 | 0.916660736 | 0.133655968 | 0.81382108 |
| 0.508647071 | 0.934595402 | 0.388584442 | 0.727706213 |
| 0.582805494 | 0.961639741 | 0.626361113 | 0.768842075 |
| 0.442744289 | 0.925485731 | 0.095224539 | 0.738879795 |
| 0.554408939 | 0.958792968 | 0.420966479 | 0.695253007 |
| 0.614618177 | 0.964700021 | 0.535050886 | 0.900078286 |
| 0.54287951 | 0.974094371 | 0.655825208 | 0.793893673 |
| 0.583161341 | 0.916731905 | 0.138139634 | 0.524161981 |
| 0.508718241 | 0.942566365 | 0.120845491 | 0.832965625 |
| 0.736602377 | 0.972955662 | 0.602234716 | 0.721941499 |
| 0.562237563 | 0.948686926 | 0.169667639 | 0.707494129 |
| 0.629706071 | 0.925983916 | 0.104690058 | 0.662657462 |
| 0.541669632 | 0.917657106 | 0.166322682 | 0.695680023 |
| 0.620098214 | 0.974307878 | 0.697245748 | 0.91516618 |
| 0.524446659 | 0.922994805 | 0.121628354 | 0.760372927 |
| 0.711052594 | 0.936018789 | 0.244466586 | 0.829905345 |
| 0.466941855 | 0.890328091 | 0.097288449 | 0.879652694 |
| 0.423742082 | 0.924204683 | 0.094370507 | 0.841861789 |
| 0.468649918 | 0.917443598 | 0.090883211 | 0.905985339 |
| 0.780086827 | 0.963134296 | 0.431641876 | 0.856024482 |
| 0.701871753 | 0.944416767 | 0.412070315 | 0.724788271 |
| 0.786634403 | 0.902071027 | 0.364244538 | 0.716817308 |
| 0.476549712 | 0.928546011 | 0.073873746 | 0.88933172 |
| 0.484947691 | 0.910255498 | 0.109173724 | 0.832823287 |
| 0.667852822 | 0.960785709 | 0.44772614 | 0.791687424 |
| 0.729485446 | 0.938723223 | 0.491993452 | 0.699594335 |
| 0.523734965 | 0.976442958 | 0.605793182 | 0.732332218 |
| 0.695253007 | 0.952174222 | 0.40232012 | 0.651270372 |
| 0.653405452 | 0.959647 | 0.399331008 | 0.773254573 |
| 0.718240695 | 0.945555476 | 0.586648637 | 0.712333642 |
| 0.645505658 | 0.927763149 | 0.159348089 | 0.599886129 |
| 0.587858515 | 0.934737741 | 0.162123692 | 0.655398192 |
| 0.556828695 | 0.954380471 | 0.553626076 | 0.695537684 |
| 0.470429151 | 0.943562736 | 0.312077432 | 0.864707138 |
| 0.505017436 | 0.950893175 | 0.567005907 | 0.62258914 |
| 0.463027543 | 0.918724646 | 0.167319052 | 0.704860864 |
| 0.558750267 | 0.914525657 | 0.208099068 | 0.595046616 |
| 0.683937086 | 0.960216355 | 0.620098214 | 0.608924632 |
| 0.478400114 | 0.930609921 | 0.143192655 | 0.752900149 |
| 0.555974664 | 0.91723009 | 0.658956658 | 0.74044552 |
| 0.726496335 | 0.898441392 | 0.323891538 | 0.725001779 |
| 0.749626361 | 0.906839371 | 0.477047897 | 0.729841292 |

| 0.5470785 | 0.922994805 | 0.086399545 | 0.806561811 |
| --- | --- | --- | --- |
| 0.738310441 | 0.932602662 | 0.3112234 | 0.796526938 |
| 0.535122055 | 0.953811117 | 0.488862003 | 0.80556544 |
| 0.698170949 | 0.967689132 | 0.611771404 | 0.596185325 |
| 0.569140986 | 0.958792968 | 0.352857448 | 0.807771689 |
| 0.493701516 | 0.9390079 | 0.445875738 | 0.683652409 |
| 0.395701374 | 0.941854672 | 0.106326952 | 0.754964059 |
| 0.490712405 | 0.923706498 | 0.20575048 | 0.694185467 |
| 0.573695822 | 0.950466159 | 0.66593125 | 0.766635827 |
| 0.507437193 | 0.950893175 | 0.468080564 | 0.744644509 |
| 0.69909615 | 0.95032382 | 0.652764928 | 0.706497758 |
| 0.575475055 | 0.975802434 | 0.725357626 | 0.722083837 |
| 0.456479966 | 0.950822006 | 0.156074301 | 0.707992314 |
| 0.517116219 | 0.929186535 | 0.249946623 | 0.724503594 |
| 0.499039214 | 0.969397196 | 0.495978934 | 0.713187673 |
| 0.618105473 | 0.955590349 | 0.497046474 | 0.740018504 |
| 0.476407373 | 0.960287524 | 0.570279695 | 0.716105615 |
| 0.47256423 | 0.930894598 | 0.136929756 | 0.828481959 |
| 0.441747918 | 0.934310725 | 0.283609707 | 0.692762081 |
| 0.611913743 | 0.939292577 | 0.494484378 | 0.54287951 |
| 0.516831542 | 0.970322397 | 0.229734538 | 0.647569568 |
| 0.512205537 | 0.953739947 | 0.490000712 | 0.721727991 |
| 0.658600811 | 0.963490143 | 0.50985695 | 0.761298128 |
| 0.512846061 | 0.899081916 | 0.249661946 | 0.757526155 |
| 0.507223685 | 0.984058074 | 0.603444595 | 0.751120917 |
| 0.492989823 | 0.93068109 | 0.549782934 | 0.722724361 |
| 0.524090812 | 0.965340545 | 0.572984129 | 0.718596541 |
| 0.650060494 | 0.93174863 | 0.288022205 | 0.763077361 |
| 0.571062558 | 0.96277845 | 0.549640595 | 0.702085261 |
| 0.741014874 | 0.978435699 | 0.628923208 | 0.849832752 |
| 0.711977795 | 0.94107181 | 0.495409579 | 0.808981567 |
| 0.507579532 | 0.909899651 | 0.096932603 | 0.813963419 |
| 0.657817949 | 0.961924418 | 0.363888691 | 0.641306669 |
| 0.514625294 | 0.967475625 | 0.561810547 | 0.733684435 |
| 0.656465732 | 0.927834318 | 0.151092449 | 0.737527578 |
| 0.65461533 | 0.935805281 | 0.553697246 | 0.723436054 |
| 0.517756743 | 0.959575831 | 0.422888051 | 0.667141129 |
| 0.534979717 | 0.957156074 | 0.476834389 | 0.795103551 |
| 0.533912177 | 0.951035513 | 0.316347591 | 0.768557398 |
| 0.463668066 | 0.96583873 | 0.584228881 | 0.720589282 |
| 0.467226532 | 0.911394207 | 0.174862999 | 0.685787488 |
| 0.621663939 | 0.949540958 | 0.369653405 | 0.722653192 |
| 0.431357199 | 0.954949826 | 0.522026902 | 0.694754822 |
| 0.455270088 | 0.944345598 | 0.332289517 | 0.6753256 |
| 0.567077076 | 0.967190947 | 0.49227813 | 0.767062843 |
| 0.481246886 | 0.970962921 | 0.561881717 | 0.797167461 |
| 0.536403103 | 0.947334709 | 0.353924988 | 0.791545086 |
| 0.577396627 | 0.947761725 | 0.242758523 | 0.661020568 |
| 0.615401039 | 0.970251228 | 0.58636396 | 0.752259626 |
| 0.597324034 | 0.93587645 | 0.502028325 | 0.750978578 |

| 0.374208241 | 0.957796598 | 0.186463597 | 0.794463028 |
| --- | --- | --- | --- |
| 0.412497331 | 0.921927265 | 0.096505587 | 0.824923493 |
| 0.566223045 | 0.95238773 | 0.217351078 | 0.74969753 |
| 0.509572272 | 0.931036937 | 0.545726283 | 0.724788271 |
| 0.544658743 | 0.946907693 | 0.248452067 | 0.818304747 |
| 0.616824425 | 0.943776244 | 0.324176215 | 0.727208028 |
| 0.560814177 | 0.956159704 | 0.405166892 | 0.629919579 |
| 0.485872892 | 0.945626646 | 0.252935734 | 0.767632197 |
| 0.651270372 | 0.94213935 | 0.389011458 | 0.724432425 |
| 0.496263611 | 0.974379048 | 0.681090314 | 0.73318625 |
| 0.677887695 | 0.948188741 | 0.49740232 | 0.737100562 |
| 0.653405452 | 0.971176429 | 0.637677034 | 0.687353213 |
| 0.433207601 | 0.893673048 | 0.087538254 | 0.856024482 |
| 0.477403744 | 0.918795815 | 0.102981994 | 0.804711408 |
| 0.515123479 | 0.960785709 | 0.371930823 | 0.834744858 |
| 0.50359405 | 0.966052238 | 0.664009679 | 0.807771689 |
| 0.512703722 | 0.955305672 | 0.304675824 | 0.630346595 |
| 0.500747278 | 0.938296207 | 0.453846701 | 0.594548431 |
| 0.552985553 | 0.963917159 | 0.588712547 | 0.746708419 |
| 0.643939933 | 0.941641164 | 0.378193723 | 0.729841292 |
| 0.35776813 | 0.955590349 | 0.10027756 | 0.83552772 |

| **Supplementary Table 8. The changes of Cupclusters, gene clusters and CupScore.** | | | |
| --- | --- | --- | --- |
| id | Cupcluster | gene.cluster | CupScore |
| FB006 | A | A | -1.7074675 |
| FB017 | A | A | -0.273146 |
| FB403 | A | A | -3.9084366 |
| FB404 | A | A | -2.7445049 |
| FB405 | A | A | -1.2935751 |
| FB408 | A | A | -1.4433298 |
| FB409 | A | A | -1.2923348 |
| FB410 | A | A | -3.2181494 |
| FB417 | A | A | -1.2695849 |
| FB433 | A | A | -2.5989952 |
| FB435 | A | A | -2.6133107 |
| FB438 | A | A | -3.0582533 |
| FB439 | A | A | -4.0325942 |
| FB502 | A | A | -2.7550177 |
| FB506 | A | A | -0.7334673 |
| FB555 | A | A | -1.8664647 |
| FB556 | A | A | -3.4104692 |
| FB557 | A | A | -2.106614 |
| FB559 | A | A | -4.6627283 |
| FB561 | A | A | -2.7730929 |
| FB564 | A | A | -4.5023287 |
| FB566 | A | A | -6.4333173 |
| FB567 | A | A | -2.4210067 |
| FB568 | A | A | -2.9135856 |
| FB569 | A | A | -5.0208979 |
| GSM405215 | C | B | 3.37857291 |
| GSM405218 | B | C | 3.60193166 |
| GSM405222 | B | B | 1.00376266 |
| GSM405230 | B | C | 2.58042854 |
| GSM405233 | B | C | 2.72999731 |
| GSM405243 | B | B | 1.51773639 |
| GSM405247 | C | B | 5.59050094 |
| GSM405251 | B | A | -1.090935 |
| GSM405252 | C | C | 2.28666186 |
| GSM405254 | B | C | 3.71785732 |
| GSM405264 | C | C | 3.23379389 |
| GSM405267 | B | B | 2.68273576 |
| GSM405268 | B | B | 2.48785994 |
| GSM405293 | B | C | 2.88816904 |
| GSM405294 | B | B | 4.49431549 |
| GSM405296 | B | B | 2.23692853 |
| GSM405299 | B | C | 2.5946835 |
| GSM405301 | C | C | 2.92429722 |
| GSM405304 | C | C | 2.96541084 |
| GSM405305 | C | C | 1.75844487 |
| GSM405308 | C | B | -1.0894959 |
| GSM405312 | C | B | 2.77749943 |
| GSM405314 | C | C | 2.94224896 |

| GSM405315 | B | A | -2.3395765 |
| --- | --- | --- | --- |
| GSM405322 | C | C | 2.34067147 |
| GSM405323 | C | B | 2.89143048 |
| GSM405326 | C | C | 3.86783448 |
| GSM405330 | B | B | 3.68957796 |
| GSM405337 | B | B | 2.5908857 |
| GSM405350 | C | A | -0.3258155 |
| GSM405352 | B | B | 3.11681025 |
| GSM405362 | B | C | 1.54133234 |
| GSM405363 | B | B | 1.23518718 |
| GSM405365 | B | B | 0.34270583 |
| GSM405371 | B | C | 1.24142482 |
| GSM405372 | C | B | 2.81897978 |
| GSM405385 | B | B | -0.1838451 |
| GSM405389 | C | B | -0.8232106 |
| GSM405393 | C | C | 2.61390638 |
| GSM405397 | C | B | 2.22093543 |
| GSM405412 | C | C | 3.44914992 |
| GSM405416 | C | C | 3.13171412 |
| GSM405417 | C | C | 4.46234453 |
| GSM405426 | C | C | 3.23927289 |
| GSM405428 | C | C | 1.92786151 |
| GSM405432 | C | C | 3.41200219 |
| GSM405434 | C | C | 2.72946912 |
| GSM405436 | C | B | 3.49770758 |
| GSM405438 | C | C | 4.45951276 |
| GSM405442 | C | C | 3.06244061 |
| GSM405443 | C | C | 1.764772 |
| GSM405446 | C | C | 2.19450624 |
| GSM405447 | C | C | 2.45948151 |
| GSM405448 | C | C | 2.7825046 |
| GSM405452 | C | C | 2.81460583 |
| GSM405453 | C | C | 2.27494699 |
| GSM405454 | C | C | 1.22118893 |
| GSM405461 | C | B | 2.20433075 |
| GSM405463 | C | C | 2.65437809 |
| GSM405464 | C | C | 0.96056368 |
| GSM405465 | C | C | 3.90021606 |
| GSM405471 | C | C | 2.94902069 |
| GSM405473 | C | C | 2.60175121 |
| GSM405474 | C | C | 3.69911279 |
| GSM405475 | B | B | 1.27798094 |
| GSM405479 | C | C | 3.30217841 |
| GSM187159 | A | A | -1.6534621 |
| GSM187160 | A | A | -2.2980485 |
| GSM187161 | A | A | -3.1213724 |
| GSM187162 | B | A | -2.0250549 |
| GSM187163 | A | A | -3.0004142 |
| GSM187166 | B | A | -0.6951379 |
| GSM187167 | B | A | -2.6043628 |

| GSM187168 | B | A | -2.8931427 |
| --- | --- | --- | --- |
| GSM187171 | B | A | -1.0462964 |
| GSM187172 | A | A | -4.8927952 |
| GSM187174 | A | A | -2.9138103 |
| GSM187175 | B | A | -1.271489 |
| GSM187177 | B | A | -3.2988264 |
| GSM187179 | B | A | -1.9634619 |
| GSM187181 | A | A | -4.9228324 |
| GSM187182 | B | A | -0.836451 |
| GSM187186 | B | A | -0.1980116 |
| GSM187190 | B | A | -1.6738622 |
| GSM187191 | B | A | -3.9079402 |
| GSM187192 | B | A | -2.5548556 |
| GSM187193 | A | A | -0.1682754 |
| GSM187195 | A | A | -4.3726004 |
| GSM187196 | B | A | -0.7684096 |
| GSM187197 | B | A | -2.1886597 |
| GSM187198 | B | A | 0.42092287 |
| GSM187200 | A | A | -3.0507239 |
| GSM187202 | B | A | -1.7916443 |
| GSM187204 | B | A | -1.7921463 |
| GSM187208 | B | A | -2.0642207 |
| GSM187211 | B | A | -1.2703998 |
| GSM187214 | A | A | -1.9383013 |
| GSM187215 | A | A | -4.5115781 |
| GSM187217 | B | A | -2.2527577 |
| GSM187222 | B | A | -2.3973461 |
| GSM187226 | B | A | -1.9981942 |
| GSM187227 | B | A | -1.5464688 |
| GSM187228 | A | A | -4.4732441 |
| GSM187231 | B | A | 0.09328662 |
| GSM187232 | B | A | -4.1921076 |
| 900-00-53-32 | B | C | 1.30845765 |
| 900-00-5317 | B | B | -3.4941436 |
| 900-00-5338 | C | C | -0.0420674 |
| 900-00-5342 | C | C | 0.93827744 |
| 900-00-5346 | C | C | 0.46704622 |
| 900-00-5381 | B | C | 0.34364596 |
| 900-00-5384 | B | C | 1.77010497 |
| 900-00-5396 | C | C | 0.8160281 |
| 900-00-5413 | C | C | 1.40720746 |
| 900-00-5445 | C | B | -0.0902407 |
| 900-00-5458 | B | B | -0.13522 |
| 900-00-5488 | C | C | 0.39110452 |
| 900-00-5540 | B | C | -0.0570578 |
| 900-00-5542 | C | C | 1.38732851 |
| 900-00-5543 | B | C | 0.74385665 |
| 900-00-5544 | C | C | 1.48519767 |
| 900-00-5546 | C | C | 2.34404926 |
| 900-00-5548 | C | C | 1.14482021 |

| 900-00-5554 | C | B | 1.26481285 |
| --- | --- | --- | --- |
| 900-0053-03 | C | B | -0.574095 |
| E09139 | B | C | 0.7015467 |
| E09278 | C | B | -1.3328971 |
| E09348 | B | B | -2.1894753 |
| E09430 | B | C | -1.9585069 |
| E09483 | B | C | -0.2182527 |
| E09489 | C | C | 0.71348675 |
| E09535 | B | C | -0.7592923 |
| E09605 | B | C | -0.9411341 |
| E09606 | C | C | -0.8475105 |
| E09610 | B | C | 2.15900848 |
| E09615 | B | C | 0.30085343 |
| E09623 | B | C | 0.89404705 |
| E09647 | B | C | 0.42342208 |
| E09730 | B | C | -2.1476443 |
| E09744 | C | C | 1.09510756 |
| E09759 | B | C | -0.3268191 |
| E09787 | B | B | -2.3914648 |
| E09800 | C | C | 0.48553096 |
| E09810 | C | C | 0.90471754 |
| E09832 | B | C | 0.46293609 |
| E09847 | B | B | -1.9989629 |
| E09852 | B | A | -2.8033717 |
| E09951 | B | C | 1.7065176 |
| E09967 | C | C | 1.40668909 |
| E10013 | C | C | 1.55089323 |
| E10016 | B | C | -1.5363282 |
| E10026 | C | C | -1.988004 |
| E10077 | B | C | -1.4649421 |
| E10102 | C | C | -1.2867121 |
| E10110 | B | B | 1.2042745 |
| E10144 | B | B | -2.333976 |
| E10158 | B | B | -1.3947926 |
| E10184 | B | B | -3.0980768 |
| E10211 | B | B | -3.3993564 |
| E10226 | B | B | 0.40224009 |
| E10262 | C | B | 0.36151349 |
| E10267 | B | C | 2.6121817 |
| E10292 | C | C | 0.04594213 |
| E10300 | C | C | 0.94548467 |
| E10305 | C | C | 0.76309969 |
| E10444 | C | C | 1.1977609 |
| E10514 | B | B | -0.7563845 |
| E10551 | B | C | 1.64604132 |
| E50074 | B | C | -0.4860038 |
| E50091 | B | B | -0.5493014 |
| HF0024 | B | C | -0.6508038 |
| HF0048 | B | B | -2.6573127 |
| HF0180 | C | C | 2.50591982 |

| HF0268 | B | B | 0.34511042 |
| --- | --- | --- | --- |
| HF0300.3 | C | C | -0.3443781 |
| HF0442.5 | B | B | 0.61020179 |
| HF0627 | B | C | 0.30993807 |
| HF0891 | C | C | 1.07492362 |
| HF0894 | C | C | 1.51841108 |
| HF0963 | B | C | 0.65107065 |
| HF0986 | B | B | -1.4592537 |
| HF0990 | B | B | -0.7895536 |
| HF0992 | B | B | 1.06617554 |
| HF1122 | C | C | -0.9605174 |
| HF1137 | C | C | 0.91782766 |
| HF1139 | B | C | 0.81110496 |
| HF1178 | B | C | -0.4181096 |
| HF1191 | C | C | 1.06029839 |
| HF1269 | C | C | -0.1692937 |
| HF1292 | B | C | -0.8302026 |
| HF1356 | B | C | 0.76583754 |
| HF1397 | C | C | 2.31337245 |
| HF1469 | B | B | -2.3872276 |
| HF1492 | B | B | -2.8273072 |
| HF1517 | C | C | 2.26421785 |
| HF1538 | B | C | 0.94398966 |
| HF1585 | B | C | -0.8809485 |
| HF1589 | C | C | 2.08921553 |
| HF1608 | C | C | 3.24208724 |
| HF1628 | C | C | 0.77554983 |
| HF1640 | B | A | -2.9111164 |
| HF1667 | B | B | -3.1837945 |
| MD545226 | C | B | -0.2915387 |
| MD602958 | C | C | 0.44184661 |
| MD607103 | C | C | 1.37375429 |
| MD608660 | B | C | 1.40592024 |
| MD621233 | B | A | -3.3170985 |
| FB013 | A | A | -3.1901404 |
| FB108 | A | A | -0.9260191 |
| FB206 | A | A | -2.321174 |
| FB402 | A | A | -0.1727512 |
| FB406 | A | A | -4.7449223 |
| FB407 | A | A | -1.2735035 |
| FB412 | A | A | -2.3099678 |
| FB414 | A | A | -1.1150016 |
| FB416 | A | A | -4.97472 |
| FB418 | A | A | -3.8605786 |
| FB419 | A | A | -2.9112393 |
| FB420 | A | A | -2.8514464 |
| FB425 | A | A | -0.8362351 |
| FB426 | A | A | -0.7553194 |
| FB427 | A | A | -2.6169306 |
| FB434 | A | A | -3.5334795 |

| FB436 | A | A | -2.3143923 |
| --- | --- | --- | --- |
| FB503 | A | A | -3.5402578 |
| FB508 | A | A | -4.8692702 |
| FB516 | A | A | -4.5343494 |
| FB553 | A | A | -3.3882543 |
| FB554 | A | A | -0.0778397 |
| FB558 | A | A | -3.1104832 |
| GSM405213 | B | B | 1.98993981 |
| GSM405214 | B | C | -0.9816491 |
| GSM405216 | B | A | -4.512626 |
| GSM405217 | B | B | 0.88566854 |
| GSM405219 | B | C | 0.31552336 |
| GSM405220 | C | C | 0.13005113 |
| GSM405221 | C | C | 2.50496757 |
| GSM405223 | B | B | 0.783196 |
| GSM405224 | B | B | 2.25390893 |
| GSM405228 | B | B | 1.67361414 |
| GSM405229 | B | B | -1.7892889 |
| GSM405231 | C | B | -1.9418584 |
| GSM405232 | B | B | -2.9177563 |
| GSM405234 | B | B | 0.31138101 |
| GSM405235 | B | C | 2.05600515 |
| GSM405236 | B | C | 3.02861155 |
| GSM405237 | B | C | 2.75391697 |
| GSM405238 | B | B | -0.4351791 |
| GSM405239 | C | C | 2.52976777 |
| GSM405240 | B | B | -0.6211404 |
| GSM405241 | C | C | 3.34885772 |
| GSM405242 | C | B | -1.8083236 |
| GSM405244 | B | A | -1.7642154 |
| GSM405245 | B | B | -0.3935683 |
| GSM405246 | B | A | -5.8012684 |
| GSM405248 | B | B | 0.01742762 |
| GSM405249 | B | B | -3.1972013 |
| GSM405253 | B | B | -1.4529265 |
| GSM405255 | C | B | 2.3734757 |
| GSM405260 | C | C | 1.54685579 |
| GSM405262 | B | B | -0.8044303 |
| GSM405263 | B | C | 0.59315658 |
| GSM405266 | B | B | 0.51687342 |
| GSM405269 | B | C | 2.52007998 |
| GSM405270 | B | C | 0.93725928 |
| GSM405271 | B | B | 2.09276056 |
| GSM405274 | B | C | 1.24109864 |
| GSM405275 | B | B | -3.0281961 |
| GSM405276 | C | C | 4.08028221 |
| GSM405278 | B | B | 1.41952526 |
| GSM405280 | B | C | 0.25496457 |
| GSM405282 | C | C | 1.35455556 |
| GSM405290 | B | C | 1.199212 |

| GSM405292 | B | B | -1.257857 |
| --- | --- | --- | --- |
| GSM405297 | B | B | 0.42510839 |
| GSM405302 | C | B | 0.89037023 |
| GSM405303 | B | B | 1.06444512 |
| GSM405307 | C | C | 5.1987848 |
| GSM405309 | C | B | 2.96018856 |
| GSM405313 | C | B | -0.0178527 |
| GSM405317 | C | C | 5.0442752 |
| GSM405320 | C | C | 2.46282864 |
| GSM405324 | C | C | 4.03729009 |
| GSM405328 | C | C | 4.00297861 |
| GSM405340 | B | B | -2.2508934 |
| GSM405345 | C | C | 2.49350119 |
| GSM405349 | C | B | -3.0707205 |
| GSM405351 | C | C | 0.81521567 |
| GSM405353 | B | C | 4.10157073 |
| GSM405356 | B | B | -1.2505321 |
| GSM405367 | C | B | -0.5674535 |
| GSM405368 | B | B | -0.81095 |
| GSM405369 | C | B | -3.9814238 |
| GSM405370 | B | B | -4.2206028 |
| GSM405373 | C | C | 3.55352224 |
| GSM405374 | C | C | 5.05454648 |
| GSM405375 | C | C | 4.92928594 |
| GSM405376 | B | B | -0.4512813 |
| GSM405379 | C | B | 1.1961646 |
| GSM405384 | C | C | 3.35821167 |
| GSM405391 | C | C | 2.11925586 |
| GSM405392 | B | C | 1.93806691 |
| GSM405396 | C | C | 0.49980313 |
| GSM405405 | C | C | 2.38356294 |
| GSM405415 | C | C | 2.64143625 |
| GSM405418 | C | B | -2.4486101 |
| GSM405419 | C | C | -0.8850659 |
| GSM405422 | C | C | 0.8906868 |
| GSM405427 | C | C | 0.90974157 |
| GSM405430 | C | C | 3.7041608 |
| GSM405431 | C | B | -3.3980997 |
| GSM405440 | C | C | 3.64905717 |
| GSM405455 | C | C | 1.01267695 |
| GSM405458 | B | B | -0.4935395 |
| GSM405459 | C | C | 5.72294396 |
| GSM405466 | C | C | 2.75374668 |
| GSM405470 | C | B | 0.21829868 |
| GSM405472 | C | C | 3.05838712 |
| GSM405477 | C | C | 3.29437517 |
| GSM187153 | B | A | -0.2970183 |
| GSM187154 | B | A | -1.0364731 |
| GSM187155 | B | A | 0.39874666 |
| GSM187157 | B | A | -3.4138294 |

| GSM187158 | B | A | 0.52426481 |
| --- | --- | --- | --- |
| GSM187164 | B | A | -0.2147987 |
| GSM187165 | B | A | -1.765133 |
| GSM187169 | B | A | -0.9644542 |
| GSM187176 | B | A | -2.5983575 |
| GSM187178 | B | A | -0.3315507 |
| GSM187183 | B | A | -5.6453839 |
| GSM187184 | A | A | -1.4915028 |
| GSM187185 | B | A | -1.835418 |
| GSM187187 | A | A | -1.2863349 |
| GSM187188 | B | A | -1.0586694 |
| GSM187189 | B | A | -0.2444903 |
| GSM187194 | B | A | 0.4972937 |
| GSM187199 | A | A | -1.7034608 |
| GSM187201 | A | A | -0.4343357 |
| GSM187203 | B | A | -1.5817419 |
| GSM187205 | B | A | -1.6131437 |
| GSM187206 | A | A | 0.16001323 |
| GSM187207 | C | A | 0.85620912 |
| GSM187209 | B | A | 0.11508111 |
| GSM187210 | B | A | -1.2893965 |
| GSM187212 | A | A | -0.919354 |
| GSM187213 | B | A | -2.1355278 |
| GSM187216 | B | A | -4.9815087 |
| GSM187218 | A | A | -3.883002 |
| GSM187219 | B | A | 1.3549559 |
| GSM187220 | B | A | -0.8525681 |
| GSM187221 | A | A | -2.6968416 |
| GSM187223 | B | A | -1.8004721 |
| GSM187224 | A | A | -1.5691874 |
| GSM187225 | C | A | -1.5819531 |
| GSM187229 | B | A | -2.6183931 |
| GSM187230 | B | A | 1.10208258 |
| GSM187233 | B | A | -2.2908819 |
| GSM187234 | B | A | 0.5124981 |
| GSM187235 | B | A | 2.01038872 |
| GSM187236 | B | A | -2.081463 |
| 900-00-5379 | C | C | 1.97847131 |
| 900-00-5404 | B | C | 1.120918 |
| 900-00-5404- | B | C | 2.61243771 |
| 900-00-5414 | B | B | 0.20811733 |
| 900-00-5462 | C | C | 3.21144109 |
| 900-00-5489 | C | C | 1.118169 |
| 900-00-5541 | C | C | 0.66966614 |
| 900-00-5551 | C | C | 1.13466135 |
| 900-0052-99 | C | C | 3.13414089 |
| E09192 | C | B | -1.3820512 |
| E09331 | B | B | -0.4487701 |
| E09451 | B | C | -0.2339414 |
| E09454 | B | B | -1.8067485 |

| E09569 | B | B | -2.3627428 |
| --- | --- | --- | --- |
| E09601 | C | C | 2.39958171 |
| E09602 | B | C | 2.61217317 |
| E09649 | B | C | 0.64085532 |
| E09654 | B | C | 1.57867294 |
| E09670 | B | B | -1.8489445 |
| E09690 | C | B | -2.7381829 |
| E09722 | B | B | -2.5906891 |
| E09740 | C | C | 1.32744174 |
| E09782 | C | C | 1.61940931 |
| E09786 | B | B | -0.9496332 |
| E09791 | C | C | 2.23039011 |
| E09802 | B | C | -0.4140639 |
| E09833 | C | C | -0.2613576 |
| E09846 | B | A | -2.524029 |
| E09907 | C | C | -0.4032262 |
| E09917 | B | C | 1.31863401 |
| E09930 | B | B | -1.2605995 |
| E09938 | B | C | 0.0500655 |
| E09956 | B | B | -1.7959631 |
| E10002 | C | C | 1.80639348 |
| E10031 | B | C | -0.7098556 |
| E10041 | C | C | 1.76546295 |
| E10227 | C | C | 3.9293037 |
| E10252 | B | B | -3.0022643 |
| E10258 | B | B | -1.7524078 |
| E10271 | C | C | -0.5506003 |
| E10284 | C | C | 2.62608687 |
| E10290 | B | C | 1.47089911 |
| E10312 | B | B | -1.2748078 |
| E10313 | C | C | 2.48085836 |
| E10433 | B | B | 0.29366823 |
| E10462 | B | C | 0.41046347 |
| E10488 | B | C | 0.25059222 |
| E50057 | B | B | -2.0940502 |
| E50123 | C | C | 0.24913615 |
| HF0031 | C | C | 1.7573834 |
| HF0050 | B | C | 0.16219352 |
| HF0066 | B | C | 0.03293773 |
| HF0138 | C | C | 0.79135447 |
| HF0142 | C | C | 0.38966823 |
| HF0408 | C | C | 0.84090609 |
| HF0445 | C | C | 1.54863848 |
| HF0505 | B | A | -2.9252292 |
| HF0520 | C | C | 3.17346723 |
| HF0543 | C | C | 2.21351899 |
| HF0583 | C | C | 1.0878027 |
| HF0654 | B | B | 0.29141146 |
| HF0790 | C | C | 1.6954087 |
| HF0996 | B | B | -1.7394581 |

| HF1058 | B | A | -2.2156514 |
| --- | --- | --- | --- |
| HF1077 | B | C | 1.39949154 |
| HF1078 | B | C | 2.09814095 |
| HF1097 | B | C | -0.0925749 |
| HF1220 | C | C | 2.34286048 |
| HF1262 | C | C | 1.1568908 |
| HF1297 | B | C | 0.52450566 |
| HF1318 | C | C | -0.2007982 |
| HF1338 | B | B | -1.7090117 |
| HF1382 | C | C | 0.56130859 |
| HF1509 | C | C | 1.39653561 |
| HF1534 | B | B | 2.43687662 |
| HF1540 | C | C | 1.3390386 |
| HF1618 | C | C | 1.44995926 |
| HF1671 | B | B | -1.0265787 |
| MD607216 | C | C | 1.74629365 |
